# Dietary phytochemical index and the risk of cancer: A systematic review and meta-analysis

**DOI:** 10.1371/journal.pone.0319591

**Published:** 2025-04-02

**Authors:** Hamid Ahmadirad, Morteza Omrani, Nikoo Azmi, Amir Hesam Saeidian, Mitra Kazemi Jahromi, Hanifeh Mirtavoos-Mahyari, Mahdi Akbarzadeh, Farshad Teymoori, Hossein Farhadnejad, Parvin Mirmiran

**Affiliations:** 1 Nutrition and Endocrine Research Center, Research Institute for Endocrine Disorders, Research Institute for Endocrine Sciences, Shahid Beheshti University of Medical Sciences, Tehran, Iran; 2 Department of Nutrition, School of Public Health, Iran University of Medical Sciences, Tehran, Iran; 3 Department of Surgery, Rasool-E Akram Hospital School of Medicine, Iran University of Medical Sciences, Tehran, Iran; 4 Endocrinology and Metabolism Research Center, Hormozgan University of Medical Sciences, Bandar Abbas, Iran; 5 Lung Transplantation Research Center, National Research Institute of Tuberculosis and Lung Diseases, Shahid Beheshti University of Medical Sciences, Tehran, Iran; 6 Cellular and Molecular Endocrine Research Center, Research Institute for Endocrine Molecular Biology, Research Institute for Endocrine Sciences, Shahid Beheshti University of Medical Sciences, Tehran, Iran; 7 Nutritional Sciences Research Center, Iran University of Medical Sciences, Tehran, Iran; Endocrinology and Metabolism Population Sciences Institute, Tehran University of Medical Sciences, IRAN, ISLAMIC REPUBLIC OF

## Abstract

**Background:**

Recently, the association between dietary phytochemical index (DPI) and the risk of cancer has been the focus of researchers, however, this possible association has not been fully understood. The current meta-analysis aimed to assess the relationship between DPI and the risk of cancers.

**Methods:**

A literature search by the main keywords such as “dietary phytochemical index”, “DPI”, and “cancer” was completed using Scopus, PubMed, and Web of Science up to December 2024 and references of retrieved relevant articles. Observational studies examining the association between the DPI and the risk of cancers were included. The reported odds ratio (OR) with a 95% confidence interval (CI) for each study was converted into log OR, and their standard deviation was calculated. Then to compute the pooled OR, the random-effects model with inverse variance weighting method was performed.

**Results:**

Nine case-control studies were included in the present meta-analysis. The sample size ranged from 120 to 851 with an age range from 18 to 75 years. The pooled results indicate an inverse association between DPI and the risk of all cancers (OR: 0.40; 95% CI: 0.29–0.54, I^2^ = 0.00%; P-value < 0.001). Also, subgroup analysis indicated that higher a DPI score is related to the decreased risk of breast cancer (OR: 0.38; 95% CI: 0.26–0.55, I^2^ = 0.00%; P-value < 0.001) and pooled non-breast cancer including glioma, prostate, and colorectal cancers (OR: 0.43; 95% CI: 0.27–0.71, I^2^ = 0.00%; P-value = 0.001).

**Conclusions:**

The results of the current meta-analysis revealed that the higher DPI score is associated with a decreased odds of cancers. Large-scale cohort studies are recommended to validate the findings presented in the current study.

## Introduction

Cancer progression results from the cumulating of random genetic mutation and the selection of cells harboring mutations that award them a growth advantage under certain conditions. Cancer is the main cause of death worldwide, accounting for about 10 million deaths in 2020, or nearly one in six deaths [1]. In the United States, an estimated 2 million new cancer cases and 611,720 cancer deaths took place in 2024 [2].

Diet and physical activity are the main behavioral and environmental risk factors for cancer death worldwide [3]. According to estimates, lifestyle and dietary measures alone can prevent 30-40 percent of all cancers [4]. Although dietary factors are considered important in determining cancer risk, determining the precise effect of diet on cancer risk has proven challenging. However, it has been shown that a diet rich in fruits and vegetables can protect against cancer [4]. Oxidative stress, another risk factor for cancer, visibly damages DNA molecules, alters signaling pathways, and modulates the progression of many cancers [5].

Many studies have shown that a plant-based diet and certain food groups, such as fruits, vegetables, whole grains, legumes, and nuts, are anti-cancer [6]. Phytochemical compounds in these sources play an important role in biological activity, including anti-proliferative, antioxidant, and anticancer properties [7]. The activity of phytochemicals in tumor suppression is facilitated by modulation of gene expression associated with signaling pathways in different stages of carcinogenesis, progression, metastasis, and regulation of cell death [8].

As a result, a diet rich in phytochemicals can have an effective role in cancer. McCarty et al created a straightforward and functional instrument called dietary phytochemical index (DPI) to present the phytochemical content of a complete diet [9]. The DPI consists of eight components including fruits, vegetables, whole grains, legumes, nuts, soy products, seeds, olive, and olive oil. DPI is defined as the ratio of energy obtained from the mentioned eight foods (kilocalories) to the total daily energy intake multiplied by 100. Recently, studies that focused on the relationship between DPI and cancers showed that individuals with a high DPI score are likely to be less at risk of various cancers including breast cancer (BC) [10–13], colon cancer [14], benign prostatic hyperplasia [15,16], and glioma [17]. Several studies demonstrated an inverse association between DPI score and risk of various cancers [10,12–15,17], however, other studies did not observe a significant association between DPI and risk of BC [11,13].

To the best of our knowledge, no meta-analytic studies have been performed in this field, and the need for studies with pooled results has been recognized to achieve a synthesis on this topic. Therefore, in the current meta-analysis, we aimed to assess the literature of observational studies looking at the relationship between DPI and cancer risk and pool together all the evidence to explore whether a higher DPI results in lower odds of cancer.

## Materials and methods

### Search strategy

We performed a systematic review and meta-analysis of observational studies investigating the association of DPI with cancer risk. PubMed, Scopus, and Web of Science databases were searched for all relevant published articles up to December 2024. Only articles published in English were considered in this systematic review and meta-analysis. We conducted a systematic search using the following related MeSH (medical subject heading) terms and keywords: “dietary phytochemical” or “dietary phytochemical index” or “DPI” combined with “cancer” or “tumor” or “malignance” or “neoplasm” or “carcinoma” (S1 Table). This systematic review was performed according to the Preferred Reporting Items for Systematic Reviews and Meta-analysis (PRISMA) statement (S2 Table) [18]. Furthermore, the protocol of the present systematic review has been registered in PROSPERO (Number: CRD42023492239).

### Ethics statement

The study protocol was approved by the Ethics Committee of the Research Institute for Endocrine Sciences at the Shahid Beheshti University of Medical Sciences, Tehran, Iran (Ethics Code: IR.SBMU.ENDOCRINE.REC.1403.018).

### Inclusion and exclusion criteria

All identified papers were exported to Endnote software. Two investigators screened publications independently through refinement of titles, abstracts, and the full text to select relevant papers based on the inclusion and exclusion criteria. Disagreements between the two reviewers were resolved by discussion and consensus with a third person. Original articles that fulfilled the following criteria were included in the present systematic review and meta-analysis: (1) were conducted in the adult population (18 years and older); and (2) studies with observational design that reported the hazard ratio (HR) or odds ratio (OR) with 95% confidence interval (CI). We also excluded meta-analyses, reviews, randomized clinical trials (RCT), unpublished data, conference letters, and gray literature. The patients, exposure, comparison, and outcome (PECO) description can be observed in S3 Table.

### DPI definition

DPI was computed based on the method developed by McCarty, which was as follows: DPI [9] =  (daily energy derived from phytochemical-rich foods (kcal)/total daily energy intake (kcal)) ×  100. Foods included in the phytochemical-rich classification were whole grains, vegetables, fruits, soy products, legumes, nuts, seeds, olive, olive oil, tomato sauces, natural fruit, and vegetable juices. Among the plant-based foods, potatoes were not included due to their low phytochemical content.

### Data extraction

Information on the characteristics of the studies and participants including author’s name, publication date, research location, number of cases and controls, participants’ age and sex, tools used for dietary assessment, types of cancer, compared categories, reported OR with 95% CI for the association between DPI and cancers risk, adjusted covariates, Newcastle-Ottawa scale (NOS) score were collected by H.A, M.O and N.A.

### Quality assessment

A methodological quality assessment of the qualified studies was evaluated using the NOS designed for the case-control design by two reviewers, independently (S4 Table). The NOS score ranged from 0–9, and we considered studies with 0–3, 3–6, and 7–9 scores as low, moderate, and high quality, respectively [19].

### Statistical analysis

The current meta-analysis includes 8 eligible papers that consist of 9 case-control studies. We extracted the OR with 95% CI for all case-control studies and transformed them into log OR, and then their standard error (SE) was computed. A random-effects model with an inverse variance weighting method was used to estimate the overall effect size. Between-study heterogeneity was assessed using I^2^ quantity [20] (categories as low =  25%, moderate =  50%, and high =  75%) and Cochrane’s Q statistics [21] (P-value <  0.10 considered significant). In our meta-analyses, I^2^ quantity was lower than 50% (I^2^ = 0.0%) and its P-value was not statistically significant (P-value = 0.730). Therefore, we just conducted a subgroup analysis based on types of cancer types. To evaluate the presence of potential publication bias, the visual observational of the funnel plot and Egger’s regression test were used. We used sensitivity analysis, to assess the findings’ robustness. We assessed the risk of bias in individual studies using the Risk of Bias in Non-randomized Studies of Interventions (ROBINS-I) [22]. All statistical analyses were performed using the Stata version 11.2 software, and P <  0.05 was considered statistically significant. All statistical tests were two-sided.

## Results

### Search results

**Fig 1:**
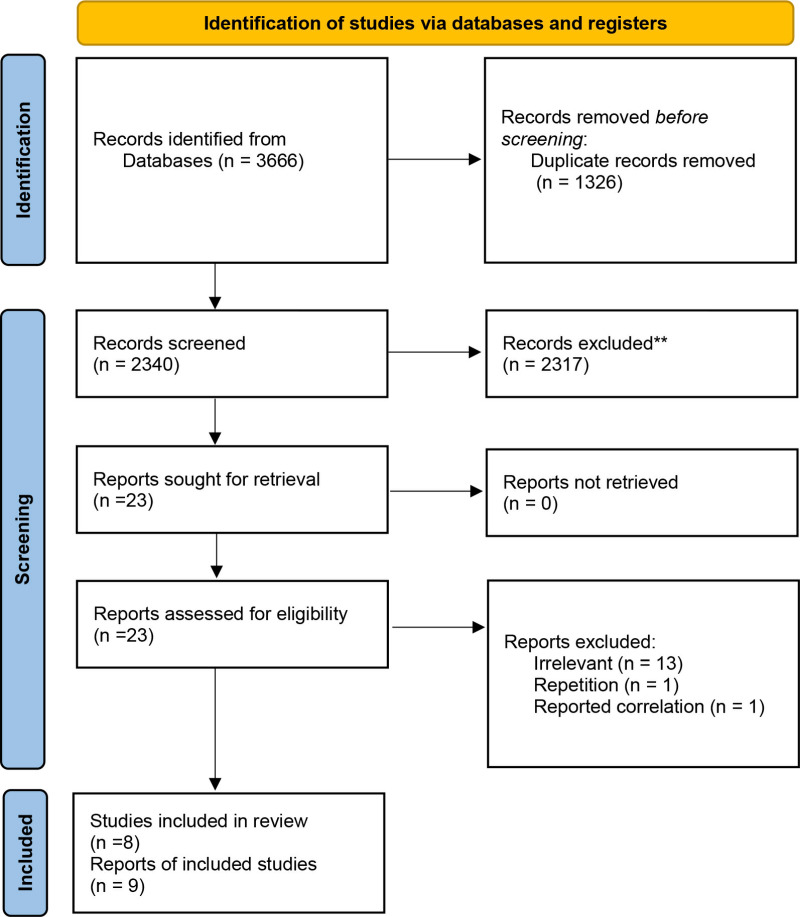
Flow diagram of selection of the published studies.

The study selection process is illustrated in Fig 1. A total of 3666 papers were initially detected. After excluding duplicate (n = 1326) and irrelevant articles (n = 2317), 23 full-text papers of potentially relevant publications were identified. After full-text review (S5 Table), papers were excluded due to the following reasons: irrelevant (n = 13), reported correlation (n = 1), and repetition (n = 1). Finally, 8 papers, including 9 case-control studies were included in the present meta-analysis.

### Study characteristics

Table 1 reported the characteristics of eight case-control studies (including nine reports) [10–17]. These studies were published between 2013 and 2024 and were conducted in Iran (n =  8) and Turkey (n =  1). The sample size in the studies examining the association between DPI and cancer risk ranged from 120 to 851 with an age range of 20 and 75 years. Most studies included females (n =  5), and males (n = 2), and two studies were conducted on both genders. These studies investigated the association between DPI and the risk of BC (n = 5), glioma (n = 1), colorectal cancer (n = 1) and prostate (n = 2). Most studies controlled for some conventional risk factors, including smoking (n = 7), age (n = 6), family history of cancer (n = 6), menopausal status (n = 5), physical activity (n = 6), and education level (n = 6). All studies had high quality based on the NOS criteria (S4 Table). S6 Table illustrates the results of the risk of bias assessment for the individual studies. In summary, we found a moderate risk of bias in all included studies.

**Table 1:** Characteristics of eligible and confirmed studies in the current meta-analysis.^*^

Studies	Country	Cases/Controls	Gender, Age range	Exposure assessment	Cancer type	Comparison	OR (95% CI)	Adjustment for covariate	NOS scores
Aghababayan, 2019	Iran	115/116	Female, 20–65 years	168-Items FFQ	Breast cancer	Q4 vs Q1	0.30 (0.12–0.93)	Age, estrogen therapy, family history of breast disease, intake of dietary supplements, menopause status, waist circumference, and physical activity	9/9
Bahadoran, 2013	Iran	100/175	Female, 30–65 years	168-Items FFQ	Breast cancer	Q4 vs Q1	0.08 (0.01–0.84)	Age, age at menarche (y), age at first pregnancy (y), number of full pregnancy, smoking (yes/no), use of oral contraceptive (yes/no), and the use of bra (<12 h/ > 12h), body mass index (kg/m2) and life satisfaction (yes/no/ partly), menopause status (yes/no), family history of breast cancer (yes/no), physical activity (MET-hours/week), energy intake (kcal/d), and energy density of the diet (kcal/100 g foods)	9/9
Bentyaghoob, 2023	Iran	71/142	Male and Female, 40–75 years	168-Items FFQ	Colorectal cancer	T3 vs T1	0.36 (0.15–0.86)	Energy, smoking, physical activity, Common methods of consuming vegetables, history of CRC, fiber intake, and education level	9/9
Ghoreishy, 2021	Iran	All = 199	Female, 30 years <	106-Items FFQ	Breast cancer	Q4 vs Q1	Premenopausal: 0.88 (0.25–3.04)	Age, residence, marital status, SES, education, family history of BC, menopausal status, history of disease, breastfeeding, supplement use, smoking, and alcohol, BMI.	9/9
Ghoreishy, 2021	Iran	All = 851	Female, 30 years <	106-Items FFQ	Breast cancer	Q4 vs Q1	Postmenopausal: 0.38 (0.24–0.60)	Age, residence, marital status, SES, education, family history of Breast cancer, menopausal status, history of disease, breastfeeding, supplement use, smoking, and alcohol, BMI.	9/9
Rigi, 2021	Iran	128/256	Male and Female, 20–75 years	123-Items FFQ	Glioma cancer	T3 vs T1	0.43 (0.19–0.97)	Age, sex, and energy intake, physical activity, family history of cancer, family history of glioma, marital status, education, high-risk residential area, duration of cell phone use, supplement use, history of exposure to the radiographic X-ray, history of head trauma, history of allergy, history of hypertension, smoking status, exposure to chemicals, drug use, personal hair dye use, frequent fried food intake, frequent use of barbecue, canned foods and microwave, red and processed meat, fish, tea and coffee and, sugar-sweetened beverage, egg, total fat, dietary fiber, cholesterol, folate, selenium	9/9
Pinar, 2022	Turkey	70/70	Female, 18–50 years	229-Items FFQ	Breast cancer	Q4 vs Q1	0.28 (0.02–3.40)^**^	Energy intake, first pregnancy (year), breastfeeding status (yes/no), smoking (yes/no).	8/9
Mousavi, 2024	Iran	112/112	Male, 40–75 years	168-Items FFQ	Benign prostatic hyperplasia	T3 vs T1	0.23 (0.10–0.54)	Energy intake, waist circumference, BMI, weight, education level, family history of BPH, smoking status, physical activity, marital status, red meat, fish, cholesterol, egg, coffee, total fat, and refined grains.	9/9
Mahmoodi, 2024	Iran	60/60	Male, 62.7 ± 12.1 years	FFQ	Prostate cancer	Highest vs lowest	0.52 (0.22–1.21)	Age, BMI, physical activity and education	9/9

* All included studies were case-control. Name of data extractors: Hamid Ahmadirad, Morteza Omrani, and Nikoo Azmi. Date of data extraction: 2024/Dec/29. Sources of data: PubMed, Scopus, and Web of Science.

** We changed the reference group and recalculated the OR (95% CI).

Abbreviations: BMI, body mass index; CI, confidence interval; CRC, Colorectal cancer; FFQ, food frequency questionnaire; OR, odds ratio; Q, quartile; SES, Socioeconomic status; T, Tertile.

### Meta-analysis on DPI and cancer risk

**Fig 2:**
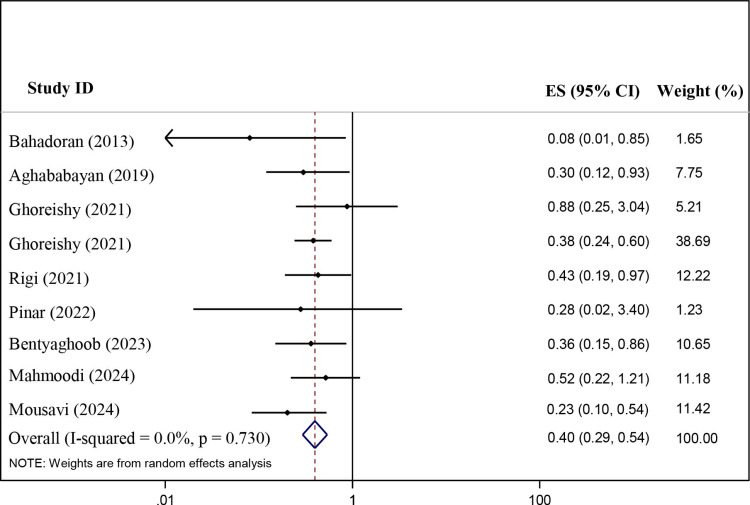
The association of dietary phytochemical index with the risk of different cancer in case-control studies.

Fig 2 reports the results of each study and the overall summary estimate of RR (95% CI) for the association between DPI and cancer risk. The range of RR across the studies was 0.08–0.88 and six studies show a protective effect of DPI on cancer risk, however, three studies didn’t observe a significant relationship in DPI-cancer association. Pooled RR (95% CI) using the random-effects model was 0.40(0.29–0.54) (P-value < 0.001) and the heterogeneity value among studies was not significant (I^2^ =  0.00%; P-heterogeneity = 0.730).

**Fig 3:**
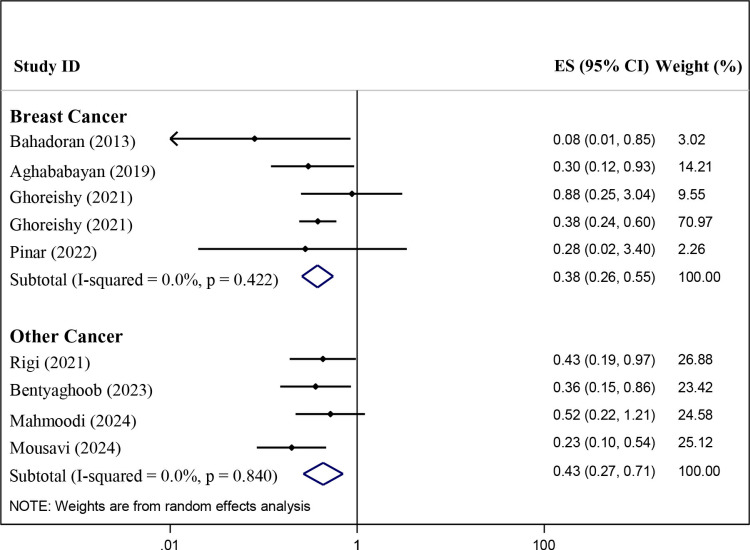
Subgroup analysis based on cancer types for the association of dietary phytochemical index and the cancer risk in the case-control studies.

As indicated in Fig 3, we conducted a subgroup analysis based on cancer types (BC = 5; non-breast cancer = 4). Pooled RR (95% CI) was 0.38 (0.26–0.55) (P-value < 0.001) and 0.43 (0.27–0.71) (P-value = 0.001) for BC and non-breast cancer, respectively. There was no significant heterogeneity among studies for BC (*I*^2^ = 0.00%; *P*-heterogeneity = 0.422) and non-breast cancer (*I*^2^ = 0.00%; *P*-heterogeneity = 0.840).

### Publication bias

A visual inspection of the funnel plot and Egger’s test indicated that there was no publication bias in the association between DPI and cancer risk (P-value =  0.561, Egger’s test) (S1 Fig).

### Sensitivity analysis

We conducted a sensitivity analysis using a random-effects model, excluding none of the studies that had a considerable change on the pooled effect size of the association between DPI and risk of cancer (range: 0.33–0.38) (S7 Table).

## Discussion

In the current meta-analysis, we comprehensively reviewed the association of DPI with various types of cancer risk. The results of our study indicated that a diet with higher scores of DPI could significantly reduce the risk of various types of cancer. Also, the result of subgroup analysis based on types of cancer (BC and other types of cancer) showed that a high DPI score diet was associated with reducing the risk of BC. No evidence of heterogeneity was observed among the included studies for DPI which strengthened the statistical power to approve our findings.

To the best of our knowledge, there is no systematic review and meta-analysis on the possible association between DPI and the risk of cancers, however, the extracted pooled results of the current study are in line with findings of recent systematic reviews that have investigated the relationship between antioxidant compounds with the risk of cancers, and interesting and significant results have been obtained [23–25]. A systematic review study showed an inverse relationship between carotenoid intake from natural sources and the risk of colorectal cancer, which is similar to the study conducted by Bentyaghoob et al. that was included in the current study [14,25]. Also, another meta-analysis revealed that a diet with higher contents of genistein, daidzein, and isoflavones present in soy may reduce the risk of BC in premenopausal and postmenopausal women; these results are similar to the studies of Aghababayan et al., Ghoreishi et al., Pinar et al., and Bahadoran et al., which are included in the current study [10–13,24]. As mentioned, high DPI diets have more calories with phytochemicals such as high amounts of soy [9], or the effectiveness of certain phytochemicals such as resveratrol on glioma, which is similar to the results of Rigi’s study included in this study [17,26], all prove the fact that the phytochemical composition in a diet such as diets with a high DPI score can reduce the risk of cancer.

As we said earlier, after subgroup analysis based on types of cancer, the results still remained significant. Types of cancer utilized for our meta-analysis were BC (Aghababayan et al., Ghoreishi et al., Pinar et al., and Bahadoran et al., studies) and other types of cancer (Rigi et al. and Bentyaghoob et al. studies) which can convey this message for us that higher DPI diet can be effective in the initiation phase of all type of cancer and can hinder formation of tumor cells at the first place. In line with our result, a review study explained that phytochemicals can contribute to the initiation phase of cancer and can prevent cancer incidence [27].

The mechanism and biological pathways explaining the noticeable role of a higher score of DPI in reducing the risk of a wide span of cancers can be as follows; high DPI score diets mostly contain high amounts of fruits, vegetables (excluding potatoes), legumes, whole grains, nuts, seeds, fruit/vegetable juices, soy products, wine, beer, cider and foods compounded that consist of a lot of fiber, antioxidant and valuable vitamins [9]. it is previously suggested that antioxidant compounds of fruits such as berries, vegetables, and also other types of foods rich in phytochemicals can suppress and scavenge free radicals like superoxide radicals, hydrogen peroxide, hydroxyl radicals, and singlet oxygen in the human body [28,29]. As proved earlier free radicals can damage human body cells and even make them mutate and produce a cancerous cell that can progress [30]. Also, high phytochemicals content of diets with higher scores of PI diets can reduce oxidative stresses and damage resulting from sedentary lifestyles, high consumption of long-time fried foods, exposure against toxic agents like insect killers, long-time exposure against UV from sunlight, and mobile phone use [31,32]. Also, refined sugars, refined grains, potato products, hard liquors, and animal products – unfortunately, the main foundations of energy intake in most parts of the world diets for instance, the western diet as an oxidative stress source – were excluded in DPI diets that can also reduce oxidative stresses [9].

As strengths of this study can be mentioned; the current meta-analysis was the first study that investigated the overall outcome of all observational studies conducted on the association of DPI and cancer risk. Another strength of the present study is that we did not observe heterogeneity in the results of the analyzed studies. Furthermore, there was no significant publication bias among the analyzed studies which can make a better image for the final results of this study. Despite this, the limitations of the present study deserve to be mentioned. Firstly, the small number of eligible studies for the present meta-analysis, all of which were case-control in nature, has limited our ability to explore the exact causal relationship between DPI and the risk of various types of cancers. We also know that the type ranges of cancer vary, and this fact can affect the definitive conclusion. Also, eight of nine studies included in this systematic review study were conducted in Iran, because of a lack of data on some items that existed in the DPI diet (e.g., wine, beer, and cider) due to regional and religious sensitivity, they could not attain exact score for their study that can damage overall outcome this meta-analysis. Finally, five out of the nine studies were conducted on women as well as eight out of nine included studies were conducted in Iran which might challenges in generalizing findings across genders and countries.

## Conclusion

In conclusion, the results of the meta-analysis showed that adherence to high DPI diets has a promising improvement in reducing of risk of different types of cancer, especially BC. In general, although the pooled results from our study supported the claim that a diet with higher scores of DPI could be a good dietary pattern for preventing cancer, due to the limited number of studies, their case-control nature included in the current meta-analysis, more observational studies especially with prospective design and larger sample sizes are needed to perform on various society with different food habits to explore a causal relationship and approve the predictive value of DPI in cancer.

## Supporting information

S1 Table Search strategies used for different databases. (DOCX)

S2 Table PRISMA checklist of the current study. (DOCX)

S3 Table The PECO criteria used for the present systematic review and meta-analysis. (DOCX)

S4 Table Quality assessment using New Castle - Ottawa Scale for case-control studies. (DOCX)

S5 Table The list of excluded studies based on full text assessment. (DOCX)

S6 Table The risk of bias assessment of case-control studies using ROBINS-I tool. (DOCX)

S7 Table Sensitivity analyses after removing any study in the association between dietary phytochemical index with the risk of cancers. (DOCX)

S1 Fig Egger’s funnel plot (with pseudo 95% confidence interval) depicting log RR (relative risk) against their corresponding standard error for assessing the presence of publication bias in studies that investigated the association between dietary phytochemical index (DPI) with the cancer risk. (DOCX)

## Abbreviations

BC: Breast cancer
BMI: Body mass index
CI: Confidence interval
DPI: Dietary phytochemical index
HR: Hazard ratio
MeSH: Medical subject heading
NOS: Newcastle-Ottawa scale
OR: Odds ratio
PRISMA: Preferred Reporting Items for Systematic Reviews and Meta-analysis
SE: Standard error.
