# Supplementary material for: Dietary phytochemical index and the risk of cancer: A systematic review and meta-analysis
Source: PLoS One. 2025 Apr 2;20(4):e0319591. doi: 10.1371/journal.pone.0319591 (PMC11964270; doi:10.1371/journal.pone.0319591)
Supplement: S1 Table — (DOCX) [file pone.0319591.s001.docx]

**Table S1.** Search Strategies used for different databases.

**Search date:** 2024/Dec/29

| **databases** | **Search Strategy** | **Amount of articles** |
| --- | --- | --- |
| **PubMed** | ("cancer*"[Title/Abstract] OR "tumor*"[Title/Abstract] OR "malignanc*"[Title/Abstract] OR "carcinogen*"[Title/Abstract] OR "carcinoma*"[Title/Abstract]) AND ("dietary phytochemical*"[Title/Abstract] OR "dietary phytochemical index*"[Title/Abstract] OR "DPI"[Title/Abstract]) | 912 |
| **Scopus** | ( TITLE-ABS-KEY ( ( cancer* ) OR ( neoplasm* ) OR ( malignancy* ) OR ( tumor* ) OR ( death* ) OR ( carcinogen* ) ) AND TITLE-ABS-KEY ( ( "dietary phytochemical" ) OR ( "dietary phytochemical index" ) OR ( dpi* ) ) ) | 2299 |
| **Web of science** | cancer* OR neoplasm* OR malignancy* OR tumor* OR carcinogen* OR carcinoma* (Topic) and "dietary phytochemical" OR "dietary phytochemical index" OR "DPI" (Topic) | 642 |
