# Supplementary material for: Dietary phytochemical index and the risk of cancer: A systematic review and meta-analysis
Source: PLoS One. 2025 Apr 2;20(4):e0319591. doi: 10.1371/journal.pone.0319591 (PMC11964270; doi:10.1371/journal.pone.0319591)
Supplement: S3 Table — (DOCX) [file pone.0319591.s003.docx]

**Table S3. The PECO criteria used for the present systematic review and meta-analysis.**

| **PECO criteria** | **Description** |
| --- | --- |
| **Patients** | All age range |
| **Exposure** | Dietary phytochemical index (DPI) |
| **Comparison** | The highest category of DPI versus the lowest category |
| **Outcome** | The risk of cancers |
