# Supplementary material for: Dietary phytochemical index and the risk of cancer: A systematic review and meta-analysis
Source: PLoS One. 2025 Apr 2;20(4):e0319591. doi: 10.1371/journal.pone.0319591 (PMC11964270; doi:10.1371/journal.pone.0319591)
Supplement: S4 Table — (DOCX) [file pone.0319591.s004.docx]

**Table S4.** Quality assessment using New Castle - Ottawa Scale for case-control studies^*^

|  | **Selection** | | |  | **Comparability** | **Exposure** | | | **Study score** | **Quality of study** |
| --- | --- | --- | --- | --- | --- | --- | --- | --- | --- | --- |
| **First author (Year)** | Is the case definition adequate? | Representativeness of the cases | Selection of Controls | Definition of Controls | Comparability of cases and controls on the basis of the design or analysis | Ascertainment of exposure | Same method of ascertainment for cases and controls | Non-Response rate |  |  |
| Aghababayan,2019 | * | * | * | * | ** | * | * | * | 9/9 | high-quality |
| Bahadoran, 2013 | * | * | * | * | ** | * | * | * | 9/9 | high-quality |
| Bentyaghoob, 2023 | * | * | * | * | ** | * | * | * | 9/9 | high-quality |
| Ghoreishy, 2021 | * | * | * | * | ** | * | * | * | 9/9 | high-quality |
| Rigi, 2021 | * | * | * | * | ** | * | * | * | 9/9 | high-quality |
| Pinar, 2022 | - | * | * | * | ** | * | * | * | 8/9 | high-quality |
| Mousavi, 2024 | * | * | * | * | ** | * | * | * | 9/9 | high-quality |
| Mahmoodi, 2024 | * | * | * | * | ** | * | * | * | 9/9 | high-quality |

*^*^ Study score less than 4 indicates low quality, a score of 4 to 6 represents moderate, and a score of more than 6 indicates as a high-quality*
