# Supplementary material for: Dietary phytochemical index and the risk of cancer: A systematic review and meta-analysis
Source: PLoS One. 2025 Apr 2;20(4):e0319591. doi: 10.1371/journal.pone.0319591 (PMC11964270; doi:10.1371/journal.pone.0319591)
Supplement: S5 Table — (DOCX) [file pone.0319591.s005.docx]

**Table S5.** The list of excluded studies based on full text assessment.

|  | The references |
| --- | --- |
| Duplicate (n=1326) | (1-1302)(1303-1326) |
| Irrelevant (n=2317) | (34, 38, 51, 52, 59, 66, 71, 74, 82, 85, 93, 96, 102, 104, 112, 114, 122, 125, 126, 149, 151, 154, 155, 157, 166, 181, 183, 185, 191, 194, 201, 207, 225, 233, 235, 236, 239, 247, 257, 278, 280, 282, 283, 286, 288, 292, 306, 309, 314, 319, 337, 342, 344, 357, 361, 372, 376, 384, 394, 396, 400, 403, 414, 428, 433, 438, 449, 461, 465, 467, 475, 479, 484, 489, 502, 508, 512, 536, 537, 543, 546, 552, 557, 581, 586, 593, 605, 614, 615, 618, 655, 661, 664, 666, 674, 679, 684, 685, 698, 705, 706, 708, 712, 715, 731, 733, 750, 755, 769, 776, 777, 781, 788, 795, 813, 817, 842, 856, 858, 866, 870, 872, 875, 877, 879, 881, 883, 891, 903, 910, 914, 945, 946, 957, 963, 986, 998, 1003, 1005, 1019, 1062, 1072, 1081, 1111, 1120, 1121, 1134, 1140, 1187, 1205, 1237, 1240, 1254, 1262, 1291, 1327-3484) |
| Irrelevant outcome (n=13) | (3485-3497) |
| Repetition (n=1) | (3498) |
| Reported correlation (n=1) | (3499) |

1. Penn EJ, Hobson C, Rees DA, Magee AI. Structure and assembly of desmosome junctions: biosynthesis, processing, and transport of the major protein and glycoprotein components in cultured epithelial cells. The Journal of cell biology. 1987;105(1):57-68.

2. Bernstein JJ, Goldberg WJ, Laws ER. Human malignant astrocytoma xenografts migrate in rat brain: A model for central nervous system cancer research. J Neurosci Res. 1989;22(2):134-43.

3. Bernstein JJ, Goldberg WJ, Laws Jr ER, Conger D, Morreale V, Wood LR. C6 glioma cell invasion and migration of rat brain after neural homografting: Ultrastructure. Neurosurgery. 1990;26(4):622-8.

4. Tochikura TS, Hayes KA, Cheney CM, Tanabe-Tochikura A, Rojko JL, Mathes LE, et al. In vitro replication and cytopathogenicity of the feline immunodeficiency virus for feline t4 thymic lymphoma 3201 cells. Virology. 1990;179(1):492-7.

5. Hancock JT, White JI, Jones OTG, Silver IA. The use of diphenylene iodonium and its analogues to investigate the role of the nadph oxidase in the tumoricidal activity of macrophages in vitro. Free Radic Biol Med. 1991;11(1):25-9.

6. Hancock JT, White JI, Jones OTG, Silver IA. THE USE OF DIPHENYLENE IODONIUM AND ITS ANALOGS TO INVESTIGATE THE ROLE OF THE NADPH OXIDASE IN THE TUMORICIDAL ACTIVITY OF MACROPHAGES INVITRO. Free Radic Biol Med. 1991;11(1):25-9.

7. Leen E, Goldberg JA, Robertson J, Sutherland G, Hemingway DM, Cooke TG, et al. Detection of hepatic metastases using duplex/color Doppler sonography. Ann Surg. 1991;214(5):599-604.

8. Leen E, Goldberg JA, Robertson J, Sutherland GR, McArdle CS. The use of duplex sonography in the detection of colorectal hepatic metastases. Br J Cancer. 1991;63(2):323-5.

9. Grunt TW, Somay C, Oeller H, Dittrich E, Dittrich C. Comparative analysis of the effects of dimethyl sulfoxide and retinoic acid on the antigenic pattern of human ovarian adenocarcinoma cells. J Cell Sci. 1992;103 ( Pt 2):501-9.

10. Grunt TW, Somay C, Oeller H, Dittrich E, Dittrich C. COMPARATIVE-ANALYSIS OF THE EFFECTS OF DIMETHYL-SULFOXIDE AND RETINOIC ACID ON THE ANTIGENIC PATTERN OF HUMAN OVARIAN ADENOCARCINOMA CELLS. J Cell Sci. 1992;103:501-9.

11. Keller R, Bassetti S, Keist R, Mülsch A, Klauser S. Induction of nitric oxide synthase is a necessary precondition for expression of tumor necrosis factor-independent tumoricidal activity by activated macrophages. Biochem Biophys Res Commun. 1992;184(3):1364-71.

12. Mokrý J, Nemecek S, Adler J. Inoculation of C6 glioma cell suspension into the brain of adult rats: morphological study. Sborník vedeckých prací Lékarské fakulty Karlovy university v Hradci Králové. 1992;35(4):293-305.

13. Robertson J, Leen E, Goldberg JA, Angerson WJ, Sutherland GR, McArdle CS. Flow measurement using duplex Doppler ultrasound: Haemodynamic changes in patients with colorectal liver metastases. Clinical Physics and Physiological Measurement. 1992;13(4):299-310.

14. Robertson J, Leen E, Goldberg JA, Angerson WJ, Sutherland GR, McArdle CS. FLOW MEASUREMENT USING DUPLEX DOPPLER ULTRASOUND - HEMODYNAMIC-CHANGES IN PATIENTS WITH COLORECTAL LIVER METASTASES. Clinical Physics and Physiological Measurement. 1992;13(4):299-310.

15. Leen E, Goldberg JA, Robertson J, Angerson WJ, Sutherland GR, Cooke TG, et al. Early detection of occult colorectal hepatic metastases using duplex colour Doppler sonography. Br J Surg. 1993;80(10):1249-51.

16. Leen E, Goldberg JA, Robertson J, Angerson WJ, Sutherland GR, Cooke TG, et al. IMAGE-DIRECTED DOPPLER ULTRASONOGRAPHY - A NOVEL TECHNIQUE FOR THE DIAGNOSIS OF COLORECTAL LIVER METASTASES. J Clin Ultrasound. 1993;21(4):221-30.

17. Norian LA, Rosenbusch RF. Mycoplasma bovoculi-Augmented bovine natural killer activity. Comparative Immunology, Microbiology and Infectious Diseases. 1993;16(2):113-22.

18. Filippone A, Basilico R, Guidotti A, Boni R, Belfiore G, Bonomo L. [Color Doppler ultrasonography in the identification and characterization of secondary focal lesions of the liver]. Radiol Med. 1994;87(3):283-8.

19. Hall DG, Stoica G. Characterization of brain and bone-metastasizing clones selected from an ethylnitrosourea-induced rat mammary carcinoma. Clin Exp Metastasis. 1994;12(4):283-95.

20. Hall DG, Stoica G. CHARACTERIZATION OF BRAIN AND BONE-METASTASIZING CLONES SELECTED FROM AN ETHYLNITROSOUREA-INDUCED RAT MAMMARY-CARCINOMA. Clin Exp Metastasis. 1994;12(4):283-95.

21. Leen E, Angerson WJ, Wotherspoon H, Moule B, Cooke TG, McArdle CS. Comparison of the Doppler perfusion index and intraoperative ultrasonography in diagnosing colorectal liver metastases. Evaluation with postoperative follow-up results. Ann Surg. 1994;220(5):663-7.

22. Leen E, Angerson WJ, Wotherspoon H, Moule B, Cooke TG, McArdle CS. COMPARISON OF THE DOPPLER PERFUSION INDEX AND INTRAOPERATIVE ULTRASONOGRAPHY IN DIAGNOSING COLORECTAL LIVER METASTASES - EVALUATION WITH POSTOPERATIVE FOLLOW-UP RESULTS. Ann Surg. 1994;220(5):663-7.

23. Leen E, Angerson WJ, Wotherspoon H, Moule B, Cook TG, McArdle CS. Detection of colorectal liver metastases: Comparison of laparotomy, CT, US, and Doppler perfusion index and evaluation of postoperative follow-up results. Radiology. 1995;195(1):113-6.

24. Surh YJ, Lee RC, Park KK, Mayne ST, Liem A, Miller JA. Chemoprotective effects of capsaicin and diallyl sulfide against mutagenesis or tumorigenesis by vinyl carbamate and N-nitrosodimethylamine. Carcinogenesis. 1995;16(10):2467-71.

25. Surh YJ, Lee RCJ, Park KK, Mayne ST, Liem A, Miller JA. CHEMOPROTECTIVE EFFECTS OF CAPSAICIN AND DIALLYL SULFIDE AGAINST MUTAGENESIS OR TUMORIGENESIS BY VINYL CARBAMATE AND N-NITROSODIMETHYLAMINE. Carcinogenesis. 1995;16(10):2467-71.

26. Leen E, Angerson WG, Cooke TG, McArdle CS. Prognostic power of Doppler perfusion index in colorectal cancer: Correlation with survival. Ann Surg. 1996;223(2):199-203.

27. Leen E, Angerson WG, Cooke TG, McArdle CS. Prognostic power of Doppler perfusion index in colorectal cancer. Correlation with survival. Ann Surg. 1996;223(2):199-203.

28. Miesel R, Kurpisz M, Kröger H. Suppression of inflammatory arthritis by simultaneous inhibition of nitric oxide synthase and NADPH oxidase. Free Radic Biol Med. 1996;20(1):75-81.

29. Ng KH, Looi LM, Bradley DA. Microcalcification clustering parameters in breast disease: A morphometric analysis of radiographs of excision specimens. British Journal of Radiology. 1996;69(820):326-34.

30. Ruhfberg C, Williamson JA, Sheer D, Watt FM. Chromosomal localisation of the human envoplakin gene (EVPL) to the region of the tylosis oesophageal cancer gene (TOCG) on 17q25. Genomics. 1996;37(3):381-5.

31. Ruhrberg C, Williamson JA, Sheer D, Watt FM. Chromosomal localisation of the human envoplakin gene (EVPL) to the region of the tylosis oesophageal cancer gene (TOCG) on 17q25. Genomics. 1996;37(3):381-5.

32. Vuong PN, Bayssade-Dufour C, Albaret JL, Farhati K. Histopathological observations in new and classic models of experimental Schistosoma haematobium infections. Trop Med Int Health. 1996;1(3):348-58.

33. Vuong PN, BayssadeDufour C, Albaret JL, Farhati K. Histopathological observations in new and classic models of experimental Schistosoma haematobium infections. Trop Med Int Health. 1996;1(3):348-58.

34. Chan MMY, Fong D, Ho CT, Huang HI. Inhibition of inducible nitric oxide synthase gene expression and enzyme activity by epigallocatechin gallate, a natural product from green tea. Biochem Pharmacol. 1997;54(12):1281-6.

35. Cooper DA, Webb DR, Peters JC, Middleton SJ. Evaluation of the potential for olestra to affect the availability of dietary phytochemicals. J Nutr. 1997;127(8 SUPPL.):1699S-709S.

36. Leen E, Anderson JR, Robertson J, O'Gorman P, Cooke TG, McArdle CS. Doppler index perfusion in the detection of hepatic metastases secondary to gastric carcinoma. Am J Surg. 1997;173(2):99-102.

37. Leen E, Anderson JR, Robertson J, O'Gorman P, Cooke TG, McArdle CS. Doppler index perfusion in the detection of hepatic metastases secondary to gastric carcinoma. Am J Surg. 1997;173(2):99-102.

38. Shi M, Feng YM. Studies on growth-promoting action of insulin: Mitogenic activity of insulin and its analogues in mouse mammary tumor cells. Biochem Mol Biol Int. 1997;43(4):705-11.

39. Shi M, Feng YM. Studies on growth-promoting action of insulin: mitogenic activity of insulin and its analogues in mouse mammary tumor cells. Biochem Mol Biol Int. 1997;43(4):705-11.

40. Sokoloski JA, Hodnick WF, Mayne ST, Cinquina C, Kim CS, Sartorelli AC. Induction of the differentiation of HL-60 promyelocytic leukemia cells by vitamin E and other antioxidants in combination with low levels of vitamin D3: Possible relationship to NF-κB. Leukemia. 1997;11(9):1546-53.

41. Zippelius A, Kufer P, Honold G, Köllermann MW, Oberneder R, Schlimok G, et al. Limitations of reverse-transcriptase polymerase chain reaction analyses for detection of micrometastatic epithelial cancer cells in bone marrow. J Clin Oncol. 1997;15(7):2701-8.

42. Zippelius A, Kufer P, Honold G, Kollermann MW, Oberneder R, Schlimok G, et al. Limitations of reverse-transcriptase polymerase chain reaction analyses for detection of micrometastatic epithelial cancer cells in bone marrow. J Clin Oncol. 1997;15(7):2701-8.

43. Buchbinder SS, Leichter IS, Bamberger PN, Novak B, Lederman R, Fields S, et al. Analysis of clustered microcalcifications by using a single numeric classifier extracted from mammographic digital images. Acad Radiol. 1998;5(11):779-84.

44. Buchbinder SS, Leichter IS, Bamberger PN, Novak B, Lederman R, Fields S, et al. Analysis of clustered microcalcifications by using a single numeric classifier extracted from mammographic digital images. Acad Radiol. 1998;5(11):779-84.

45. Hihara H, Maeda M, Nakamura K, Ishino S, Tsukamoto K, Yuasa N, et al. Rapid Induction of Lymphoid Leukosis and Ascites by Avian Leukosis Virus from a Lymphoid Leukosis Cell Line. J Vet Med Sci. 1998;60(1):77-85.

46. Jankevicius F, Shibayama T, Decken K, Bojar H, Gerharz CD, Ebert T, et al. Dual-parameter immunoflow cytometry in diagnosis and follow-up of patients with bladder cancer. Eur Urol. 1998;34(6):492-9.

47. Jankevicius F, Shibayama T, Decken K, Bojar H, Gerharz CD, Ebert T, et al. Dual-parameter immunoflow cytometry in diagnosis and follow-up of patients with bladder cancer. Eur Urol. 1998;34(6):492-9.

48. Macé K, Offord EA, Harris CC, Pfeifer AM. Development of in vitro models for cellular and molecular studies in toxicology and chemoprevention. Archives of toxicology Supplement = Archiv für Toxikologie Supplement. 1998;20:227-36.

49. Mersseman B, De Wagter C. Characteristics of a commercially available film digitizer and their significance for film dosimetry. Physics in Medicine and Biology. 1998;43(6):1803-12.

50. Michnovicz JJ. Increased estrogen 2-hydroxylation in obese women using oral indole-3-carbinol. Int J Obes Relat Metab Disord. 1998;22(3):227-9.

51. Michnovicz JJ. Increased estrogen 2-hydroxylation in obese women using oral indole-3-carbinol. Int J Obes. 1998;22(3):227-9.

52. Oppo K, Leen E, Angerson WJ, Cooke TG, McArdle CS. Doppler perfusion index: An interobserver and intraobserver reproducibility study. Radiology. 1998;208(2):453-7.

53. Oppo K, Leen E, Angerson WJ, Cooke TG, McArdle CS. Doppler perfusion index: an interobserver and intraobserver reproducibility study. Radiology. 1998;208(2):453-7.

54. Surh YJ, Lee E, Lee JM, editors. Chemoprotective properties of some pungent ingredients present in red pepper and ginger. Mutation Research - Fundamental and Molecular Mechanisms of Mutagenesis; 1998.

55. Surh YJ, Lee E, Lee JM. Chemoprotective properties of some pungent ingredients present in red pepper and ginger. Mutat Res-Fundam Mol Mech Mutagen. 1998;402(1-2):259-67.

56. Waladkhani AR, Clemens MR. Effect of dietary phytochemicals on cancer development (review). Int J Mol Med. 1998;1(4):747-53.

57. Walsh KM, Leen E, MacSween RN, Morris AJ. Hepatic blood flow changes in chronic hepatitis C measured by duplex Doppler color sonography: relationship to histological features. Dig Dis Sci. 1998;43(12):2584-90.

58. Zhai S, Dai R, Friedman FK, Vestal RE. Comparative inhibition of human cytochromes P450 1A1 and 1A2 by flavonoids. Drug Metabolism and Disposition. 1998;26(10):989-92.

59. Blouin E, Halbwachs-Mecarelli L, Rieu P. Redox regulation of β2-integrin CD11b/CD18 activation. Eur J Immunol. 1999;29(11):3419-31.

60. Leen E. The detection of occult liver metastases of colorectal carcinoma. Journal of Hepato-Biliary-Pancreatic Surgery. 1999;6(1):7-15.

61. Sauer H, Dagdanova A, Hescheler J, Wartenberg M. Redox-regulation of intrinsic prion expression in multicellular prostate tumor spheroids. Free Radic Biol Med. 1999;27(11-12):1276-83.

62. Shinomiya T, Mori T, Ariyama Y, Sakabe T, Fukuda Y, Murakami Y, et al. Comparative genomic hybridization of squamous cell carcinoma of the esophagus: the possible involvement of the DPI gene in the 13q34 amplicon. Genes Chromosomes Cancer. 1999;24(4):337-44.

63. Shinomiya T, Mori T, Ariyama Y, Sakabe T, Fukuda Y, Murakami Y, et al. Comparative genomic hybridization of squamous cell carcinoma of the esophagus:: The possible involvement of the <i>DPI</i> gene in the 13q34 amplicon. Gene Chromosomes Cancer. 1999;24(4):337-44.

64. Zhukov A, Ingelman-Sundberg M. Relationship between cytochrome P450 catalytic cycling and stability: fast degradation of ethanol-inducible cytochrome P450 2E1 (CYP2E1) in hepatoma cells is abolished by inactivation of its electron donor NADPH-cytochrome P450 reductase. Biochem J. 1999;340 ( Pt 2)(Pt 2):453-8.

65. Arai Y, Nakamura Y, Inoue F, Yamamoto K, Saito K, Furusawa S. Glucocorticoid-induced apoptotic pathways in eosinophils: comparison with glucocorticoid-sensitive leukemia cells. Int J Hematol. 2000;71(4):340-9.

66. Ashida H, Fukuda I, Yamashita T, Kanazawa K. Flavones and flavonols at dietary levels inhibit a transformation of aryl hydrocarbon receptor induced by dioxin. FEBS Lett. 2000;476(3):213-7.

67. Dorsam G, Taher MM, Valerie KC, Kuemmerle NB, Chan JC, Franson RC. Diphenyleneiodium chloride blocks inflammatory cytokine-induced up- regulation of group IIA phospholipase A2 in rat mesangial cells. J Pharmacol Exp Ther. 2000;292(1):271-9.

68. Dorsam G, Taher MM, Valerie KC, Kuemmerle NB, Chan JC, Franson RC. Diphenyleneiodium chloride blocks inflammatory cytokine-induced up-regulation of group IIA phospholipase A<sub>2</sub> in rat mesangial cells. J Pharmacol Exp Ther. 2000;292(1):271-9.

69. Krüger S, Strobel D, Wehler M, Wein A, Hahn EG, Becker D. [Hepatic Doppler perfusion index--a sensitive screening method for detecting liver metastases?]. Ultraschall Med. 2000;21(5):206-9.

70. Krüger S, Strobel D, Wehler M, Wein A, Hahn EG, Becker D. Hepatic Doppler perfusion index -: a sensitive tool for detection of liver metastases? Ultraschall Med. 2000;21(5):206-9.

71. Leen E, Goldberg JA, Angerson WJ, McArdle CS. Potential role of doppler perfusion index in selection of patients with colorectal cancer for adjuvant chemotherapy. Lancet. 2000;355(9197):34-7.

72. Leen E, Goldberg JA, Angerson WJ, McArdle CS. Potential role of doppler perfusion index in selection of patients with colorectal cancer for adjuvant chemotherapy. Lancet. 2000;355(9197):34-7.

73. Oppo K, Leen E, Angerson WJ, McArdle CS. The effect of resecting the primary tumour on the Doppler Perfusion Index in patients with colorectal cancer. Clin Radiol. 2000;55(10):791-3.

74. Oppo K, Leen E, Angerson WJ, McArdle CS. The effect of resecting the primary tumour on the Doppler perfusion index in patients with colorectal cancer. Clin Radiol. 2000;55(10):791-3.

75. Peeters CF, Thomas CM, Sweep FC, Span PN, Wobbes T, Ruers TM. Elevated serum endothelin-1 levels in patients with colorectal cancer: Relevance for prognosis. Int J Biol Markers. 2000;15(4):288-93.

76. Peeters CF, Thomas CM, Sweep FC, Span PN, Wobbes T, Ruers TM. Elevated serum endothelin-1 levels in patients with colorectal cancer; relevance for prognosis. Int J Biol Markers. 2000;15(4):288-93.

77. Tolando R, Jovanović A, Brigelius-Flohé R, Ursini F, Maiorino M. Reactive oxygen species and proinflammatory cytokine signaling in endothelial cells: Effect of selenium supplementation. Free Radic Biol Med. 2000;28(6):979-86.

78. Tolando R, Jovanovic A, Brigelius-Flohé R, Ursini F, Maiorino M. Reactive oxygen species and proinflammatory cytokine signaling in endothelial cells:: Effect of selenium supplementation. Free Radic Biol Med. 2000;28(6):979-86.

79. Tummala PE, Chen XL, Medford RM. NF-κB independent suppression of endothelial vascular cell adhesion molecule-1 and intercellular adhesion molecule-1 gene expression by inhibition of flavin binding proteins and superoxide production. J Mol Cell Cardiol. 2000;32(8):1499-508.

80. Waladkhani AR, Clemens MR. Effect of dietary phytochemicals on cancer development. Vegetables, Fruits, and Herbs in Health Promotion2000. p. 3-18.

81. Yarmenitis SD, Kalogeropoulou CP, Hatjikondi O, Ravazoula P, Petsas T, Siamblis D, et al. An experimental approach of the Doppler perfusion index of the liver in detecting occult hepatic metastases: histological findings related to the hemodynamic measurements in Wistar rats. Eur Radiol. 2000;10(3):417-24.

82. Yarmenitis SD, Kalogeropoulou CP, Hatjikondi O, Ravazoula P, Petsas T, Siamblis D, et al. An experimental approach of the Doppler perfusion index of the liver in detecting occult hepatic metastases: histological findings related to the hemodynamic measurements in Wistar rats. Eur Radiol. 2000;10(3):417-24.

83. Blum CA, Xu M, Orner GA, Fong AT, Bailey GS, Stoner GD, et al. beta-Catenin mutation in rat colon tumors initiated by 1,2-dimethylhydrazine and 2-amino-3-methylimidazo[4,5-f]quinoline, and the effect of post-initiation treatment with chlorophyllin and indole-3-carbinol. Carcinogenesis. 2001;22(2):315-20.

84. Ferreira AM, Martins MS, Vitor RWA. Virulence for BALB/c mice and antigenic diversity of eight Toxoplasma gondii strains isolated from animals and humans in Brazil. Parasite. 2001;8(2):99-105.

85. Kissel A, Rixe O, Methlin A, Nabet M, Tranquart F, Rubini B, et al. Quantification of hepatic arterial and portal venous flow using US contrast agents for early detection of liver metastases from colon carcinoma. J Radiol. 2001;82(11):1621-5.

86. Kissel A, Rixe O, Methlin A, Nabet M, Tranquart F, Rubini B, et al. [Quantification of hepatic arterial and portal venous flow using ultrasound contrast agents for early detection of liver metastases of colorectal cancers]. J Radiol. 2001;82(11):1621-5.

87. Kono H, Rusyn I, Uesugi T, Yamashina S, Connor HD, Dikalova A, et al. Diphenyleneiodonium sulfate, an NADPH oxidase inhibitor, prevents early alcohol-induced liver injury in the rat. Am J Physiol Gastrointest Liver Physiol. 2001;280(5):G1005-12.

88. Kono H, Rusyn I, Uesugi T, Yamashina S, Connor HD, Dikalova A, et al. Diphenyleneiodonium sulfate, an NADPH oxidase inhibitor, prevents early alcohol-induced liver injury in the rat. Am J Physiol-Gastroint Liver Physiol. 2001;280(5):G1005-G12.

89. Laudański P, Dziecioł J, Anchim T, Wołczyński S. The influence of glyco-nitric oxide conjugate on proliferation of breast cancer cells in vitro. Folia Histochem Cytobiol. 2001;39 Suppl 2:87-8.

90. Laudanski P, Dzieciol J, Anchim T, Wolczynski S. The influence of glyco-nitric oxide conjugate on proliferation of breast cancer cells in vitro. Folia Histochem Cytobiol. 2001;39:87-8.

91. Lobell RB, Omer CA, Abrams MT, Bhimnathwala HG, Brucker MJ, Buser CA, et al. Evaluation of farnesyl:protein transferase and geranylgeranyl:protein transferase inhibitor combinations in preclinical models. Cancer Res. 2001;61(24):8758-68.

92. Lobell RB, Omer CA, Abrams MT, Bhimnathwala HG, Brucker MJ, Buser CA, et al. Evaluation of farnesyl:protein transferase and geranylgeranyl:protein transferase inhibitor combinations in preclinical models. Cancer Res. 2001;61(24):8758-68.

93. Nomura S, Tatemichi M, Kaminishi M, Esumi H. A novel method for detecting single glandular intestinal metaplasia in the mucosal surface of the fixed stomach using methylene blue. Jpn J Cancer Res. 2001;92(6):659-65.

94. Nomura S, Tatemichi M, Kaminishi M, Esumi H. A novel method for detecting single glandular intestinal metaplasia in the mucosal surface of the fixed stomach using methylene blue. Jpn J Cancer Res. 2001;92(6):659-65.

95. O'Blenes CA, Kinnear C, Rabinovitch M. Tumor necrosis factor-alpha induces fibronectin synthesis in coronary artery smooth muscle cells by a nitric oxide-dependent posttranscriptional mechanism. Circ Res. 2001;89(1):26-32.

96. O'Blenes CAE, Kinnear C, Rabinovitch M. Tumor necrosis factor-α induces fibronectin synthesis in coronary artery smooth muscle cells by a nitric oxide-dependent posttranscriptional mechanism. CircRes. 2001;89(1):26-32.

97. Sauer H, Klimm B, Hescheler J, Wartenberg M. Activation of p90RSK and growth stimulation of multicellular tumor spheroids are dependent on reactive oxygen species generated after purinergic receptor stimulation by ATP. The FASEB journal : official publication of the Federation of American Societies for Experimental Biology. 2001;15(13):2539-41.

98. Sauer H, Klimm B, Hescheler J, Wartenberg M. Activation of p90RSK and growth stimulation of multicellular tumor spheroids are dependent on reactive oxygen species generated after purinergic receptor stimulation by ATP. Faseb j. 2001;15(13):2539-41.

99. White JE, Tsan MF. Differential induction of TNF-α and MnSOD by endotoxin role of reactive oxygen species and NADPH oxidase. Am J Respir Cell Mol Biol. 2001;24(2):164-9.

100. White JE, Tsan MF. Differential induction of TNF-alpha and MnSOD by endotoxin: role of reactive oxygen species and NADPH oxidase. Am J Respir Cell Mol Biol. 2001;24(2):164-9.

101. Buchbinder SS, Leichter IS, Lederman RB, Novak B, Bamberger PN, Coopersmith H, et al. Can the size of microcalcifications predict malignancy of clusters at mammography? Acad Radiol. 2002;9(1):18-25.

102. Buchbinder SS, Leichter IS, Lederman RB, Novak B, Bamberger PN, Coopersmith H, et al. Can the size of microcalcifications predict malignancy of clusters at mammography? Acad Radiol. 2002;9(1):18-25.

103. Chen YC, Shen SC, Lee WR, Hsu FL, Lin HY, Ko CH, et al. Emodin induces apoptosis in human promyeloleukemic HL-60 cells accompanied by activation of caspase 3 cascade but independent of reactive oxygen species production. Biochem Pharmacol. 2002;64(12):1713-24.

104. Chen YC, Shen SC, Lee WR, Hsu FL, Lin HY, Ko CH, et al. Emodin induces apoptosis in human promyeloleukemic HL-60 cells accompanied by activation of caspase 3 cascade but independent of reactive oxygen species production. Biochem Pharmacol. 2002;64(12):1713-24.

105. Holian O, Wahid S, Atten MJ, Attar BM. Inhibition of gastric cancer cell proliferation by resveratrol: Role of nitric oxide. American Journal of Physiology - Gastrointestinal and Liver Physiology. 2002;282(5 45-5):G809-G16.

106. Holian O, Wahid S, Atten MJ, Attar BM. Inhibition of gastric cancer cell proliferation by resveratrol: role of nitric oxide. Am J Physiol Gastrointest Liver Physiol. 2002;282(5):G809-16.

107. Holy JM. Curcumin disrupts mitotic spindle structure and induces micronucleation in MCF-7 breast cancer cells. Mutation Research - Genetic Toxicology and Environmental Mutagenesis. 2002;518(1):71-84.

108. Holy JM. Curcumin disrupts mitotic spindle structure and induces micronucleation in MCF-7 breast cancer cells. Mutat Res. 2002;518(1):71-84.

109. Ikegami H, Kajikawa S, Ito K, Nii A, Okamiya H, Nakayama H, et al. Immunohistochemical study on inducible type of nitric oxide (iNOS), basic fibroblast growth factor (bFGF) and tumor growth factor-β1 (TGF-β1) in arteritis induced in rats by fenoldopam and theophylline, vasodilators. Exp Toxicol Pathol. 2002;54(1):1-7.

110. Paysant JR, Rupin A, Verbeuren TJ. Effect of NADPH oxidase inhibition on E-selectin expression induced by concomitant anoxia/reoxygenation and TNF-alpha. Endothelium. 2002;9(4):263-71.

111. Paysant JR, Rupin A, Verbeuren TJ. Effect of NADPH oxidase inhibition on E-selectin expression induced by concomitant anoxia/reoxygenation and TNF-α. Endothelium. 2002;9(4):263-71.

112. Pullar JM, Hampton MB. Diphenyleneiodonium triggers the efflux of glutathione from cultured cells. J Biol Chem. 2002;277(22):19402-7.

113. Pullar JM, Hampton MB. Diphenyleneiodonium triggers the efflux of glutathione from cultured cells. J Biol Chem. 2002;277(22):19402-7.

114. Ruutu M, Peitsaro P, Johansson B, Syrjänen S. Transcriptional profiling of a human papillomavirus 33-positive squamous epithelial cell line which acquired a selective growth advantage after viral integration. Int J Cancer. 2002;100(3):318-26.

115. Zhu BT. Catechol-O-methyltransferase (COMT)-mediated methylation metabolism of endogenous bioactive catechols and modulation by endobiotics and kenobiotics: Importance in pathophysiology and pathogenesis. Curr Drug Metab. 2002;3(3):321-49.

116. Blum CA, Xu M, Orner GA, Díaz GD, Li Q, Dashwood WM, et al., editors. Promotion versus suppression of rat colon carcinogenesis by chlorophyllin and chlorophyll: Modulation of apoptosis, cell proliferation, and β-catenin/Tcf signaling. Mutation Research - Fundamental and Molecular Mechanisms of Mutagenesis; 2003.

117. Castro GD, Delgado de Layño AM, Costantini MH, Castro JA. Rat breast microsomal biotransformation of ethanol to acetaldehyde but not to free radicals: its potential role in the association between alcohol drinking and breast tumor promotion. Teratog Carcinog Mutagen. 2003;Suppl 1:61-70.

118. Castro GD, Delgado de Layño AMA, Costantini MH, Castro JA, editors. Rat breast microsomal biotransformation of ethanol to acetaldehyde but not to free radicals: Its potential role in the association between alcohol drinking and breast tumor promotion. Teratogenesis Carcinog Mutagen; 2003.

119. De Biase L, Pignatelli P, Lenti L, Tocci G, Piccioni F, Riondino S, et al. Enhanced TNFα and oxidative stress in patients with heart failure:: effect of TNFα on platelet O<sub>2</sub><SUP>-</SUP> production. Thromb Haemost. 2003;90(2):317-25.

120. Díaz GD, Li Q, Dashwood RH. Caspase-8 and apoptosis-inducing factor mediate a cytochrome c-independent pathway of apoptosis in human colon cancer cells induced by the dietary phytochemical chlorophyllin. Cancer Res. 2003;63(6):1254-61.

121. Díaz GD, Li QJ, Dashwood RH. Caspase-8 and apoptosis-inducing factor mediate a cytochrome <i>c</i>-independent pathway of apoptosis in human colon cancer cells induced by the dietary phytochemical chlorophyllin. Cancer Res. 2003;63(6):1254-61.

122. Kajon AE, Gigliotti AP, Harrod KS. Acute inflammatory response and remodeling of airway epithelium after subspecies B1 human adenovirus infection of the mouse lower respiratory tract. J Med Virol. 2003;71(2):233-44.

123. Kajon AE, Gigliotti AP, Harrod KS. Acute inflammatory response and remodeling of airway epithelium after subspecies B1 human adenovirus infection of the mouse lower respiratory tract. J Med Virol. 2003;71(2):233-44.

124. Kang SH, Song JH, Kang HK, Kang JH, Kim SJ, Kang HW, et al. Arsenic trioxide-induced apoptosis is independent of stress-responsive signaling pathways but sensitive to inhibition of inducible nitric oxide synthase in HepG2 cells. Exp Mol Med. 2003;35(2):83-90.

125. Kang SH, Song JH, Kang HK, Kang JH, Kim SJ, Kang HW, et al. Arsenic trioxide-induced apoptosis is independent of stress-responsive signaling pathways but sensitive to inhibition of inducible nitric oxide synthase in HepG2 cells. Exp Mol Med. 2003;35(2):83-90.

126. Kim HW, Murakami A, Williams MV, Ohigashi H. Mutagenicity of reactive oxygen and nitrogen species as detected by co-culture of activated inflammatory leukocytes and AS52 cells. Carcinogenesis. 2003;24(2):235-41.

127. Kim HW, Murakami A, Williams MV, Ohigashi H. Mutagenicity of reactive oxygen and nitrogen species as detected by co-culture of activated inflammatory leukocytes and AS52 cells. Carcinogenesis. 2003;24(2):235-41.

128. Li N, Ragheb K, Lawler G, Sturgis J, Rajwa B, Melendez JA, et al. DPI induces mitochondrial superoxide-mediated apoptosis. Free Radic Biol Med. 2003;34(4):465-77.

129. Meuillet EJ, Mahadevan D, Vankayalapati H, Berggren M, Williams R, Coon A, et al. Specific inhibition of the akt1 pleckstrin homology domain by D-3-deoxy-phosphatidyl-myo-inositol analogues. Mol Cancer Ther. 2003;2(4):389-99.

130. Meuillet EJ, Mahadevan D, Vankayalapati H, Berggren M, Williams R, Coon A, et al. Specific inhibition of the Akt1 pleckstrin homology domain by D-3-deoxy-phosphatidyl-<i>myo</i>-inositol analogues. Mol Cancer Ther. 2003;2(4):389-99.

131. Sauer H, Wefer K, Vetrugno V, Pocchiari M, Gissel C, Sachinidis A, et al. Regulation of intrinsic prion protein by growth factors and TNF-alpha: the role of intracellular reactive oxygen species. Free Radic Biol Med. 2003;35(6):586-94.

132. Sauer H, Wefer K, Vetrugno V, Pocchiari M, Gissel C, Sachinidis A, et al. Regulation of intrinsic prion protein by growth factors and TNF-α:: The role of intracellular reactive oxygen species. Free Radic Biol Med. 2003;35(6):586-94.

133. Shatrov VA, Brüne B. Induced expression of manganese superoxide dismutase by non-toxic concentrations of oxidized low-density lipoprotein (oxLDL) protects against oxLDL-mediated cytotoxicity. Biochem J. 2003;374(2):505-11.

134. Surh YJ. Cancer chemoprevention with dietary phytochemicals. Nature Reviews Cancer. 2003;3(10):768-80.

135. Surh YJ. Cancer chemoprevention with dietary phytochemicals. Nat Rev Cancer. 2003;3(10):768-80.

136. Zhang S, Qin C, Safe SH. Flavonoids as aryl hydrocarbon receptor agonists/antagonists: Effects of structure and cell context. Environmental Health Perspectives. 2003;111(16):1877-82.

137. Carter O, Bailey GS, Dashwood RH. The dietary phytochemical chlorophyllin alters E-cadherin and beta-catenin expression in human colon cancer cells. J Nutr. 2004;134(12 Suppl):3441s-4s.

138. Carter O, Bailey GS, Dashwood RH. The dietary phytochemical chlorophyllin alters e-cadherin and β-catenin expression in human colon cancer cells. J Nutr. 2004;134(12):3441S-4S.

139. Chen YC, Shen SC, Chow JM, Ko CH, Tseng SW. Flavone inhibition of tumor growth via apoptosis in vitro and in vivo. Int J Oncol. 2004;25(3):661-70.

140. Chen YC, Shen SC, Chow JM, Ko CH, Tseng SW. Flavone inhibition of tumor growth via apoptosis <i>in</i> <i>vitro</i> and <i>in</i> <i>vivo</i>. Int J Oncol. 2004;25(3):661-70.

141. Dodd-o JM, Welsh LE, Salazar JD, Walinsky PL, Peck EA, Shake JG, et al. Effect of NADPH oxidase inhibition on cardiopulmonary bypass-induced lung injury. Am J Physiol Heart Circ Physiol. 2004;287(2):H927-36.

142. Dodd-o JM, Welsh LE, Salazar JD, Walinsky PL, Peck EA, Shake JG, et al. Effect of NADPH oxidase inhibition on cardiopulmonary bypass-induced lung injury. Am J Physiol-Heart Circul Physiol. 2004;287(2):H927-H36.

143. Dong JM, Zhao SG, Huang GY, Liu Q. NADPH oxidase-mediated generation of reactive oxygen species is critically required for survival of undifferentiated human promyelocytic leukemia cell line HL-60. Free Radic Res. 2004;38(6):629-37.

144. Gujral JS, Hinson JA, Farhood A, Jaeschke H. NADPH oxidase-derived oxidant stress is critical for neutrophil cytotoxicity during endotoxemia. American Journal of Physiology - Gastrointestinal and Liver Physiology. 2004;287(1 50-1):G243-G52.

145. Higgins RJ, McKisic M, Dickinson PJ, Jimenez DF, Dow SW, Tripp LD, et al. Growth inhibition of an orthotopic glioblastoma in immunocompetent mice by cationic lipid-DNA complexes. Cancer Immunology, Immunotherapy. 2004;53(4):338-44.

146. Higgins RJ, McKisic M, Dickinson PJ, Jimenez DF, Dow SW, Tripp LD, et al. Growth inhibition of an orthotopic glioblastoma in immunocompetent mice by cationic lipid-DNA complexes. Cancer Immunol Immunother. 2004;53(4):338-44.

147. Holy J. Curcumin inhibits cell motility and alters microfilament organization and function in prostate cancer cells. Cell Motil Cytoskeleton. 2004;58(4):253-68.

148. Holy J. Curcumin inhibits cell motility and alters microfilament organization and function in prostate cancer cells. Cell Motil Cytoskeleton. 2004;58(4):253-68.

149. Kim YK, Lee JM, Kim CS. Gadobenate dimeglumine-enhanced liver MR imaging: Value of dynamic and delayed imaging for the characterization and detection of focal liver lesions. Eur Radiol. 2004;14(1):5-13.

150. Kim YK, Lee JM, Kim CS. Gadobenate dimeglumine-enhanced liver MR imaging: value of dynamic and delayed imaging for the characterization and detection of focal liver lesions. Eur Radiol. 2004;14(1):5-13.

151. Ko CH, Shen SC, Chen YC. Hydroxylation at C4′ or C6 is essential for apoptosis-inducing activity of flavanone through activation of the caspase-3 cascade and production of reactive oxygen species. Free Radic Biol Med. 2004;36(7):897-910.

152. Kundu JK, Surh YJ. Molecular basis of chemoprevention by resveratrol: NF-κB and AP-1 as potential targets. Mutation Research - Fundamental and Molecular Mechanisms of Mutagenesis. 2004;555(1-2 SPEC. ISS.):65-80.

153. Licanin Z, Lincender L, Djurović V, Salihefendić N, Smajlović F. [Color Doppler sonography of focal abdominal lesions]. Med Arh. 2004;58(1 Suppl 2):119-21.

154. McCarty MF. Proposal for a dietary "phytochemical index". Med Hypotheses. 2004;63(5):813-7.

155. Min KJ, Pyo HK, Yang MS, Ji KA, Jou I, Joe EH. Gangliosides activate microglia via protein kinase C and NADPH oxidase. Glia. 2004;48(3):197-206.

156. Mirza N, Schwartz SK, Antin-Ozerkis D. Laryngeal findings in users of combination corticosteroid and bronchodilator therapy. Laryngoscope. 2004;114(9 I):1566-9.

157. Morazzani M, De Carvalho DD, Kovacic H, Smida-Rezgui S, Briand C, Penel C. Monolayer versus aggregate balance in survival process for EGF-induced apoptosis in A431 carcinoma cells: Implication of ROS-P38 mapk-integrin A2B1 pathway. Int J Cancer. 2004;110(6):788-99.

158. Morazzani M, de Carvalho DD, Kovacic H, Smida-Rezgui S, Briand C, Penel C. Monolayer versus aggregate balance in survival process for EGF-induced apoptosis in A431 carcinoma cells: Implication of ROS-P38 MAPK-integrin alpha2beta1 pathway. Int J Cancer. 2004;110(6):788-99.

159. Nguyen TT, Tran E, Nguyen TH, Do PT, Huynh TH, Huynh H. The role of activated MEK-ERK pathway in quercetin-induced growth inhibition and apoptosis in A549 lung cancer cells. Carcinogenesis. 2004;25(5):647-59.

160. Ohnishi H, Asamoto M, Tujimura K, Hokaiwado N, Takahashi S, Ogawa K, et al. Inhibition of cell proliferation by nobiletin, a dietary phytochemical, associated with apoptosis and characteristic gene expression, but lack of effect on early rat hepatocarcinogenesis in vivo. Cancer Sci. 2004;95(12):936-42.

161. Ohnishi H, Asamoto M, Tujimura K, Hokaiwado N, Takahashi S, Ogawa K, et al. Inhibition of cell proliferation by nobiletin, a dietary phytochemical, associated with apoptosis and characteristic gene expression, but lack of effect on early rat hepatocarcinogenesis in vivo. Cancer Sci. 2004;95(12):936-42.

162. Pawate S, Shen Q, Fan F, Bhat NR. Redox regulation of glial inflammatory response to lipopolysaccharide and interferonγ. J Neurosci Res. 2004;77(4):540-51.

163. Shetty K, Wahlqvist M. A model for the role of the proline-linked pentosephosphate pathway in phenolic phytochemical biosynthesis and mechanism of action for human health and environmental applications. Asia Pacific Journal of Clinical Nutrition. 2004;13(1):1-24.

164. Singh RP, Sharma G, Mallikarjuna GU, Dhanalakshmi S, Agarwal C, Agarwal R. In vivo suppression of hormone-refractory prostate cancer growth by inositol hexaphosphate: induction of insulin-like growth factor binding protein-3 and inhibition of vascular endothelial growth factor. Clin Cancer Res. 2004;10(1 Pt 1):244-50.

165. Stewart JR, O'Brian CA. Resveratrol antagonizes EGFR-dependent Erk1/2 activation in human androgen-independent prostate cancer cells with associated isozyme-selective PKC alpha inhibition. Invest New Drugs. 2004;22(2):107-17.

166. Stewart JR, O'Brian CA. Resveratrol antagonizes EGFR-dependent Erk1/2 activation in human androgen-independent prostate cancer cells with associated isozyme-selective PKCα inhibition. Invest New Drugs. 2004;22(2):107-17.

167. Sun S, Han J, Ralph Jr WM, Chandrasekaran A, Liu K, Auborn KJ, et al. Endoplasmic reticulum stress as a correlate of cytotoxicity in human tumor cells exposed to diindolylmethane in vitro. Cell Stress and Chaperones. 2004;9(1):76-87.

168. Sun S, Han J, Ralph WM, Jr., Chandrasekaran A, Liu K, Auborn KJ, et al. Endoplasmic reticulum stress as a correlate of cytotoxicity in human tumor cells exposed to diindolylmethane in vitro. Cell Stress Chaperones. 2004;9(1):76-87.

169. Wang S, Leonard SS, Ye J, Gao N, Wang L, Shi X. Role of reactive oxygen species and Cr(VI) in Ras-mediated signal transduction. Mol Cell Biochem. 2004;255(1-2):119-27.

170. Xie Z, Singh M, Singh K. ERK1/2 and JNKs, but not p38 kinase, are involved in reactive oxygen species-mediated induction of osteopontin gene expression by angiotensin II and interleukin-1beta in adult rat cardiac fibroblasts. J Cell Physiol. 2004;198(3):399-407.

171. Xie ZL, Singh M, Singh K. ERK1/2 and JNKs, but not p38 kinase, are involved in reactive oxygen species-mediated induction of osteopontin gene expression by angiotensin II and interleukin-1β in adult rat cardiac fibroblasts. J Cell Physiol. 2004;198(3):399-407.

172. Ding Y, Chen ZJ, Liu S, Che D, Vetter M, Chang CH. Inhibition of Nox-4 activity by plumbagin, a plant-derived bioactive naphthoquinone. J Pharm Pharmacol. 2005;57(1):111-6.

173. Hebert C, Siavash H, Norris K, Nikitakis NG, Sauk JJ. Endostatin inhibits nitric oxide and diminishes VEGF and collagen XVIII in squamous carcinoma cells. Int J Cancer. 2005;114(2):195-201.

174. Kallio A, Zheng A, Dahllund J, Heiskanen KM, Härkönen P. Role of mitochondria in tamoxifen-induced rapid death of MCF-7 breast cancer cells. Apoptosis. 2005;10(6):1395-410.

175. Kersemans V, Cornelissen B, Bacher K, Kersemans K, Thierens H, Dierckx RA, et al. In vivo evaluation and dosimetry of 123I-2-iodo-D-phenylalanine, a new potential tumor-specific tracer for SPECT, in an R1M rhabdomyosarcoma athymic mouse model. J Nucl Med. 2005;46(12):2104-11.

176. Kersemans V, Cornelissen B, Bacher K, Kersemans K, Thierens H, Dierckx RA, et al. In vivo evaluation and dosimetry of <SUP>123</SUP>I-2-iodo-D-phenylalanine, a new potential tumor-specific tracer for SPECT in an R1M rhabdomyrosarcoma athymic mouse model. J Nucl Med. 2005;46(12):2104-11.

177. Kersemans V, Cornelissen B, Kersemans K, Bauwens M, Achten E, Dierckx RA, et al. In vivo characterization of 123/125I-2-iodo-L-phenylalanine in an R1M rhabdomyosarcoma athymic mouse model as a potential tumor tracer for SPECT. J Nucl Med. 2005;46(3):532-9.

178. Kersemans V, Cornelissen B, Kersemans K, Bauwens M, Achten E, Dierckx RA, et al. In vivo characterization of 123/125I-2-iodo-L-phenylalanine in an R1M rhabdomyosarcoma athymic mouse model as a potential tumor tracer for SPECT. J Nucl Med. 2005;46(3):532-9.

179. Kim BC, Kim HG, Lee SA, Lim S, Park EH, Kim SJ, et al. Genipin-induced apoptosis in hepatoma cells is mediated by reactive oxygen species/c-Jun NH2-terminal kinase-dependent activation of mitochondrial pathway. Biochem Pharmacol. 2005;70(9):1398-407.

180. Kim BC, Kim HG, Lee SA, Lim S, Park EH, Kim SJ, et al. Genipin-induced apoptosis in hepatoma cells is mediated by reactive oxygen species/c-Jun NH<sub>2</sub>-terminal kinase-dependent activation of mitochondrial pathway. Biochem Pharmacol. 2005;70(9):1398-407.

181. Lee NK, Choi YG, Baik JY, Han SY, Jeong DW, Bae YS, et al. A crucial role for reactive oxygen species in RANKL-induced osteoclast differentiation. Blood. 2005;106(3):852-9.

182. Lee NK, Choi YG, Baik JY, Han SY, Jeong DW, Bae YS, et al. A crucial role for reactive oxygen species in RANKL-induced osteoclast differentiation. Blood. 2005;106(3):852-9.

183. Lee YJ, Lee DH, Cho CK, Chung HY, Bae S, Jhon GJ, et al. HSP25 inhibits radiation-induced apoptosis through reduction of PKCδ-mediated ROS production. Oncogene. 2005;24(23):3715-25.

184. Nabekura T, Kamiyama S, Kitagawa S. Effects of dietary chemopreventive phytochemicals on P-glycoprotein function. Biochem Biophys Res Commun. 2005;327(3):866-70.

185. Qin L, Li G, Qian X, Liu Y, Wu X, Liu B, et al. Interactive role of the toll-like receptor 4 and reactive oxygen species in LPS-induced microglia activation. Glia. 2005;52(1):78-84.

186. Roumen RMH, Scheltinga MRM, Slooter GD, van der Linden AWM. Doppler perfusion index fails to predict the presence of occult hepatic colorectal metastases. European Journal of Surgical Oncology. 2005;31(5):521-7.

187. Roumen RMH, Scheltinga MRM, Slooter GD, van der Linden AWM. Doppler perfusion index fails to predict the presence of occult hepatic colorectal metastases. Ejso. 2005;31(5):521-7.

188. Shahiwala A, Misra A. A preliminary pharmacokinetic study of liposomal leuprolide dry powder inhaler: A technical note. AAPS PharmSciTech. 2005;6(3).

189. Singh RP, Agarwal R. Prostate cancer and inositol hexaphosphate: Efficacy and mechanisms. Anticancer Res. 2005;25(4):2891-904.

190. Steinbrenner H, Ramos MC, Stuhlmann D, Mitic D, Sies H, Brenneisen P. Tumor promoter TPA stimulates MMP-9 secretion from human keratinocytes by activation of superoxide-producing NADPH oxidase. Free Radic Res. 2005;39(3):245-53.

191. Steinbrenner H, Ramos MC, Stuhlmann D, Mitic D, Sies H, Brenneisen P. Tumor promoter TPA stimulates MMP-9 secretion from human keratinocytes by activation of superoxide-producing NADPH oxidase. Free Radic Res. 2005;39(3):245-53.

192. Traka M, Gasper AV, Smith JA, Hawkey CJ, Bao Y, Mithen RF. Transcriptome analysis of human colon Caco-2 cells exposed to sulforaphane. J Nutr. 2005;135(8):1865-72.

193. Traka M, Gasper AV, Smith JA, Hawkey CJ, Bao YP, Mithen RF. Transcriptome analysis of human colon Caco-2 cells exposed to sulforaphane. J Nutr. 2005;135(8):1865-72.

194. Wartenberg M, Gronczynska S, Bekhite MM, Saric T, Niedermeier W, Hescheler J, et al. Regulation of the multidrug resistance transporter P-glycoprotein in multicellular prostate tumor spheroids by hyperthermia and reactive oxygen species. Int J Cancer. 2005;113(2):229-40.

195. Wartenberg M, Gronczynska S, Bekhite MM, Saric T, Niedermeier W, Hescheler J, et al. Regulation of the multidrug resistance transporter P-glycoprotein in multicellular prostate tumor spheroids by hyperthermia and reactive oxygen species. Int J Cancer. 2005;113(2):229-40.

196. Wartenberg M, Hoffmann E, Schwindt H, Grünheck F, Petros J, Arnold JRS, et al. Reactive oxygen species-linked regulation of the multidrug resistance transporter P-glycoprotein in Nox-1 overexpressing prostate tumor spheroids. FEBS Lett. 2005;579(20):4541-9.

197. Yamashina S, Takei Y, Ikejima K, Enomoto N, Kitamura T, Sato N, editors. Ethanol-induced sensitization to endotoxin in Kupffer cells is dependent upon oxidative stress. Alcoholism: Clinical and Experimental Research; 2005.

198. Bárta I, Smerák P, Polívková Z, Sestáková H, Langová M, Turek B, et al. Current trends and perspectives in nutrition and cancer prevention. Neoplasma. 2006;53(1):19-25.

199. Bobe G, Wang B, Seeram NP, Nair MG, Bourquin LD. Dietary anthocyanin-rich tart cherry extract inhibits intestinal tumorigenesis in APC(Min) mice fed suboptimal levels of sulindac. J Agric Food Chem. 2006;54(25):9322-8.

200. Chien PS, Mak OT, Huang HJ. Induction of COX-2 protein expression by vanadate in A549 human lung carcinoma cell line through EGF receptor and p38 MAPK-mediated pathway. Biochem Biophys Res Commun. 2006;339(2):562-8.

201. Chien PS, Mak OT, Huang HJ. Induction of COX-2 protein expression by vanadate in A549 human lung carcinoma cell line through EGF receptor and p38 MAPK-mediated pathway. Biochem Biophys Res Commun. 2006;339(2):562-8.

202. Cho SO, Kim KH, Yoon JH, Kim H. Signaling for integrin alpha5/beta1 expression in Helicobacter pylori-infected gastric epithelial AGS cells. Ann N Y Acad Sci. 2006;1090:298-304.

203. Choi C, Kwon D, Jung K, Ha Y, Lee YH, Kim O, et al. Expression of inflammatory cytokines in pigs experimentally infected with Mycoplasma hyopneumoniae. J Comp Pathol. 2006;134(1):40-6.

204. Choi C, Kwon D, Jung K, Ha Y, Lee YH, Kim O, et al. Expression of inflammatory cytokines in pigs experimentally infected with <i>Mycoplasma hyopneumoniae</i>. J Comp Pathol. 2006;134(1):40-6.

205. Chow JM, Shen SC, Wu CY, Chen YC. 12-<i>o</i>-tetradecanoylphorbol 13-acetate prevents baicalein-induced apoptosis via activation of protein kinase C and JNKs in human leukemia cells. Apoptosis. 2006;11(11):1999-2011.

206. Gustot T, Lemmers A, Moreno C, Nagy N, Quertinmont E, Nicaise C, et al. Differential liver sensitization to toll-like receptor pathways in mice with alcoholic fatty liver. Hepatology. 2006;43(5):989-1000.

207. Gustot T, Lemmers A, Moreno C, Nagy N, Quertinmont E, Nicaise C, et al. Differential liver sensitization to toll-like receptor pathways in mice with alcoholic fatty liver. Hepatology. 2006;43(5):989-1000.

208. Jeronimo J, Long R, Neve L, Ferris D, Noller K, Spitzer M, et al. Preparing digitized cervigrams for colposcopy research and education: Determination of optimal resolution and compression parameters. Journal of Lower Genital Tract Disease. 2006;10(1):39-44.

209. Kersemans V, Cornelissen B, Kersemans K, Dierckx RA, De Spiegeleer B, Mertens J, et al. Comparative biodistribution study of the new tumor tracer [123I]-2-iodo-L-phenylalanine with [123I]-2-iodo-L-tyrosine. Nucl Med Biol. 2006;33(1):111-7.

210. Kersemans V, Cornelissen B, Kersemans K, Dierckx RA, De Spiegeleer B, Mertens J, et al. Comparative biodistribution study of the new tumor tracer <SUP>123</SUP>I -2-iodo-L-phenylalanine with <SUP>123</SUP>I -2-iodo-L-tyrosine. Nucl Med Biol. 2006;33(1):111-7.

211. Köhle C, Bock KW. Activation of coupled Ah receptor and Nrf2 gene batteries by dietary phytochemicals in relation to chemoprevention. Biochem Pharmacol. 2006;72(7):795-805.

212. Komine Y, Komine K, Kai K, Itagaki M, Kuroishi T, Aso H, et al. Effect of combination therapy with lactoferrin and antibiotics against staphylococcal mastitis on drying cows. J Vet Med Sci. 2006;68(3):205-11.

213. Komine Y, Komine KI, Kai K, Itagaki M, Kuroishi T, Aso H, et al. Effect of combination therapy with lactoferrin and antibiotics against staphylococcal mastitis on drying cows. J Vet Med Sci. 2006;68(3):205-11.

214. Mandlekar S, Hong JL, Kong AN. Modulation of metabolic enzymes by dietary phytochemicals: a review of mechanisms underlying beneficial versus unfavorable effects. Curr Drug Metab. 2006;7(6):661-75.

215. Mochizuki T, Furuta S, Mitsushita J, Shang WH, Ito M, Yokoo Y, et al. Inhibition of NADPH oxidase 4 activates apoptosis via the AKT/apoptosis signal-regulating kinase 1 pathway in pancreatic cancer PANC-1 cells. Oncogene. 2006;25(26):3699-707.

216. Mochizuki T, Furuta S, Mitsushita J, Shang WH, Ito M, Yokoo Y, et al. Inhibition of NADPH oxidase 4 activates apoptosis via the AKT/apoptosis signal-regulating kinase 1 pathway in pancreatic cancer PANC-1 cells. Oncogene. 2006;25(26):3699-707.

217. Ohori H, Yamakoshi H, Tomizawa M, Shibuya M, Kakudo Y, Takahashi A, et al. Synthesis and biolgical analysis of new curcumin analogues bearing an enhanced potential for the medicinal treatment of cancer. Mol Cancer Ther. 2006;5(10):2563-71.

218. Ohori H, Yamakoshi H, Tomizawa M, Shibuya M, Kakudo Y, Takahashi A, et al. Synthesis and biological analysis of new curcumin analogues bearing an enhanced potential for the medicinal treatment of cancer. Mol Cancer Ther. 2006;5(10):2563-71.

219. Porthouse KH, Chirgwin SR, Coleman SU, Taylor HW, Klei TR. Inflammatory responses to migrating Brugia pahangi third-stage larvae. Infect Immun. 2006;74(4):2366-72.

220. Porthouse KH, Chirgwin SR, Coleman SU, Taylor HW, Klei TR. Inflammatory responses to migrating Brugia pahangi third-stage larvae. Infect Immun. 2006;74(4):2366-72.

221. Psalla D, Psychas V, Spyrou V, Billinis C, Papaioannou N, Vlemmas I. Pathogenesis of Experimental Encephalomyocarditis: A Histopathological, Immunohistochemical and Virological Study in Mice. J Comp Pathol. 2006;135(2-3):142-5.

222. Reis K, Hälldin J, Fernaeus S, Pettersson C, Land T. NADPH oxidase inhibitor diphenyliodonium abolishes lipopolysaccharide- induced down-regulation of transferrin receptor expression in N2a and BV-2 cells. J Neurosci Res. 2006;84(5):1047-52.

223. Reis K, Hälldin J, Fernaeus S, Pettersson C, Land T. NADPH oxidase inhibitor diphenyliodonium abolishes lipopolysaccharide-induced down-regulation of transferrin receptor expression in N2a and BV-2 cells. J Neurosci Res. 2006;84(5):1047-52.

224. Schaefer CA, Kuhlmann CR, Weiterer S, Fehsecke A, Abdallah Y, Schaefer C, et al. Statins inhibit hypoxia-induced endothelial proliferation by preventing calcium-induced ROS formation. Atherosclerosis. 2006;185(2):290-6.

225. Schaefer CA, Kuhlmann CRW, Weiterer S, Fehsecke A, Abdallah Y, Schaefer C, et al. Statins inhibit hypoxia-induced endothelial proliferation by preventing calcium-induced ROS formation. Atherosclerosis. 2006;185(2):290-6.

226. Siddiqui IA, Zaman N, Aziz MH, Reagan-Shaw SR, Sarfaraz S, Adhami VM, et al. Inhibition of CWR22Rν1 tumor growth and PSA secretion in athymic nude mice by green and black teas. Carcinogenesis. 2006;27(4):833-9.

227. Thakur V, Pritchard MT, McMullen MR, Wang Q, Nagy LE. Chronic ethanol feeding increases activation of NADPH oxidase by lipopolysaccharide in rat Kupffer cells: Role of increased reactive oxygen in LPS-stimulated ERK1/2 activation and TNF-α production. J Leukoc Biol. 2006;79(6):1348-56.

228. Thakur V, Pritchard MT, McMullen MR, Wang Q, Nagy LE. Chronic ethanol feeding increases activation of NADPH oxidase by lipopolysaccharide in rat Kupffer cells: role of increased reactive oxygen in LPS-stimulated ERK1/2 activation and TNF-alpha production. J Leukoc Biol. 2006;79(6):1348-56.

229. Zhao Y, Chaiswing L, Bakthavatchalu V, Oberley TD, St Clair DK. Ras mutation promotes p53 activation and apoptosis of skin keratinocytes. Carcinogenesis. 2006;27(8):1692-8.

230. Becher R, Bucht A, Øvrevik J, Hongslo JK, Dahlman HJ, Samuelsen JT, et al. Involvement of NADPH oxidase and iNOS in rodent pulmonary cytokine responses to urban air and mineral particles. Inhal Toxicol. 2007;19(8):645-55.

231. Becher R, Bucht A, Øvrevik J, Hongslo JK, Dahlman HJ, Samuelsen JT, et al. Involvement of NADPH oxidase and iNOS in rodent pulmonary cytokine responses to urban air and mineral particles. Inhal Toxicol. 2007;19(8):645-55.

232. Block K, Gorin Y, Hoover P, Williams P, Chelmicki T, Clark RA, et al. NAD(P)H oxidases regulate HIF-2alpha protein expression. J Biol Chem. 2007;282(11):8019-26.

233. Block K, Gorin Y, Hoover P, Williams P, Chelmicki T, Clark RA, et al. NAD(P)H oxidases regulate HIF-2α protein expression. J Biol Chem. 2007;282(11):8019-26.

234. Chang ET, Lee VS, Canchola AJ, Clarke CA, Purdie DM, Reynolds P, et al. Diet and risk of ovarian cancer in the California Teachers Study cohort. Am J Epidemiol. 2007;165(7):802-13.

235. Chang ET, Lee VS, Canchola AJ, Clarke CA, Purdie DM, Reynolds P, et al. Diet and risk of ovarian cancer in the California teachers study cohort. Am J Epidemiol. 2007;165(7):802-13.

236. Gerhauser I, Ulrich R, Alldinger S, Baumgärtner W. Induction of activator protein-1 and nuclear factor-κB as a prerequisite for disease development in susceptible SJL/J mice after theiler murine encephalomyelitis. J Neuropathol Exp Neurol. 2007;66(9):809-18.

237. Gerhauser I, Ulrich R, Alldinger S, Baumgärtner W. Induction of activator protein-1 and nuclear factor-kappaB as a prerequisite for disease development in susceptible SJL/J mice after theiler murine encephalomyelitis. J Neuropathol Exp Neurol. 2007;66(9):809-18.

238. Hong HY, Kim BC. Mixed lineage kinase 3 connects reactive oxygen species to c-Jun NH2-terminal kinase-induced mitochondrial apoptosis in genipin-treated PC3 human prostate cancer cells. Biochem Biophys Res Commun. 2007;362(2):307-12.

239. Ko CH, Shen SC, Yang LY, Lin CW, Chen YC. Gossypol reduction of tumor growth through ROS-dependent mitochondria pathway in human colorectal carcinoma cells. Int J Cancer. 2007;121(8):1670-9.

240. Ko CH, Shen SC, Yang LY, Lin CW, Chen YC. Gossypol reduction of tumor growth through ROS-dependent mitochondria pathway in human colorectal carcinoma cells. Int J Cancer. 2007;121(8):1670-9.

241. Kundu JK, Surh YJ, editors. Epigallocatechin Gallate inhibits phorbol ester-induced activation of NF-κB and CREB in mouse skin role of p38 MAPK. Annals of the New York Academy of Sciences; 2007.

242. KuoLee R, Zhao X, Austin J, Harris G, Conlan JW, Chen W. Mouse model of oral infection with virulent type A Francisella tularensis. Infect Immun. 2007;75(4):1651-60.

243. KuoLee R, Zhao X, Austin J, Harris G, Conlan JW, Chen W. Mouse model of oral infection with virulent type A <i>Francisella tularensis</i>. Infect Immun. 2007;75(4):1651-60.

244. Kwon KH, Barve A, Yu S, Huang MT, Kong AN. Cancer chemoprevention by phytochemicals: potential molecular targets, biomarkers and animal models. Acta Pharmacol Sin. 2007;28(9):1409-21.

245. Lambert IH. Activation and inactivation of the volume-sensitive taurine leak pathway in NIH3T3 fibroblasts and Ehrlich Lettre ascites cells. American Journal of Physiology - Cell Physiology. 2007;293(1):C390-C400.

246. Lin YC, Uang HW, Lin RJ, Chen IJ, Lo YC. Neuroprotective effects of glyceryl nonivamide against microglia-like cells and 6-hydroxydopamine-induced neurotoxicity in SH-SY5Y human dopaminergic neuroblastoma cells. J Pharmacol Exp Ther. 2007;323(3):877-87.

247. Lin YC, Uang HW, Lin RJ, Chen IJ, Lo YC. Neuroprotective effects of glyceryl nonivamide against microglia-like cells and 6-hydroxydopamine-induced neurotoxicity in SH-SY5Y human Dopaminergic neuroblastoma cells. J Pharmacol Exp Ther. 2007;323(3):877-87.

248. Mahipal SVK, Subhashini J, Reddy MC, Reddy MM, Anilkumar K, Roy KR, et al. Effect of 15-lipoxygenase metabolites, 15-(S)-HPETE and 15-(S)-HETE on chronic myelogenous leukemia cell line K-562: Reactive oxygen species (ROS) mediate caspase-dependent apoptosis. Biochem Pharmacol. 2007;74(2):202-14.

249. Manson MM, Foreman BE, Howells LM, Moiseeva EP. Determining the efficacy of dietary phytochemicals in cancer prevention. Biochem Soc Trans. 2007;35(Pt 5):1358-63.

250. Meyns T, Maes D, Calus D, Ribbens S, Dewulf J, Chiers K, et al. Interactions of highly and low virulent Mycoplasma hyopneumoniae isolates with the respiratory tract of pigs. Vet Microbiol. 2007;120(1-2):87-95.

251. Moiseeva EP, Almeida GM, Jones GD, Manson MM. Extended treatment with physiologic concentrations of dietary phytochemicals results in altered gene expression, reduced growth, and apoptosis of cancer cells. Mol Cancer Ther. 2007;6(11):3071-9.

252. Murillo MM, Carmona-Cuenca I, Del Castillo G, Ortiz C, Roncero C, Sánchez A, et al. Activation of NADPH oxidase by transforming growth factor-β in hepatocytes mediates up-regulation of epidermal growth factor receptor ligands through a nuclear factor-κB-dependent mechanism. Biochem J. 2007;405(2):251-9.

253. Nair S, Li W, Kong AN. Natural dietary anti-cancer chemopreventive compounds: redox-mediated differential signaling mechanisms in cytoprotection of normal cells versus cytotoxicity in tumor cells. Acta Pharmacol Sin. 2007;28(4):459-72.

254. Nair S, Li W, Kong ANT. Natural dietary anti-cancer chemopreventive compounds: Redox-mediated differential signaling mechanisms in cytoprotection of normal cells versus cytotoxicity in tumor cells. Acta Pharmacol Sin. 2007;28(4):459-72.

255. Nitti M, Furfaro AL, Traverso N, Odetti P, Storace D, Cottalasso D, et al. PKC delta and NADPH oxidase in AGE-induced neuronal death. Neurosci Lett. 2007;416(3):261-5.

256. Non L, Duong D, Peehl DM. Chemopreventive anti-inflammatory activities of curcumin and other phytochemicals mediated by MAP kinase phosphatase-5 in prostate cells. Carcinogenesis. 2007;28(6):1188-96.

257. Payne CM, Weber C, Crowley-Skillicorn C, Dvorak K, Bernstein H, Bernstein C, et al. Deoxycholate induces mitochondrial oxidative stress and activates NF-κB through multiple mechanisms in HCT-116 colon epithelial cells. Carcinogenesis. 2007;28(1):215-22.

258. Payne CM, Weber C, Crowley-Skillicorn C, Dvorak K, Bernstein H, Bernstein C, et al. Deoxycholate induces mitochondrial oxidative stress and activates NF-kappaB through multiple mechanisms in HCT-116 colon epithelial cells. Carcinogenesis. 2007;28(1):215-22.

259. Russo GL. Ins and outs of dietary phytochemicals in cancer chemoprevention. Biochem Pharmacol. 2007;74(4):533-44.

260. Tilton SC, Hendricks JD, Orner GA, Pereira CB, Bailey GS, Williams DE. Gene expression analysis during tumor enhancement by the dietary phytochemical, 3,3′-diindolylmethane, in rainbow trout. Carcinogenesis. 2007;28(7):1589-98.

261. Tilton SC, Hendricks JD, Orner GA, Pereira CB, Bailey GS, Williams DE. Gene expression analysis during tumor enhancement by the dietary phytochemical, 3,3'-diindolylmethane, in rainbow trout. Carcinogenesis. 2007;28(7):1589-98.

262. Tsuji PA, Walle T. Benzo[a]pyrene-induced cytochrome P450 1A and DNA binding in cultured trout hepatocytes-Inhibition by plant polyphenols. Chem-Biol Interact. 2007;169(1):25-31.

263. Xia C, Meng Q, Liu LZ, Rojanasakul Y, Wang XR, Jiang BH. Reactive oxygen species regulate angiogenesis and tumor growth through vascular endothelial growth factor. Cancer Res. 2007;67(22):10823-30.

264. Xia C, Meng Q, Liu LZ, Rojanasakul Y, Wang XR, Jiang BH. Reactive oxygen species regulate angiogenesis and tumor growth through vascular endothelial growth factor. Cancer Res. 2007;67(22):10823-30.

265. Barve A, Khor TO, Hao X, Keum YS, Yang CS, Reddy B, et al. Murine prostate cancer inhibition by dietary phytochemicals - Curcumin and phenyethylisothiocyanate. Pharm Res. 2008;25(9):2181-9.

266. Chandor A, Dijols S, Ramassamy B, Frapart Y, Mansuy D, Stuehr D, et al. Metabolic activation of the antitumor drug 5-(Aziridin-1-yl)-2,4-dinitrobenzamide (CB1954) by NO synthases. Chem Res Toxicol. 2008;21(4):836-43.

267. de Carvalho DD, Sadok A, Bourgarel-Rey V, Gattacceca F, Penel C, Lehmann M, et al. Nox1 downstream of 12-lipoxygenase controls cell proliferation but not cell spreading of colon cancer cells. Int J Cancer. 2008;122(8):1757-64.

268. de Carvalho DD, Sadolk A, Bourgarel-Rey V, Gattacceca F, Penel C, Lehmann M, et al. Nox1 downstream of 12-lipoxygenase controls cell proliferation but not cell spreading of colon cancer cells. Int J Cancer. 2008;122(8):1757-64.

269. Eggler AL, Gay KA, Mesecar AD. Molecular mechanisms of natural products in chemoprevention: induction of cytoprotective enzymes by Nrf2. Mol Nutr Food Res. 2008;52 Suppl 1:S84-94.

270. Feagins LA, Zhang HY, Zhang X, Hormi-Carver K, Thomas T, Terada LS, et al. Mechanisms of oxidant production in esophageal squamous cell and Barrett's cell lines. Am J Physiol Gastrointest Liver Physiol. 2008;294(2):G411-7.

271. Feagins LA, Zhang HY, Zhang X, Hormi-Carver K, Thomas T, Terada LS, et al. Mechanisms of oxidant production in esophageal squamous cell and Barrett's cell lines. Am J Physiol-Gastroint Liver Physiol. 2008;294(2):G411-G7.

272. Gopalakrishnan A, Tony Kong AN. Anticarcinogenesis by dietary phytochemicals: cytoprotection by Nrf2 in normal cells and cytotoxicity by modulation of transcription factors NF-kappa B and AP-1 in abnormal cancer cells. Food Chem Toxicol. 2008;46(4):1257-70.

273. Hsieh TC, Wu JM. Suppression of cell proliferation and gene expression by combinatorial synergy of EGCG, resveratrol and γ-tocotrienol in estrogen receptor-positive MCF-7 breast cancer cells. Int J Oncol. 2008;33(4):851-9.

274. Huang D, Chen Y, Li K, Zhang Q. Hemodynamic changes on color Doppler flow imaging and intravenous contrast-enhanced ultrasound for assessing transplanted liver and early diagnosis of complications. J Huazhong Univ Sci Technolog Med Sci. 2008;28(3):284-6.

275. Huang DZ, Chen YC, Li KY, Zhang QP. Hemodynamic changes on color Doppler flow imaging and intravenous contrast-enhanced ultrasound for assessing transplanted liver and early diagnosis of complications. J Huazhong Univ Sci Tech-Med. 2008;28(3):284-6.

276. Jin S, Ray RM, Johnson LR. TNF-alpha/cycloheximide-induced apoptosis in intestinal epithelial cells requires Rac1-regulated reactive oxygen species. Am J Physiol Gastrointest Liver Physiol. 2008;294(4):G928-37.

277. Kim H, Hwang JS, Woo CH, Kim EY, Kim TH, Cho KJ, et al. TNF-alpha-induced up-regulation of intercellular adhesion molecule-1 is regulated by a Rac-ROS-dependent cascade in human airway epithelial cells. Exp Mol Med. 2008;40(2):167-75.

278. Kim H, Hwang JS, Woo CH, Kim EY, Kim TH, Cho KJ, et al. TNF-α-induced up-regulation of intercellular adhesion molecule-1 is regulated by a Rac-ROS-dependent cascade in human airway epithelial cells. Exp Mol Med. 2008;40(2):167-75.

279. Kim JI, Lee JM, Choi JY, Kim YK, Kim SH, Lee JY, et al. The value of gadobenate dimeglumine-enhanced delayed phase MR imaging for characterization of hepatocellular nodules in the cirrhotic liver. Invest Radiol. 2008;43(3):202-10.

280. Kim JI, Lee JM, Choi JY, Kim YK, Kim SH, Lee JY, et al. The value of gadobenate dimeglumine-enhanced delayed phase MR imaging for characterization of hepatocellular nodules in the cirrhotic liver. Invest Radiol. 2008;43(3):202-10.

281. Komatsu D, Kato M, Nakayama J, Miyagawa S, Kamata T. NADPH oxidase 1 plays a critical mediating role in oncogenic Ras-induced vascular endothelial growth factor expression. Oncogene. 2008;27(34):4724-32.

282. Komatsu D, Kato M, Nakayama J, Miyagawa S, Kamata T. NADPH oxidase 1 plays a critical mediating role in oncogenic Ras-induced vascular endothelial growth factor expression. Oncogene. 2008;27(34):4724-32.

283. Kyriakopoulou K, Antoniou A, Fezoulidis IV, Kelekis NL, Dalekos GN, Vlychou M. The role of Doppler Perfusion Index as screening test in the characterization of focal liver lesions. Dig Liver Dis. 2008;40(9):755-60.

284. Kyriakopoulou K, Antoniou A, Fezoulidis IV, Kelekis NL, Dalekos GN, Vlychou M. The role of Doppler Perfusion Index as screening test in the characterization of focal liver lesions. Dig Liver Dis. 2008;40(9):755-60.

285. Nabekura T, Yamaki T, Ueno K, Kitagawa S. Inhibition of P-glycoprotein and multidrug resistance protein 1 by dietary phytochemicals. Cancer Chemother Pharmacol. 2008;62(5):867-73.

286. Nabekura T, Yamaki T, Ueno K, Kitagawa S. Inhibition of P-glycoprotein and multidrug resistance protein 1 by dietary phytochemicals. Cancer Chemother Pharmacol. 2008;62(5):867-73.

287. Nguyen HH, Aronchik I, Brar GA, Nguyen DH, Bjeldanes LF, Firestone GL. The dietary phytochemical indole-3-carbinol is a natural elastase enzymatic inhibitor that disrupts cyclin E protein processing. Proc Natl Acad Sci U S A. 2008;105(50):19750-5.

288. Nguyen HH, Aronchik I, Brar GA, Nguyen DHH, Bjeldanes LF, Firestone GL. The dietary phytochemical indole-3-carbinol is a natural elastase enzymatic inhibitor that disrupts cyclin e protein processing. Proc Natl Acad Sci U S A. 2008;105(50):19750-5.

289. Nisimoto Y, Tsubouchi R, Diebold BA, Qiao S, Ogawa H, Ohara T, et al. Activation of NADPH oxidase 1 in tumour colon epithelial cells. Biochem J. 2008;415(1):57-65.

290. Nisimoto Y, Tsubouchi R, Diebold BA, Qiao S, Ogawas H, Ohara T, et al. Activation of NADPH oxidase 1 in tumour colon epithelial cells. Biochem J. 2008;415(1):57-65.

291. Overhoff KA, Clayborough R, Crowley M. Review of the TAIFUN multidose dry powder inhaler technology. Drug Dev Ind Pharm. 2008;34(9):960-5.

292. Overhoff KA, Clayborough R, Crowley M. Review of the TAIFUN® multidose dry powder inhaler technology. Drug Dev Ind Pharm. 2008;34(9):960-5.

293. Parkin DR, Lu Y, Bliss RL, Malejka-Giganti D. Inhibitory effects of a dietary phytochemical 3,3'-diindolylmethane on the phenobarbital-induced hepatic CYP mRNA expression and CYP-catalyzed reactions in female rats. Food Chem Toxicol. 2008;46(7):2451-8.

294. Roy M, Sinha D, Mukherjee S, Paul S, Bhattacharya RK. Protective effect of dietary phytochemicals against arsenite induced genotoxicity in mammalian V79 cells. Indian Journal of Experimental Biology. 2008;46(10):690-7.

295. Ryan E, Horsington J, Brownlie J, Zhang Z. Foot-and-Mouth Disease Virus Infection in Fetal Lambs: Tissue Tropism and Cytokine Response. J Comp Pathol. 2008;138(2-3):108-20.

296. Son TG, Camandola S, Mattson MP. Hormetic dietary phytochemicals. NeuroMolecular Medicine. 2008;10(4):236-46.

297. Song JD, Kim KM, Kim KH, Kim CD, Kim JM, Yoo YH, et al. Differential role of diphenyleneiodonium, a flavoenzyme inhibitor, on p53-dependent and -independent cell cycle progression. Int J Oncol. 2008;33(6):1299-306.

298. Sun J, Liu RH. Apple phytochemical extracts inhibit proliferation of estrogen-dependent and estrogen-independent human breast cancer cells through cell cycle modulation. J Agric Food Chem. 2008;56(24):11661-7.

299. Surh YJ, Kundu JK, Na HK. Nrf2 as a master redox switch in turning on the cellular signaling involved in the induction of cytoprotective genes by some chemopreventive phytochemicals. Planta Medica. 2008;74(13):1526-39.

300. Venkatachalam P, De Toledo SM, Pandey BN, Tephly LA, Carter AB, Little JB, et al. Regulation of normal cell cycle progression by flavin-containing oxidases. Oncogene. 2008;27(1):20-31.

301. Wang J, Li L, Cang H, Shi G, Yi J. NADPH oxidase-derived reactive oxygen species are responsible for the high susceptibility to arsenic cytotoxicity in acute promyelocytic leukemia cells. Leukemia Research. 2008;32(3):429-36.

302. Wang YJ, Lin MW, Lin AA, Peng H, Wu SN. Evidence for state-dependent block of DPI 201-106, a synthetic inhibitor of NA+ channel inactivation, on delayed-rectifier K+ current in pituitary tumor (GH3) cells. J Physiol Pharmacol. 2008;59(3):409-23.

303. Wang YJ, Lin MW, Lin AA, Peng H, Wu SN. Evidence for state-dependent block of DPI 201-106, a synthetic inhibitor of Na+ channel inactivation, on delayed-rectifier K+ current in pituitary tumor (GH3) cells. J Physiol Pharmacol. 2008;59(3):409-23.

304. Zhao Y, Liu J, McMartin KE. Inhibition of NADPH oxidase activity promotes differentiation of B16 melanoma cells. Oncol Rep. 2008;19(5):1225-30.

305. Assanah MC, Bruce JN, Suzuki SO, Chen A, Goldman JE, Canoll P. PDGF stimulates the massive expansion of glial progenitors in the neonatal forebrain. Glia. 2009;57(16):1835-47.

306. Assanah MC, Bruce JN, Suzuki SO, Chen A, Goldman JE, Canoll P. PDGF stimulates the massive expansion of glial progenitors in the neonatal forebrain. Glia. 2009;57(16):1835-47.

307. Barh D. Biomarkers, critical disease pathways, drug targets, and alternative medicine in male breast cancer. Curr Drug Targets. 2009;10(1):1-8.

308. Chiu-Tsao ST, Chan MF. Photon beam dosimetry in the superficial buildup region using radiochromic EBT film stack. Med Phys. 2009;36(6):2074-83.

309. Chiu-Tsao ST, Chan MF. Photon beam dosimetry in the superficial buildup region using radiochromic EBT film stack. Med Phys. 2009;36(6):2074-83.

310. Daghestani HN, Fernig DG, Day BW. Evaluation of biosensor surfaces for the detection of microtubule perturbation. Biosens Bioelectron. 2009;25(1):136-41.

311. Daghestani HN, Fernig DG, Day BW. Evaluation of biosensor surfaces for the detection of microtubule perturbation. Biosens Bioelectron. 2009;25(1):136-41.

312. Das I, Saha T. Effect of garlic on lipid peroxidation and antioxidation enzymes in DMBA-induced skin carcinoma. Nutrition. 2009;25(4):459-71.

313. Firestone GL, Sundar SN. Minireview: modulation of hormone receptor signaling by dietary anticancer indoles. Mol Endocrinol. 2009;23(12):1940-7.

314. Hallak M, Win T, Shpilberg O, Bittner S, Granot Y, Levy I, et al. The anti-leukaemic activity of novel synthetic naphthoquinones against acute myeloid leukaemia: induction of cell death via the triggering of multiple signalling pathways. Br J Haematol. 2009;147(4):459-70.

315. Hallak M, Win T, Shpilberg O, Bittner S, Granot Y, Levy I, et al. The anti-leukaemic activity of novel synthetic naphthoquinones against acute myeloid leukaemia: induction of cell death via the triggering of multiple signalling pathways. Br J Haematol. 2009;147(4):459-70.

316. Hsieh TC, Wu JM. Targeting CWR22Rv1 prostate cancer cell proliferation and gene expression by combinations of the phytochemicals EGCG, genistein and quercetin. Anticancer Res. 2009;29(10):4025-32.

317. Kennedy BC, Maier LM, D'Amico R, Mandigo CE, Fontana EJ, Waziri A, et al. Dynamics of central and peripheral immunomodulation in a murine glioma model. BMC Immunol. 2009;10:11.

318. Kennedy BC, Maier LM, D'Amico R, Mandigo CE, Fontana EJ, Waziri A, et al. Dynamics of central and peripheral immunomodulation in a murine glioma model. BMC Immunol. 2009;10:8.

319. Kim B, Ahn KK, Ha Y, Lee YH, Kim D, Lim JH, et al. Association of Tumor Necrosis Factor-α with Fever and Pulmonary Lesion Score in Pigs Experimentally Infected with Swine Influenza Virus Subtype H1N2. J Vet Med Sci. 2009;71(5):611-6.

320. Kim B, Ahn KK, Ha Y, Lee YH, Kim D, Lim JH, et al. Association of tumor necrosis factor-alpha with fever and pulmonary lesion score in pigs experimentally infected with swine influenza virus subtype H1N2. J Vet Med Sci. 2009;71(5):611-6.

321. Kim WH, Goo SY, Lee KH, Park SJ. Vibrio vulnificus-induced cell death of human mononuclear cells requires ROS-dependent activation of p38 and ERK 1/2 MAPKs. Immunological Investigations. 2009;38(1):31-48.

322. Kumar S, Buza JJ, Burgess SC. Genotype-dependent tumor regression in Marek's disease mediated at the level of tumor immunity. Cancer Microenvironment. 2009;2(1):23-31.

323. Lee YS. Arachidonic acid activates K+-Cl--cotransport in HepG2 human hepatoblastoma cells. Korean Journal of Physiology and Pharmacology. 2009;13(5):401-8.

324. Lee YS. Arachidonic Acid Activates K-Cl-cotransport in HepG2 Human Hepatoblastoma Cells. Korean J Physiol Pharmacol. 2009;13(5):401-8.

325. Leung HY, Yung LH, Poon CH, Shi G, Lu AL, Leung LK. Genistein protects against polycyclic aromatic hydrocarbon-induced oxidative DNA damage in non-cancerous breast cells MCF-10A. Br J Nutr. 2009;101(2):257-62.

326. Leung HY, Yung LH, Shi G, Lu AL, Leung LK. The red wine polyphenol resveratrol reduces polycyclic aromatic hydrocarbon-induced DNA damage in MCF-10A cells. British Journal of Nutrition. 2009;102(10):1462-8.

327. Lin RJ, Cheng MJ, Huang JC, Lo WL, Yeh YT, Yen CM, et al. Cytotoxic compounds from the stems of Cinnamomum tenuifolium. J Nat Prod. 2009;72(10):1816-24.

328. Lin RJ, Cheng MJ, Huang JC, Lo WL, Yeh YT, Yen CM, et al. Cytotoxic compounds from the stems of Cinnamomum tenuifolium. J Nat Prod. 2009;72(10):1816-24.

329. Martín R, Hernández M, Ibeas E, Fuentes L, Salicio V, Arnés M, et al. Secreted phospholipase A2-IIA modulates key regulators of proliferation on astrocytoma cells. J Neurochem. 2009;111(4):988-99.

330. Martín R, Hernández M, Ibeas E, Fuentes L, Salicio V, Arnés M, et al. Secreted phospholipase A2-IIA modulates key regulators of proliferation on astrocytoma cells. J Neurochem. 2009;111(4):988-99.

331. Mishra MK, Ghosh D, Duseja R, Basu A. Antioxidant potential of Minocycline in Japanese Encephalitis Virus infection in murine neuroblastoma cells: Correlation with membrane fluidity and cell death. Neurochemistry International. 2009;54(7):464-70.

332. Moiseeva EP, Manson MM. Dietary chemopreventive phytochemicals: Too little or too much? Cancer Prev Res. 2009;2(7):611-6.

333. Nakamura Y, Yogosawa S, Izutani Y, Watanabe H, Otsuji E, Sakai T. A combination of indol-3-carbinol and genistein synergistically induces apoptosis in human colon cancer HT-29 cells by inhibiting Akt phosphorylation and progression of autophagy. Mol Cancer. 2009;8.

334. Odom RY, Dansby MY, Rollins-Hairston AM, Jackson KM, Kirlin WG. Phytochemical induction of cell cycle arrest by glutathione oxidation and reversal by N-acetylcysteine in human colon carcinoma cells. Nutrition and Cancer. 2009;61(3):332-9.

335. Odom RY, Dansby MY, Rollins-Hairston AM, Jackson KM, Kirlin WG. Phytochemical induction of cell cycle arrest by glutathione oxidation and reversal by N-acetylcysteine in human colon carcinoma cells. Nutr Cancer. 2009;61(3):332-9.

336. Pasciu V, Posadino AM, Cossu A, Sanna B, Tadolini B, Gaspa L, et al. Akt downregulation by flavin oxidase-induced ROS generation mediates dose-dependent endothelial cell damage elicited by natural antioxidants. Toxicol Sci. 2009;114(1):101-12.

337. Rodriguez M, Zoecklein L, Papke L, Gamez J, Denic A, Macura S, et al. Tumor Necrosis Factor α is Reparative via TNFR1 in the Hippocampus and via TNFR2 in the Striatum after Virus-Induced Encephalitis. Brain Pathol. 2009;19(1):12-26.

338. Rodriguez M, Zoecklein L, Papke L, Gamez J, Denic A, Macura S, et al. Tumor necrosis factor alpha is reparative via TNFR2 [corrected] in the hippocampus and via TNFR1 [corrected] in the striatum after virus-induced encephalitis. Brain Pathol. 2009;19(1):12-26.

339. Scofield VL, Yan M, Kuang X, Kim SJ, Crunk D, Wong PKY. The drug monosodium luminol (GVT®) preserves thymic epithelial cell cytoarchitecture and allows thymocyte survival in mice infected with the T cell-tropic, cytopathic retrovirus ts1. Immunol Lett. 2009;122(2):159-69.

340. Scofield VL, Yan M, Kuang X, Kim SJ, Wong PK. The drug monosodium luminol (GVT) preserves crypt-villus epithelial organization and allows survival of intestinal T cells in mice infected with the ts1 retrovirus. Immunol Lett. 2009;122(2):150-8.

341. Scofield VL, Yan M, Kuang X, Kim SJ, Wong PKY. The drug monosodium luminol (GVT) preserves crypt-villus epithelial organization and allows survival of intestinal T cells in mice infected with the ts1 retrovirus. Immunol Lett. 2009;122(2):150-8.

342. Scott EN, Gescher AJ, Steward WP, Brown K. Development of dietary phytochemical chemopreventive agents: Biomarkers and choice of dose for early clinical trials. Cancer Prev Res. 2009;2(6):525-30.

343. Scott EN, Gescher AJ, Steward WP, Brown K. Development of dietary phytochemical chemopreventive agents: biomarkers and choice of dose for early clinical trials. Cancer Prev Res (Phila). 2009;2(6):525-30.

344. Song CH, Honmou O, Ohsawa N, Nakamura K, Hamada H, Furuoka H, et al. Effect of transplantation of bone marrow-derived mesenchymal stem cells on mice infected with prions. J Virol. 2009;83(11):5918-27.

345. Song CH, Honmou O, Ohsawa N, Nakamura K, Hamada H, Furuoka H, et al. Effect of transplantation of bone marrow-derived mesenchymal stem cells on mice infected with prions. J Virol. 2009;83(11):5918-27.

346. Torres-Sanchez L, Galvan-Portillo M, Wolff MS, Lopez-Carrillo L. Dietary consumption of phytochemicals and breast cancer risk in Mexican women. Public Health Nutrition. 2009;12(6):825-31.

347. Tosetti F, Noonan DM, Albini A. Metabolic regulation and redox activity as mechanisms for angioprevention by dietary phytochemicals. Int J Cancer. 2009;125(9):1997-2003.

348. Wipf P, Arnold D, Carter K, Dong S, Johnston PA, Sharlow E, et al. A case study from the chemistry core of the Pittsburgh Molecular Library Screening Center: the Polo-like kinase polo-box domain (Plk1-PBD). Curr Top Med Chem. 2009;9(13):1194-205.

349. Wipf P, Arnold D, Carter K, Dong S, Johnston PA, Sharlow E, et al. A case study from the chemistry core of the Pittsburgh molecular library screening center: The polo-like kinase polo-box domain (Plk1-PBD). Curr Top Med Chem. 2009;9(13):1194-205.

350. Xavier CP, Lima CF, Preto A, Seruca R, Fernandes-Ferreira M, Pereira-Wilson C. Luteolin, quercetin and ursolic acid are potent inhibitors of proliferation and inducers of apoptosis in both KRAS and BRAF mutated human colorectal cancer cells. Cancer Lett. 2009;281(2):162-70.

351. Adams LS, Phung S, Yee N, Seeram NP, Li L, Chen S. Blueberry phytochemicals inhibit growth and metastatic potential of MDA-MB-231 breast cancer cells through modulation of the phosphatidylinositol 3-kinase pathway. Cancer Res. 2010;70(9):3594-605.

352. Ali K, Reichert T, Gomez D, Lu Y, Jan A, Christensen C. A new approach to concordance in mid-infrared spectromicroscopy mapping of malignant tumors. Oncol Rep. 2010;24(4):857-60.

353. Ali K, Reichert T, Gomez D, Lu YJ, Jan A, Christensen C. A new approach to concordance in mid-infrared spectromicroscopy mapping of malignant tumors. Oncol Rep. 2010;24(4):857-60.

354. Chiu WT, Shen SC, Chow JM, Lin CW, Shia LT, Chen YC. Contribution of reactive oxygen species to migration/invasion of human glioblastoma cells U87 via ERK-dependent COX-2/PGE(2) activation. Neurobiol Dis. 2010;37(1):118-29.

355. de Kok TMCM, de Waard P, Wilms LC, van Breda SGJ. Antioxidative and antigenotoxic properties of vegetables and dietary phytochemicals: The value of genomics biomarkers in molecular epidemiology. Molecular Nutrition and Food Research. 2010;54(2):208-17.

356. Fong D, Yeh A, Naftalovich R, Choi TH, Chan MM. Curcumin inhibits the side population (SP) phenotype of the rat C6 glioma cell line: towards targeting of cancer stem cells with phytochemicals. Cancer Lett. 2010;293(1):65-72.

357. Fong D, Yeh A, Naftalovich R, Choi TH, Chan MM. Curcumin inhibits the side population (SP) phenotype of the rat C6 glioma cell line: Towards targeting of cancer stem cells with phytochemicals. Cancer Lett. 2010;293(1):65-72.

358. Gagliano N, Aldini G, Colombo G, Rossi R, Colombo R, Gioia M, et al. The potential of resveratrol against human gliomas. Anticancer Drugs. 2010;21(2):140-50.

359. Gómez-Laguna J, Salguero FJ, Pallarés FJ, de Marco MF, Barranco I, Cerón JJ, et al. Acute phase response in porcine reproductive and respiratory syndrome virus infection. Comp Immunol Microbiol Infect Dis. 2010;33(6):E51-E8.

360. Gómez-Laguna J, Salguero FJ, Pallarés FJ, Fernández de Marco M, Barranco I, Cerón JJ, et al. Acute phase response in porcine reproductive and respiratory syndrome virus infection. Comparative Immunology, Microbiology and Infectious Diseases. 2010;33(6):e51-e8.

361. Guo HJ, Li HM, Cheng ZQ, Liu JZ, Cui ZZ. Influence of REV and ALV-J co-infection on immunologic function of T lymphocytes and histopathology in broiler chickens. Agric Sci China. 2010;9(11):1667-76.

362. Heng MC. Curcumin targeted signaling pathways: basis for anti-photoaging and anti-carcinogenic therapy. Int J Dermatol. 2010;49(6):608-22.

363. Horst RJ, Doehlemann G, Wahl R, Hofmann J, Schmiedl A, Kahmann R, et al. Ustilago maydis infection strongly alters organic nitrogen allocation in maize and stimulates productivity of systemic source leaves. Plant Physiol. 2010;152(1):293-308.

364. Kaminski BM, Loitsch SM, Ochs MJ, Reuter KC, Steinhilber D, Stein J, et al. Isothiocyanate sulforaphane inhibits protooncogenic ornithine decarboxylase activity in colorectal cancer cells via induction of the TGF-β/Smad signaling pathway. Mol Nutr Food Res. 2010;54(10):1486-96.

365. Kaminski BM, Loitsch SM, Ochs MJ, Reuter KC, Steinhilber D, Stein J, et al. Isothiocyanate sulforaphane inhibits protooncogenic ornithine decarboxylase activity in colorectal cancer cells <i>via</i> induction of the TGF-β/Smad signaling pathway. Mol Nutr Food Res. 2010;54(10):1486-96.

366. Lee JY, Song JJ, Wooming A, Li X, Zhou H, Bottje WG, et al. Transcriptional profiling of host gene expression in chicken embryo lung cells infected with laryngotracheitis virus. BMC Genomics. 2010;11(1).

367. Lee JY, Song JJ, Wooming A, Li XY, Zhou HJ, Bottje WG, et al. Transcriptional profiling of host gene expression in chicken embryo lung cells infected with laryngotracheitis virus. BMC Genomics. 2010;11:15.

368. Lee KM, Lee KW, Byun S, Jung SK, Seo SK, Heo YS, et al. 5-Deoxykaempferol plays a potential therapeutic role by targeting multiple signaling pathways in skin cancer. Cancer Prev Res. 2010;3(4):454-65.

369. Li Y, Revalde JL, Reid G, Paxton JW. Interactions of dietary phytochemicals with ABC transporters: Possible implications for drug disposition and multidrug resistance in cancer. Drug Metabolism Reviews. 2010;42(4):590-611.

370. Liao Y, Du X, Lönnerdal B. miR-214 regulates lactoferrin expression and pro-apoptotic function in mammary epithelial cells. J Nutr. 2010;140(9):1552-6.

371. Mesquita FS, Dyer SN, Heinrich DA, Bulun SE, Marsh EE, Nowak RA. Reactive oxygen species mediate mitogenic growth factor signaling pathways in human leiomyoma smooth muscle cells. Biol Reprod. 2010;82(2):341-51.

372. Mesquita FS, Dyer SN, Heinrich DA, Bulun SE, Marsh EE, Nowak RA. Reactive Oxygen Species Mediate Mitogenic Growth Factor Signaling Pathways in Human Leiomyoma Smooth Muscle Cells. Biol Reprod. 2010;82(2):341-51.

373. Morales HD, Abramowitz L, Gertz J, Sowa J, Vogel A, Robert J. Innate immune responses and permissiveness to ranavirus infection of peritoneal leukocytes in the frog Xenopus laevis. J Virol. 2010;84(10):4912-22.

374. Morales HD, Abramowitz L, Gertz J, Sowa J, Vogel A, Robert J. Innate immune responses and permissiveness to ranavirus infection of peritoneal leukocytes in the frog Xenopus laevis. J Virol. 2010;84(10):4912-22.

375. Nair S, Barve A, Khor TO, Shen GX, Lin W, Chan JY, et al. Regulation of Nrf2-and AP-1-mediated gene expression by epigallocatechin-3-gallate and sulforaphane in prostate of Nrf2-knockout or C57BL/6J mice and PC-3 AP-1 human prostate cancer cells. Acta Pharmacol Sin. 2010;31(9):1223-40.

376. Nishanth RP, Ramakrishna BS, Jyotsna RG, Roy KR, Reddy GV, Reddy PK, et al. C-Phycocyanin inhibits MDR1 through reactive oxygen species and cyclooxygenase-2 mediated pathways in human hepatocellular carcinoma cell line. Eur J Pharmacol. 2010;649(1-3):74-83.

377. Nishanth RP, Ramakrishna BS, Jyotsna RG, Roy KR, Reddy GV, Reddy PK, et al. C-Phycocyanin inhibits MDR1 through reactive oxygen species and cyclooxygenase-2 mediated pathways in human hepatocellular carcinoma cell line. Eur J Pharmacol. 2010;649(1-3):74-83.

378. Nitti M, Furfaro AL, Cevasco C, Traverso N, Marinari UM, Pronzato MA, et al. PKC delta and NADPH oxidase in retinoic acid-induced neuroblastoma cell differentiation. Cell Signal. 2010;22(5):828-35.

379. Paek AR, Kim SH, Kim SS, Kim KT, You HJ. IGF-1 induces expression of zinc-finger protein 143 in colon cancer cells through phosphatidylinositide 3-kinase and reactive oxygen species. Exp Mol Med. 2010;42(10):696-702.

380. Paluszczak J, Krajka-Kuźniak V, Baer-Dubowska W. The effect of dietary polyphenols on the epigenetic regulation of gene expression in MCF7 breast cancer cells. Toxicol Lett. 2010;192(2):119-25.

381. Saunders JA, Rogers LC, Klomsiri C, Poole LB, Daniel LW. Reactive oxygen species mediate lysophosphatidic acid induced signaling in ovarian cancer cells. Free Radic Biol Med. 2010;49(12):2058-67.

382. Seymour EM, Bennink MR, Watts SW, Bolling SF. Whole grape intake impacts cardiac peroxisome proliferator-activated receptor and nuclear factor κb activity and cytokine expression in rats with diastolic dysfunction. Hypertension. 2010;55(5):1179-85.

383. Shinohara M, Adachi Y, Mitsushita J, Kuwabara M, Nagasawa A, Harada S, et al. Reactive oxygen generated by NADPH oxidase 1 (Nox1) contributes to cell invasion by regulating matrix metalloprotease-9 production and cell migration. J Biol Chem. 2010;285(7):4481-8.

384. Shinohara M, Adachi Y, Mitsushita J, Kuwabara M, Nagasawa A, Harada S, et al. Reactive oxygen generated by NADPH oxidase 1 (Nox1) contributes to cell invasion by regulating matrix metalloprotease-9 production and cell migration. J Biol Chem. 2010;285(7):4481-8.

385. Subramaniam D, Ramalingam S, Houchen CW, Anant S. Cancer stem cells: a novel paradigm for cancer prevention and treatment. Mini reviews in medicinal chemistry. 2010;10(5):359-71.

386. Tan XL, Shi M, Tang H, Han W, Spivack SD. Candidate dietary phytochemicals modulate expression of phase II enzymes GSTP1 and NQO1 in human lung cells. J Nutr. 2010;140(8):1404-10.

387. Tobar N, Villar V, Santibanez JF. ROS-NFκΒ mediates TGF-β1-induced expression of urokinase-type plasminogen activator, matrix metalloproteinase-9 and cell invasion. Mol Cell Biochem. 2010;340(1-2):195-202.

388. Tobar N, Villar V, Santibanez JF. ROS-NFκI' mediates TGF-β1-induced expression of urokinase-type plasminogen activator, matrix metalloproteinase-9 and cell invasion. Mol Cell Biochem. 2010;340(1-2):195-202.

389. Vanden Berghe W, Haegeman G. Epigenetic remedies by dietary phytochemicals against inflammatory skin disorders: Myth or reality? Curr Drug Metab. 2010;11(5):436-50.

390. Vidya Priyadarsini R, Senthil Murugan R, Maitreyi S, Ramalingam K, Karunagaran D, Nagini S. The flavonoid quercetin induces cell cycle arrest and mitochondria-mediated apoptosis in human cervical cancer (HeLa) cells through p53 induction and NF-κB inhibition. Eur J Pharmacol. 2010;649(1-3):84-91.

391. Vilela MC, Lima GK, Rodrigues DH, Lacerda-Queiroz N, Mansur DS, de Miranda AS, et al. TNFR1 plays a critical role in the control of severe HSV-1 encephalitis. Neurosci Lett. 2010;479(1):58-62.

392. Vilela MC, Lima GK, Rodrigues DH, Lacerda-Queiroz N, Mansur DS, de Miranda AS, et al. TNFR1 plays a critical role in the control of severe HSV-1 encephalitis. Neurosci Lett. 2010;479(1):58-62.

393. Wang H, Guo J, Zhao Y, Bian GL, Liu FF, Yu CY, et al. Recovery of movement after spinal cord injury in DO11.10 transgenic mouse. Xi bao yu fen zi mian yi xue za zhi = Chinese journal of cellular and molecular immunology. 2010;26(3):231-4.

394. Yang SJ, Chang SC, Wen HC, Chen CY, Liao JF, Chang CH. Plumbagin activates ERK1/2 and Akt via superoxide, Src and PI3-kinase in 3T3-L1 Cells. Eur J Pharmacol. 2010;638(1-3):21-8.

395. Yang SJ, Chang SC, Wen HC, Chen CY, Liao JF, Chang CH. Plumbagin activates ERK1/2 and Akt via superoxide, Src and PI3-kinase in 3T3-L1 cells. Eur J Pharmacol. 2010;638(1-3):21-8.

396. Yoon S, Woo SU, Kang JH, Kim K, Kwon MH, Park S, et al. STAT3 transcriptional factor activated by reactive oxygen species induces IL6 in starvation-induced autophagy of cancer cells. Autophagy. 2010;6(8):1125-38.

397. Yoon S, Woo SU, Kang JH, Kim K, Kwon MH, Park S, et al. STAT3 transcriptional factor activated by reactive oxygen species induces IL6 in starvation-induced autophagy of cancer cells. Autophagy. 2010;6(8):1125-38.

398. Yu XD, Guo ZS. Epigenetic drugs for cancer treatment and prevention: Mechanisms of action. Biomolecular Concepts. 2010;1(3-4):239-51.

399. Ahmad R, Sylvester J, Ahmad M, Zafarullah M. Involvement of H-Ras and reactive oxygen species in proinflammatory cytokine-induced matrix metalloproteinase-13 expression in human articular chondrocytes. Arch Biochem Biophys. 2011;507(2):350-5.

400. Ahmad R, Sylvester J, Ahmad M, Zafarullah M. Involvement of H-Ras and reactive oxygen species in proinflammatory cytokine-induced matrix metalloproteinase-13 expression in human articular chondrocytes. Arch Biochem Biophys. 2011;507(2):350-5.

401. Alshatwi AA, Shafi G, Hasan TN, Al-Hazzani AA, Alsaif MA, Alfawaz MA, et al. Apoptosis-mediated inhibition of human breast cancer cell proliferation by lemon citrus extract. Asian Pac J Cancer Prev. 2011;12(6):1555-9.

402. Chang SP, Shen SC, Lee WR, Yang LL, Chen YC. Imatinib mesylate induction of ROS-dependent apoptosis in melanoma B16F0 cells. J Dermatol Sci. 2011;62(3):183-91.

403. Chang SP, Shen SC, Lee WR, Yang LL, Chen YC. Imatinib mesylate induction of ROS-dependent apoptosis in melanoma B16F0 cells. J Dermatol Sci. 2011;62(3):183-91.

404. Chatterjee M, Das S, Janarthan M, Ramachandran HK, Chatterjee M. Role of 5-lipoxygenase in resveratrol mediated suppression of 7,12-dimethylbenz(α)anthracene-induced mammary carcinogenesis in rats. Eur J Pharmacol. 2011;668(1-2):99-106.

405. Chatterjee M, Das S, Janarthan M, Ramachandran HK, Chatterjee M. Role of 5-lipoxygenase in resveratrol mediated suppression of 7,12-dimethylbenz(α)anthracene-induced mammary carcinogenesis in rats. Eur J Pharmacol. 2011;668(1-2):99-106.

406. Chavez-Perez VA, Strasberg-Rieber M, Rieber M. Metabolic utilization of exogenous pyruvate by mutant p53 (R175H) human melanoma cells promotes survival under glucose depletion. Cancer Biol Ther. 2011;12(7):647-56.

407. Chavez-Perez VA, Strasberg-Rieber M, Rieber M. Metabolic utilization of exogenous pyruvate by mutant p53 (R175H) human melanoma cells promotes survival under glucose depletion. Cancer Biol Ther. 2011;12(7):647-56.

408. Chung YW, Kim HK, Kim IY, Yim MB, Chock PB. Dual function of protein kinase C (PKC) in 12-O-tetradecanoylphorbol-13- acetate (TPA)-induced manganese superoxide dismutase (MnSOD) expression: Activation of creb and foxo3a by PKC-α phosphorylation and by PKC-mediated inactivation of akt, respectively. J Biol Chem. 2011;286(34):29681-90.

409. Chung YW, Kim HK, Kim IY, Yim MB, Chock PB. Dual function of protein kinase C (PKC) in 12-O-tetradecanoylphorbol-13-acetate (TPA)-induced manganese superoxide dismutase (MnSOD) expression: activation of CREB and FOXO3a by PKC-alpha phosphorylation and by PKC-mediated inactivation of Akt, respectively. J Biol Chem. 2011;286(34):29681-90.

410. Fang KM, Wang YL, Huang MC, Sun SH, Cheng H, Tzeng SF. Expression of macrophage inflammatory protein-1α and monocyte chemoattractant protein-1 in glioma-infiltrating microglia: involvement of ATP and P2X₇ receptor. J Neurosci Res. 2011;89(2):199-211.

411. Gao X, Deeb D, Liu P, Liu Y, Arbab-Ali S, Dulchavsky SA, et al. Role of reactive oxygen species (ROS) in CDDO-Me-mediated growth inhibition and apoptosis in colorectal cancer cells. Journal of Experimental Therapeutics and Oncology. 2011;9(2):119-27.

412. Ho E, Beaver LM, Williams DE, Dashwood RH. Dietary factors and epigenetic regulation for prostate cancer prevention. Advances in Nutrition. 2011;2(6):497-510.

413. Hsum YW, Yew WT, Hong PL, Soo KK, Hoon LS, Chieng YC, et al. Cancer chemopreventive activity of maslinic acid: suppression of COX-2 expression and inhibition of NF-κB and AP-1 activation in Raji cells. Planta Med. 2011;77(2):152-7.

414. Jeong WK, Byun JH, Lee SS, Won HJ, Kim KW, Shin YM, et al. Gadobenate dimeglumine-enhanced liver MR imaging in cirrhotic patients: Quantitative and qualitative comparison of 1-hour and 3-hour delayed images. J Magn Reson Imaging. 2011;33(4):889-97.

415. Jeong WK, Byun JH, Lee SS, Won HJ, Kim KW, Shin YM, et al. Gadobenate dimeglumine-enhanced liver MR imaging in cirrhotic patients: quantitative and qualitative comparison of 1-hour and 3-hour delayed images. J Magn Reson Imaging. 2011;33(4):889-97.

416. Jiang Z, Fletcher NM, Ali-Fehmi R, Diamond MP, Abu-Soud HM, Munkarah AR, et al. Modulation of redox signaling promotes apoptosis in epithelial ovarian cancer cells. Gynecol Oncol. 2011;122(2):418-23.

417. Jiang Z, Fletcher NM, Ali-Fehmi R, Diamond MP, Abu-Soud HM, Munkarah AR, et al. Modulation of redox signaling promotes apoptosis in epithelial ovarian cancer cells. Gynecol Oncol. 2011;122(2):418-23.

418. Jiménez-Aliaga K, Bermejo-Bescós P, Benedí J, Martín-Aragón S. Quercetin and rutin exhibit antiamyloidogenic and fibril-disaggregating effects in vitro and potent antioxidant activity in APPswe cells. Life Sci. 2011;89(25-26):939-45.

419. Katiyar SK. Green tea prevents non-melanoma skin cancer by enhancing DNA repair. Arch Biochem Biophys. 2011;508(2):152-8.

420. Kim KA, Kim JY, Lee YA, Song KJ, Min D, Shin MH. NOX1 participates in ROS-dependent cell death of colon epithelial Caco2 cells induced by Entamoeba histolytica. Microbes Infect. 2011;13(12-13):1052-61.

421. Kim KA, Kim JY, Lee YA, Song KJ, Min D, Shin MH. NOX1 participates in ROS-dependent cell death of colon epithelial Caco2 cells induced by <i>Entamoeba histolytica</i>. Microbes Infect. 2011;13(12-13):1052-61.

422. Lee CW, Lin CC, Lee IT, Lee HC, Yang CM. Activation and induction of cytosolic phospholipase A2 by TNF-α mediated through Nox2, MAPKs, NF-κB, and p300 in human tracheal smooth muscle cells. J Cell Physiol. 2011;226(8):2103-14.

423. Lee CW, Lin CC, Lee IT, Lee HC, Yang CM. Activation and induction of cytosolic phospholipase A2 by TNF-α mediated through Nox2, MAPKs, NF-κB, and p300 in human tracheal smooth muscle cells. J Cell Physiol. 2011;226(8):2103-14.

424. Lee KW, Bode AM, Dong Z. Molecular targets of phytochemicals for cancer prevention. Nature Reviews Cancer. 2011;11(3):211-8.

425. Lee M, Suk K, Kang Y, McGeer E, McGeer PL. Neurotoxic factors released by stimulated human monocytes and THP-1 cells. Brain Res. 2011;1400:99-111.

426. Lee PK, Law WH, Liu HW, Lo KK. Luminescent cyclometalated iridium(III) polypyridine di-2-picolylamine complexes: synthesis, photophysics, electrochemistry, cation binding, cellular internalization, and cytotoxic activity. Inorg Chem. 2011;50(17):8570-9.

427. Lee TKW, Castilho A, Cheung VCH, Tang KH, Ma S, Ng IOL. Lupeol targets liver tumor-initiating cells through phosphatase and tensin homolog modulation. Hepatology. 2011;53(1):160-70.

428. Moody TW, Sancho V, Di Florio A, Nuche-Berenguer B, Mantey S, Jensen RT. Bombesin receptor subtype-3 agonists stimulate the growth of lung cancer cells and increase EGF receptor tyrosine phosphorylation. Peptides. 2011;32(8):1677-84.

429. Moody TW, Sancho V, di Florio A, Nuche-Berenguer B, Mantey S, Jensen RT. Bombesin receptor subtype-3 agonists stimulate the growth of lung cancer cells and increase EGF receptor tyrosine phosphorylation. Peptides. 2011;32(8):1677-84.

430. Muregi FW, Ohta I, Masato U, Kino H, Ishih A. Resistance of a rodent malaria parasite to a thymidylate synthase inhibitor induces an apoptotic parasite death and imposes a huge cost of fitness. PLoS One. 2011;6(6).

431. Muregi FW, Ohta I, Masato U, Kino H, Ishih A. Resistance of a rodent malaria parasite to a thymidylate synthase inhibitor induces an apoptotic parasite death and imposes a huge cost of fitness. PLoS One. 2011;6(6):e21251.

432. Nandakumar V, Vaid M, Katiyar SK. (-)-Epigallocatechin-3-gallate reactivates silenced tumor suppressor genes, Cip1/p21 and p16INK4a, by reducing DNA methylation and increasing histones acetylation in human skin cancer cells. Carcinogenesis. 2011;32(4):537-44.

433. Okamoto H, Shiraki K, Yasuda R, Danjo K, Watanabe Y. Chitosan-interferon-β gene complex powder for inhalation treatment of lung metastasis in mice. J Control Release. 2011;150(2):187-95.

434. Pazhanisamy SK, Li H, Wang Y, Batinic-Haberle I, Zhou D. NADPH oxidase inhibition attenuates total body irradiation-induced haematopoietic genomic instability. Mutagenesis. 2011;26(3):431-5.

435. Priyadarsini RV, Vinothini G, Murugan RS, Manikandan P, Nagini S. The flavonoid quercetin modulates the hallmark capabilities of hamster buccal pouch tumors. Nutrition and Cancer. 2011;63(2):218-26.

436. Priyadarsini RV, Vinothini G, Murugan RS, Manikandan P, Nagini S. The flavonoid quercetin modulates the hallmark capabilities of hamster buccal pouch tumors. Nutr Cancer. 2011;63(2):218-26.

437. Qian J, Keyes KT, Long B, Chen G, Ye Y. Impact of HMG-CoA reductase inhibition on oxidant-induced injury in human retinal pigment epithelium cells. J Cell Biochem. 2011;112(9):2480-9.

438. Queisser N, Schupp N, Stopper H, Schinzel R, Oteiza PI. Aldosterone increases kidney tubule cell oxidants through calcium-mediated activation of NADPH oxidase and nitric oxide synthase. Free Radic Biol Med. 2011;51(11):1996-2006.

439. Queisser N, Schupp N, Stopper H, Schinzel R, Oteiza PI. Aldosterone increases kidney tubule cell oxidants through calcium-mediated activation of NADPH oxidase and nitric oxide synthase. Free Radic Biol Med. 2011;51(11):1996-2006.

440. Rezaei PF, Fouladdel S, Cristofanon S, Ghaffari SM, Amin GR, Azizi E. Comparative cellular and molecular analysis of cytotoxicity and apoptosis induction by doxorubicin and Baneh in human breast cancer T47D cells. Cytotechnology. 2011;63(5):503-12.

441. Ricciardiello L, Bazzoli F, Fogliano V. Phytochemicals and colorectal cancer prevention-myth or reality? Nature Reviews Gastroenterology and Hepatology. 2011;8(10):592-6.

442. Saw CLL, Cintrón M, Wu TY, Guo Y, Huang Y, Jeong WS, et al. Pharmacodynamics of dietary phytochemical indoles I3C and DIM: Induction of Nrf2-mediated phase II drug metabolizing and antioxidant genes and synergism with isothiocyanates. Biopharmaceutics and Drug Disposition. 2011;32(5):289-300.

443. Saw CLL, Cintrón M, Wu TY, Guo Y, Huang Y, Jeong WS, et al. Pharmacodynamics of dietary phytochemical indoles I3C and DIM: Induction of Nrf2-mediated phase II drug metabolizing and antioxidant genes and synergism with isothiocyanates. Biopharm Drug Dispos. 2011;32(5):289-300.

444. Singh T, Sharma SD, Katiyar SK. Grape proanthocyanidins induce apoptosis by loss of mitochondrial membrane potential of human non-small cell lung cancer cells In Vitro and In Vivo. PLoS One. 2011;6(11).

445. Stoolman JS, Vannella KM, Coomes SM, Wilke CA, Sisson TH, Toews GB, et al. Latent infection by γherpesvirus stimulates profibrotic mediator release from multiple cell types. American Journal of Physiology - Lung Cellular and Molecular Physiology. 2011;300(2):L274-L85.

446. Sukumari-Ramesh S, Bentley JN, Laird MD, Singh N, Vender JR, Dhandapani KM. Dietary phytochemicals induce p53- and caspase-independent cell death in human neuroblastoma cells. Int J Dev Neurosci. 2011;29(7):701-10.

447. Tan AC, Konczak I, Sze DM, Ramzan I. Molecular pathways for cancer chemoprevention by dietary phytochemicals. Nutr Cancer. 2011;63(4):495-505.

448. Thoppil RJ, Bishayee A. Terpenoids as potential chemopreventive and therapeutic agents in liver cancer. World Journal of Hepatology. 2011;3(9):228-49.

449. Wauthoz N, Deleuze P, Saumet A, Duret C, Kiss R, Amighi K. Temozolomide-Based Dry Powder Formulations for Lung Tumor-Related Inhalation Treatment. Pharm Res. 2011;28(4):762-75.

450. Xu M, Zhang H, Lee L, Gao H, Sharif S, Silva RF, et al. Gene expression profiling in rMd5- and rMd5Δmeq-infected chickens. Avian Dis. 2011;55(3):358-67.

451. Xu M, Zhang H, Lee L, Gao H, Sharif S, Silva RF, et al. Gene expression profiling in rMd5- and rMd5deltameq-infected chickens. Avian Dis. 2011;55(3):358-67.

452. Adam V, Ekblad M, Sweeney K, Müller H, Busch KH, Johnsen CT, et al. Synergistic and selective cancer cell killing mediated by the oncolytic adenoviral mutant adδδ and dietary phytochemicals in prostate cancer models. Human Gene Therapy. 2012;23(9):1003-15.

453. Anete Lallo M, Porta Miche Hirschfeld M. Encephalitozoonosis in pharmacologically immunosuppressed mice. Exp Parasitol. 2012;131(3):339-43.

454. Avunje S, Kim WS, Oh MJ, Choi I, Jung SJ. Temperature-dependent viral replication and antiviral apoptotic response in viral haemorrhagic septicaemia virus (VHSV)-infected olive flounder (<i>Paralichthys olivaceus</i>). Fish Shellfish Immunol. 2012;32(6):1162-70.

455. Bainer RO, Veneris JT, Yamada SD, Montag A, Lingen MW, Gilad Y, et al. Time-dependent transcriptional profiling links gene expression to mitogen-activated protein kinase kinase 4 (MKK4)-mediated suppression of omental metastatic colonization. Clin Exp Metastasis. 2012;29(5):397-408.

456. Bainer RO, Veneris JT, Yamada SD, Montag A, Lingen MW, Gilad Y, et al. Time-dependent transcriptional profiling links gene expression to mitogen-activated protein kinase kinase 4 (MKK4)-mediated suppression of omental metastatic colonization. Clin Exp Metastasis. 2012;29(5):397-408.

457. Bartoncini S, Fiandra C, Redda MGR, Allis S, Munoz F, Ricardi U. Target registration errors with surface imaging system in conformal radiotherapy for prostate cancer: study on 19 patients. Radiol Med. 2012;117(8):1419-28.

458. Bartoncini S, Fiandra C, Ruo Redda MG, Allis S, Munoz F, Ricardi U. Target registration errors with surface imaging system in conformal radiotherapy for prostate cancer: Study on 19 patients. Radiol Med. 2012;117(8):1419-28.

459. Bishayee A, Thoppil RJ, Waghray A, Kruse JA, Novotny NA, Darvesh AS. Dietary phytochemicals in the chemoprevention and treatment of hepatocellular carcinoma: in vivo evidence, molecular targets, and clinical relevance. Curr Cancer Drug Targets. 2012;12(9):1191-232.

460. Canese R, Pisanu ME, Mezzanzanica D, Ricci A, Paris L, Bagnoli M, et al. Characterisation of in vivo ovarian cancer models by quantitative 1H magnetic resonance spectroscopy and diffusion-weighted imaging. NMR Biomed. 2012;25(4):632-42.

461. Canese R, Pisanu ME, Mezzanzanica D, Ricci A, Paris L, Bagnoli M, et al. Characterisation of in vivo ovarian cancer models by quantitative 1H magnetic resonance spectroscopy and diffusion-weighted imaging. NMR Biomed. 2012;25(4):632-42.

462. Cavell BE, Alwi SSS, Donleyy AM, Proud CG, Packham G. Natural Product-Derived Antitumor Compound Phenethyl Isothiocyanate Inhibits mTORC1 Activity via TSC2. J Nat Prod. 2012;75(6):1051-7.

463. Cavell BE, Syed Alwi SS, Donlevy AM, Proud CG, Packham G. Natural product-derived antitumor compound phenethyl isothiocyanate inhibits mTORC1 activity via TSC2. J Nat Prod. 2012;75(6):1051-7.

464. Che TM, Song M, Liu Y, Johnson RW, Kelley KW, Van Alstine WG, et al. Mannan oligosaccharide increases serum concentrations of antibodies and inflammatory mediators in weanling pigs experimentally infected with porcine reproductive and respiratory syndrome virus. J Anim Sci. 2012;90(8):2784-93.

465. Che TM, Song M, Liu Y, Johnson RW, Kelley KW, Van Alstine WG, et al. Mannan oligosaccharide increases serum concentrations of antibodies and inflammatory mediators in weanling pigs experimentally infected with porcine reproductive and respiratory syndrome virus. J Anim Sci. 2012;90(8):2784-93.

466. Chen CC, Cheng YY, Chen SC, Tuan YF, Chen YJ, Chen CY, et al. Cyclooxygenase-2 expression is up-regulated by 2-aminobiphenyl in a ROS and MAPK-dependent signaling pathway in a bladder cancer cell line. Chem Res Toxicol. 2012;25(3):695-705.

467. Chen CC, Cheng YY, Chen SC, Tuan YF, Chen YJ, Chen CY, et al. Cyclooxygenase-2 Expression Is Up-regulated by 2-Aminobiphenyl in a ROS and MAPK-Dependent Signaling Pathway in a Bladder Cancer Cell Line. Chem Res Toxicol. 2012;25(3):695-705.

468. Chin DH, Li HH, Kuo HM, Chao PDL, Liu CW. Neocarzinostatin as a probe for DNA protection activity-molecular interaction with caffeine. Molecular Carcinogenesis. 2012;51(4):327-38.

469. Deeb D, Gao X, Liu YB, Gautam SC. Inhibition of cell proliferation and induction of apoptosis by CDDO-Me in pancreatic cancer cells is ROS-dependent. J Exp Ther Oncol. 2012;10(1):51-64.

470. Donini M, Marongiu L, Fontana E, Dusi S. Prostate carcinoma cells LNCaP and glucan cooperate in induction of cytokine synthesis by dendritic cells: effect on natural killer cells and CD4+ lymphocytes activation. Prostate. 2012;72(5):566-76.

471. Doroshow JH, Juhasz A, Ge Y, Holbeck S, Lu J, Antony S, et al. Antiproliferative mechanisms of action of the flavin dehydrogenase inhibitors diphenylene iodonium and di-2-thienyliodonium based on molecular profiling of the NCI-60 human tumor cell panel. Biochem Pharmacol. 2012;83(9):1195-207.

472. Doroshow JH, Juhasz A, Ge Y, Holbeck S, Lu JM, Antony S, et al. Antiproliferative mechanisms of action of the flavin dehydrogenase inhibitors diphenylene iodonium and di-2-thienyliodonium based on molecular profiling of the NCI-60 human tumor cell panel. Biochem Pharmacol. 2012;83(9):1195-207.

473. Gordon MH. Significance of dietary antioxidants for health. Int J Mol Sci. 2012;13(1):184-99.

474. Gurley BJ. Pharmacokinetic herb-drug interactions (part 1): Origins, mechanisms, and the impact of botanical dietary supplements. Planta Medica. 2012;78(13):1478-89.

475. Hörmann V, Kumi-Diaka J, Durity M, Rathinavelu A. Anticancer activities of genistein-topotecan combination in prostate cancer cells. J Cell Mol Med. 2012;16(11):2631-6.

476. Hörmann V, Kumi-Diaka J, Durity M, Rathinavelu A. Anticancer activities of genistein-topotecan combination in prostate cancer cells. J Cell Mol Med. 2012;16(11):2631-6.

477. Im YS, Ryu YK, Moon EY. Mouse melanoma cell migration is dependent on production of reactive oxygen species under normoxia condition. Biomolecules and Therapeutics. 2012;20(2):165-70.

478. Im YS, Ryu YK, Moon EY. Mouse Melanoma Cell Migration is Dependent on Production of Reactive Oxygen Species under Normoxia Condition. Biomol Ther (Seoul). 2012;20(2):165-70.

479. Karumbaiah L, Norman SE, Rajan NB, Anand S, Saxena T, Betancur M, et al. The upregulation of specific interleukin (IL) receptor antagonists and paradoxical enhancement of neuronal apoptosis due to electrode induced strain and brain micromotion. Biomaterials. 2012;33(26):5983-96.

480. Karumbaiah L, Norman SE, Rajan NB, Anand S, Saxena T, Betancur M, et al. The upregulation of specific interleukin (IL) receptor antagonists and paradoxical enhancement of neuronal apoptosis due to electrode induced strain and brain micromotion. Biomaterials. 2012;33(26):5983-96.

481. Liu GS, Peshavariya H, Higuchi M, Brewer AC, Chang CWT, Chan EC, et al. Microphthalmia-associated transcription factor modulates expression of NADPH oxidase type 4: A negative regulator of melanogenesis. Free Radic Biol Med. 2012;52(9):1835-43.

482. MacDonald RS, Wagner K. Influence of dietary phytochemicals and microbiota on colon cancer risk. J Agric Food Chem. 2012;60(27):6728-35.

483. Macrì A, Saladino E, Caminiti R, Bartolo V, Fortugno A, Asero S, et al. Doppler perfusion index and colorectal hepatic metastases: personal experience and review of the literature. Hepatogastroenterology. 2012;59(115):731-3.

484. Macrì A, Saladino E, Caminiti R, Bartolo V, Fortugno A, Asero S, et al. Doppler perfusion index and colorectal hepatic metastases: Personal experience and review of the literature. Hepato-Gastroenterol. 2012;59(115):731-3.

485. Meyer MD, Hausbeck MK. Using cultural practices and cultivar resistance to manage Phytophthora crown rot on summer squash. HortScience. 2012;47(8):1080-4.

486. Mooi LY, Yew WT, Hsum YW, Soo KK, Hoon LS, Chieng YC. Suppressive effect of maslinic acid on PMA-induced protein kinase C in human B-lymphoblastoid cells. Asian Pac J Cancer Prev. 2012;13(4):1177-82.

487. Morgan MA, Onono FO, Spielmann HP, Subramanian T, Scherr M, Venturini L, et al. Modulation of anthracycline-induced cytotoxicity by targeting the prenylated proteome in myeloid leukemia cells. J Mol Med (Berl). 2012;90(2):149-61.

488. Morgan MA, Onono FO, Spielmann HP, Subramanian T, Scherr M, Venturini L, et al. Modulation of anthracycline-induced cytotoxicity by targeting the prenylated proteome in myeloid leukemia cells. Journal of Molecular Medicine. 2012;90(2):149-61.

489. Nguyen HM, Reyland ME, Barlow LA. Mechanisms of taste bud cell loss after head and neck irradiation. J Neurosci. 2012;32(10):3474-84.

490. Nguyen HM, Reyland ME, Barlow LA. Mechanisms of taste bud cell loss after head and neck irradiation. J Neurosci. 2012;32(10):3474-84.

491. O'Leary DP, Bhatt L, Woolley JF, Gough DR, Wang JH, Cotter TG, et al. TLR-4 Signalling Accelerates Colon Cancer Cell Adhesion via NF-κB Mediated Transcriptional Up-Regulation of Nox-1. PLoS One. 2012;7(10).

492. O'Leary DP, Bhatt L, Woolley JF, Gough DR, Wang JH, Cotter TG, et al. TLR-4 signalling accelerates colon cancer cell adhesion via NF-κB mediated transcriptional up-regulation of Nox-1. PLoS One. 2012;7(10):e44176.

493. Pettigrew CA, Clerkin JS, Cotter TG. DUOX enzyme activity promotes AKT signalling in prostate cancer cells. Anticancer Res. 2012;32(12):5175-81.

494. Pettigrew CA, Clerkin JS, Cotter TG. DUOX Enzyme Activity Promotes AKT Signalling in Prostate Cancer Cells. Anticancer Res. 2012;32(12):5175-81.

495. Prantner D, Perkins DJ, Lai W, Williams MS, Sharma S, Fitzgerald KA, et al. 5,6-Dimethylxanthenone-4-acetic acid (DMXAA) activates stimulator of interferon gene (STING)-dependent innate immune pathways and is regulated by mitochondrial membrane potential. J Biol Chem. 2012;287(47):39776-88.

496. Prantner D, Perkins DJ, Lai W, Williams MS, Sharma S, Fitzgerald KA, et al. 5,6-Dimethylxanthenone-4-acetic acid (DMXAA) activates stimulator of interferon gene (STING)-dependent innate immune pathways and is regulated by mitochondrial membrane potential. J Biol Chem. 2012;287(47):39776-88.

497. Pratheeshkumar P, Sreekala C, Zhang Z, Budhraja A, Ding S, Son YO, et al. Cancer prevention with promising natural products: Mechanisms of action and molecular targets. Anti-Cancer Agents Med Chem. 2012;12(10):1159-84.

498. Priyadarsini RV, Nagini S. Cancer chemoprevention by dietary phytochemicals: Promises and pitfalls. Current Pharmaceutical Biotechnology. 2012;13(1):125-36.

499. Reuben SC, Gopalan A, Petit DM, Bishayee A. Modulation of angiogenesis by dietary phytoconstituents in the prevention and intervention of breast cancer. Molecular Nutrition and Food Research. 2012;56(1):14-29.

500. Rudzitis-Auth J, Körbel C, Scheuer C, Menger MD, Laschke MW. Xanthohumol inhibits growth and vascularization of developing endometriotic lesions. Hum Reprod. 2012;27(6):1735-44.

501. Rudzitis-Auth J, Krbel C, Scheuer C, Menger MD, Laschke MW. Xanthohumol inhibits growth and vascularization of developing endometriotic lesions. Hum Reprod. 2012;27(6):1735-44.

502. Singh DJ, Lohade AA, Parmar JJ, Hegde DD, Soni P, Samad A, et al. Development of Chitosan-based Dry Powder Inhalation System of Cisplatin for Lung Cancer. Indian J Pharm Sci. 2012;74(6):521-6.

503. Singh DJ, Lohade AA, Parmar JJ, Hegde DD, Soni P, Samad A, et al. Development of Chitosan-based Dry Powder Inhalation System of Cisplatin for Lung Cancer. Indian J Pharm Sci. 2012;74(6):521-6.

504. Singh MM, Irwin ME, Gao Y, Ban K, Shi P, Arlinghaus RB, et al. Inhibition of the NADPH oxidase regulates heme oxygenase 1 expression in chronic myeloid leukemia. Cancer. 2012;118(13):3433-45.

505. Singh MM, Irwin ME, Gao Y, Ban KC, Shi P, Arlinghaus RB, et al. Inhibition of the NADPH oxidase regulates heme oxygenase 1 expression in chronic myeloid leukemia. Cancer. 2012;118(13):3433-45.

506. Stȩpnik M, Ferlińska M, Smok-Pienia̧zek A, Gradecka-Meesters D, Arkusz J, Stańczyk M. Assessment of the involvement of oxidative stress and Mitogen-Activated Protein Kinase signaling pathways in the cytotoxic effects of arsenic trioxide and its combination with sulindac or its metabolites: Sulindac sulfide and sulindac sulfone on human leukemic cell lines. Med Oncol. 2012;29(2):1161-72.

507. Stępnik M, Ferlińska M, Smok-Pieniążek A, Gradecka-Meesters D, Arkusz J, Stańczyk M. Assessment of the involvement of oxidative stress and Mitogen-Activated Protein Kinase signaling pathways in the cytotoxic effects of arsenic trioxide and its combination with sulindac or its metabolites: sulindac sulfide and sulindac sulfone on human leukemic cell lines. Med Oncol. 2012;29(2):1161-72.

508. Tseng HY, Liu ZM, Huang HS. NADPH oxidase-produced superoxide mediates EGFR transactivation by c-Src in arsenic trioxide-stimulated human keratinocytes. Arch Toxicol. 2012;86(6):935-45.

509. Vadodkar AS, Suman S, Lakshmanaswamy R, Damodaran C. Chemoprevention of breast cancer by dietary compounds. Anti-Cancer Agents Med Chem. 2012;12(10):1185-202.

510. Weng CJ, Yen GC. Chemopreventive effects of dietary phytochemicals against cancer invasion and metastasis: Phenolic acids, monophenol, polyphenol, and their derivatives. Cancer Treatment Reviews. 2012;38(1):76-87.

511. Weng JR, Bai LY, Chiu CF, Wang YC, Tsai MH. The dietary phytochemical 3,3'-diindolylmethane induces G2/M arrest and apoptosis in oral squamous cell carcinoma by modulating Akt-NF-κB, MAPK, and p53 signaling. Chem Biol Interact. 2012;195(3):224-30.

512. Weng JR, Bai LY, Chiu CF, Wang YC, Tsai MH. The dietary phytochemical 3,3′-diindolylmethane induces G2/M arrest and apoptosis in oral squamous cell carcinoma by modulating Akt-NF-κB, MAPK, and p53 signaling. Chem-Biol Interact. 2012;195(3):224-30.

513. Whitlock NC, Baek SJ. The anticancer effects of resveratrol: Modulation of transcription factors. Nutrition and Cancer. 2012;64(4):493-502.

514. Whitlock NC, Baek SJ. The anticancer effects of resveratrol: modulation of transcription factors. Nutr Cancer. 2012;64(4):493-502.

515. Woolley JF, Naughton R, Stanicka J, Gough DR, Bhatt L, Dickinson BC, et al. H2O2 production downstream of FLT3 is mediated by p22phox in the endoplasmic reticulum and is required for STAT5 signalling. PLoS One. 2012;7(7).

516. Xiao J, Wang Y, Peng J, Guo L, Hu J, Cao M, et al. A synthetic compound, 1,5-bis(2-methoxyphenyl)penta-1,4-dien-3-one (B63), induces apoptosis and activates endoplasmic reticulum stress in non-small cell lung cancer cells. Int J Cancer. 2012;131(6):1455-65.

517. Xiao J, Wang Y, Peng J, Guo L, Hu J, Cao MH, et al. A synthetic compound, 1,5-bis(2-methoxyphenyl)penta-1,4-dien-3-one (B63), induces apoptosis and activates endoplasmic reticulum stress in non-small cell lung cancer cells. Int J Cancer. 2012;131(6):1455-65.

518. Zhang L, Li L, Jiao M, Wu D, Wu K, Li X, et al. Genistein inhibits the stemness properties of prostate cancer cells through targeting Hedgehog-Gli1 pathway. Cancer Lett. 2012;323(1):48-57.

519. Zhang LL, Li L, Jiao M, Wu DP, Wu KJ, Li X, et al. Genistein inhibits the sternness properties of prostate cancer cells through targeting Hedgehog-Gli1 pathway. Cancer Lett. 2012;323(1):48-57.

520. Abdull Razis AF, Mohd Noor N. Cruciferous vegetables: Dietary phytochemicals for cancer prevention. Asian Pac J Cancer Prev. 2013;14(3):1565-70.

521. Ahmad N, Mukhtar H. Antioxidants meet molecular targets for cancer prevention and therapeutics. Antioxid Redox Signal. 2013;19(2):85-8.

522. Arango D, Morohashi K, Yilmaz A, Kuramochi K, Parihar A, Brahimaj B, et al. Molecular basis for the action of a dietary flavonoid revealed by the comprehensive identi fication of apigenin human targets. Proc Natl Acad Sci U S A. 2013;110(24):E2153-E62.

523. Arul D, Subramanian P. Naringenin (citrus flavonone) induces growth inhibition, cell cycle arrest and apoptosis in human hepatocellular carcinoma cells. Pathol Oncol Res. 2013;19(4):763-70.

524. Bahadoran Z, Karimi Z, Houshiar-rad A, Mirzayi HR, Rashidkhani B. Dietary phytochemical index and the risk of breast cancer: a case control study in a population of Iranian women. Asian Pac J Cancer Prev. 2013;14(5):2747-51.

525. Bahadoran Z, Karimi Z, Houshiar-rad A, Mirzayi HR, Rashidkhani B. Dietary Phytochemical Index and the Risk of Breast Cancer: A Case Control Study in a Population of Iranian Women. Asian Pac J Cancer Prev. 2013;14(5):2747-51.

526. Bode AM, Dong Z. Signal transduction and molecular targets of selected flavonoids. Antioxid Redox Signal. 2013;19(2):163-80.

527. Bommareddy A, Eggleston W, Prelewicz S, Antal A, Witczak Z, McCune DF, et al. Chemoprevention of prostate cancer by major dietary phytochemicals. Anticancer Res. 2013;33(10):4163-74.

528. Brahmbhatt M, Gundala SR, Asif G, Shamsi SA, Aneja R. Ginger phytochemicals exhibit synergy to inhibit prostate cancer cell proliferation. Nutr Cancer. 2013;65(2):263-72.

529. Carnicer A, Angellier G, Thariat J, Sauerwein W, Caujolle JP, Hérault J. Quantification of dose perturbations induced by external and internal accessories in ocular proton therapy and evaluation of their dosimetric impact. Med Phys. 2013;40(6):061708.

530. Carnicer A, Angellier G, Thariat J, Sauerwein W, Caujolle JP, Hérault J. Quantification of dose perturbations induced by external and internal accessories in ocular proton therapy and evaluation of their dosimetric impact. Med Phys. 2013;40(6):12.

531. Catania A, Barrajón-Catalán E, Nicolosi S, Cicirata F, Micol V. Immunoliposome encapsulation increases cytotoxic activity and selectivity of curcumin and resveratrol against HER2 overexpressing human breast cancer cells. Breast Cancer Res Treat. 2013;141(1):55-65.

532. Citrin DE, Shankavaram U, Horton JA, Shield Iii W, Zhao S, Asano H, et al. Role of type II pneumocyte senescence in radiation-induced lung fibrosis. Journal of the National Cancer Institute. 2013;105(19):1474-84.

533. Citrin DE, Shankavaram U, Horton JA, Shield W, Zhao SP, Asano H, et al. Role of Type II Pneumocyte Senescence in Radiation-Induced Lung Fibrosis. JNCI-J Natl Cancer Inst. 2013;105(19):1474-84.

534. Doroshow JH, Gaur S, Markel S, Lu J, van Balgooy J, Synold TW, et al. Effects of iodonium-class flavin dehydrogenase inhibitors on growth, reactive oxygen production, cell cycle progression, NADPH oxidase 1 levels, and gene expression in human colon cancer cells and xenografts. Free Radic Biol Med. 2013;57:162-75.

535. Doroshow JH, Gaur S, Markel S, Lu J, van Balgooy J, Synold TW, et al. Effects of iodonium-class flavin dehydrogenase inhibitors on growth, reactive oxygen production, cell cycle progression, NADPH oxidase 1 levels, and gene expression in human colon cancer cells and xenografts. Free Radic Biol Med. 2013;57:162-75.

536. Doucette CD, Hilchie AL, Liwski R, Hoskin DW. Piperine, a dietary phytochemical, inhibits angiogenesis. J Nutr Biochem. 2013;24(1):231-9.

537. Gatto A, De Gaetano AM, Giuga M, Ciresa M, Siciliani L, Miele L, et al. Differentiating hepatocellular carcinoma from dysplastic nodules at gadobenate dimeglumine-enhanced hepatobiliary-phase magnetic resonance imaging. Abdom Imaging. 2013;38(4):736-44.

538. Gatto A, De Gaetano AM, Giuga M, Ciresa M, Siciliani L, Miele L, et al. Differentiating hepatocellular carcinoma from dysplastic nodules at gadobenate dimeglumine-enhanced hepatobiliary-phase magnetic resonance imaging. Abdom Imaging. 2013;38(4):736-44.

539. Goher M, Hicks JA, Liu HC. The interplay between MDV and HVT affects viral miRNA expression. Avian Dis. 2013;57(2 SUPPL. 1):372-9.

540. Goher M, Hicks JA, Liu HC. The interplay between MDV and HVT affects viral miRNa expression. Avian Dis. 2013;57(2 Suppl):372-9.

541. González-Vallinas M, González-Castejón M, Rodríguez-Casado A, Ramírez de Molina A. Dietary phytochemicals in cancer prevention and therapy: A complementary approach with promising perspectives. Nutrition Reviews. 2013;71(9):585-99.

542. Jang H, Koo BS, Jeon EO, Lee HR, Lee SM, Mo IP. Altered pro-inflammatory cytokine mRNA levels in chickens infected with infectious bronchitis virus. Poult Sci. 2013;92(9):2290-8.

543. Jang H, Koo BS, Jeon EO, Lee HR, Lee SM, Mo IP. Altered pro-inflammatory cytokine mRNA levels in chickens infected with infectious bronchitis virus. Poult Sci. 2013;92(9):2290-8.

544. Jang IS, Margolin A, Califano A. hARACNe: improving the accuracy of regulatory model reverse engineering via higher-order data processing inequality tests. Interface Focus. 2013;3(4):20130011.

545. Jang IS, Margolin A, Califano A. hARACNe: improving the accuracy of regulatory model reverse engineering via higher-order data processing inequality tests. Interface Focus. 2013;3(4):10.

546. Jie H, Lian L, Qu LJ, Zheng JX, Hou ZC, Xu GY, et al. Differential expression of Toll-like receptor genes in lymphoid tissues between Marek's disease virus-infected and noninfected chickens. Poult Sci. 2013;92(3):645-54.

547. Jie H, Lian L, Qu LJ, Zheng JX, Hou ZC, Xu GY, et al. Differential expression of Toll-like receptor genes in lymphoid tissues between Marek's disease virus-infected and noninfected chickens. Poult Sci. 2013;92(3):645-54.

548. Jung CL, Kim HJ, Park JHY, Kong ANT, Lee CH, Kim JS. Synergistic activation of the Nrf2-signaling pathway by glyceollins under oxidative stress induced by glutathione depletion. J Agric Food Chem. 2013;61(17):4072-8.

549. Karthik L, Kumar G, Keswani T, Bhattacharyya A, Reddy BP, Rao KVB. Marine actinobacterial mediated gold nanoparticles synthesis and their antimalarial activity. Nanomedicine: Nanotechnology, Biology, and Medicine. 2013;9(7):951-60.

550. Kaur P, Shorey LE, Ho E, Dashwood RH, Williams DE. The epigenome as a potential mediator of cancer and disease prevention in prenatal development. Nutrition Reviews. 2013;71(7):441-57.

551. Kavitha K, Thiyagarajan P, Rathna J, Mishra R, Nagini S. Chemopreventive effects of diverse dietary phytochemicals against DMBA-induced hamster buccal pouch carcinogenesis via the induction of Nrf2-mediated cytoprotective antioxidant, detoxification, and DNA repair enzymes. Biochimie. 2013;95(8):1629-39.

552. Kawamoto EM, Scavone C, Mattson MP, Camandola S. Curcumin requires tumor necrosis factor α signaling to alleviate cognitive impairment elicited by lipopolysaccharide. Neurosignals. 2013;21(1-2):75-88.

553. Kim Y, Kim YS, Kim DE, Lee JS, Song JH, Kim HG, et al. BIX-01294 induces autophagy-associated cell death via EHMT2/G9a dysfunction and intracellular reactive oxygen species production. Autophagy. 2013;9(12):2126-39.

554. Lee JH, Khor TO, Shu L, Su ZY, Fuentes F, Kong AN. Dietary phytochemicals and cancer prevention: Nrf2 signaling, epigenetics, and cell death mechanisms in blocking cancer initiation and progression. Pharmacol Ther. 2013;137(2):153-71.

555. Lee JH, Khor TO, Shu L, Su ZY, Fuentes F, Kong ANT. Dietary phytochemicals and cancer prevention: Nrf2 signaling, epigenetics, and cell death mechanisms in blocking cancer initiation and progression. Pharmacology and Therapeutics. 2013;137(2):153-71.

556. Lee YJ, Lee YJ, Im JH, Won SY, Kim YB, Cho MK, et al. Synergistic anti-cancer effects of resveratrol and chemotherapeutic agent clofarabine against human malignant mesothelioma MSTO-211H cells. Food Chem Toxicol. 2013;52:61-8.

557. Li J, Yang JY, Yao XC, Xue X, Zhang QC, Wang XX, et al. Oligomeric Aβ-Induced Microglial Activation is Possibly Mediated by NADPH Oxidase. Neurochem Res. 2013;38(2):443-52.

558. Li J, Yang JY, Yao XC, Xue X, Zhang QC, Wang XX, et al. Oligomeric Aβ-induced microglial activation is possibly mediated by NADPH oxidase. Neurochem Res. 2013;38(2):443-52.

559. Martel-Gallegos G, Casas-Pruneda G, Ortega-Ortega F, Sánchez-Armass S, Olivares-Reyes JA, Diebold B, et al. Oxidative stress induced by P2X7 receptor stimulation in murine macrophages is mediated by c-Src/Pyk2 and ERK1/2. Biochimica et Biophysica Acta - General Subjects. 2013;1830(10):4650-9.

560. Martínez-Rodríguez I, De Arcocha Torres M, Banzo I, Quirce R, Jimenez-Bonilla J, Medina-Quiroz P, et al. Evaluation of the contribution of the dynamic phase of lymphoscintigraphy to the detection of sentinel lymph node in breast cancer. Q J Nucl Med Mol Imag. 2013;57(3):296-300.

561. Martínez-Rodríguez I, De Arcocha Torres M, Banzo I, Quirce R, Jiménez-Bonilla J, Medina-Quiroz P, et al. Evaluation of the contribution of the dynamic phase of lymphoscintigraphy to the detection of sentinel lymph node in breast cancer. Q J Nucl Med Mol Imaging. 2013;57(3):296-300.

562. Martínez-Rodríguez I, De Arcocha Torres M, Banzo I, Quirce R, Jiménez-Bonilla J, Medina-Quiroz P, et al. Evaluation of the contribution of the dynamic phase of lymphoscintigraphy to the detection of sentinel lymph node in breast cancer. Q J Nucl Med Mol Imaging. 2013.

563. Meenach SA, Anderson KW, Zach Hilt J, McGarry RC, Mansour HM. Characterization and aerosol dispersion performance of advanced spray-dried chemotherapeutic PEGylated phospholipid particles for dry powder inhalation delivery in lung cancer. Eur J Pharm Sci. 2013;49(4):699-711.

564. Parasramka MA, Ali S, Banerjee S, Deryavoush T, Sarkar FH, Gupta S. Garcinol sensitizes human pancreatic adenocarcinoma cells to gemcitabine in association with microRNA signatures. Molecular Nutrition and Food Research. 2013;57(2):235-48.

565. Samarakoon R, Dobberfuhl AD, Cooley C, Overstreet JM, Patel S, Goldschmeding R, et al. Induction of renal fibrotic genes by TGF-β1 requires EGFR activation, p53 and reactive oxygen species. Cell Signal. 2013;25(11):2198-209.

566. Shankar S, Kumar D, Srivastava RK. Epigenetic modifications by dietary phytochemicals: Implications for personalized nutrition. Pharmacology and Therapeutics. 2013;138(1):1-17.

567. Siddesha JM, Valente AJ, Sakamuri SS, Yoshida T, Gardner JD, Somanna N, et al. Angiotensin II stimulates cardiac fibroblast migration via the differential regulation of matrixins and RECK. J Mol Cell Cardiol. 2013;65:9-18.

568. Sobhakumari A, Schickling BM, Love-Homan L, Raeburn A, Fletcher EV, Case AJ, et al. NOX4 mediates cytoprotective autophagy induced by the EGFR inhibitor erlotinib in head and neck cancer cells. Toxicol Appl Pharmacol. 2013;272(3):736-45.

569. Speciale A, Anwar S, Canali R, Chirafisi J, Saija A, Virgili F, et al. Cyanidin-3-O-glucoside counters the response to TNF-alpha of endothelial cells by activating Nrf2 pathway. Molecular Nutrition and Food Research. 2013;57(11):1979-87.

570. Su J, Xu Y, Zhou L, Yu HM, Kang JS, Liu N, et al. Suppression of chloride channel 3 expression facilitates sensitivity of human glioma U251 cells to cisplatin through concomitant inhibition of Akt and autophagy. Anat Rec (Hoboken). 2013;296(4):595-603.

571. Su J, Xu Y, Zhou L, Yu HM, Kang JS, Liu N, et al. Suppression of Chloride Channel 3 Expression Facilitates Sensitivity of Human Glioma U251 Cells to Cisplatin Through Concomitant Inhibition of Akt and Autophagy. Anat Rec. 2013;296(4):595-603.

572. Su ZY, Shu L, Khor TO, Lee JH, Fuentes F, Kong AN. A perspective on dietary phytochemicals and cancer chemoprevention: oxidative stress, nrf2, and epigenomics. Top Curr Chem. 2013;329:133-62.

573. Syed DN, Chamcheu JC, Adhami VM, Mukhtar H. Pomegranate extracts and cancer prevention: Molecular and cellular activities. Anti-Cancer Agents Med Chem. 2013;13(8):1149-61.

574. Tamanoi F, Lu J. Recent Progress in Developing Small Molecule Inhibitors Designed to Interfere with Ras Membrane Association: Toward Inhibiting K-Ras and N-Ras Functions. Enzymes. 342013. p. 181-200.

575. Tan KW, Li Y, Paxton JW, Birch NP, Scheepens A. Identification of novel dietary phytochemicals inhibiting the efflux transporter breast cancer resistance protein (BCRP/ABCG2). Food Chemistry. 2013;138(4):2267-74.

576. Wang LF, Huang ZC, Wu XF, Wang HF. [Primary mechanism of the role of dual oxidase-1 causing airway allergic diseases in human bronchial epithelium]. Zhonghua er bi yan hou tou jing wai ke za zhi = Chinese journal of otorhinolaryngology head and neck surgery. 2013;48(10):823-9.

577. Watson JM, Crosby H, Dale VM, Tober G, Wu Q, Lang J, et al. AESOPS: a randomised controlled trial of the clinical effectiveness and cost-effectiveness of opportunistic screening and stepped care interventions for older hazardous alcohol users in primary care. Health Technol Assess. 2013;17(25):1-+.

578. Wu TY, Khor TO, Lee JH, Cheung KL, Shu L, Chen C, et al. Pharmacogenetics, pharmacogenomics and epigenetics of Nrf2-regulated xenobioticmetabolizing enzymes and transporters by dietary phytochemical and cancer chemoprevention. Curr Drug Metab. 2013;14(6):688-94.

579. Wu TY, Khor TO, Lee JH, Cheung KL, Shu L, Chen C, et al. Pharmacogenetics, pharmacogenomics and epigenetics of Nrf2-regulated xenobiotic-metabolizing enzymes and transporters by dietary phytochemical and cancer chemoprevention. Curr Drug Metab. 2013;14(6):688-94.

580. Yaffe PB, Doucette CD, Walsh M, Hoskin DW. Piperine impairs cell cycle progression and causes reactive oxygen species-dependent apoptosis in rectal cancer cells. Exp Mol Pathol. 2013;94(1):109-14.

581. Yaffe PB, Doucette CD, Walsh M, Hoskin DW. Piperine impairs cell cycle progression and causes reactive oxygen species-dependent apoptosis in rectal cancer cells. Exp Mol Pathol. 2013;94(1):109-14.

582. Yu C, Liu Q, Qin A, Hu X, Xu W, Qian K, et al. Expression kinetics of chicken β2-microglobulin and Class i MHC in vitro and in vivo during Marek's disease viral infections. Vet Res Commun. 2013;37(4):277-83.

583. Yu C, Liu Q, Qin A, Hu X, Xu W, Qian K, et al. Expression kinetics of chicken β2-microglobulin and Class I MHC in vitro and in vivo during Marek's disease viral infections. Vet Res Commun. 2013;37(4):277-83.

584. Yuan X, Zhou Y, Wang W, Li J, Xie G, Zhao Y, et al. Activation of TLR4 signaling promotes gastric cancer progression by inducing mitochondrial ROS production. Cell Death and Disease. 2013;4(9).

585. Yuan X, Zhou Y, Wang W, Li J, Xie G, Zhao Y, et al. Activation of TLR4 signaling promotes gastric cancer progression by inducing mitochondrial ROS production. Cell Death Dis. 2013;4(9):e794.

586. Zheng S, Zhong ZM, Qin S, Chen GX, Wu Q, Zeng JH, et al. Advanced oxidation protein products induce inflammatory response in fibroblast-like synoviocytes through NADPH oxidase -dependent activation of NF-κB. Cell Physiol Biochem. 2013;32(4):972-85.

587. Zheng S, Zhong ZM, Qin S, Chen GX, Wu Q, Zeng JH, et al. Advanced oxidation protein products induce inflammatory response in fibroblast-like synoviocytes through NADPH oxidase -dependent activation of NF-κB. Cell Physiol Biochem. 2013;32(4):972-85.

588. Biniecka M, Connolly M, Gao W, Ng CT, Balogh E, Gogarty M, et al. Redox-mediated angiogenesis in the hypoxic joint of inflammatory arthritis. Arthritis Rheumatol. 2014;66(12):3300-10.

589. Biniecka M, Connolly M, Gao W, Ng CT, Balogh E, Gogarty M, et al. Redox-Mediated Angiogenesis in the Hypoxic Joint of Inflammatory Arthritis. Arthritis Rheumatol. 2014;66(12):3300-10.

590. Brown EM, Nitecki S, Pereira-Caro G, McDougall GJ, Stewart D, Rowland I, et al. Comparison of in vivo and in vitro digestion on polyphenol composition in lingonberries: Potential impact on colonic health. BioFactors. 2014;40(6):611-23.

591. Byun E, Lim JW, Kim JM, Kim H. α-Lipoic Acid Inhibits <i>Helicobacter</i> <i>pylori</i>-Induced Oncogene Expression and Hyperproliferation by Suppressing the Activation of NADPH Oxidase in Gastric Epithelial Cells. Mediat Inflamm. 2014;2014:12.

592. Carlisi D, D'Anneo A, Martinez R, Emanuele S, Buttitta G, Di Fiore R, et al. The oxygen radicals involved in the toxicity induced by parthenolide in MDA-MB-231 cells. Oncol Rep. 2014;32(1):167-72.

593. Carlisi D, D'Anneo A, Martinez R, Emanuele S, Buttitta G, Di Fiore R, et al. The oxygen radicals involved in the toxicity induced by parthenolide in MDA-MB-231 cells. Oncol Rep. 2014;32(1):167-72.

594. Festuccia C, Mancini A, Gravina GL, Scarsella L, Llorens S, Alonso GL, et al. Antitumor effects of saffron-derived carotenoids in prostate cancer cell models. Biomed Res Int. 2014;2014:135048.

595. Fuentes F, Shu L, Lee JH, Su ZY, Lee KR, Kong ANT. Nrf2-target approaches in cancer chemoprevention mediated by dietary phytochemicals. Methods in Pharmacology and Toxicology. 2014:53-83.

596. Grabacka MM, Gawin M, Pierzchalska M. Phytochemical modulators of mitochondria: The search for chemopreventive agents and supportive therapeutics. Pharmaceuticals. 2014;7(9):913-42.

597. Gundala SR, Yang C, Mukkavilli R, Paranjpe R, Brahmbhatt M, Pannu V, et al. Hydroxychavicol, a betel leaf component, inhibits prostate cancer through ROS-driven DNA damage and apoptosis. Toxicol Appl Pharmacol. 2014;280(1):86-96.

598. Hwang SD, Shim SH, Kwon MG, Chae YS, Shim WJ, Jung JH, et al. Molecular cloning and expression analysis of two lipopolysaccharide-induced TNF-α factors (LITAFs) from rock bream, Oplegnathus fasciatus. Fish Shellfish Immunol. 2014;36(2):467-74.

599. Hwang SD, Shim SH, Kwon MG, Chae YS, Shim WJ, Jung JH, et al. Molecular cloning and expression analysis of two lipopolysaccharide-induced TNF-α factors (LITAFs) from rock bream, <i>Oplegnathus fasciatus</i>. Fish Shellfish Immunol. 2014;36(2):467-74.

600. Islam MS, Akhtar MM, Ciavattini A, Giannubilo SR, Protic O, Janjusevic M, et al. Use of dietary phytochemicals to target inflammation, fibrosis, proliferation, and angiogenesis in uterine tissues: Promising options for prevention and treatment of uterine fibroids? Molecular Nutrition and Food Research. 2014;58(8):1667-84.

601. Lin MH, Cheng CH, Chen KC, Lee WT, Wang YF, Xiao CQ, et al. Induction of ROS-independent JNK-activation-mediated apoptosis by a novel coumarin-derivative, DMAC, in human colon cancer cells. Chem-Biol Interact. 2014;218:42-9.

602. Liu Y, Cui Y, Shi M, Zhang Q, Wang Q, Chen X. Deferoxamine promotes MDA-MB-231 cell migration and invasion through increased ros-dependent HIF-1α accumulation. Cell Physiol Biochem. 2014;33(4):1036-46.

603. Moe KT, Khairunnisa K, Yin NO, Chin-Dusting J, Wong P, Wong MC. Tumor necrosis factor-α-induced nuclear factor-kappaB activation in human cardiomyocytes is mediated by NADPH oxidase. J Physiol Biochem. 2014;70(3):769-79.

604. Moe KT, Khairunnisa K, Yin NO, Chin-Dusting J, Wong P, Wong MC. Tumor necrosis factor-α-induced nuclear factor-kappaB activation in human cardiomyocytes is mediated by NADPH oxidase. J Physiol Biochem. 2014;70(3):769-79.

605. Mohammadi-Bardbori A, Rannug A. Arsenic, cadmium, mercury and nickel stimulate cell growth via NADPH oxidase activation. Chem-Biol Interact. 2014;224:183-8.

606. Mohammadi-Bardbori A, Rannug A. Arsenic, cadmium, mercury and nickel stimulate cell growth via NADPH oxidase activation. Chem Biol Interact. 2014;224:183-8.

607. Noriega-Navarro R, Lopez-Charcas O, Hernández-Enríquez B, Reyes-Gutiérrez PE, Martínez R, Landa A, et al. Novel TASK channels inhibitors derived from dihydropyrrolo[2,1-a] isoquinoline. Neuropharmacology. 2014;79:28-36.

608. Noriega-Navarro R, Lopez-Charcas O, Hernández-Enríquez B, Reyes-Gutiérrez PE, Martínez R, Landa A, et al. Novel TASK channels inhibitors derived from dihydropyrrolo[2,1-a]isoquinoline. Neuropharmacology. 2014;79:28-36.

609. Paredes-Gonzalez X, Fuentes F, Su ZY, Kong AN. Apigenin reactivates Nrf2 anti-oxidative stress signaling in mouse skin epidermal JB6 P + cells through epigenetics modifications. Aaps j. 2014;16(4):727-35.

610. Parikh NR, Mandal A, Bhatia D, Siveen KS, Sethi G, Bishayee A. Oleanane triterpenoids in the prevention and therapy of breast cancer: current evidence and future perspectives. Phytochemistry Reviews. 2014;13(4):793-810.

611. Ping M, Xiao W, Mo L, Xiao X, Song S, Tang W, et al. Paeonol attenuates advanced oxidation protein product-induced oxidative stress injury in THP-1 Macrophages. Pharmacology. 2014;93(5-6):286-95.

612. Ping M, Xiao W, Mo L, Xiao X, Song S, Tang W, et al. Paeonol attenuates advanced oxidation protein product-induced oxidative stress injury in THP-1 macrophages. Pharmacology. 2014;93(5-6):286-95.

613. Poanta L, Serban O, Pascu I, Pop S, Cosgarea M, Fodor D. The place of CEUS in distinguishing benign from malignant cervical lymph nodes: a prospective study. Med Ultrason. 2014;16(1):7-14.

614. Poanta L, Serban O, Pascu I, pop S, Cosgarea M, Fodor D. The place of CEUS in distinguishing benign from malignant cervical lymph nodes: A prospective study. Med Ultrason. 2014;16(1):7-14.

615. Pomázi A, Ambrus R, Szabó-Révész P. Physicochemical stability and aerosolization performance of mannitol-based microcomposites. J Drug Deliv Sci Technol. 2014;24(4):397-403.

616. Pooja T, Karunagaran D. Emodin suppresses Wnt signaling in human colorectal cancer cells SW480 and SW620. Eur J Pharmacol. 2014;742:55-64.

617. Qiao S, Fan K, Iwashita T, Ichihara M, Yoshino M, Takahashi M. The involvement of reactive oxygen species derived from NADPH oxidase-1 activation on the constitutive tyrosine auto-phosphorylation of RET proteins. Free Radic Res. 2014;48(4):427-34.

618. Qiao S, Fan K, Iwashita T, Ichihara M, Yoshino M, Takahashi M. The involvement of reactive oxygen species derived from NADPH oxidase-1 activation on the constitutive tyrosine auto-phosphorylation of RET proteins. Free Radic Res. 2014;48(4):427-34.

619. Ruan L, Wang S, Zhang J, Ai H, Zhao Q, Yang J, et al. Doppler perfusion index and contrast-enhanced ultrasound in patients with colorectal cancer liver metastases. Hepato-Gastroenterol. 2014;61(129):37-41.

620. Ruan L, Wang S, Zhang J, Ai H, Zhao Q, Yang J, et al. Doppler perfusion index and contrast-enhanced ultrasound in patients with colorectal cancer liver metastases. Hepatogastroenterology. 2014;61(129):37-41.

621. Saxena A, Kaur K, Hegde S, Kalekhan F, Baliga M, Fayad R. Dietary agents and phytochemicals in the prevention and treatment of experimental ulcerative colitis. Journal of Traditional and Complementary Medicine. 2014;4(4):203-17.

622. Senggunprai L, Kukongviriyapan V, Prawan A, Kukongviriyapan U. Quercetin and EGCG exhibit chemopreventive effects in cholangiocarcinoma cells via suppression of JAK/STAT signaling pathway. Phytotherapy Research. 2014;28(6):841-8.

623. Shukla S, Meeran SM, Katiyar SK. Epigenetic regulation by selected dietary phytochemicals in cancer chemoprevention. Cancer Lett. 2014;355(1):9-17.

624. Singh BN, Singh HB, Singh A, Naqvi AH, Singh BR. Dietary phytochemicals alter epigenetic events and signaling pathways for inhibition of metastasis cascade: Phytoblockers of metastasis cascade. Cancer and Metastasis Reviews. 2014;33(1):41-85.

625. Subramaniam D, Thombre R, Dhar A, Anant S. DNA methyltransferases: A novel target for prevention and therapy. Front Oncol. 2014;4 MAY.

626. Sudan S, Rupasinghe HP. Flavonoid-enriched apple fraction AF4 induces cell cycle arrest, DNA topoisomerase II inhibition, and apoptosis in human liver cancer HepG2 cells. Nutr Cancer. 2014;66(7):1237-46.

627. Tobar N, Toyos M, Urra C, Méndez N, Arancibia R, Smith PC, et al. c-Jun N terminal kinase modulates NOX-4 derived ROS production and myofibroblasts differentiation in human breast stromal cells. BMC Cancer. 2014;14(1).

628. Tobar N, Toyos M, Urra C, Méndez N, Arancibia R, Smith PC, et al. c-Jun N terminal kinase modulates NOX-4 derived ROS production and myofibroblasts differentiation in human breast stromal cells. BMC Cancer. 2014;14:640.

629. Tuorkey M. Curcumin a potent cancer preventive agent: Mechanisms of cancer cell killing. Interventional Medicine and Applied Science. 2014;6(4):139-46.

630. Vergara D, Simeone P, Bettini S, Tinelli A, Valli L, Storelli C, et al. Antitumor activity of the dietary diterpene carnosol against a panel of human cancer cell lines. Food and Function. 2014;5(6):1261-9.

631. Vijayarathna S, Sasidharan S. Coaxing cancer pro-apoptoticity: An approach blending therapeutic miRNAs and dietary phytochemicals. Asian Pac J Cancer Prev. 2014;15(13):5499-500.

632. Wawszczyk J, Kapral M, Hollek A, Węglarz L. In vitro evaluation of antiproliferative and cytotoxic properties of pterostilbene against human colon cancer cells. Acta Poloniae Pharmaceutica - Drug Research. 2014;71(6):1051-5.

633. Witkowska J, Karpowicz P, Gaczynska M, Osmulski PA, Jankowska E. Dissecting a role of a charge and conformation of Tat2 peptide in allosteric regulation of 20S proteasome. J Pept Sci. 2014;20(8):649-56.

634. Yim CY, Mao P, Spinella MJ. Headway and hurdles in the clinical development of dietary phytochemicals for cancer therapy and prevention: Lessons learned from vitamin A derivatives. AAPS Journal. 2014;16(2):281-8.

635. Zhang S, Wang Y, Li SJ. Lansoprazole induces apoptosis of breast cancer cells through inhibition of intracellular proton extrusion. Biochem Biophys Res Commun. 2014;448(4):424-9.

636. Zhang S, Wang Y, Li SJ. Lansoprazole induces apoptosis of breast cancer cells through inhibition of intracellular proton extrusion. Biochem Biophys Res Commun. 2014;448(4):424-9.

637. Zhang WW, Feng Z, Narod SA. Multiple therapeutic and preventive effects of 3,3'-diindolylmethane on cancers including prostate cancer and high grade prostatic intraepithelial neoplasia. Journal of Biomedical Research. 2014;28(5):339-48.

638. Abbas A, Hall JA, Patterson WL, Ho E, Hsu A, Al-Mulla F, et al. Sulforaphane modulates telomerase activity via epigenetic regulation in prostate cancer cell lines1. Biochemistry and Cell Biology. 2015;94(1):71-81.

639. Banskota S, Regmi SC, Kim JA. NOX1 to NOX2 switch deactivates AMPK and induces invasive phenotype in colon cancer cells through overexpression of MMP-7. Mol Cancer. 2015;14:123.

640. Banskota S, Regmi SC, Kim JA. NOX1 to NOX2 switch deactivates AMPK and induces invasive phenotype in colon cancer cells through overexpression of MMP-7. Mol Cancer. 2015;14:14.

641. Banudevi S, Swaminathan S, Maheswari KU. Pleiotropic Role of Dietary Phytochemicals in Cancer: Emerging Perspectives for Combinational Therapy. Nutr Cancer. 2015;67(7):1021-48.

642. Boyanapalli SSS, Kong ANT. “Curcumin, the King of Spices”: Epigenetic Regulatory Mechanisms in the Prevention of Cancer, Neurological, and Inflammatory Diseases. Current Pharmacology Reports. 2015;1(2):129-39.

643. Darakhshan S, Bidmeshki Pour A, Hosseinzadeh Colagar A, Sisakhtnezhad S. Thymoquinone and its therapeutic potentials. Pharmacol Res. 2015;95-96:138-58.

644. Darakhshan S, Pour AB, Colagar AH, Sisakhtnezhad S. Thymoquinone and its therapeutic potentials. Pharmacol Res. 2015;95-96:138-58.

645. Ding Y, Zhu W, Sun R, Yuan G, Zhang D, Fan Y, et al. Diphenylene iodonium interferes with cell cycle progression and induces apoptosis by modulating NAD(P)H oxidase/ROS/cell cycle regulatory pathways in Burkitt's lymphoma cells. Oncol Rep. 2015;33(3):1434-42.

646. Ding Y, Zhu WJ, Sun R, Yuan G, Zhang DS, Fan YH, et al. Diphenylene iodonium interferes with cell cycle progression and induces apoptosis by modulating NAD(P)H oxidase/ROS/cell cycle regulatory pathways in Burkitt's lymphoma cells. Oncol Rep. 2015;33(3):1434-42.

647. Elatrech I, Marzaioli V, Boukemara H, Bournier O, Neut C, Darfeuille-Michaud A, et al. Escherichia coli LF82 differentially regulates ROS production and mucin expression in intestinal epithelial T84 cells: implication of NOX1. Inflamm Bowel Dis. 2015;21(5):1018-26.

648. Esparza-Araiza MJ, Bañuelos-Hernández B, Argüello-Astorga GR, Lara-Ávila JP, Goodwin PH, Isordia-Jasso MI, et al. Evaluation of a SUMO E2 conjugating enzyme involved in resistance to clavibacter michiganensis subsp. Michiganensis in solanum peruvianum, through a tomato mottle virus VIGS assay. Frontiers in Plant Science. 2015;6(DEC).

649. Galal AM, Walker LA, Khan IA. Induction of GST and related events by dietary phytochemicals: sources, chemistry, and possible contribution to chemoprevention. Curr Top Med Chem. 2015;14(24):2802-21.

650. Gandhi M, Pandya T, Gandhi R, Patel S, Mashru R, Misra A, et al. Inhalable liposomal dry powder of gemcitabine-HCl: Formulation, in vitro characterization and in vivo studies. Int J Pharm. 2015;496(2):886-95.

651. Gandhi M, Pandya T, Gandhi R, Patel S, Mashru R, Misra A, et al. Inhalable liposomal dry powder of gemcitabine-HCl: Formulation, <i>in vitro</i> characterization and <i>in vivo</i> studies. Int J Pharm. 2015;496(2):886-95.

652. Ghorbani A, Zand H, Jeddi-Tehrani M, Koohdani F, Shidfar F, Keshavarz SA. PTEN over-expression by resveratrol in acute lymphoblastic leukemia cells along with suppression of AKT/PKB and ERK1/2 in genotoxic stress. Journal of Natural Medicines. 2015;69(4):507-12.

653. Guo Y, Su ZY, Kong AN. Current Perspectives on Epigenetic Modifications by Dietary Chemopreventive and Herbal Phytochemicals. Curr Pharmacol Rep. 2015;1(4):245-57.

654. Hu X, Qin A, Xu W, Wu G, Li D, Qian K, et al. Transcriptional analysis of host responses to Marek's disease virus infection in chicken thymus. Intervirology. 2015;58(2):95-105.

655. Inchingolo R, De Gaetano AM, Curione D, Ciresa M, Miele L, Pompili M, et al. Role of diffusion-weighted imaging, apparent diffusion coefficient and correlation with hepatobiliary phase findings in the differentiation of hepatocellular carcinoma from dysplastic nodules in cirrhotic liver. Eur Radiol. 2015;25(4):1087-96.

656. Inchingolo R, De Gaetano AM, Curione D, Ciresa M, Miele L, Pompili M, et al. Role of diffusion-weighted imaging, apparent diffusion coefficient and correlation with hepatobiliary phase findings in the differentiation of hepatocellular carcinoma from dysplastic nodules in cirrhotic liver. Eur Radiol. 2015;25(4):1087-96.

657. Jones CM, Monge ME, Kim J, Matzuk MM, Fernández FM. Metabolomic serum profiling detects early-stage high-grade serous ovarian cancer in a mouse model. J Proteome Res. 2015;14(2):917-27.

658. Kapral M, Wawszczyk J, Sośnicki S, Węglarz L. DOWN-REGULATION OF INDUCIBLE NITRIC OXIDE SYNTHASE EXPRESSION BY INOSITOL HEXAPHOSPHATE IN HUMAN COLON CANCER CELLS. Acta Pol Pharm. 2015;72(4):705-11.

659. Kapral M, Wawszczyk J, Sosnicki S, Weglarz L. DOWN-REGULATION OF INDUCIBLE NITRIC OXIDE SYNTHASE EXPRESSION BY INOSITOL HEXAPHOSPHATE IN HUMAN COLON CANCER CELLS. Acta Pol Pharm. 2015;72(4):705-11.

660. Kasala ER, Bodduluru LN, Barua CC, Sriram CS, Gogoi R. Benzo(a)pyrene induced lung cancer: Role of dietary phytochemicals in chemoprevention. Pharmacol Rep. 2015;67(5):996-1009.

661. Kim HJ, Magesh V, Lee JJ, Kim S, Knaus UG, Lee KJ. Ubiquitin C-terminal hydrolase-L1 increases cancer cell invasion by modulating hydrogen peroxide generated via NADPH oxidase 4. Oncotarget. 2015;6(18):16287-303.

662. Kim HJ, Magesh V, Lee JJ, Kim S, Knaus UG, Lee KJ. Ubiquitin C-terminal hydrolase-L1 increases cancer cell invasion by modulating hydrogen peroxide generated via NADPH oxidase 4. Oncotarget. 2015;6(18):16287-303.

663. Kim SH, Kim KY, Yu SN, Park SK, Choi HD, Ji JH, et al. Autophagy inhibition enhances silibinin-induced apoptosis by regulating reactive oxygen species production in human prostate cancer PC-3 cells. Biochem Biophys Res Commun. 2015;468(1-2):151-6.

664. Kim SH, Kim KY, Yu SN, Park SK, Choi HD, Ji JH, et al. Autophagy inhibition enhances silibinin-induced apoptosis by regulating reactive oxygen species production in human prostate cancer PC-3 cells. Biochem Biophys Res Commun. 2015;468(1-2):151-6.

665. Kreikemeier CA, Engle TB, Lucot KL, Kachman SD, Burkey TE, Ciobanu DC. Genome-wide analysis of TNF-alpha response in pigs challenged with porcine circovirus 2b. Anim Genet. 2015;46(2):205-8.

666. Kreikemeier CA, Engle TB, Lucot KL, Kachman SD, Burkey TE, Ciobanu DC. Genome-wide analysis of TNF-alpha response in pigs challenged with porcine circovirus 2b. Anim Genet. 2015;46(2):205-8.

667. Lee HY, Mohammed KA, Goldberg EP, Kaye F, Nasreen N. Cisplatin loaded albumin mesospheres for lung cancer treatment. Am J Cancer Res. 2015;5(2):603-15.

668. Li E, Wang G, Yang Y, Xiao J, Mao Z, Xie B. Microscopic analysis of the compatible and incompatible interactions between Fusarium oxysporum f. sp. conglutinans and cabbage. European Journal of Plant Pathology. 2015;141(3):597-609.

669. Li K, Lian L, Yang N, Qu L. Temporal expression and DNA hypomethylation profile of CD30 in Marek's disease virus-infected chicken spleens. Poult Sci. 2015;94(6):1165-9.

670. Li S, Chen G, Wu M, Zhang J, Wu S. Restraining of reactive oxygen species promotes invasion of Listeria monocytogenes into glia cells. FEMS Microbiol Lett. 2015;363(2).

671. Lian L, Zhang D, Wang Q, Yang N, Qu L. The inhibitory effects of gga-MIR-199-3p, gga-MIR-140-3p, and gga-MIR-221-5p in Marek's disease tumorigenesis. Poult Sci. 2015;94(9):2131-5.

672. Lian L, Zhang D, Wang Q, Yang N, Qu L. The inhibitory effects of gga-miR-199-3p, gga-miR-140-3p, and gga-miR-221-5p in Marek's disease tumorigenesis. Poult Sci. 2015;94(9):2131-5.

673. Lian S, Xia Y, Khoi PN, Ung TT, Yoon HJ, Kim NH, et al. Cadmium induces matrix metalloproteinase-9 expression via ROS-dependent EGFR, NF-кB, and AP-1 pathways in human endothelial cells. Toxicology. 2015;338:104-16.

674. Lian S, Xia Y, Khoi PN, Ung TT, Yoon HJ, Kim NH, et al. Cadmium induces matrix metalloproteinase-9 expression via ROS-dependent EGFR, NF-κB, and AP-1 pathways in human endothelial cells. Toxicology. 2015;338:104-16.

675. Liu G, Yang G, Guan G, Zhang Y, Ren W, Yin J, et al. Effect of dietary selenium yeast supplementation on porcine circovirus type 2 (PCV2) infections in mice. PLoS One. 2015;10(2):e0115833.

676. Liu G, Yang G, Guan GP, Zhang YZ, Ren WK, Yin J, et al. Effect of Dietary Selenium Yeast Supplementation on Porcine Circovirus Type 2 (PCV2) Infections in Mice. PLoS One. 2015;10(2):11.

677. Lu H, Wu Q, Yang H. DUOX2 promotes the elimination of the Klebsiella pneumoniae strain K5 from T24 cells through the reactive oxygen species pathway. Int J Mol Med. 2015;36(2):551-8.

678. Lu H, Wu Q, Yang H. DUOX2 promotes the elimination of the Klebsiella pneumoniae strain K5 from T24 cells through the reactive oxygen species pathway. Int J Mol Med. 2015;36(2):551-8.

679. Mohammed A, Janakiram NB, Pant S, Rao CV. Molecular Targeted Intervention for Pancreatic Cancer. Cancers. 2015;7(3):1499-542.

680. Mohammed A, Janakiram NB, Pant S, Rao CV. Molecular Targeted Intervention for Pancreatic Cancer. Cancers (Basel). 2015;7(3):1499-542.

681. Nabavi SM, Habtemariam S, Daglia M, Nabavi SF. Apigenin and Breast Cancers: From Chemistry to Medicine. Anticancer Agents Med Chem. 2015;15(6):728-35.

682. Parrott MD, Winocur G, Bazinet RP, Ma DW, Greenwood CE. Whole-food diet worsened cognitive dysfunction in an Alzheimer's disease mouse model. Neurobiol Aging. 2015;36(1):90-9.

683. Radmard AR, Abrishami A, Gholamrezanezhad A, Kolahdoozan S, Sedighi N, Taheri AP. Relationship between CT volumetric measurements and Doppler perfusion indices in gastrointestinal liver metastasis. Radiol Med. 2015;120(2):171-9.

684. Radmard AR, Abrishami A, Gholamrezanezhad A, Kolahdoozan S, Sedighi N, Taheri APH. Relationship between CT volumetric measurements and Doppler perfusion indices in gastrointestinal liver metastasis. Radiol Med. 2015;120(2):171-9.

685. Rochell SJ, Alexander LS, Rocha GC, Van Alstine WG, Boyd RD, Pettigrew JE, et al. Effects of dietary soybean meal concentration on growth and immune response of pigs infected with porcine reproductive and respiratory syndrome virus. J Anim Sci. 2015;93(6):2987-97.

686. Rochell SJ, Alexander LS, Rocha GC, Van Alstine WG, Boyd RD, Pettigrew JE, et al. Effects of dietary soybean meal concentration on growth and immune response of pigs infected with porcine reproductive and respiratory syndrome virus. J Anim Sci. 2015;93(6):2987-97.

687. Rosière R, Gelbcke M, Mathieu V, Antwerpen P, Amighi K, Wauthoz N. New dry powders for inhalation containing temozolomide-based nanomicelles for improved lung cancer therapy. Int J Oncol. 2015;47(3):1131-42.

688. Rosière R, Gelbcke M, Mathieu V, Van Antwerpen P, Amighi K, Wauthoz N. New dry powders for inhalation containing temozolomide-based nanomicelles for improved lung cancer therapy. Int J Oncol. 2015;47(3):1131-42.

689. Sovadinova I, Babica P, Böke H, Kumar E, Wilke A, Park JS, et al. Phosphatidylcholine specific PLC-induced dysregulation of gap junctions, a robust cellular response to environmental toxicants, and prevention by Resveratrol in a rat liver cell model. PLoS One. 2015;10(5).

690. Sun C, Wang H, Mao S, Liu J, Li S, Wang J. Reactive oxygen species involved in CT26 immunogenic cell death induced by Clostridium difficile toxin B. Immunol Lett. 2015;164(2):65-71.

691. Sun C, Wang H, Mao S, Liu J, Li S, Wang J. Reactive oxygen species involved in CT26 immunogenic cell death induced by Clostridium difficile toxin B. Immunol Lett. 2015;164(2):65-71.

692. Sun J, Hu C, Zhu Y, Sun R, Fang Y, Fan Y, et al. LMP1 Increases Expression of NADPH Oxidase (NOX) and Its Regulatory Subunit p22 in NP69 Nasopharyngeal Cells and Makes Them Sensitive to a Treatment by a NOX Inhibitor. PLoS One. 2015;10(8):e0134896.

693. Sun J, Hu CY, Zhu YH, Sun R, Fang YJ, Fan YH, et al. LMP1 Increases Expression of NADPH Oxidase (NOX) and Its Regulatory Subunit p22 in NP69 Nasopharyngeal Cells and Makes Them Sensitive to a Treatment by a NOX Inhibitor. PLoS One. 2015;10(8):18.

694. Szarc Vel Szic K, Declerck K, Vidaković M, Vanden Berghe W. From inflammaging to healthy aging by dietary lifestyle choices: Is epigenetics the key to personalized nutrition? Clinical Epigenetics. 2015;7(1).

695. Takahashi R, Odera K. [An overview of current research of the effect of foods on aging and stress]. Yakugaku Zasshi. 2015;135(1):33-40.

696. Tanaka M, Miura Y, Numanami H, Karnan S, Ota A, Konishi H, et al. Inhibition of NADPH oxidase 4 induces apoptosis in malignant mesothelioma: Role of reactive oxygen species. Oncol Rep. 2015;34(4):1726-32.

697. Tanaka M, Miura Y, Numanami H, Karnan S, Ota A, Konishi H, et al. Inhibition of NADPH oxidase 4 induces apoptosis in malignant mesothelioma: Role of reactive oxygen species. Oncol Rep. 2015;34(4):1726-32.

698. Varshosaz J, Hassanzadeh F, Mardani A, Rostami M. Feasibility of haloperidol-anchored albumin nanoparticles loaded with doxorubicin as dry powder inhaler for pulmonary delivery. Pharm Dev Technol. 2015;20(2):183-96.

699. Varshosaz J, Hassanzadeh F, Mardani A, Rostami M. Feasibility of haloperidol-anchored albumin nanoparticles loaded with doxorubicin as dry powder inhaler for pulmonary delivery. Pharm Dev Technol. 2015;20(2):183-96.

700. Wang Y, Wang Y, Liu D, Wang W, Zhao H, Wang M, et al. Cordyceps sinensis polysaccharide inhibits PDGF-BB-induced inflammation and ROS production in human mesangial cells. Carbohydr Polym. 2015;125:135-45.

701. Yan S, Liu G, Pei C, Chen W, Li P, Wang Q, et al. Inhibition of NADPH oxidase protects against metastasis of human lung cancer by decreasing microRNA-21. Anti-Cancer Drugs. 2015;26(4):388-98.

702. Yan S, Liu G, Pei C, Chen W, Li P, Wang Q, et al. Inhibition of NADPH oxidase protects against metastasis of human lung cancer by decreasing microRNA-21. Anticancer Drugs. 2015;26(4):388-98.

703. Zhang Z, Liu S, Ma C, Zhao P, Cui Z. Absolute quantification of a very virulent Marek's disease virus dynamic quantity and distributions in different tissues. Poult Sci. 2015;94(6):1150-7.

704. Zhang Z, Liu S, Ma C, Zhao P, Cui Z. Absolute quantification of a very virulent Marek's disease virus dynamic quantity and distributions in different tissues. Poult Sci. 2015;94(6):1150-7.

705. Baena Ruiz R, Salinas Hernández P. Cancer chemoprevention by dietary phytochemicals: Epidemiological evidence. Maturitas. 2016;94:13-9.

706. Bernardes D, Oliveira-Lima OC, da Silva TV, Juliano MA, dos Santos DM, Carvalho-Tavares J. Metabolic Alterations in Experimental Autoimmune Encephalomyelitis in Mice: Effects of Prior Physical Exercise. Neurophysiology. 2016;48(2):117-21.

707. Biswas G, Nagamine R, Hikima JI, Sakai M, Kono T. Inductive immune responses in the Japanese pufferfish (<i>Takifugu</i> <i>rubripes</i>) treated with recombinant IFN-γ, IFN-γrel, IL-4/13A and IL-4/13B. Int Immunopharmacol. 2016;31:50-6.

708. Chatterjee N, Anwar T, Islam NS, Ramasarma T, Ramakrishna G. Growth arrest of lung carcinoma cells (A549) by polyacrylate-anchored peroxovanadate by activating Rac1-NADPH oxidase signalling axis. Mol Cell Biochem. 2016;420(1-2):9-20.

709. Chatterjee N, Anwar T, Islam NS, Ramasarma T, Ramakrishna G. Growth arrest of lung carcinoma cells (A549) by polyacrylate-anchored peroxovanadate by activating Rac1-NADPH oxidase signalling axis. Mol Cell Biochem. 2016;420(1-2):9-20.

710. Clark R, Lee SH. Anticancer Properties of Capsaicin Against Human Cancer. Anticancer Res. 2016;36(3):837-43.

711. Coombs MRP, Harrison ME, Hoskin DW. Apigenin inhibits the inducible expression of programmed death ligand 1 by human and mouse mammary carcinoma cells. Cancer Lett. 2016;380(2):424-33.

712. Coombs MRP, Harrison ME, Hoskin DW. Apigenin inhibits the inducible expression of programmed death ligand 1 by human and mouse mammary carcinoma cells. Cancer Lett. 2016;380(2):424-33.

713. Cragg GM, Pezzuto JM. Natural Products as a Vital Source for the Discovery of Cancer Chemotherapeutic and Chemopreventive Agents. Med Princ Pract. 2016;25 Suppl 2(Suppl 2):41-59.

714. Farhan M, Khan HY, Oves M, Al-Harrasi A, Rehmani N, Arif H, et al. Cancer therapy by catechins involves redox cycling of copper ions and generation of reactive oxygenspecies. Toxins. 2016;8(2).

715. Giuga M, De Gaetano AM, Guerra A, Infante A, Iezzi R, Spinelli I, et al. An update on clinical applications of hepatospecific contrast media in magnetic resonance imaging of liver parenchyma. Eur Rev Med Pharmacol Sci. 2016;20(12):2515-25.

716. Giuga M, De Gaetano AM, Guerra A, Infante A, Iezzi R, Spinelli I, et al. An update on clinical applications of hepatospecific contrast media in magnetic resonance imaging of liver parenchyma. Eur Rev Med Pharmacol Sci. 2016;20(12):2515-25.

717. Hankinson O. The role of AHR-inducible cytochrome P450s in metabolism of polyunsaturated fatty acids. Drug Metabolism Reviews. 2016;48(3):342-50.

718. Hankinson O. The role of AHR-inducible cytochrome P450s in metabolism of polyunsaturated fatty acids. Drug Metab Rev. 2016;48(3):342-50.

719. Hao W, Zhang X, Zhao W, Zhu H, Liu ZY, Lu J, et al. Cryptotanshinone Induces Pro-death Autophagy through JNK Signaling Mediated by Reactive Oxygen Species Generation in Lung Cancer Cells. Anticancer Agents Med Chem. 2016;16(5):593-600.

720. Hao WH, Zhang XN, Zhao WW, Zhu H, Liu ZY, Lu JJ, et al. Cryptotanshinone Induces Pro-death Autophagy through JNK Signaling Mediated by Reactive Oxygen Species Generation in Lung Cancer Cells. Anti-Cancer Agents Med Chem. 2016;16(5):593-600.

721. He Y, Xie Z, Dai J, Cao Y, Hou J, Zheng Y, et al. Responses of the Toll-like receptor and melanoma differentiation-associated protein 5 signaling pathways to avian infectious bronchitis virus infection in chicks. Virol Sin. 2016;31(1):57-68.

722. He YN, Xie ZW, Dai JL, Cao YJ, Hou JL, Zheng YS, et al. Responses of the Toll-like receptor and melanoma differentiation-associated protein 5 signaling pathways to avian infectious bronchitis virus infection in chicks. Virol Sin. 2016;31(1):57-68.

723. Ho PY, Byadgi O, Wang PC, Tsai MA, Liaw LL, Chen SC. Identification, molecular cloning of IL-1β and its expression profile during Nocardia seriolae infection in largemouth bass, Micropterus salmoides. Int J Mol Sci. 2016;17(10).

724. Ho PY, Byadgi O, Wang PC, Tsai MA, Liaw LL, Chen SC. Identification, Molecular Cloning of IL-1β and Its Expression Profile during Nocardia seriolae Infection in Largemouth Bass, Micropterus salmoides. Int J Mol Sci. 2016;17(10).

725. Ismail T, Calcabrini C, Diaz AR, Fimognari C, Turrini E, Catanzaro E, et al. Ellagitannins in cancer chemoprevention and therapy. Toxins. 2016;8(5).

726. Jiang X, Zhang Z, Zhao N, He X, Guo H. Effects of Platelet-Derived Endothelial Cell Growth Factor and Doppler Perfusion Index in Patients with Colorectal Hepatic Metastases. Visc Med. 2016;32(3):206-10.

727. Jiang XM, Zhang ZX, Zhao N, He XH, Guo H. Effects of Platelet-Derived Endothelial Cell Growth Factor and Doppler Perfusion Index in Patients with Colorectal Hepatic Metastases. Visc Med. 2016;32(3):206-10.

728. Jung JS, Ahn YH, Moon BI, Kim HS. Exogenous C2 ceramide suppresses matrix metalloproteinase gene expression by inhibiting ROS production and MAPK signaling pathways in PMA-stimulated human astroglioma cells. Int J Mol Sci. 2016;17(4).

729. Katiyar SK. Dietary proanthocyanidins inhibit UV radiation-induced skin tumor development through functional activation of the immune system. Mol Nutr Food Res. 2016;60(6):1374-82.

730. Kim JM, Ko H, Kim SJ, Shim SH, Ha CH, Chang HI. Chemopreventive Properties of Genipin on AGS Cell Line via Induction of JNK/Nrf2/ARE Signaling Pathway. Journal of Biochemical and Molecular Toxicology. 2016;30(1):45-54.

731. Kotecha R, Takami A, Espinoza JL. Dietary phytochemicals and cancer chemoprevention: A review of the clinical evidence. Oncotarget. 2016;7(32):52517-29.

732. Levet V, Rosière R, Merlos R, Fusaro L, Berger G, Amighi K, et al. Development of controlled-release cisplatin dry powders for inhalation against lung cancers. Int J Pharm. 2016;515(1-2):209-20.

733. Levet V, Rosière R, Merlos R, Fusaro L, Berger G, Amighi K, et al. Development of controlled-release cisplatin dry powders for inhalation against lung cancers. Int J Pharm. 2016;515(1-2):209-20.

734. Li S, Chen G, Wu M, Zhang J, Wu S. Restraining of reactive oxygen species promotes invasion of Listeria monocytogenes into glia cells. FEMS Microbiol Lett. 2016;363(2):fnv228.

735. Li W, Guo Y, Zhang C, Wu R, Yang AY, Gaspar J, et al. Dietary Phytochemicals and Cancer Chemoprevention: A Perspective on Oxidative Stress, Inflammation, and Epigenetics. Chem Res Toxicol. 2016;29(12):2071-95.

736. Lin CC, Lin WN, Cho RL, Wang CY, Hsiao LD, Yang CM. TNF-α-Induced cPLA(2) Expression via NADPH Oxidase/Reactive Oxygen Species-Dependent NF-κB Cascade on Human Pulmonary Alveolar Epithelial Cells. Front Pharmacol. 2016;7:447.

737. Lin HR, Wu YH, Yen WC, Yang CM, Chiu DT. Diminished COX-2/PGE2-Mediated Antiviral Response Due to Impaired NOX/MAPK Signaling in G6PD-Knockdown Lung Epithelial Cells. PLoS One. 2016;11(4):e0153462.

738. Lin HR, Wu YH, Yen WC, Yang CM, Chiu DTY. Diminished COX-2/PGE2-mediated antiviral response due to impaired NOX/MAPK signaling in G6PD-knockdown lung epithelial cells. PLoS One. 2016;11(4).

739. Liu N, Yang HL, Wang P, Lu YC, Yang YJ, Wang L, et al. Functional proteomic analysis revels that the ethanol extract of Annona muricata L. induces liver cancer cell apoptosis through endoplasmic reticulum stress pathway. J Ethnopharmacol. 2016;189:210-7.

740. Liu N, Yang HL, Wang P, Lu YC, Yang YJ, Wang L, et al. Functional proteomic analysis revels that the ethanol extract of Annona muricata L. induces liver cancer cell apoptosis through endoplasmic reticulum stress pathway. J Ethnopharmacol. 2016;189:210-7.

741. Liu P, Qi CB, Zhu QF, Yuan BF, Feng YQ. Determination of thiol metabolites in human urine by stable isotope labeling in combination with pseudo-targeted mass spectrometry analysis. Sci Rep. 2016;6.

742. Liu P, Qi CB, Zhu QF, Yuan BF, Feng YQ. Determination of thiol metabolites in human urine by stable isotope labeling in combination with pseudo-targeted mass spectrometry analysis. Sci Rep. 2016;6:21433.

743. Liu XL, Shan WJ, Jia LJ, Yang X, Zhang JJ, Wu YR, et al. Avian leukosis virus subgroup J triggers caspase-1-mediated inflammatory response in chick livers. Virus Res. 2016;215:65-71.

744. Megna BW, Carney PR, Nukaya M, Geiger P, Kennedy GD. Indole-3-carbinol induces tumor cell death: Function follows form. Journal of Surgical Research. 2016;204(1):47-54.

745. Mergia E, Shibeshi W, Terefe G, Teklehaymanot T. Antitrypanosomal activity of Verbascum sinaiticum Benth. (Scrophulariaceae) against Trypanosoma congolense isolates. BMC Complement Altern Med. 2016;16:362.

746. Mergia E, Shibeshi W, Terefe G, Teklehaymanot T. Antitrypanosomal activity of <i>Verbascum sinaiticum</i> Benth. (Scrophulariaceae) against <i>Trypanosoma congolense</i> isolates. BMC Complement Altern Med. 2016;16:9.

747. Mindova S, Ivanovski K, Pesevska S, Pandilova M, Georgieva S, Stefanovska E, et al. Effects of smoking to the lipid profile in patients with periodontal disease. Research Journal of Pharmaceutical, Biological and Chemical Sciences. 2016;7(3):2001-9.

748. Moga MA, Dimienescu OG, Arvatescu CA, Mironescu A, Dracea L, Ples L. The role of natural polyphenols in the prevention and treatment of cervical cancer - An overview. Molecules. 2016;21(8).

749. Mohana S, Ganesan M, Agilan B, Karthikeyan R, Srithar G, Beaulah Mary R, et al. Screening dietary flavonoids for the reversal of P-glycoprotein-mediated multidrug resistance in cancer. Molecular BioSystems. 2016;12(8):2458-70.

750. Momtazi AA, Sahebkar A. Difluorinated curcumin: A promising curcumin analogue with improved anti-tumor activity and pharmacokinetic profile. Curr Pharm Design. 2016;22(28):4386-97.

751. Momtazi AA, Sahebkar A. Difluorinated Curcumin: A Promising Curcumin Analogue with Improved Anti-Tumor Activity and Pharmacokinetic Profile. Curr Pharm Des. 2016;22(28):4386-97.

752. Oh J, Hlatky L, Jeong YS, Kim D. Therapeutic effectiveness of anticancer phytochemicals on cancer stem cells. Toxins. 2016;8(7).

753. Pajari AM, Päivärinta E, Paavolainen L, Vaara E, Koivumäki T, Garg R, et al. Ellagitannin-rich cloudberry inhibits hepatocyte growth factorinduced cell migration and phosphatidylinositol 3-kinase/AKT activation in colon carcinoma cells and tumors in Min mice. Oncotarget. 2016;7(28):43907-23.

754. Pérez D, Muñoz MC, Molina JM, Muñoz-Caro T, Silva LMR, Taubert A, et al. Eimeria ninakohlyakimovae induces NADPH oxidase-dependent monocyte extracellular trap formation and upregulates IL-12 and TNF-α, IL-6 and CCL2 gene transcription. Veterinary Parasitology. 2016;227:143-50.

755. Pierzchalska M, Grabacka M. The potential role of some phytochemicals in recognition of mitochondrial damage-associated molecular patterns. Mitochondrion. 2016;30:24-34.

756. Qin S, Hou DX. Multiple regulations of Keap1/Nrf2 system by dietary phytochemicals. Mol Nutr Food Res. 2016;60(8):1731-55.

757. Ran X, Zhao W, Li W, Shi J, Chen X. Cryptotanshinone inhibits TNF-α-induced LOX-1 expression by suppressing reactive oxygen species (ROS) formation in endothelial cells. Korean J Physiol Pharmacol. 2016;20(4):347-55.

758. Ran XL, Zhao WW, Li WP, Shi JS, Chen XP. Cryptotanshinone inhibits TNF-α-induced LOX-1 expression by suppressing reactive oxygen species (ROS) formation in endothelial cells. Korean J Physiol Pharmacol. 2016;20(4):347-55.

759. Ren G, Luo W, Sun W, Niu Y, Ma DL, Leung CH, et al. Psoralidin induced reactive oxygen species (ROS)-dependent DNA damage and protective autophagy mediated by NOX4 in breast cancer cells. Phytomedicine. 2016;23(9):939-47.

760. Ren G, Luo W, Sun W, Niu Y, Ma DL, Leung CH, et al. Psoralidin induced reactive oxygen species (ROS)-dependent DNA damage and protective autophagy mediated by NOX4 in breast cancer cells. Phytomedicine. 2016;23(9):939-47.

761. Saxena R, Rida PC, Kucuk O, Aneja R. Ginger augmented chemotherapy: A novel multitarget nontoxic approach for cancer management. Mol Nutr Food Res. 2016;60(6):1364-73.

762. Shankar E, Kanwal R, Candamo M, Gupta S. Dietary phytochemicals as epigenetic modifiers in cancer: Promise and challenges. Semin Cancer Biol. 2016;40-41:82-99.

763. Thomas NS, George K, Namasivayam N. Molecular aspects and chemoprevention of dimethylaminoazobenzene-induced hepatocarcinogenesis: A review. Hepatology Research. 2016;46(1):72-88.

764. Vitale P, Panella A, Scilimati A, Perrone MG. COX-1 Inhibitors: Beyond Structure Toward Therapy. Medicinal Research Reviews. 2016;36(4):641-71.

765. Zhang L, Huang Y, Zhuo W, Zhu Y, Zhu B, Chen Z. Fisetin, a dietary phytochemical, overcomes erlotinib-resistance of lung adenocarcinoma cells through inhibition of MAPK and AKT pathways. Am J Transl Res. 2016;8(11):4857-68.

766. Zhang L, Huang Y, Zhuo W, Zhu Y, Zhu B, Chen Z. Fisetin, a dietary phytochemical, overcomes Erlotinib-resistance of lung adenocarcinoma cells through inhibition of MAPK and AKT pathways. Am J Transl Res. 2016;8(11):4857-68.

767. Zheng K, Li Y, Wang S, Wang X, Liao C, Hu X, et al. Inhibition of autophagosome-lysosome fusion by ginsenoside Ro via the ESR2-NCF1-ROS pathway sensitizes esophageal cancer cells to 5-fluorouracil-induced cell death via the CHEK1-mediated DNA damage checkpoint. Autophagy. 2016;12(9):1593-613.

768. Zheng K, Li Y, Wang S, Wang X, Liao C, Hu X, et al. Inhibition of autophagosome-lysosome fusion by ginsenoside Ro via the ESR2-NCF1-ROS pathway sensitizes esophageal cancer cells to 5-fluorouracil-induced cell death via the CHEK1-mediated DNA damage checkpoint. Autophagy. 2016;12(9):1593-613.

769. Ahn HS, Han SH, Kim YH, Park BJ, Kim DH, Lee JB, et al. Adverse fetal outcomes in pregnant rabbits experimentally infected with rabbit hepatitis E virus. Virology. 2017;512:187-93.

770. Bakhtiary Z, Barar J, Aghanejad A, Saei AA, Nemati E, Ezzati Nazhad Dolatabadi J, et al. Microparticles containing erlotinib-loaded solid lipid nanoparticles for treatment of non-small cell lung cancer. Drug Dev Ind Pharm. 2017;43(8):1244-53.

771. Bakhtiary Z, Barar J, Aghanejad A, Saei AA, Nemati E, Ezzati Nazhad Dolatabadi J, et al. Microparticles containing erlotinib-loaded solid lipid nanoparticles for treatment of non-small cell lung cancer. Drug Dev Ind Pharm. 2017;43(8):1244-53.

772. Brasili E, Filho VC. Metabolomics of cancer cell cultures to assess the effects of dietary phytochemicals. Crit Rev Food Sci Nutr. 2017;57(7):1328-39.

773. Dong W, Li Z, Chen Y, Zhang L, Ye Z, Liang H, et al. NADPH oxidase inhibitor, diphenyleneiodonium prevents necroptosis in HK-2 cells. Biomed Rep. 2017;7(3):226-30.

774. Filippi A, Ciolac OA, Ganea C, Mocanu MM. ErbB Proteins as Molecular Target of Dietary Phytochemicals in Malignant Diseases. J Oncol. 2017;2017:1532534.

775. Fimognari C. Introduction to the Toxins special issue on dietary and non-dietary phytochemicals and cancer. Toxins. 2017;9(1).

776. Gaballah HH, Gaber RA, Mohamed DA. Apigenin potentiates the antitumor activity of 5-FU on solid Ehrlich carcinoma: Crosstalk between apoptotic and JNK-mediated autophagic cell death platforms. Toxicol Appl Pharmacol. 2017;316:27-35.

777. Heidari M, Delekta PC. Transcriptomic Analysis of Host Immune Response in the Skin of Chickens Infected with Marek's Disease Virus. Viral Immunol. 2017;30(5):377-87.

778. Heidari M, Delekta PC. Transcriptomic Analysis of Host Immune Response in the Skin of Chickens Infected with Marek's Disease Virus. Viral Immunol. 2017;30(5):377-87.

779. Illam SP, Narayanankutty A, Mathew SE, Valsalakumari R, Jacob RM, Raghavamenon AC. Epithelial mesenchymal transition in cancer progression: Preventive phytochemicals. Recent Patents on Anti-Cancer Drug Discovery. 2017;12(3):234-46.

780. Islam MS, Akhtar MM, Segars JH, Castellucci M, Ciarmela P. Molecular targets of dietary phytochemicals for possible prevention and therapy of uterine fibroids: Focus on fibrosis. Crit Rev Food Sci Nutr. 2017;57(17):3583-600.

781. Islam MS, Segars JH, Castellucci M, Ciarmela P. Dietary phytochemicals for possible preventive and therapeutic option of uterine fibroids: Signaling pathways as target. Pharmacol Rep. 2017;69(1):57-70.

782. Islam MS, Segars JH, Castellucci M, Ciarmela P. Dietary phytochemicals for possible preventive and therapeutic option of uterine fibroids: Signaling pathways as target. Pharmacol Rep. 2017;69(1):57-70.

783. Joo HK, Choi S, Lee YR, Lee EO, Park MS, Lim YP, et al. Ethanol Extract of Brassica rapa ssp. pekinensis Suppresses Tumor Necrosis Factor-α-Induced Inflammatory Response in Human Umbilical Vein Endothelial Cells. Journal of medicinal food. 2017;20(5):511-8.

784. Kaneko C, Ogura J, Sasaki S, Okamoto K, Kobayashi M, Kuwayama K, et al. Fructose suppresses uric acid excretion to the intestinal lumen as a result of the induction of oxidative stress by NADPH oxidase activation. Biochimica et Biophysica Acta - General Subjects. 2017;1861(3):559-66.

785. Kaneko C, Ogura J, Sasaki S, Okamoto K, Kobayashi M, Kuwayama K, et al. Fructose suppresses uric acid excretion to the intestinal lumen as a result of the induction of oxidative stress by NADPH oxidase activation. Biochim Biophys Acta Gen Subj. 2017;1861(3):559-66.

786. Kashyap D, Kumar G, Sharma A, Sak K, Tuli HS, Mukherjee TK. Mechanistic insight into carnosol-mediated pharmacological effects: Recent trends and advancements. Life Sci. 2017;169:27-36.

787. Kolanjiyil AV, Kleinstreuer C, Sadikot RT. Computationally efficient analysis of particle transport and deposition in a human whole-lung-airway model. Part II: Dry powder inhaler application. Comput Biol Med. 2017;84:247-53.

788. Kolanjiyil AV, Kleinstreuer C, Sadikot RT. Computationally efficient analysis of particle transport and deposition in a human whole-lung-airway model. Part II: Dry powder inhaler application. Comput Biol Med. 2017;84:247-53.

789. Kumar A, Rimando AM, Levenson AS. Resveratrol and pterostilbene as a microRNA-mediated chemopreventive and therapeutic strategy in prostate cancer. Annals of the New York Academy of Sciences. 2017;1403(1):15-26.

790. Lamovšek J, Stare BG, Pleško IM, Širca S, Urek G. Agrobacteria enhance plant defense against root-knot nematodes on tomato. Phytopathology. 2017;107(6):681-91.

791. Lan X, Wang Y, Tian K, Ye F, Yin H, Zhao X, et al. Integrated host and viral transcriptome analyses reveal pathology and inflammatory response mechanisms to ALV-J injection in SPF chickens. Sci Rep. 2017;7:46156.

792. Lan X, Wang Y, Tian K, Ye F, Yin H, Zhao X, et al. Integrated host and viral transcriptome analyses reveal pathology and inflammatory response mechanisms to ALV-J injection in SPF chickens. Sci Rep. 2017;7.

793. Lee Y. Cancer chemopreventive potential of procyanidin. Toxicological Research. 2017;33(4):273-82.

794. Levet V, Merlos R, Rosière R, Amighi K, Wauthoz N. Platinum pharmacokinetics in mice following inhalation of cisplatin dry powders with different release and lung retention properties. Int J Pharm. 2017;517(1-2):359-72.

795. Levet V, Merlos R, Rosière R, Amighi K, Wauthoz N. Platinum pharmacokinetics in mice following inhalation of cisplatin dry powders with different release and lung retention properties. Int J Pharm. 2017;517(1-2):359-72.

796. Lin Q, Liu G, Zhao Z, Wei D, Pang J, Jiang Y. Design of gefitinib-loaded poly (L-lactic acid) microspheres via a supercritical anti-solvent process for dry powder inhalation. Int J Pharm. 2017;532(1):573-80.

797. Lin Q, Liu G, Zhao Z, Wei D, Pang J, Jiang Y. Design of gefitinib-loaded poly (l-lactic acid) microspheres via a supercritical anti-solvent process for dry powder inhalation. Int J Pharm. 2017;532(1):573-80.

798. Liu P, Atkinson SJ, Akbareian SE, Zhou Z, Munsterberg A, Robinson SD, et al. Sulforaphane exerts anti-angiogenesis effects against hepatocellular carcinoma through inhibition of STAT3/HIF-1α/VEGF signalling. Sci Rep. 2017;7(1):12651.

799. Liu P, Atkinson SJ, Akbareian SE, Zhou ZG, Munsterberg A, Robinson SD, et al. Sulforaphane exerts anti-angiogenesis effects against hepatocellular carcinoma through inhibition of STAT3/HIF-1α/VEGF signalling. Sci Rep. 2017;7:11.

800. Lu J, Risbood P, Kane CT, Jr., Hossain MT, Anderson L, Hill K, et al. Characterization of potent and selective iodonium-class inhibitors of NADPH oxidases. Biochem Pharmacol. 2017;143:25-38.

801. Lu JM, Risbood P, Kane CT, Hossain MT, Anderson L, Hill K, et al. Characterization of potent and selective iodonium-class inhibitors of NADPH oxidases. Biochem Pharmacol. 2017;143:25-38.

802. Mahbouli S, Der Vartanian A, Ortega S, Rougé S, Vasson MP, Rossary A. Leptin induces ROS via NOX5 in healthy and neoplastic mammary epithelial cells. Oncol Rep. 2017;38(5):3254-64.

803. Mahbouli S, Der Vartanian A, Ortega S, Rougé S, Vasson MP, Rossary A. Leptin induces ROS via NOX5 in healthy and neoplastic mammary epithelial cells. Oncol Rep. 2017;38(5):3254-64.

804. Mayoral M, Paredes P, Domènech B, Fusté P, Vidal-Sicart S, Tapias A, et al. 18F-FDG PET/CT and sentinel lymph node biopsy in the staging of patients with cervical and endometrial cancer. Role of dual-time-point imaging. Rev Esp Med Nucl Imagen Mol. 2017;36(1):20-6.

805. McDougall GJ, Allwood JW, Pereira-Caro G, Brown EM, Verrall S, Stewart D, et al. Novel colon-available triterpenoids identified in raspberry fruits exhibit antigenotoxic activities in vitro. Mol Nutr Food Res. 2017;61(2).

806. Naseri N, Zakeri-Milani P, Hamishehkar H, Pilehvar-Soltanahmadi Y, Valizadeh H. Development, in Vitro Characterization, Antitumor and Aerosol Performance Evaluation of Respirable Prepared by Self-nanoemulsification Method. Drug Research. 2017;67(6):343-8.

807. Ninfali P, Antonini E, Frati A, Scarpa ES. C-Glycosyl Flavonoids from Beta vulgaris Cicla and Betalains from Beta vulgaris rubra: Antioxidant, Anticancer and Antiinflammatory Activities—A Review. Phytotherapy Research. 2017;31(6):871-84.

808. Nishimura M, Umeda K, Suwa M, Furuoka H, Nishikawa Y. CCR5 is involved in interruption of pregnancy in mice infected with Toxoplasma gondii during early pregnancy. Infect Immun. 2017;85(9).

809. Nishimura M, Umeda K, Suwa M, Furuoka H, Nishikawa Y. CCR5 Is Involved in Interruption of Pregnancy in Mice Infected with Toxoplasma gondii during Early Pregnancy. Infect Immun. 2017;85(9).

810. Östreicher C, Bartenbacher S, Pischetsrieder M. Targeted proteome analysis with isotope-coded protein labels for monitoring the influence of dietary phytochemicals on the expression of cytoprotective proteins in primary human colon cells. Journal of Proteomics. 2017;166:27-38.

811. Ozsvari B, Bonuccelli G, Sanchez-Alvarez R, Foster R, Sotgia F, Lisanti MP. Targeting flavin-containing enzymes eliminates cancer stem cells (CSCs), by inhibiting mitochondrial respiration: Vitamin B2 (Riboflavin) in cancer therapy. Aging. 2017;9(12):2610-28.

812. Ozsvari B, Bonuccelli G, Sanchez-Alvarez R, Foster R, Sotgia F, Lisanti MP. Targeting flavin-containing enzymes eliminates cancer stem cells (CSCs), by inhibiting mitochondrial respiration: Vitamin B2 (Riboflavin) in cancer therapy. Aging (Albany NY). 2017;9(12):2610-28.

813. Park EJ, Kim YM, Chang KC. Hemin Reduces HMGB1 Release by UVB in an AMPK/HO-1-dependent Pathway in Human Keratinocytes HaCaT Cells. Arch Med Res. 2017;48(5):423-31.

814. Park EJ, Kim YM, Chang KC. Hemin Reduces HMGB1 Release by UVB in an AMPK/HO-1-dependent Pathway in Human Keratinocytes HaCaT Cells. Arch Med Res. 2017;48(5):423-31.

815. Park JE, Sun Y, Lim SK, Tam JP, Dekker M, Chen H, et al. Dietary phytochemical PEITC restricts tumor development via modulation of epigenetic writers and erasers. Sci Rep. 2017;7.

816. Park JE, Sun Y, Lim SK, Tam JP, Dekker M, Chen H, et al. Dietary phytochemical PEITC restricts tumor development via modulation of epigenetic writers and erasers. Sci Rep. 2017;7:40569.

817. Park JM, Han YM, Jeong M, Chung MH, Kwon CI, Ko KH, et al. Synthetic 8-hydroxydeoxyguanosine inhibited metastasis of pancreatic cancer through concerted inhibitions of ERM and Rho-GTPase. Free Radic Biol Med. 2017;110:151-61.

818. Park JM, Han YM, Jeong M, Chung MH, Kwon CI, Ko KH, et al. Synthetic 8-hydroxydeoxyguanosine inhibited metastasis of pancreatic cancer through concerted inhibitions of ERM and Rho-GTPase. Free Radic Biol Med. 2017;110:151-61.

819. Probst YC, Guan VX, Kent K. Dietary phytochemical intake from foods and health outcomes: a systematic review protocol and preliminary scoping. BMJ Open. 2017;7(2):e013337.

820. Probst YC, Guan VX, Kent K. Dietary phytochemical intake from foods and health outcomes: a systematic review protocol and preliminary scoping. BMJ Open. 2017;7(2):9.

821. Rai G, Suman S, Mishra S, Shukla Y. Evaluation of growth inhibitory response of Resveratrol and Salinomycin combinations against triple negative breast cancer cells. Biomed Pharmacother. 2017;89:1142-51.

822. Rai G, Suman S, Mishra S, Shukla Y. Evaluation of growth inhibitory response of Resveratrol and Salinomycin combinations against triple negative breast cancer cells. Biomed Pharmacother. 2017;89:1142-51.

823. Ramirez CN, Li W, Zhang C, Wu R, Su S, Wang C, et al. In Vitro-In Vivo Dose Response of Ursolic Acid, Sulforaphane, PEITC, and Curcumin in Cancer Prevention. Aaps j. 2017;20(1):19.

824. Sayeed MA, Bracci M, Lucarini G, Lazzarini R, Di Primio R, Santarelli L. Regulation of microRNA using promising dietary phytochemicals: Possible preventive and treatment option of malignant mesothelioma. Biomedicine and Pharmacotherapy. 2017;94:1197-224.

825. Singh AK, Sharma N, Ghosh M, Park YH, Jeong DK. Emerging importance of dietary phytochemicals in fight against cancer: Role in targeting cancer stem cells. Crit Rev Food Sci Nutr. 2017;57(16):3449-63.

826. Spyridopoulou K, Tiptiri-Kourpeti A, Lampri E, Fitsiou E, Vasileiadis S, Vamvakias M, et al. Dietary mastic oil extracted from Pistacia lentiscus var. chia suppresses tumor growth in experimental colon cancer models. Sci Rep. 2017;7(1).

827. Suman S, Kumar S, Moon BH, Fornace AJ, Kallakury BVS, Datta K. Increased transgenerational intestinal tumorigenesis in offspring of ionizing radiation exposed parent APC1638N/+ mice. J Cancer. 2017;8(10):1769-73.

828. Suman S, Kumar S, Moon BH, Fornace AJ, Jr., Kallakury BVS, Datta K. Increased Transgenerational Intestinal Tumorigenesis in Offspring of Ionizing Radiation Exposed Parent APC(1638N/+) Mice. J Cancer. 2017;8(10):1769-73.

829. Sun G, Zheng Z, Lee MH, Xu Y, Kang S, Dong Z, et al. Chemoprevention of Colorectal Cancer by Artocarpin, a Dietary Phytochemical from Artocarpus heterophyllus. J Agric Food Chem. 2017;65(17):3474-80.

830. Sun GC, Zheng ZP, Lee MH, Xu YJ, Kang S, Dong ZG, et al. Chemoprevention of Colorectal Cancer by Artocarpin, a Dietary Phytochemical from <i>Artocarpus heterophyllus</i>. J Agric Food Chem. 2017;65(17):3474-80.

831. Wang J, Wang L, Ho CT, Zhang K, Liu Q, Zhao H. Garcinol from Garcinia indica Downregulates Cancer Stem-like Cell Biomarker ALDH1A1 in Nonsmall Cell Lung Cancer A549 Cells through DDIT3 Activation. J Agric Food Chem. 2017;65(18):3675-83.

832. Wang J, Wang L, Ho CT, Zhang K, Liu Q, Zhao H. Garcinol from Garcinia indica Downregulates Cancer Stem-like Cell Biomarker ALDH1A1 in Nonsmall Cell Lung Cancer A549 Cells through DDIT3 Activation. J Agric Food Chem. 2017;65(18):3675-83.

833. Zhao W, Feng H, Sun W, Liu K, Lu JJ, Chen X. Tert-butyl hydroperoxide (t-BHP) induced apoptosis and necroptosis in endothelial cells: Roles of NOX4 and mitochondrion. Redox Biol. 2017;11:524-34.

834. Akhtar MH, Hussain KK, Gurudatt NG, Chandra P, Shim YB. Ultrasensitive dual probe immunosensor for the monitoring of nicotine induced-brain derived neurotrophic factor released from cancer cells. Biosens Bioelectron. 2018;116:108-15.

835. Akhtar MH, Hussain KK, Gurudatt NG, Chandra P, Shim YB. Ultrasensitive dual probe immunosensor for the monitoring of nicotine induced-brain derived neurotrophic factor released from cancer cells. Biosens Bioelectron. 2018;116:108-15.

836. Alves CRR, Faria DDP, Carneiro CDG, Garcez AT, Gutierrez VP, das Neves W, et al. 18F-Fluoride PET/CT and 99mTc-MDP SPECT/CT can detect bone cancer at early stage in rodents. Life Sci. 2018;206:29-34.

837. Amadori M, Barone D, Scarpi E, Oboldi D, Amadori E, Bandi G, et al. Dynamic contrast-enhanced ultrasonography (D-CEUS) for the early prediction of bevacizumab efficacy in patients with metastatic colorectal cancer. Eur Radiol. 2018;28(7):2969-78.

838. Amadori M, Barone D, Scarpi E, Oboldi D, Amadori E, Bandi G, et al. Dynamic contrast-enhanced ultrasonography (D-CEUS) for the early prediction of bevacizumab efficacy in patients with metastatic colorectal cancer. Eur Radiol. 2018;28(7):2969-78.

839. Arif H, Sohail A, Farhan M, Rehman AA, Ahmad A, Hadi SM. Flavonoids-induced redox cycling of copper ions leads to generation of reactive oxygen species: A potential role in cancer chemoprevention. Int J Biol Macromol. 2018;106:569-78.

840. Avci ME, Keskus AG, Targen S, Isilak ME, Ozturk M, Atalay RC, et al. Development of a novel zebrafish xenograft model in ache mutants using liver cancer cell lines. Sci Rep. 2018;8(1):1570.

841. Avci ME, Keskus AG, Targen S, Isilak ME, Ozturk M, Atalay RC, et al. Development of a novel zebrafish xenograft model in <i>ache</i> mutants using liver cancer cell lines. Sci Rep. 2018;8:14.

842. Bavananthasivam J, Alkie TN, Astill J, Abdul-Careem MF, Wootton SK, Behboudi S, et al. In ovo administration of Toll-like receptor ligands encapsulated in PLGA nanoparticles impede tumor development in chickens infected with Marek's disease virus. Vaccine. 2018;36(28):4070-6.

843. Chan MM, Chen R, Fong D. Targeting cancer stem cells with dietary phytochemical - Repositioned drug combinations. Cancer Lett. 2018;433:53-64.

844. Chan MM, Chen RS, Fong DN. Targeting cancer stem cells with dietary phytochemical - Repositioned drug combinations. Cancer Lett. 2018;433:53-64.

845. Chen G, Li Z, Su S, Chang G, Qiu L, Zhu P, et al. Identification of key genes fluctuated induced by avian leukemia virus (ALV-J) infection in chicken cells. In Vitro Cell Dev Biol Anim. 2018;54(1):41-51.

846. Chen GH, Li ZT, Su S, Chang GB, Qiu LL, Zhu PF, et al. Identification of key genes fluctuated induced by avian leukemia virus (ALV-J) infection in chicken cells. In Vitro Cell Dev Biol-Anim. 2018;54(1):41-51.

847. Chen GW, Wu M, Liu WK, Xie MM, Zhang WS, Fan EG, et al. Reactive oxygen species inhibits Listeria monocytogenes invasion into HepG2 epithelial cells. Food Sci Nutr. 2018;6(6):1501-7.

848. Chen GW, Wu M, Liu WK, Xie MM, Zhang WS, Fan EG, et al. Reactive oxygen species inhibits <i>Listeria monocytogenes</i> invasion into HepG2 epithelial cells. Food Sci Nutr. 2018;6(6):1501-7.

849. Chen H, Liu RH. Potential Mechanisms of Action of Dietary Phytochemicals for Cancer Prevention by Targeting Cellular Signaling Transduction Pathways. J Agric Food Chem. 2018;66(13):3260-76.

850. Chikara S, Nagaprashantha LD, Singhal J, Horne D, Awasthi S, Singhal SS. Oxidative stress and dietary phytochemicals: Role in cancer chemoprevention and treatment. Cancer Lett. 2018;413:122-34.

851. Chiou YS, Li S, Ho CT, Pan MH. Prevention of Breast Cancer by Natural Phytochemicals: Focusing on Molecular Targets and Combinational Strategy. Mol Nutr Food Res. 2018;62(23):e1800392.

852. Eastham LL, Howard CM, Balachandran P, Pasco DS, Claudio PP. Eating Green: Shining Light on the Use of Dietary Phytochemicals as a Modern Approach in the Prevention and Treatment of Head and Neck Cancers. Curr Top Med Chem. 2018;18(3):182-91.

853. Fitsiou E, Mitropoulou G, Spyridopoulou K, Vamvakias M, Bardouki H, Galanis A, et al. Chemical composition and evaluation of the biological properties of the essential oil of the dietary phytochemical Lippia citriodora. Molecules. 2018;23(1).

854. Fitsiou E, Mitropoulou G, Spyridopoulou K, Vamvakias M, Bardouki H, Galanis A, et al. Chemical Composition and Evaluation of the Biological Properties of the Essential Oil of the Dietary Phytochemical <i>Lippia citriodora</i>. Molecules. 2018;23(1):13.

855. Gulei D, Mehterov N, Nabavi SM, Atanasov AG, Berindan-Neagoe I. Targeting ncRNAs by plant secondary metabolites: The ncRNAs game in the balance towards malignancy inhibition. Biotechnology Advances. 2018;36(6):1779-99.

856. Gunasekaran VP, Nishi K, Sivakumar D, Sivaraman T, Mathan G. Identification of 2,4-dihydroxy-5-pyrimidinyl imidothiocarbomate as a novel inhibitor to Y box binding protein-1 (YB-1) and its therapeutic actions against breast cancer. Eur J Pharm Sci. 2018;116:2-14.

857. Gunasekaran VP, Nishi K, Sivakumar D, Sivaraman T, Mathan G. Identification of 2,4-dihydroxy-5-pyrimidinyl imidothiocarbomate as a novel inhibitor to Y box binding protein-1 (YB-1) and its therapeutic actions against breast cancer. Eur J Pharm Sci. 2018;116:2-14.

858. Guo Y, Wu R, Gaspar JM, Sargsyan D, Su ZY, Zhang C, et al. DNA methylome and transcriptome alterations and cancer prevention by curcumin in colitis-accelerated colon cancer in mice. Carcinogenesis. 2018;39(5):669-80.

859. Guo Y, Wu RY, Gaspar JM, Sargsyan D, Su ZY, Zhang CY, et al. DNA methylome and transcriptome alterations and cancer prevention by curcumin in colitis-accelerated colon cancer in mice. Carcinogenesis. 2018;39(5):669-80.

860. Hao L, Kemmenoe DJ, Orel DC, Burr T. The impacts of tumorigenic and nontumorigenic Agrobacterium vitis strains on graft strength and growth of grapevines. Plant Dis. 2018;102(2):375-81.

861. Hao L, Kemmenoe DJ, Orel DC, Burr T. The Impacts of Tumorigenic and Nontumorigenic Agrobacterium vitis Strains on Graft Strength and Growth of Grapevines. Plant Dis. 2018;102(2):375-81.

862. Hori K, Ajioka K, Goda N, Shindo A, Takagishi M, Tenno T, et al. Discovery of potent Disheveled/Dvl inhibitors using virtual screening optimized with NMR-based docking performance index. Front Pharmacol. 2018;9(SEP).

863. Hori K, Ajioka K, Goda N, Shindo A, Takagishi M, Tenno T, et al. Discovery of Potent Disheveled/Dvl Inhibitors Using Virtual Screening Optimized With NMR-Based Docking Performance Index. Front Pharmacol. 2018;9:983.

864. Hsiao SH, Murakami M, Yeh N, Li YQ, Hung TH, Wu YS, et al. The positive inotropic agent DPI-201106 selectively reverses ABCB1-mediated multidrug resistance in cancer cell lines. Cancer Lett. 2018;434:81-90.

865. Huang TH, Huang XL, Shi BM, Wang FF, Feng WZ, Yao M. Regulators of <i>Salmonella</i>-host interaction identified by peripheral blood transcriptome profiling: roles of TGFB1 and TRP53 in intracellular <i>Salmonella</i> replication in pigs. Vet Res. 2018;49:14.

866. Jaman MS, Sayeed MA. Ellagic acid, sulforaphane, and ursolic acid in the prevention and therapy of breast cancer: current evidence and future perspectives. Breast Cancer. 2018;25(5):517-28.

867. Jin X, Su R, Li R, Cheng L, Li Z. Crucial role of pro-inflammatory cytokines from respiratory tract upon PM2.5 exposure in causing the BMSCs differentiation in cells and animals. Oncotarget. 2018;9(2):1745-59.

868. Jin X, Su R, Li R, Cheng L, Li Z. Crucial role of pro-inflammatory cytokines from respiratory tract upon PM(2.5) exposure in causing the BMSCs differentiation in cells and animals. Oncotarget. 2018;9(2):1745-59.

869. Kamiyanagi A, Sumita Y, Ino S, Chikai M, Nakane A, Tohara H, et al. Evaluation of swallowing ability using swallowing sounds in maxillectomy patients. J Oral Rehabil. 2018;45(2):126-31.

870. Kamiyanagi A, Sumita Y, Ino S, Chikai M, Nakane A, Tohara H, et al. Evaluation of swallowing ability using swallowing sounds in maxillectomy patients. J Oral Rehabil. 2018;45(2):126-31.

871. Kapinova A, Kubatka P, Golubnitschaja O, Kello M, Zubor P, Solar P, et al. Dietary phytochemicals in breast cancer research: Anticancer effects and potential utility for effective chemoprevention. Environmental Health and Preventive Medicine. 2018;23(1).

872. Kim TH, Shin S, Yoo SD, Shin BS. Effects of Phytochemical P-Glycoprotein Modulators on the Pharmacokinetics and Tissue Distribution of Doxorubicin in Mice. Molecules. 2018;23(2).

873. Kip E, Staal J, Verstrepen L, Tima HG, Terryn S, Romano M, et al. MALT1 controls attenuated rabies virus by inducing early inflammation and T cell activation in the brain. J Virol. 2018;92(8).

874. Kip E, Staal J, Verstrepen L, Tima HG, Terryn S, Romano M, et al. MALT1 Controls Attenuated Rabies Virus by Inducing Early Inflammation and T Cell Activation in the Brain. J Virol. 2018;92(8).

875. Kitamoto K, Miura Y, Karnan S, Ota A, Konishi H, Hosokawa Y, et al. Inhibition of NADPH oxidase 2 induces apoptosis in osteosarcoma: The role of reactive oxygen species in cell proliferation. Oncol Lett. 2018;15(5):7955-62.

876. Kitamoto K, Miura Y, Karnan S, Ota A, Konishi H, Hosokawa Y, et al. Inhibition of NADPH oxidase 2 induces apoptosis in osteosarcoma: The role of reactive oxygen species in cell proliferation. Oncol Lett. 2018;15(5):7955-62.

877. Knickle A, Fernando W, Greenshields AL, Rupasinghe HPV, Hoskin DW. Myricetin-induced apoptosis of triple-negative breast cancer cells is mediated by the iron-dependent generation of reactive oxygen species from hydrogen peroxide. Food Chem Toxicol. 2018;118:154-67.

878. Knickle A, Fernando W, Greenshields AL, Rupasinghe HPV, Hoskin DW. Myricetin-induced apoptosis of triple-negative breast cancer cells is mediated by the iron-dependent generation of reactive oxygen species from hydrogen peroxide. Food Chem Toxicol. 2018;118:154-67.

879. Kume A, Kasai S, Furuya H, Suzuki H. α-Tocopheryl succinate-suppressed development of cerebral malaria in mice. Parasitol Res. 2018;117(10):3177-82.

880. Kume A, Kasai S, Furuya H, Suzuki H. α-Tocopheryl succinate-suppressed development of cerebral malaria in mice. Parasitol Res. 2018;117(10):3177-82.

881. Lee PS, Chiou YS, Ho CT, Pan MH. Chemoprevention by resveratrol and pterostilbene: Targeting on epigenetic regulation. Biofactors. 2018;44(1):26-35.

882. Liao YF, Yin S, Chen ZQ, Li F, Zhao B. High glucose promotes tumor cell proliferation and migration in lung adenocarcinoma via the RAGE‑NOXs pathway. Mol Med Rep. 2018;17(6):8536-41.

883. Liao YF, Yin S, Chen ZQ, Li F, Zhao B. High glucose promotes tumor cell proliferation and migration in lung adenocarcinoma via the RAGE-NOXs pathway. Mol Med Rep. 2018;17(6):8536-41.

884. Liu P, Behray M, Wang Q, Wang W, Zhou Z, Chao Y, et al. Anti-cancer activities of allyl isothiocyanate and its conjugated silicon quantum dots. Sci Rep. 2018;8(1).

885. Liu P, Behray M, Wang Q, Wang W, Zhou Z, Chao Y, et al. Anti-cancer activities of allyl isothiocyanate and its conjugated silicon quantum dots. Sci Rep. 2018;8(1):1084.

886. Lubecka K, Kaufman-Szymczyk A, Cebula-Obrzut B, Smolewski P, Szemraj J, Fabianowska-Majewska K. Novel clofarabine-based combinations with polyphenols epigenetically reactivate retinoic acid receptor beta, inhibit cell growth, and induce apoptosis of breast cancer cells. Int J Mol Sci. 2018;19(12).

887. Manangeeswaran M, Kielczewski JL, Sen HN, Xu BC, Ireland DDC, McWilliams IL, et al. ZIKA virus infection causes persistent chorioretinal lesions article. Emerging Microbes and Infections. 2018;7(1).

888. Manangeeswaran M, Kielczewski JL, Sen HN, Xu BC, Ireland DDC, McWilliams IL, et al. ZIKA virus infection causes persistent chorioretinal lesions. Emerg Microbes Infect. 2018;7(1):96.

889. Martin SL, Royston KJ, Tollefsbol TO. The Role of Non-Coding RNAs and Isothiocyanates in Cancer. Molecular Nutrition and Food Research. 2018;62(18).

890. Mo ZX, Guo JQ, She D, Zhang X, Puthiyakunnon S, Chen XG, et al. Infection by the nematode Angiostrongylus cantonensis induces differential expression of miRNAs in mouse brain. Journal of Microbiology, Immunology and Infection. 2018;51(1):94-102.

891. Okuda T, Morishita M, Mizutani K, Shibayama A, Okazaki M, Okamoto H. Development of spray-freeze-dried siRNA/PEI powder for inhalation with high aerosol performance and strong pulmonary gene silencing activity. J Control Release. 2018;279:99-113.

892. Okuda T, Morishita M, Mizutani K, Shibayama A, Okazaki M, Okamoto H. Development of spray-freeze-dried siRNA/PEI powder for inhalation with high aerosol performance and strong pulmonary gene silencing activity. J Control Release. 2018;279:99-113.

893. Pacheco IL, Abril N, Zafra R, Molina-Hernández V, Morales-Prieto N, Bautista MJ, et al. Fasciola hepatica induces Foxp3 T cell, proinflammatory and regulatory cytokine overexpression in liver from infected sheep during early stages of infection. Vet Res. 2018;49(1).

894. Phan MAT, Paterson J, Bucknall M, Arcot J. Interactions between phytochemicals from fruits and vegetables: Effects on bioactivities and bioavailability. Crit Rev Food Sci Nutr. 2018;58(8):1310-29.

895. Rather RA, Bhagat M. Cancer chemoprevention and piperine: Molecular mechanisms and therapeutic opportunities. Front Cell Dev Biol. 2018;6(FEB).

896. Rather RA, Bhagat M. Cancer Chemoprevention and Piperine: Molecular Mechanisms and Therapeutic Opportunities. Front Cell Dev Biol. 2018;6:10.

897. Rawat D, Shrivastava S, Naik RA, Chhonker SK, Mehrotra A, Koiri RK. An overview of natural plant products in the treatment of hepatocellular carcinoma. Anti-Cancer Agents Med Chem. 2018;18(13):1838-59.

898. Shao C, Liu Y, Qi J, Su Y, Chen Y, Xu H, et al. Real-time detection of the interaction between alpha-fetoprotein and its ssDNA aptamer by dual polarization interferometry. New J Chem. 2018;42(24):19564-70.

899. Shin B, Park W. Zoonotic diseases and phytochemical medicines for microbial infections in veterinary science: current state and future perspective. Front Vet Sci. 2018;5.

900. Smeriglio A, Denaro M, Trombetta D. Dietary phytochemicals and endrocrine-related activities: An update. Mini-Reviews in Medicinal Chemistry. 2018;18(16):1382-97.

901. Sultana S, Dey R, Bishayi B. Dual neutralization of TNFR-2 and MMP-2 regulates the severity of S. aureus induced septic arthritis correlating alteration in the level of interferon gamma and interleukin-10 in terms of TNFR2 blocking. Immunol Res. 2018;66(1):97-119.

902. Suzuki É Y, Amaro MI, de Almeida GS, Cabral LM, Healy AM, de Sousa VP. Development of a new formulation of roflumilast for pulmonary drug delivery to treat inflammatory lung conditions. Int J Pharm. 2018;550(1-2):89-99.

903. Tang CT, Lin XL, Wu S, Liang Q, Yang L, Gao YJ, et al. NOX4-driven ROS formation regulates proliferation and apoptosis of gastric cancer cells through the GLI1 pathway. Cell Signal. 2018;46:52-63.

904. Tang CT, Lin XL, Wu S, Liang Q, Yang L, Gao YJ, et al. NOX4-driven ROS formation regulates proliferation and apoptosis of gastric cancer cells through the GLI1 pathway. Cell Signal. 2018;46:52-63.

905. Wang C, Shu L, Zhang C, Li W, Wu R, Guo Y, et al. Histone Methyltransferase Setd7 Regulates Nrf2 Signaling Pathway by Phenethyl Isothiocyanate and Ursolic Acid in Human Prostate Cancer Cells. Molecular Nutrition and Food Research. 2018;62(18).

906. Wu Y, Shao Y, Song B, Zhen W, Wang Z, Guo Y, et al. Effects of Bacillus coagulans supplementation on the growth performance and gut health of broiler chickens with Clostridium perfringens-induced necrotic enteritis. J Anim Sci Biotechnol. 2018;9(1).

907. Zheng X, Ma W, Sun R, Yin H, Lin F, Liu Y, et al. Butaselen prevents hepatocarcinogenesis and progression through inhibiting thioredoxin reductase activity. Redox Biol. 2018;14:237-49.

908. Zheng X, Ma W, Sun R, Yin H, Lin F, Liu Y, et al. Butaselen prevents hepatocarcinogenesis and progression through inhibiting thioredoxin reductase activity. Redox Biol. 2018;14:237-49.

909. Zhong Q. Co-Spray Dried Mannitol/Poly(amidoamine)-Doxorubicin Dry-Powder Inhaler Formulations for Lung Adenocarcinoma: Morphology, In Vitro Evaluation, and Aerodynamic Performance. AAPS PharmSciTech. 2018;19(2):531-40.

910. Zhu L, Fu X, Yuan C, Jiang X, Zhang G. Induction of Oxidative DNA Damage in Bovine Herpesvirus 1 Infected Bovine Kidney Cells (MDBK Cells) and Human Tumor Cells (A549 Cells and U2OS Cells). Viruses. 2018;10(8).

911. Zuo Q, Wu R, Xiao X, Yang C, Yang Y, Wang C, et al. The dietary flavone luteolin epigenetically activates the Nrf2 pathway and blocks cell transformation in human colorectal cancer HCT116 cells. J Cell Biochem. 2018;119(11):9573-82.

912. Zuo Q, Wu RY, Xiao X, Yang CZ, Yang YQ, Wang C, et al. The dietary flavone luteolin epigenetically activates the Nrf2 pathway and blocks cell transformation in human colorectal cancer HCT116 cells. J Cell Biochem. 2018;119(11):9573-82.

913. Alvarez-Sala A, Attanzio A, Tesoriere L, Garcia-Llatas G, Barberá R, Cilla A. Apoptotic effect of a phytosterol-ingredient and its main phytosterol (β-sitosterol) in human cancer cell lines. Int J Food Sci Nutr. 2019;70(3):323-34.

914. Aumeeruddy MZ, Mahomoodally MF. Combating breast cancer using combination therapy with 3 phytochemicals: Piperine, sulforaphane, and thymoquinone. Cancer. 2019;125(10):1600-11.

915. Baci D, Gallazzi M, Cascini C, Tramacere M, De Stefano D, Bruno A, et al. Downregulation of Pro-Inflammatory and Pro-Angiogenic Pathways in Prostate Cancer Cells by a Polyphenol-Rich Extract from Olive Mill Wastewater. Int J Mol Sci. 2019;20(2).

916. Bai SC, Xu Q, Li H, Qin YF, Song LC, Wang CG, et al. NADPH Oxidase Isoforms Are Involved in Glucocorticoid-Induced Preosteoblast Apoptosis. Oxidative Med Cell Longev. 2019;2019.

917. Bastos RG, Sears K, Dinkel KD, Knowles DP, Fry LM. Changes in the Molecular and Functional Phenotype of Bovine Monocytes during Theileria parva Infection. Infect Immun. 2019;87(12).

918. Bastos RG, Sears K, Dinkel KD, Knowles DP, Fry LM. Changes in the Molecular and Functional Phenotype of Bovine Monocytes during <i>Theileria parva</i> Infection. Infect Immun. 2019;87(12):14.

919. Chatterjee S, Patra D, Chakraborti U, Sengupta D, Ghosh P, Basu A, et al. Association of p38MAPK-p53-Fas aggregation in S-allyl cysteine mediated regulation of hepatocarcinoma. Environmental Toxicology. 2019;34(8):928-40.

920. Das B, Sarkar N, Bishayee A, Sinha D. Dietary phytochemicals in the regulation of epithelial to mesenchymal transition and associated enzymes: A promising anticancer therapeutic approach. Semin Cancer Biol. 2019;56:196-218.

921. Das M, Kandimalla R, Gogoi B, Dutta KN, Choudhury P, Devi R, et al. Mahanine, A dietary phytochemical, represses mammary tumor burden in rat and inhibits subtype regardless breast cancer progression through suppressing self-renewal of breast cancer stem cells. Pharmacol Res. 2019;146:104330.

922. Das M, Kandimalla R, Gogoi B, Dutta KN, Choudhury P, Devi R, et al. Mahanine, A dietary phytochemical, represses mammary tumor burden in rat and inhibits subtype regardless breast cancer progression through suppressing self-renewal of breast cancer stem cells. Pharmacol Res. 2019;146:13.

923. de Moura MLC, Alvares-Saraiva AM, Pérez EC, Xavier JG, Spadacci-Morena DD, Moysés CRS, et al. Cyclophosphamide Treatment Mimics Sub-Lethal Infections With Encephalitozoon intestinalis in Immunocompromised Individuals. Front Microbiol. 2019;10:2205.

924. de Moura MLC, Alvares-Saraiva AM, Pérez EC, Xavier JG, Spadacci-Morena DD, Moysés CRS, et al. Cyclophosphamide Treatment Mimics Sub-Lethal Infections With <i>Encephalitozoon intestinalis</i> in Immunocompromised Individuals. Front Microbiol. 2019;10:13.

925. Dong K, Chang S, Xie Q, Zhao P, Zhang H. RNA Sequencing revealed differentially expressed genes functionally associated with immunity and tumor suppression during latent phase infection of a vv + MDV in chickens. Sci Rep. 2019;9(1):14182.

926. Dong KZ, Chang S, Xie QM, Zhao P, Zhang HM. RNA Sequencing revealed differentially expressed genes functionally associated with immunity and tumor suppression during latent phase infection of a vv plus MDV in chickens. Sci Rep. 2019;9:14.

927. Fan JJ, Hsu WH, Hung HH, Zhang WJ, Lee YA, Chen KC, et al. Reduction in MnSOD promotes the migration and invasion of squamous carcinoma cells. Int J Oncol. 2019;54(5):1639-50.

928. Fan JJ, Hsu WH, Hung HH, Zhang WJ, Lee YLA, Chen KC, et al. Reduction in MnSOD promotes the migration and invasion of squamous carcinoma cells. Int J Oncol. 2019;54(5):1639-50.

929. Fernando W, Rupasinghe HPV, Hoskin DW. Dietary phytochemicals with anti-oxidant and pro-oxidant activities: A double-edged sword in relation to adjuvant chemotherapy and radiotherapy? Cancer Lett. 2019;452:168-77.

930. Fiorillo M, Sotgia F, Lisanti MP. "Energetic" cancer stem cells (e-CSCs): A new hyper-metabolic and proliferative tumor cell phenotype, driven by mitochondrial energy. Front Oncol. 2019;9(FEB).

931. Fiorillo M, Sotgia F, Lisanti MP. "Energetic" Cancer Stem Cells (e-CSCs): A New Hyper-Metabolic and Proliferative Tumor Cell Phenotype, Driven by Mitochondrial Energy. Front Oncol. 2019;8:15.

932. Gao S, Jiang H, Sun J, Diao Y, Tang Y, Hu J. Integrated analysis of mirna and mrna expression profiles in spleen of specific pathogen-free chicken infected with avian reticuloendotheliosis virus strain snv. Int J Mol Sci. 2019;20(5).

933. Gao S, Wang Z, Jiang H, Sun J, Diao Y, Tang Y, et al. Transcriptional analysis of host responses related to immunity in chicken spleen tissues infected with reticuloendotheliosis virus strain SNV. Infection, Genetics and Evolution. 2019;74.

934. Gao S, Wang ZZ, Jiang H, Sun J, Diao YX, Tang Y, et al. Transcriptional analysis of host responses related to immunity in chicken spleen tissues infected with reticuloendotheliosis virus strain SNV. Infect Genet Evol. 2019;74:10.

935. George K, Thomas NS, Malathi R. Modulatory Effect of Selected Dietary Phytochemicals on Delayed Rectifier K+ Current in Human Prostate Cancer Cells. Journal of Membrane Biology. 2019;252(2-3):195-206.

936. Giudice A, Barbieri A, Bimonte S, Cascella M, Cuomo A, Crispo A, et al. Dissecting the prevention of estrogen-dependent breast carcinogenesis through Nrf2-dependent and independent mechanisms. Onco Targets Ther. 2019;12:4937-53.

937. Gupta MK, Vadde R, Sarojamma V. Curcumin - A Novel Therapeutic Agent in the Prevention of Colorectal Cancer. Curr Drug Metab. 2019;20(12):977-87.

938. Gupta P, Mohammad T, Dahiya R, Roy S, Noman OMA, Alajmi MF, et al. Evaluation of binding and inhibition mechanism of dietary phytochemicals with sphingosine kinase 1: Towards targeted anticancer therapy. Sci Rep. 2019;9(1).

939. Hassan FU, Rehman MS, Khan MS, Ali MA, Javed A, Nawaz A, et al. Curcumin as an Alternative Epigenetic Modulator: Mechanism of Action and Potential Effects. Front Genet. 2019;10:514.

940. Hernandez-Morato I, Tian LK, Montalbano M, Pitman MJ. Expression of trophic factors receptors during reinnervation after recurrent laryngeal nerve injury. Laryngoscope. 2019;129(11):2537-42.

941. Hu H, Wang C, Jin Y, Meng Q, Liu Q, Liu Z, et al. Catalpol Inhibits Homocysteine-induced Oxidation and Inflammation via Inhibiting Nox4/NF-κB and GRP78/PERK Pathways in Human Aorta Endothelial Cells. Inflammation. 2019;42(1):64-80.

942. Huang W, Kabbani N, Brannan TK, Lin MK, Theiss MM, Hamilton JF, et al. Association of a Functional Polymorphism in the CHRFAM7A Gene with Inflammatory Response Mediators and Neuropathic Pain after Spinal Cord Injury. J Neurotrauma. 2019;36(21):3026-33.

943. Huang W, Kabbani N, Brannan TK, Lin MK, Theiss MM, Hamilton JF, et al. Association of a Functional Polymorphism in the CHRFAM7A Gene with Inflammatory Response Mediators and Neuropathic Pain after Spinal Cord Injury. J Neurotrauma. 2019;36(21):3026-33.

944. Islam N, Richard D. Inhaled Micro/Nanoparticulate Anticancer Drug Formulations: An Emerging Targeted Drug Delivery Strategy for Lung Cancers. Curr Cancer Drug Targets. 2019;19(3):162-78.

945. Islam N, Richard D. Inhaled micro/nanoparticulate anticancer drug formulations: An emerging targeted drug delivery strategy for lung cancers. Curr Cancer Drug Targets. 2019;19(3):162-78.

946. Jasek K, Kubatka P, Samec M, Liskova A, Smejkal K, Vybohova D, et al. DNA Methylation Status in Cancer Disease: Modulations by Plant-Derived Natural Compounds and Dietary Interventions. Biomolecules. 2019;9(7).

947. Kanai Y, Kawagishi T, Matsuura Y, Kobayashi T. In Vivo Live Imaging of Oncolytic Mammalian Orthoreovirus Expressing NanoLuc Luciferase in Tumor Xenograft Mice. J Virol. 2019;93(14).

948. Kanai Y, Kawagishi T, Matsuura Y, Kobayashi T. <i>In Vivo</i> Live Imaging of Oncolytic Mammalian Orthoreovirus Expressing NanoLuc Luciferase in Tumor Xenograft Mice. J Virol. 2019;93(14):10.

949. Kedhari Sundaram M, Hussain A, Haque S, Raina R, Afroze N. Quercetin modifies 5′CpG promoter methylation and reactivates various tumor suppressor genes by modulating epigenetic marks in human cervical cancer cells. J Cell Biochem. 2019;120(10):18357-69.

950. Kemler I, Ennis MK, Neuhauser CM, Dingli D. In Vivo Imaging of Oncolytic Measles Virus Propagation with Single-Cell Resolution. Molecular Therapy Oncolytics. 2019;12:68-78.

951. Kemler I, Ennis MK, Neuhauser CM, Dingli D. <i>In Vivo</i> Imaging of Oncolytic Measles Virus Propagation with Single-Cell Resolution. Mol Ther-Oncolytics. 2019;12:68-78.

952. Kim DH, Khan H, Ullah H, Hassan STS, Šmejkal K, Efferth T, et al. MicroRNA targeting by quercetin in cancer treatment and chemoprotection. Pharmacol Res. 2019;147.

953. Kim JS, Jeong K, Murphy JM, Rodriguez YAR, Lim SS. A Quantitative Method to Measure Low Levels of ROS in Nonphagocytic Cells by Using a Chemiluminescent Imaging System. Oxid Med Cell Longev. 2019;2019:1754593.

954. Kim JS, Jeong K, Murphy JM, Rodriguez YAR, Lim STS. A quantitative method to measure low levels of ros in nonphagocytic cells by using a chemiluminescent imaging system. Oxidative Med Cell Longev. 2019;2019.

955. Kozłowska L, Gromadzińska J, Wąsowicz W. Health risk in transport workers Part II. Dietary compounds as modulators of occupational exposure to chemicals. International Journal of Occupational Medicine and Environmental Health. 2019;32(4):441-64.

956. Lee KK, Lee JG, Park CS, Lee SH, Raja N, Yun HS, et al. Bone-targeting carbon dots: effect of nitrogen-doping on binding affinity. RSC Adv. 2019;9(5):2708-17.

957. Lee KK, Lee JG, Park CS, Lee SH, Raja N, Yun HS, et al. Bone-targeting carbon dots: Effect of nitrogen-doping on binding affinity. RSC Adv. 2019;9(5):2708-17.

958. Lee KW, Lee KN, Lillehoj HS, Park JH. Serum concentration of acute phase proteins and cytokines in vaccinated pigs challenged with foot-and-mouth disease virus serotype O. Revista Brasileira de Zootecnia. 2019;48.

959. Li Q, Burrough ER, Gabler NK, Loving CL, Sahin O, Gould SA, et al. A soluble and highly fermentable dietary fiber with carbohydrases improved gut barrier integrity markers and growth performance in F18 ETEC challenged pigs1. J Anim Sci. 2019;97(5):2139-53.

960. Li QY, Burrough ER, Gabler NK, Loving CL, Sahin O, Gould SA, et al. A soluble and highly fermentable dietary fiber with carbohydrases improved gut barrier integrity markers and growth performance in F18 ETEC challenged pigs. J Anim Sci. 2019;97(5):2139-53.

961. Lin CC, Hsiao LD, Cho RL, Yang CM. Co-releasing molecule-2 induces Nrf2/ARE-dependent heme oxygenase-1 expression suppressing TNF-α-induced pulmonary inflammation. Journal of Clinical Medicine. 2019;8(4).

962. Lin M, Xiang D, Chen X, Huo H. Role of Characteristic Components of Humulus lupulus in Promoting Human Health. J Agric Food Chem. 2019;67(30):8291-302.

963. Liskova A, Kubatka P, Samec M, Zubor P, Mlyncek M, Bielik T, et al. Dietary phytochemicals targeting cancer stem cells. Molecules. 2019;24(5).

964. Merla C, Liberti M, Consales C, Denzi A, Apollonio F, Marino C, et al. Evidences of plasma membrane-mediated ROS generation upon ELF exposure in neuroblastoma cells supported by a computational multiscale approach. Biochim Biophys Acta Biomembr. 2019;1861(8):1446-57.

965. Moody TW, Lee L, Iordanskaia T, Ramos-Alvarez I, Moreno P, Boudreau HE, et al. PAC1 regulates receptor tyrosine kinase transactivation in a reactive oxygen species-dependent manner. Peptides. 2019;120.

966. Moody TW, Lee L, Iordanskaia T, Ramos-Alvarez I, Moreno P, Boudreau HE, et al. PAC1 regulates receptor tyrosine kinase transactivation in a reactive oxygen species-dependent manner. Peptides. 2019;120:170017.

967. Moody TW, Lee L, Ramos-Alvarez I, Jensen RT. Neurotensin receptors regulate transactivation of the EGFR and HER2 in a reactive oxygen species-dependent manner. Eur J Pharmacol. 2019;865.

968. Moody TW, Lee L, Ramos-Alvarez I, Jensen RT. Neurotensin receptors regulate transactivation of the EGFR and HER2 in a reactive oxygen species-dependent manner. Eur J Pharmacol. 2019;865:172735.

969. NavaneethaKrishnan S, Rosales JL, Lee KY. ROS-mediated cancer cell killing through dietary phytochemicals. Oxidative Med Cell Longev. 2019;2019.

970. Peng S, Ding H, Fu T, Wang B, Wang W, Zhou J. Savitzky-Golay filter based contrast-enhanced ultrasound quantification in hepatic tumors: Methodology and its correlation with tumor angiogenesis. Clin Hemorheol Microcirc. 2019;73(2):271-82.

971. Peng SY, Ding H, Fu TT, Wang BG, Wang WP, Zhou JZ. Savitzky-Golay filter based contrast-enhanced ultrasound quantification in hepatic tumors: Methodology and its correlation with tumor angiogenesis. Clin Hemorheol Microcirc. 2019;73(2):271-82.

972. Samec M, Liskova A, Kubatka P, Uramova S, Zubor P, Samuel SM, et al. The role of dietary phytochemicals in the carcinogenesis via the modulation of miRNA expression. J Cancer Res Clin Oncol. 2019;145(7):1665-79.

973. Sato T, Takino JI, Nagamine K, Nishio K, Hori T. RASGRP2 Suppresses Apoptosis via Inhibition of ROS Production in Vascular Endothelial Cells. Scientific World Journal. 2019;2019.

974. Shahcheraghi SH, Zangui M, Lotfi M, Ghayour-Mobarhan M, Ghorbani A, Jaliani HZ, et al. Therapeutic potential of curcumin in the treatment of glioblastoma multiforme. Curr Pharm Design. 2019;25(3):333-42.

975. Wang C, Bauckman KA, Ross ASB, Symington JW, Ligon MM, Scholtes G, et al. A non-canonical autophagy-dependent role of the ATG16L1 T300A variant in urothelial vesicular trafficking and uropathogenic Escherichia coli persistence. Autophagy. 2019;15(3):527-42.

976. Xu S, Zhou T, Doh HM, Trinh KR, Catapang A, Lee JT, et al. An HK2 antisense oligonucleotide induces synthetic lethality in HK1-HK2+ multiple myeloma. Cancer Res. 2019;79(10):2748-60.

977. Yadav S, Bhagat SD, Gupta A, Samaiya A, Srivastava A, Shukla S. Dietary-phytochemical mediated reversion of cancer-specific splicing inhibits Warburg effect in head and neck cancer. BMC Cancer. 2019;19(1):1031.

978. Yadav S, Bhagat SD, Gupta A, Samaiya A, Srivastava A, Shukla S. Dietary-phytochemical mediated reversion of cancer-specific splicing inhibits Warburg effect in head and neck cancer. BMC Cancer. 2019;19(1):15.

979. Yin R, Kuo HC, Hudlikar R, Sargsyan D, Li S, Wang L, et al. Gut Microbiota, Dietary Phytochemicals, and Benefits to Human Health. Current Pharmacology Reports. 2019;5(5):332-44.

980. Yin Z, Yang L, Wu F, Fan J, Xu J, Jin Y, et al. Reactive oxygen species-mediated cezanne inactivation by oxidation of its catalytic cysteine residue in hepatocellular carcinoma. Oncol Res. 2019;27(9):1069-77.

981. Yin Z, Yang L, Wu F, Fan J, Xu J, Jin Y, et al. Reactive Oxygen Species-Mediated Cezanne Inactivation by Oxidation of its Catalytic Cysteine Residue in Hepatocellular Carcinoma. Oncol Res. 2019;27(9):1069-77.

982. Zhuang J, Zang N, Ye C, Xu F. Lethal avian influenza A (H5N1) virus replicates in pontomedullary chemosensitive neurons and depresses hypercapnic ventilatory response in mice. American Journal of Physiology - Lung Cellular and Molecular Physiology. 2019;316(3):L525-L36.

983. Afrin S, Giampieri F, Gasparrini M, Forbes-Hernández TY, Cianciosi D, Reboredo-Rodriguez P, et al. Dietary phytochemicals in colorectal cancer prevention and treatment: A focus on the molecular mechanisms involved. Biotechnol Adv. 2020;38:107322.

984. Aghababayan S, Sheikhi Mobarakeh Z, Qorbani M, Abbasvandi F, Tiznobeyk Z, Aminianfar A, et al. Dietary Phytochemical Index and Benign Breast Diseases: A Case–Control Study. Nutrition and Cancer. 2020;72(6):1067-73.

985. Aghababayan S, Sheikhi Mobarakeh Z, Qorbani M, Abbasvandi F, Tiznobeyk Z, Aminianfar A, et al. Dietary Phytochemical Index and Benign Breast Diseases: A Case-Control Study. Nutr Cancer. 2020;72(6):1067-73.

986. Al-Ishaq RK, Overy AJ, Büsselberg D. Phytochemicals and Gastrointestinal Cancer: Cellular Mechanisms and Effects to Change Cancer Progression. Biomolecules. 2020;10(1).

987. Albertson TE, Chenoweth JA, Pearson SJ, Murin S. The pharmacological management of asthma-chronic obstructive pulmonary disease overlap syndrome (ACOS). Expert Opin Pharmacother. 2020;21(2):213-31.

988. Asadi A, Nezhad DY, Javazm AR, Khanicheragh P, Mashouri L, Shakeri F, et al. In Vitro Effects of Curcumin on Transforming Growth Factor-β-mediated Non-Smad Signaling Pathway, Oxidative Stress, and Pro-inflammatory Cytokines Production with Human Vascular Smooth Muscle Cells. Iran J Allergy Asthma Immunol. 2020;19(1):84-93.

989. Asadi A, Yaghobi Nezhad D, Rafie Javazm A, Khanicheragh P, Mashouri L, Shakeri F, et al. In Vitro Effects of Curcumin on Transforming Growth Factor-β-mediated Non-Smad Signaling Pathway, Oxidative Stress, and Pro-inflammatory Cytokines Production with Human Vascular Smooth Muscle Cells. Iran J Allergy Asthma Immunol. 2020;19(1):84-93.

990. Becker SL, Li Q, Burrough ER, Kenne D, Sahin O, Gould SA, et al. Effects of an F18 enterotoxigenic Escherichia coli challenge on growth performance, immunological status, and gastrointestinal structure of weaned pigs and the potential protective effect of direct-fed microbial blends. J Anim Sci. 2020;98(5).

991. Becker SL, Li Q, Burrough ER, Kenne D, Sahin O, Gould SA, et al. Effects of an F18 enterotoxigenic Escherichia coli challenge on growth performance, immunological status, and gastrointestinal structure of weaned pigs and the potential protective effect of direct-fed microbial blends. J Anim Sci. 2020;98(5).

992. Bhattacharjee S, Dashwood RH. Epigenetic Regulation of NRF2/KEAP1 by Phytochemicals. Antioxidants (Basel). 2020;9(9).

993. Cao S, Zhu C, Feng J, Zhu L, Yin J, Xu Y, et al. Helicobacter hepaticus infection induces chronic hepatitis and fibrosis in male BALB/c mice via the activation of NF-κB, Stat3, and MAPK signaling pathways. Helicobacter. 2020;25(2):e12677.

994. Cao SY, Zhu C, Feng J, Zhu LQ, Yin J, Xu YL, et al. <i>Helicobacter hepaticus</i> infection induces chronic hepatitis and fibrosis in male BALB/c mice via the activation of NF-κB, Stat3, and MAPK signaling pathways. Helicobacter. 2020;25(2):10.

995. Chen LM, Tseng HY, Chen YA, Al Haq AT, Hwang PA, Hsu HL. Oligo-Fucoidan Prevents M2 Macrophage Differentiation and HCT116 Tumor Progression. Cancers. 2020;12(2):22.

996. Chen LM, Tseng HY, Chen YA, Haq ATA, Hwang PA, Hsu HL. Oligo-fucoidan prevents m2 macrophage differentiation and HCT116 tumor progression. Cancers. 2020;12(2).

997. Chen YM, Helm ET, Gabler N, Hostetter JM, Burrough ER. Alterations in Intestinal Innate Mucosal Immunity of Weaned Pigs During Porcine Epidemic Diarrhea Virus Infection. Vet Pathol. 2020;57(5):642-52.

998. Chen YM, Helm ET, Gabler N, Hostetter JM, Burrough ER. Alterations in Intestinal Innate Mucosal Immunity of Weaned Pigs During Porcine Epidemic Diarrhea Virus Infection. Vet Pathol. 2020;57(5):642-52.

999. Dai M, Li S, Shi K, Liao J, Sun H, Liao M. Systematic Identification of Host Immune Key Factors Influencing Viral Infection in PBL of ALV-J Infected SPF Chicken. Viruses. 2020;12(1).

1000. Di Franco G, Usai A, Funel N, Palmeri M, Montesanti IER, Bianchini M, et al. Use of zebrafish embryos as avatar of patients with pancreatic cancer: A new xenotransplantation model towards personalized medicine. World J Gastroenterol. 2020;26(21):2792-809.

1001. Di Franco G, Usai A, Funel N, Palmeri M, Rosamaria Montesanti IE, Bianchini M, et al. Use of zebrafish embryos as avatar of patients with pancreatic cancer: A new xenotransplantation model towards personalized medicine. World J Gastroenterol. 2020;26(21):2792-809.

1002. Ersoz M, Erdemir A, Derman S, Arasoglu T, Mansuroglu B. Quercetin-loaded nanoparticles enhance cytotoxicity and antioxidant activity on C6 glioma cells. Pharm Dev Technol. 2020;25(6):757-66.

1003. Ersoz M, Erdemir A, Derman S, Arasoglu T, Mansuroglu B. Quercetin-loaded nanoparticles enhance cytotoxicity and antioxidant activity on C6 glioma cells. Pharm Dev Technol. 2020;25(6):757-66.

1004. Greco S, Islam MS, Zannotti A, Delli Carpini G, Giannubilo SR, Ciavattini A, et al. Quercetin and indole-3-carbinol inhibit extracellular matrix expression in human primary uterine leiomyoma cells. Reprod Biomed Online. 2020;40(4):593-602.

1005. Griess B, Mir S, Datta K, Teoh-Fitzgerald M. Scavenging reactive oxygen species selectively inhibits M2 macrophage polarization and their pro-tumorigenic function in part, via Stat3 suppression. Free Radic Biol Med. 2020;147:48-60.

1006. Griess B, Mir S, Datta K, Teoh-Fitzgerald M. Scavenging reactive oxygen species selectively inhibits M2 macrophage polarization and their pro-tumorigenic function in part, via Stat3 suppression. Free Radic Biol Med. 2020;147:48-60.

1007. Huang WY, Liu KH, Lin S, Chen TY, Tseng CY, Chen HY, et al. NADPH oxidase 2 as a potential therapeutic target for protection against cognitive deficits following systemic inflammation in mice. Brain Behav Immun. 2020;84:242-52.

1008. Huang WY, Liu KH, Lin S, Chen TY, Tseng CY, Chen HY, et al. NADPH oxidase 2 as a potential therapeutic target for protection against cognitive deficits following systemic inflammation in mice. Brain Behav Immun. 2020;84:242-52.

1009. Hussain Y, Luqman S, Meena A. Research progress in flavonoids as potential anticancer drug including synergy with other approaches. Curr Top Med Chem. 2020;20(20):1791-809.

1010. Islam SU, Ahmed MB, Ahsan H, Islam M, Shehzad A, Sonn JK, et al. An update on the role of dietary phytochemicals in human skin cancer: New insights into molecular mechanisms. Antioxidants. 2020;9(10):1-30.

1011. James RE, Schalks R, Browne E, Eleftheriadou I, Munoz CP, Mazarakis ND, et al. Persistent elevation of intrathecal pro-inflammatory cytokines leads to multiple sclerosis-like cortical demyelination and neurodegeneration. Acta Neuropathol Commun. 2020;8(1).

1012. Janjua KA, Shehzad A, Shahzad R, Islam SU, Islam MU. Nanocurcumin: A Double-Edged Sword for Microcancers. Curr Pharm Des. 2020;26(45):5783-92.

1013. Kim TW, Hong DW, Kang CM, Hong SH. A novel PPARɣ ligand, PPZ023, overcomes radioresistance via ER stress and cell death in human non-small-cell lung cancer cells. Exp Mol Med. 2020;52(10):1730-43.

1014. Kim TW, Hong DW, Kang CM, Hong SH. A novel PPARɣ ligand, PPZ023, overcomes radioresistance via ER stress and cell death in human non-small-cell lung cancer cells. Exp Mol Med. 2020;52(10):1730-43.

1015. Kim TW, Hong DW, Park JW, Hong SH. CB11, a novel purine-based PPARɣ ligand, overcomes radio-resistance by regulating ATM signalling and EMT in human non-small-cell lung cancer cells. Br J Cancer. 2020;123(12):1737-48.

1016. Kim TW, Hong DW, Park JW, Hong SH. CB11, a novel purine-based PPARɣ ligand, overcomes radio-resistance by regulating ATM signalling and EMT in human non-small-cell lung cancer cells. Br J Cancer. 2020;123(12):1737-48.

1017. Konaté MM, Antony S, Doroshow JH. Inhibiting the Activity of NADPH Oxidase in Cancer. Antioxid Redox Signal. 2020;33(6):435-54.

1018. Konaté MM, Antony S, Doroshow JH. Inhibiting the Activity of NADPH Oxidase in Cancer. Antioxid Redox Signal. 2020;33(6):435-54.

1019. Kuai Y, Liu H, Liu D, Liu Y, Sun Y, Xie J, et al. An ultralow dose of the NADPH oxidase inhibitor diphenyleneiodonium (DPI) is an economical and effective therapeutic agent for the treatment of colitis-associated colorectal cancer. Theranostics. 2020;10(15):6743-57.

1020. Kuai Y, Liu H, Liu DY, Liu YL, Sun Y, Xie JS, et al. An ultralow dose of the NADPH oxidase inhibitor diphenyleneiodonium (DPI) is an economical and effective therapeutic agent for the treatment of colitis-associated colorectal cancer. Theranostics. 2020;10(15):6743-57.

1021. Lee WH, Loo CY, Traini D, Young PM. Development and Evaluation of Paclitaxel and Curcumin Dry Powder for Inhalation Lung Cancer Treatment. Pharmaceutics. 2020;13(1).

1022. Li HY, Zhang HL, zhao FJ, Wang SQ, Wang ZX, Wei ZY. Modulation of Gut Microbiota, Short-Chain Fatty Acid Production, and Inflammatory Cytokine Expression in the Cecum of Porcine Deltacoronavirus-Infected Chicks. Front Microbiol. 2020;11.

1023. Li HY, Zhang HL, Zhao FJ, Wang SQ, Wang ZX, Wei ZY. Modulation of Gut Microbiota, Short-Chain Fatty Acid Production, and Inflammatory Cytokine Expression in the Cecum of Porcine Deltacoronavirus-Infected Chicks. Front Microbiol. 2020;11:897.

1024. Li S, Ung TT, Nguyen TT, Sah DK, Park SY, Jung YD. Cholic acid stimulates mmp-9 in human colon cancer cells via activation of mapk, ap-1, and nf-κb activity. Int J Mol Sci. 2020;21(10).

1025. Lin CC, Lin WN, Cho RL, Yang CC, Yeh YC, Hsiao LD, et al. Induction of HO-1 by mevastatin mediated via a Nox/ROS-dependent c-Src/PDGFRΑ/PI3K/AKT/NRF2/ARE cascade suppresses TNF-α-induced lung inflammation. Journal of Clinical Medicine. 2020;9(1).

1026. Liskova A, Stefanicka P, Samec M, Smejkal K, Zubor P, Bielik T, et al. Dietary phytochemicals as the potential protectors against carcinogenesis and their role in cancer chemoprevention. Clinical and Experimental Medicine. 2020;20(2):173-90.

1027. Malla RR, Deepak K, Merchant N, Dasari VR. Breast Tumor Microenvironment: Emerging target of therapeutic phytochemicals. Phytomedicine. 2020;70:153227.

1028. Mandal M, Sahoo SK, Patra P, Mallik S, Zhao Z. In silico ranking of phenolics for therapeutic effectiveness on cancer stem cells. BMC Bioinformatics. 2020;21.

1029. Mandal M, Sahoo SK, Patra P, Mallik S, Zhao Z. In silico ranking of phenolics for therapeutic effectiveness on cancer stem cells. BMC Bioinformatics. 2020;21(Suppl 21):499.

1030. Muthuramalingam K, Cho M, Kim Y. Role of NAPDH oxidase and its therapeutic intervention in TGF-β-mediated EMT progression: an in vitro analysis on HeLa cervical cancer cells. Appl Biol Chem. 2020;63(1).

1031. Peregrine J, Gurung S, Lindgren MC, Husain S, Zavy MT, Myers DA, et al. Zika virus infection, reproductive organ targeting, and semen transmission in the male olive baboon. J Virol. 2020;94(1).

1032. Peregrine J, Gurung S, Lindgren MC, Husain S, Zavy MT, Myers DA, et al. Zika Virus Infection, Reproductive Organ Targeting, and Semen Transmission in the Male Olive Baboon. J Virol. 2020;94(1):16.

1033. Piszczatowska K, Przybylska D, Sikora E, Mosieniak G. Inhibition of NADPH Oxidases Activity by Diphenyleneiodonium Chloride as a Mechanism of Senescence Induction in Human Cancer Cells. Antioxidants (Basel). 2020;9(12).

1034. Piszczatowska K, Przybylska D, Sikora E, Mosieniak G. Inhibition of NADPH Oxidases Activity by Diphenyleneiodonium Chloride as a Mechanism of Senescence Induction in Human Cancer Cells. Antioxidants. 2020;9(12):15.

1035. Singer D, Bengtson LGS, Elliott C, Buikema AR, Franchino-Elder J. Healthcare Resource Utilization, Exacerbations, and Readmissions Among Medicare Patients with Chronic Obstructive Pulmonary Disease After Long-Acting Muscarinic Antagonist Therapy Initiation with Soft Mist versus Dry Powder Inhalers. Int J Chron Obstruct Pulmon Dis. 2020;15:3239-50.

1036. Singer D, Bengtson LGS, Elliott C, Buikema AR, Franchino-Elder J. Healthcare Resource Utilization, Exacerbations, and Readmissions Among Medicare Patients with Chronic Obstructive Pulmonary Disease After Long-Acting Muscarinic Antagonist Therapy Initiation with Soft Mist versus Dry Powder Inhalers. Int J Chronic Obstr Pulm Dis. 2020;15:3239-50.

1037. Song J, Li Q, Everaert N, Liu R, Zheng M, Zhao G, et al. Effects of inulin supplementation on intestinal barrier function and immunity in specific pathogen-free chickens with Salmonella infection. J Anim Sci. 2020;98(1).

1038. Song J, Li Q, Everaert N, Liu R, Zheng M, Zhao G, et al. Effects of inulin supplementation on intestinal barrier function and immunity in specific pathogen-free chickens with Salmonella infection. J Anim Sci. 2020;98(1).

1039. Sp N, Kang DY, Kim DH, Yoo JS, Jo ES, Rugamba A, et al. Tannic acid inhibits Non-small Cell Lung Cancer (NSCLC) stemness by inducing G0/G1 Cell cycle arrest and intrinsic apoptosis. Anticancer Res. 2020;40(6):3209-20.

1040. Thyagarajan A, Forino AS, Konger RL, Sahu RP. Dietary polyphenols in cancer chemoprevention: Implications in pancreatic cancer. Antioxidants. 2020;9(8):1-20.

1041. Tse C, Warner A, Farook R, Cronin JG. Phytochemical targeting of STAT3 orchestrated lipid metabolism in therapy-resistant cancers. Biomolecules. 2020;10(8):1-22.

1042. Uddin MS, Al Mamun A, Jakaria M, Thangapandiyan S, Ahmad J, Rahman MA, et al. Emerging promise of sulforaphane-mediated Nrf2 signaling cascade against neurological disorders. Sci Total Environ. 2020;707:12.

1043. Uddin MS, Mamun AA, Jakaria M, Thangapandiyan S, Ahmad J, Rahman MA, et al. Emerging promise of sulforaphane-mediated Nrf2 signaling cascade against neurological disorders. Sci Total Environ. 2020;707.

1044. Wang S, Zhang J, Zhang Y, Yang J, Wang L, Qi Y, et al. Cytokine Storm in Domestic Pigs Induced by Infection of Virulent African Swine Fever Virus. Front Vet Sci. 2020;7:601641.

1045. Yousuf M, Shamsi A, Khan P, Shahbaaz M, Alajmi MF, Hussain A, et al. Ellagic acid controls cell proliferation and induces apoptosis in breast cancer cells via inhibition of cyclin-dependent kinase 6. Int J Mol Sci. 2020;21(10).

1046. Zare-Zardini H, Alemi A, Taheri-Kafrani A, Hosseini SA, Soltaninejad H, Hamidieh AA, et al. Assessment of a New Ginsenoside Rh2 Nanoniosomal Formulation for Enhanced Antitumor Efficacy on Prostate Cancer: An in vitro Study. Drug Des Devel Ther. 2020;14:3315-24.

1047. Zare-Zardini H, Alemi A, Taheri-Kafrani A, Hosseini SA, Soltaninejad H, Hamidieh AA, et al. Assessment of a new ginsenoside RH2 nanoniosomal formulation for enhanced antitumor efficacy on prostate cancer: An in vitro study. Drug Design, Development and Therapy. 2020;14:3315-24.

1048. Zeng J, Xu H, Fan PZ, Xie J, He J, Yu J, et al. Kaempferol blocks neutrophil extracellular traps formation and reduces tumour metastasis by inhibiting ROS-PAD4 pathway. J Cell Mol Med. 2020;24(13):7590-9.

1049. Zeng J, Xu H, Fan PZ, Xie J, He J, Yu J, et al. Kaempferol blocks neutrophil extracellular traps formation and reduces tumour metastasis by inhibiting ROS-PAD4 pathway. J Cell Mol Med. 2020;24(13):7590-9.

1050. Zhang L, Chinnathambi A, Alharbi SA, Veeraraghavan VP, Mohan SK, Zhang G. Punicalagin promotes the apoptosis in human cervical cancer (ME-180) cells through mitochondrial pathway and by inhibiting the NF-kB signaling pathway. Saudi J Biol Sci. 2020;27(4):1100-6.

1051. Zhang L, Chinnathambi A, Alharbi SA, Veeraraghavan VP, Mohan SK, Zhang G. Punicalagin promotes the apoptosis in human cervical cancer (ME-180) cells through mitochondrial pathway and by inhibiting the NF-kB signaling pathway. Saudi J Biol Sci. 2020;27(4):1100-6.

1052. Bakshi M, Hebert D, Gulbronson C, Bauchan G, Tuo W, Zarlenga D. Ostertagia ostertagi Mediates Early Host Immune Responses via Macrophage and Toll-Like Receptor Pathways. Infect Immun. 2021;89(6).

1053. Barrios LC, Pinheiro APD, Gibaldi D, Silva AA, Silva P, Roffê E, et al. Behavioral alterations in long-term <i>Toxoplasma gondii</i> infection of C57BL/6 mice are associated with neuroinflammation and disruption of the blood brain barrier. PLoS One. 2021;16(10):30.

1054. Chen H, Yang H, Deng J, Fan D. Ginsenoside Rk3 Ameliorates Obesity-Induced Colitis by Regulating of Intestinal Flora and the TLR4/NF-κB Signaling Pathway in C57BL/6 Mice. J Agric Food Chem. 2021;69(10):3082-93.

1055. Cheng D, Tu W, Chen L, Wang H, Wang Q, Liu H, et al. MSCs enhances the protective effects of valsartan on attenuating the doxorubicin-induced myocardial injury via AngII/NOX/ROS/MAPK signaling pathway. Aging (Albany NY). 2021;13(18):22556-70.

1056. Cheng D, Tu WC, Chen LB, Wang HR, Wang QF, Liu HN, et al. MSCs enhances the protective effects of valsartan on attenuating the doxorubicin-induced myocardial injury via AngII/NOX/ROS/MAPK signaling pathway. Aging-US. 2021;13(18):22556-70.

1057. Choi HD, Kim KY, Park KI, Kim SH, Park SG, Yu SN, et al. Dual role of reactive oxygen species in autophagy and apoptosis induced by compound PN in prostate cancer cells. Molecular and Cellular Toxicology. 2021;17(1):41-50.

1058. Chraibi S, Rosière R, De Prez E, Antoine MH, Remmelink M, Langer I, et al. Pulmonary and renal tolerance of cisplatin-based regimens combining intravenous and endotracheal routes for lung cancer treatment in mice. Int J Pharm. 2021;599:120425.

1059. Chraibi S, Rosière R, De Prez E, Antoine MH, Remmelink M, Langer I, et al. Pulmonary and renal tolerance of cisplatin-based regimens combining intravenous and endotracheal routes for lung cancer treatment in mice. Int J Pharm. 2021;599:15.

1060. Chraibi S, Rosière R, De Prez E, Gérard P, Antoine MH, Langer I, et al. Preclinical tolerance evaluation of the addition of a cisplatin-based dry powder for inhalation to the conventional carboplatin-paclitaxel doublet for treatment of non-small cell lung cancer. Biomedicine and Pharmacotherapy. 2021;139.

1061. Chraibi S, Rosière R, De Prez E, Gérard P, Antoine MH, Langer I, et al. Preclinical tolerance evaluation of the addition of a cisplatin-based dry powder for inhalation to the conventional carboplatin-paclitaxel doublet for treatment of non-small cell lung cancer. Biomed Pharmacother. 2021;139:111716.

1062. Chraibi S, Rosière R, Larbanoix L, Gérard P, Hennia I, Laurent S, et al. The combination of an innovative dry powder for inhalation and a standard cisplatin-based chemotherapy in view of therapeutic intensification against lung tumours. Eur J Pharm Biopharm. 2021;164:93-104.

1063. Chraibi S, Rosière R, Larbanoix L, Gérard P, Hennia I, Laurent S, et al. The combination of an innovative dry powder for inhalation and a standard cisplatin-based chemotherapy in view of therapeutic intensification against lung tumours. Eur J Pharm Biopharm. 2021;164:93-104.

1064. Dede MIC, Kiper G, Ayav T, Ozdemirel B, Tatlıcıoglu E, Hanalioglu S, et al. Human–robot interfaces of the neuroboscope: A minimally invasive endoscopic pituitary tumor surgery robotic assistance system. Journal of Medical Devices, Transactions of the ASME. 2021;15(1).

1065. Ghoreishy SM, Aminianfar A, Benisi-Kohansal S, Azadbakht L, Esmaillzadeh A. Association between dietary phytochemical index and breast cancer: a case-control study. Breast Cancer. 2021;28(6):1283-91.

1066. Glass MC, Smith JM, Cheng HH, Delany ME. Marek's Disease Virus Telomeric Integration Profiles of Neoplastic Host Tissues Reveal Unbiased Chromosomal Selection and Loss of Cellular Diversity during Tumorigenesis. Genes (Basel). 2021;12(10).

1067. Glass MC, Smith JM, Cheng HH, Delany ME. Marek's Disease Virus Telomeric Integration Profiles of Neoplastic Host Tissues Reveal Unbiased Chromosomal Selection and Loss of Cellular Diversity during Tumorigenesis. Genes. 2021;12(10):20.

1068. Guan R, Van Le Q, Yang H, Zhang D, Gu H, Yang Y, et al. A review of dietary phytochemicals and their relation to oxidative stress and human diseases. Chemosphere. 2021;271:129499.

1069. He M, Yan G, Wang Y, Gong R, Lei H, Yu S, et al. Blue LED causes autophagic cell death in human osteosarcoma by increasing ROS generation and dephosphorylating EGFR. J Cell Mol Med. 2021;25(11):4962-73.

1070. Hossain MM, Pérez-López E, Todd CD, Wei Y, Bonham-Smith PC. Endomembrane-Targeting Plasmodiophora brassicae Effectors Modulate PAMP Triggered Immune Responses in Plants. Front Microbiol. 2021;12.

1071. Hossain MM, Pérez-López E, Todd CD, Wei Y, Bonham-Smith PC. Endomembrane-Targeting Plasmodiophora brassicae Effectors Modulate PAMP Triggered Immune Responses in Plants. Front Microbiol. 2021;12:651279.

1072. Jiang H, Gao S, Mao M, Diao Y, Tang Y, Hu J. The expression profile of miR-222b-5p/MAPK10 in spleens of SPF chickens infected with REV-SNV at 28-42 dpi. Pol J Vet Sci. 2021;24(3):439-43.

1073. Jiang H, Gao S, Mao M, Điao Y, Tang Y, Hu J. The expression profile of miR-222b-5p/MAPK10 in spleens of SPF chickens infected with REV-SNV at 28-42 dpi. Pol J Vet Sci. 2021;24(3):439-43.

1074. Kim K, Kang YJ, Park SR, Kim DS, Lee SW, Ko K, et al. Effect of leaf position and days post-infiltration on transient expression of colorectal cancer vaccine candidate proteins GA733-Fc and GA733-FcK in Nicotiana benthamiana plant. PeerJ. 2021;9.

1075. Kim K, Kang YJ, Park SR, Kim DS, Lee SW, Ko K, et al. Effect of leaf position and days post-infiltration on transient expression of colorectal cancer vaccine candidate proteins GA733-Fc and GA733-FcK in Nicotiana benthamiana plant. PeerJ. 2021;9:e10851.

1076. Kumar S, Gupta E, Gupta N, Kaushik S, Srivastava VK, Mehta S, et al. Functional role of iNOS-Rac2 interaction in neutrophil extracellular traps (NETs) induced cytotoxicity in sepsis. Clin Chim Acta. 2021;513:43-9.

1077. Latha K, Jamison KF, Watford WT. Tpl2 Ablation Leads to Hypercytokinemia and Excessive Cellular Infiltration to the Lungs During Late Stages of Influenza Infection. Front Immunol. 2021;12.

1078. Lee WH, Loo CY, Traini D, Young PM. Development and evaluation of paclitaxel and curcumin dry powder for inhalation lung cancer treatment. Pharmaceutics. 2021;13(1):1-19.

1079. Li K, Feng X, Hikosaka K, Norose K. Murine model of primary acquired ocular toxoplasmosis: Fluorescein angiography and multiplex immune mediator profiles in the aqueous humor. Investigative Ophthalmology and Visual Science. 2021;62(3).

1080. Li ZY, Chen SY, Weng MH, Yen GC. Ursolic acid restores sensitivity to gemcitabine through the RAGE/NF-kB/MDR1 axis in pancreatic cancer cells and in a mouse xenograft model. J Food Drug Anal. 2021;29(2):262-74.

1081. Liu J, Wu X, Wang H, Wei J, Wu Q, Wang X, et al. HFE inhibits type I IFNs signaling by targeting the SQSTM1-mediated MAVS autophagic degradation. Autophagy. 2021;17(8):1962-77.

1082. Liu M, Wang D, Luo Y, Hu L, Bi Y, Ji J, et al. Selective killing of cancer cells harboring mutant RAS by concomitant inhibition of NADPH oxidase and glutathione biosynthesis. Cell Death and Disease. 2021;12(2).

1083. Liu M, Wang D, Luo Y, Hu L, Bi Y, Ji J, et al. Selective killing of cancer cells harboring mutant RAS by concomitant inhibition of NADPH oxidase and glutathione biosynthesis. Cell Death Dis. 2021;12(2):189.

1084. Ma M, Shi F, Zhai R, Wang H, Li K, Xu C, et al. TGF-β promote epithelial-mesenchymal transition via NF-κB/NOX4/ROS signal pathway in lung cancer cells. Mol Biol Rep. 2021;48(3):2365-75.

1085. Ma MZ, Shi FX, Zhai RN, Wang H, Li K, Xu CY, et al. TGF-β promote epithelial-mesenchymal transition via NF-κB/NOX4/ROS signal pathway in lung cancer cells. Mol Biol Rep. 2021;48(3):2365-75.

1086. Ma XX, Qiu YY, Chang ZG, Gao JF, Jiang RR, Li CL, et al. Identification of Myoferlin, a Potential Serodiagnostic Antigen of Clonorchiasis, via Immunoproteomic Analysis of Sera From Different Infection Periods and Excretory-Secretory Products of Clonorchis sinensis. Front Cell Infect Microbiol. 2021;11.

1087. Ma XX, Qiu YY, Chang ZG, Gao JF, Jiang RR, Li CL, et al. Identification of Myoferlin, a Potential Serodiagnostic Antigen of Clonorchiasis, via Immunoproteomic Analysis of Sera From Different Infection Periods and Excretory-Secretory Products of Clonorchis sinensis. Front Cell Infect Microbiol. 2021;11:779259.

1088. Manogaran P, Umapathy D, Karthikeyan M, Venkatachalam K, Singaravelu A. Dietary Phytochemicals as a Potential Source for Targeting Cancer Stem Cells. Cancer Investigation. 2021;39(4):349-68.

1089. Martínez MA, Úbeda A, Trillo M. Role of NADPH oxidase in MAPK signaling activation by a 50 Hz magnetic field in human neuroblastoma cells. Electromagn Biol Med. 2021;40(1):103-16.

1090. Medina-Buelvas DM, Rodríguez-Sosa M, Vega L. Characterisation of macrophage polarisation in mice infected with ninoa strain of trypanosoma cruzi. Pathogens. 2021;10(11).

1091. Mo G, Hu B, Wang G, Xie T, Fu H, Zhang Q, et al. Prolactin affects the disappearance of ALV-J viremia in vivo and inhibits viral infection. Vet Microbiol. 2021;261.

1092. Pang S, Jia M, Gao J, Liu X, Guo W, Zhang H. Effects of dietary patterns combined with dietary phytochemicals on breast cancer metastasis. Life Sci. 2021;264.

1093. Patra S, Nayak R, Patro S, Pradhan B, Sahu B, Behera C, et al. Chemical diversity of dietary phytochemicals and their mode of chemoprevention. Biotechnology Reports. 2021;30.

1094. Patra S, Pradhan B, Nayak R, Behera C, Das S, Patra SK, et al. Dietary polyphenols in chemoprevention and synergistic effect in cancer: Clinical evidences and molecular mechanisms of action. Phytomedicine. 2021;90:153554.

1095. Patra S, Pradhan B, Nayak R, Behera C, Panda KC, Das S, et al. Apoptosis and autophagy modulating dietary phytochemicals in cancer therapeutics: Current evidences and future perspectives. Phytotherapy Research. 2021;35(8):4194-214.

1096. Petrasheuskaya TV, Wernitznig D, Kiss MA, May NV, Wenisch D, Keppler BK, et al. Estrone-salicylaldehyde <i>N</i>-methylated thiosemicarbazone hybrids and their copper complexes: solution structure, stability and anticancer activity in tumour spheroids. J Biol Inorg Chem. 2021;26(7):775-91.

1097. Petrasheuskaya TV, Wernitznig D, Kiss MA, May NV, Wenisch D, Keppler BK, et al. Estrone-salicylaldehyde N-methylated thiosemicarbazone hybrids and their copper complexes: solution structure, stability and anticancer activity in tumour spheroids. J Biol Inorg Chem. 2021;26(7):775-91.

1098. Qian S, Fang H, Zheng L, Liu M. Zingerone suppresses cell proliferation via inducing cellular apoptosis and inhibition of the PI3K/AKT/mTOR signaling pathway in human prostate cancer PC-3 cells. Journal of Biochemical and Molecular Toxicology. 2021;35(1).

1099. Quan JH, Gao FF, Chu JQ, Cha GH, Yuk JM, Wu W, et al. Silver nanoparticles induce apoptosis via NOX4-derived mitochondrial reactive oxygen species and endoplasmic reticulum stress in colorectal cancer cells. Nanomedicine. 2021;16(16):1357-75.

1100. Quan JH, Gao FF, Chu JQ, Cha GH, Yuk JM, Wu W, et al. Silver nanoparticles induce apoptosis via NOX4-derived mitochondrial reactive oxygen species and endoplasmic reticulum stress in colorectal cancer cells. Nanomedicine (Lond). 2021;16(16):1357-75.

1101. Ren CZ, Hu WY, Zhang JW, Wei YY, Yu ML, Hu TJ. Establishment of inflammatory model induced by Pseudorabies virus infection in mice. J Vet Sci. 2021;22(2):e20.

1102. Ren CZ, Hu WY, Zhang JW, Wei YY, Yu ML, Hu TJ. Establishment of inflammatory model induced by <i>Pseudorabies</i> virus infection in mice. J Vet Sci. 2021;22(2):13.

1103. Rigi S, Shayanfar M, Mousavi SM, Mohammad-Shirazi M, Sharifi G, Esmaillzadeh A. Dietary phytochemical index in relation to risk of glioma: a case-control study in Iranian adults. Nutr J. 2021;20(1):31.

1104. Rigi S, Shayanfar M, Mousavi SM, Mohammad-Shirazi M, Sharifi G, Esmaillzadeh A. Dietary phytochemical index in relation to risk of glioma: a case-control study in Iranian adults. Nutr J. 2021;20(1):9.

1105. Roy KK, Lu J, Doroshow JH. Effects of Iodonium Analogs on Nadph Oxidase 1 in Human Colon Cancer Cells. Antioxidants (Basel). 2021;10(11).

1106. Roy KK, Lu JM, Doroshow JH. Effects of Iodonium Analogs on Nadph Oxidase 1 in Human Colon Cancer Cells. Antioxidants. 2021;10(11):11.

1107. Salehi B, Quispe C, Imran M, Ul-Haq I, Živković J, Abu-Reidah IM, et al. Nigella Plants – Traditional Uses, Bioactive Phytoconstituents, Preclinical and Clinical Studies. Front Pharmacol. 2021;12.

1108. Saraf A, Dubey N, Dubey N, Sharma M. Curcumin Loaded Eudragit S100/PLGA Nanoparticles in Treatment of Colon Cancer: Formulation, Optimization, and <i>in-vitro</i> Cytotoxicity Study. Indian J Pharm Educ Res. 2021;55(2):S428-S40.

1109. Schulz C, Jung F, Küpper JH, editors. Inhibition of phase-1 biotransformation and cytostatic effects of diphenyleneiodonium on hepatoblastoma cell line HepG2 and a CYP3A4-overexpressing HepG2 cell clone. Clin Hemorheol Microcirc; 2021.

1110. Shoaib A, Tabish M, Ali S, Arafah A, Wahab S, Almarshad FM, et al. Dietary phytochemicals in cancer signalling pathways: Role of mir-na targeting. Current Medicinal Chemistry. 2021;28(39):8036-67.

1111. Singh CK, Chhabra G, Patel A, Chang H, Ahmad N. Dietary Phytochemicals in Zinc Homeostasis: A Strategy for Prostate Cancer Management. Nutrients. 2021;13(6).

1112. Singh SP, Hussain I, Konwar BK, Deka RC, Singh CB. Design of potential ikk-β inhibitors using molecular docking and molecular dynamics techniques for their anti-cancer potential. Current Computer-Aided Drug Design. 2021;17(1):83-94.

1113. Song B, Lee SJ, Kim CH. Roles of cytokines in the temporal changes of microglial membrane currents and neuronal excitability and synaptic efficacy in ATP-induced cortical injury model. Int J Mol Sci. 2021;22(13).

1114. Song B, Lee SJ, Kim CH. Roles of Cytokines in the Temporal Changes of Microglial Membrane Currents and Neuronal Excitability and Synaptic Efficacy in ATP-Induced Cortical Injury Model. Int J Mol Sci. 2021;22(13).

1115. Tang Y, Shen Y, Hong Y, Zhang Z, Zhai Q, Fu Z, et al. miR-181a regulates the host immune response against Schistosoma japonicum infection through the TLR4 receptor pathway. Parasites and Vectors. 2021;14(1).

1116. Tang Y, Shen Y, Hong Y, Zhang Z, Zhai Q, Fu Z, et al. miR-181a regulates the host immune response against Schistosoma japonicum infection through the TLR4 receptor pathway. Parasit Vectors. 2021;14(1):548.

1117. Thiruvengadam M, Venkidasamy B, Subramanian U, Samynathan R, Ali Shariati M, Rebezov M, et al. Bioactive Compounds in Oxidative Stress-Mediated Diseases: Targeting the NRF2/ARE Signaling Pathway and Epigenetic Regulation. Antioxidants (Basel). 2021;10(12).

1118. Tingting L, Wenhui L, Nianzhang Z, Zigang Q, Ohiolei JA, Li L, et al. Primary characterization of the immune responses in Tibetan pigs infected with Chinese Tibet isolate of Trichinella spiralis. BMC Vet Res. 2021;17(1).

1119. Tuli HS, Mittal S, Aggarwal D, Parashar G, Parashar NC, Upadhyay SK, et al. Path of Silibinin from diet to medicine: A dietary polyphenolic flavonoid having potential anti-cancer therapeutic significance. Seminars in Cancer Biology. 2021;73:196-218.

1120. Vaghasiya K, Ray E, Sharma A, Singh R, Jadhav K, Khan R, et al. Systematic development and optimization of spray-dried Quercetin-HP-β-cyclodextrin microparticles for DPI-based therapy of lung cancer. J Mater Sci. 2021;56(26):14700-16.

1121. Vukmirovic D, Vo NTK, Seymour C, Rollo D, Mothersill C. Influence of common dietary supplements (curcumin, andrographolide, and d-limonene) on the radiobiological responses of p53-competent colonic cancer epithelial cells. Int J Radiat Biol. 2021;97(3):341-7.

1122. Vukmirovic D, Vo NTK, Seymour C, Rollo D, Mothersill C. Influence of common dietary supplements (curcumin, andrographolide, and d-limonene) on the radiobiological responses of p53-competent colonic cancer epithelial cells. Int J Radiat Biol. 2021;97(3):341-7.

1123. Wang CM, Chen J, Zhao J, Hu SS, Zhang SQ, Mi XQ, et al. Xanthohumol Induces ROS through NADPH Oxidase, Causes Cell Cycle Arrest and Apoptosis. Oxidative Med Cell Longev. 2021;2021.

1124. Wang S, Zhang J, Zhang Y, Yang J, Wang L, Qi Y, et al. Cytokine Storm in Domestic Pigs Induced by Infection of Virulent African Swine Fever Virus. Front Vet Sci. 2021;7.

1125. Wu R, Chi Y, Yu J, Ni C, Yao J. Enhanced immersion vaccination through hyperosmotic treatment in the largemouth bass (Micropterus salmoides). Aquaculture. 2021;535.

1126. Yu Y, Li L, Sun R, Xu Z, Wang Q, Ou C, et al. Tissue distribution and developmental changes of PTEN in the immune organs of chicken and effect of IBDV infection on it. Poult Sci. 2021;100(9).

1127. Yu Y, Li L, Sun R, Xu Z, Wang Q, Ou C, et al. Tissue distribution and developmental changes of PTEN in the immune organs of chicken and effect of IBDV infection on it. Poult Sci. 2021;100(9):101356.

1128. Zhang M, Chen X, Radacsi N. New tricks of old drugs: Repurposing non-chemo drugs and dietary phytochemicals as adjuvants in anti-tumor therapies. J Control Release. 2021;329:96-120.

1129. Zhou Y, Zhang M, Wang C, Ren X, Guo T, Cao Z, et al. Solidification of volatile D-Limonene by cyclodextrin metal-organic framework for pulmonary delivery via dry powder inhalers: In vitro and in vivo evaluation. Int J Pharm. 2021;606.

1130. Zhou Y, Zhang M, Wang C, Ren X, Guo T, Cao Z, et al. Solidification of volatile D-Limonene by cyclodextrin metal-organic framework for pulmonary delivery via dry powder inhalers: In vitro and in vivo evaluation. Int J Pharm. 2021;606:120825.

1131. Akhmetzyanova ER, Timofeeva AV, Sabirov DK, Kostennikov AA, Rogozhin AA, James V, et al. Increasing Severity of Spinal Cord Injury Results in Microglia/Macrophages With Annular-Shaped Morphology and No Change in Expression of CD40 and Tumor Growth Factor-β During the Chronic Post-injury Stage. Front Molec Neurosci. 2022;14.

1132. Akhmetzyanova ER, Timofeeva AV, Sabirov DK, Kostennikov AA, Rogozhin AA, James V, et al. Increasing Severity of Spinal Cord Injury Results in Microglia/Macrophages With Annular-Shaped Morphology and No Change in Expression of CD40 and Tumor Growth Factor-β During the Chronic Post-injury Stage. Front Molec Neurosci. 2022;14:15.

1133. Al-Tarawneh SF, Dahmash EZ, Alyami H, Abu-Doleh SM, Al-Ali S, Iyire A, et al. Mechanistic modelling of targeted pulmonary delivery of dactinomycin iron oxide-loaded nanoparticles for lung cancer therapy. Pharm Dev Technol. 2022;27(10):1057-68.

1134. Al-Tarawneh SF, Dahmash EZ, Alyami H, Abu-Doleh SM, Al-Ali S, Iyire A, et al. Mechanistic modelling of targeted pulmonary delivery of dactinomycin iron oxide-loaded nanoparticles for lung cancer therapy. Pharm Dev Technol. 2022;27(10):1057-68.

1135. Al-Yozbaki M, Jabre I, Syed NH, Wilson CM. Targeting DNA methyltransferases in non-small-cell lung cancer. Seminars in Cancer Biology. 2022;83:77-87.

1136. Almazi JG, Silva DM, Trotta V, Fiore W, Ong HX, Traini D. ‘Protective Abilities of an Inhaled DPI Formulation Based on Sodium Hyaluronate against Environmental Hazards Targeting the Upper Respiratory Tract. Pharmaceutics. 2022;14(7).

1137. Balakrishnan V, Ganapathy S, Veerasamy V, Duraisamy R, Jawaharlal S, Lakshmanan V. Nerolidol assists Cisplatin to induce early apoptosis in human laryngeal carcinoma Hep 2 cells through ROS and mitochondrial-mediated pathway: An in vitro and in silico view. J Food Biochem. 2022;46(12):e14465.

1138. Balakrishnan V, Ganapathy S, Veerasamy V, Duraisamy R, Jawaharlal S, Lakshmanan V. Nerolidol assists Cisplatin to induce early apoptosis in human laryngeal carcinoma Hep 2 cells through ROS and mitochondrial-mediated pathway: An in vitro and in silico view. J Food Biochem. 2022;46(12):13.

1139. Brunaugh AD, Ding L, Wu T, Schneider M, Khalaf R, Smyth HDC. Identification of Stability Constraints in the Particle Engineering of an Inhaled Monoclonal Antibody Dried Powder. J Pharm Sci. 2022;111(2):403-16.

1140. Brunaugh AD, Ding L, Wu T, Schneider M, Khalaf R, Smyth HDC. Identification of Stability Constraints in the Particle Engineering of an Inhaled Monoclonal Antibody Dried Powder. J Pharm Sci. 2022;111(2):403-16.

1141. Bühler M, Runft S, Li D, Götting J, Detje CN, Nippold V, et al. IFN-β Deficiency Results in Fatal or Demyelinating Disease in C57BL/6 Mice Infected With Theiler’s Murine Encephalomyelitis Viruses. Front Immunol. 2022;13.

1142. Castañeda AM, Meléndez CM, Uribe D, Pedroza-Díaz J. Synergistic effects of natural compounds and conventional chemotherapeutic agents: recent insights for the development of cancer treatment strategies. Heliyon. 2022;8(6):e09519.

1143. Chan JFW, Poon VKM, Chan CCS, Chik KKH, Tsang JOL, Zou Z, et al. Low Environmental Temperature Exacerbates Severe Acute Respiratory Syndrome Coronavirus 2 Infection in Golden Syrian Hamsters. Clin Infect Dis. 2022;75(1):e1101-e11.

1144. Chan JFW, Poon VKM, Chan CCS, Chik KKH, Tsang JOL, Zou ZJ, et al. Low Environmental Temperature Exacerbates Severe Acute Respiratory Syndrome Coronavirus 2 Infection in Golden Syrian Hamsters. Clin Infect Dis. 2022;75(1):E1101-E11.

1145. Chen D, Li Q, Liang H, Huang L, Zhou H, Zheng X, et al. Exenatide enhanced the antitumor efficacy on PD-1 blockade by the attenuation of neutrophil extracellular traps. Biochem Biophys Res Commun. 2022;619:97-103.

1146. Chen D, Li Q, Liang H, Huang L, Zhou H, Zheng X, et al. Exenatide enhanced the antitumor efficacy on PD-1 blockade by the attenuation of neutrophil extracellular traps. Biochem Biophys Res Commun. 2022;619:97-103.

1147. Chen X, Li H, Zhang B, Deng Z. The synergistic and antagonistic antioxidant interactions of dietary phytochemical combinations. Crit Rev Food Sci Nutr. 2022;62(20):5658-77.

1148. Chen X, Li HY, Zhang B, Deng ZY. The synergistic and antagonistic antioxidant interactions of dietary phytochemical combinations. Crit Rev Food Sci Nutr. 2022;62(20):5658-77.

1149. Chong A, Nguyen DH, Kim HS, Chung JK, Min JJ. Pattern of F-18 FDG Uptake in Colon Cancer after Bacterial Cancer Therapy Using Engineered Salmonella Typhimurium: A Preliminary in Vivo Study. Mol Imaging. 2022;2022.

1150. Chong A, Nguyen DH, Kim HS, Chung JK, Min JJ. Pattern of F-18 FDG Uptake in Colon Cancer after Bacterial Cancer Therapy Using Engineered Salmonella Typhimurium: A Preliminary In Vivo Study. Mol Imaging. 2022;2022:9222331.

1151. Cieniewicz B, Kirillov V, Daher I, Li X, Oldenburg DG, Dong Q, et al. IKKα-Mediated Noncanonical NF-κB Signaling Is Required To Support Murine Gammaherpesvirus 68 Latency In Vivo. J Virol. 2022;96(10):e0002722.

1152. Cieniewicz B, Kirillov V, Daher I, Li XF, Oldenburg DG, Dong QW, et al. IKKα-Mediated Noncanonical NF-κB Signaling Is Required To Support Murine Gammaherpesvirus 68 Latency <i>In Vivo</i>. J Virol. 2022;96(10):15.

1153. Dakik H, El Dor M, Bourgeais J, Kouzi F, Herault O, Gouilleux F, et al. Diphenyleneiodonium Triggers Cell Death of Acute Myeloid Leukemia Cells by Blocking the Mitochondrial Respiratory Chain, and Synergizes with Cytarabine. Cancers (Basel). 2022;14(10).

1154. Dakik H, El Dor M, Bourgeais JM, Kouzi F, Herault O, Gouilleux F, et al. Diphenyleneiodonium Triggers Cell Death of Acute Myeloid Leukemia Cells by Blocking the Mitochondrial Respiratory Chain, and Synergizes with Cytarabine. Cancers. 2022;14(10):12.

1155. Gadi V, Shetty SR. Potential of Anti-inflammatory Molecules in the Chemoprevention of Breast Cancer. Recent Adv Inflamm Allergy Drug Discov. 2022;16(2):60-76.

1156. Gamage AM, Tan KS, Chan WOY, Lew ZZR, Liu J, Tan CW, et al. Human Nasal Epithelial Cells Sustain Persistent SARS-CoV-2 Infection In Vitro, despite Eliciting a Prolonged Antiviral Response. mBio. 2022;13(1).

1157. Gamage AM, Tan KS, Chan WOY, Lew ZZR, Liu J, Tan CW, et al. Human Nasal Epithelial Cells Sustain Persistent SARS-CoV-2 Infection <i>In Vitro</i>, despite Eliciting a Prolonged Antiviral Response. mBio. 2022;13(1):18.

1158. Gao XY, Liu GC, Zhang JX, Wang LH, Xu C, Yan ZA, et al. Pharmacological Properties of Ginsenoside Re. Front Pharmacol. 2022;13.

1159. Gao XY, Liu GC, Zhang JX, Wang LH, Xu C, Yan ZA, et al. Pharmacological Properties of Ginsenoside Re. Front Pharmacol. 2022;13:754191.

1160. Ghaffarian-Bahraman A, Arabnezhad MR, Keshavarzi M, Davani-Davari D, Jamshidzadeh A, Mohammadi-Bardbori A. Influence of cellular redox environment on aryl hydrocarbon receptor ligands induced melanogenesis. Toxicology in Vitro. 2022;79.

1161. Guo H, Punvittayagul C, Vachiraarunwong A, Phannasorn W, Wongpoomchai R. Cancer chemopreventive potential of cooked glutinous purple rice on the early stages of hepatocarcinogenesis in rats. Front Nutr. 2022;9.

1162. Hanieh H, Ibrahim HIM, Mohammed M, Alwassil O, Abukhalil MH, Farhan M. Activation of aryl hydrocarbon receptor signaling by gallic acid suppresses progression of human breast cancer<i> in</i><i> vitro</i> and<i> in</i><i> vivo</i>. Phytomedicine. 2022;96:13.

1163. Hanieh H, Ibrahim HIM, Mohammed M, Alwassil OI, Abukhalil MH, Farhan M. Activation of aryl hydrocarbon receptor signaling by gallic acid suppresses progression of human breast cancer in vitro and in vivo. Phytomedicine. 2022;96.

1164. Hou Z, Wang L, Su D, Cai W, Zhu Y, Liu D, et al. Global MicroRNAs Expression Profile Analysis Reveals Possible Regulatory Mechanisms of Brain Injury Induced by Toxoplasma gondii Infection. Front Neurosci. 2022;16.

1165. Hou Z, Wang L, Su D, Cai W, Zhu Y, Liu D, et al. Global MicroRNAs Expression Profile Analysis Reveals Possible Regulatory Mechanisms of Brain Injury Induced by Toxoplasma gondii Infection. Front Neurosci. 2022;16:827570.

1166. Inthawong M, Sunyakumthorn P, Wongwairot S, Anantatat T, Dunachie SJ, Im-Erbsin R, et al. A time-course comparative clinical and immune response evaluation study between the human pathogenic Orientia tsutsugamushi strains: Karp and Gilliam in a rhesus macaque (Macaca mulatta) model. Plos Neglect Trop Dis. 2022;16(8).

1167. Inthawong M, Sunyakumthorn P, Wongwairot S, Anantatat T, Dunachie SJ, Im-Erbsin R, et al. A time-course comparative clinical and immune response evaluation study between the human pathogenic Orientia tsutsugamushi strains: Karp and Gilliam in a rhesus macaque (Macaca mulatta) model. PLoS Negl Trop Dis. 2022;16(8):e0010611.

1168. Jiang K, Feng J, Qi X, Ran L, Xie L. Antiviral Activity of Oridonin Against Herpes Simplex Virus Type 1. Drug Des Devel Ther. 2022;16:4311-23.

1169. Jiang K, Feng J, Qi X, Ran LL, Xie LX. Antiviral Activity of Oridonin Against Herpes Simplex Virus Type 1. Drug Des Dev Ther. 2022;16:4311-23.

1170. Kulsantiwong J, Thitapakorn V, Sathavornmanee T, Yusuk S, Pitaksakulrat O, Tesana S, et al. Susceptibility patterns of Bithynia siamensis siamensis and Bithynia funiculata to Opisthorchis viverrini infection: an indication of the risk of opisthorchiasis transmission in non-endemic areas. Parasitol Res. 2022;121(12):3495-501.

1171. Latha K, Rao S, Sakamoto K, Watford WT. Tumor Progression Locus 2 Protects against Acute Respiratory Distress Syndrome in Influenza A Virus-Infected Mice. Microbiol Spectr. 2022;10(5).

1172. Latha K, Rao S, Sakamoto K, Watford WT. Tumor Progression Locus 2 Protects against Acute Respiratory Distress Syndrome in Influenza A Virus-Infected Mice. Microbiol Spectr. 2022;10(5):e0113622.

1173. Li C, Chen Y, Zhao Y, Lung DC, Ye Z, Song W, et al. Intravenous Injection of Coronavirus Disease 2019 (COVID-19) mRNA Vaccine Can Induce Acute Myopericarditis in Mouse Model. Clin Infect Dis. 2022;74(11):1933-50.

1174. Li C, Chen Y, Zhao Y, Lung DC, Ye Z, Song W, et al. Intravenous Injection of Coronavirus Disease 2019 (COVID-19) mRNA Vaccine Can Induce Acute Myopericarditis in Mouse Model. Clin Infect Dis. 2022;74(11):1933-50.

1175. Liang R, Chen S, Jin Y, Tao L, Ji W, Zhu P, et al. The CXCL10/CXCR3 Axis Promotes Disease Pathogenesis in Mice upon CVA2 Infection. Microbiol Spectr. 2022;10(3).

1176. Liang R, Chen S, Jin Y, Tao L, Ji W, Zhu P, et al. The CXCL10/CXCR3 Axis Promotes Disease Pathogenesis in Mice upon CVA2 Infection. Microbiol Spectr. 2022;10(3):e0230721.

1177. Maamri A, Zemova E, Moslemani K, Flockerzi F, Seitz B. Recurrent ligneous conjunctivitis after cataract surgery in a 67-year-old male patient: a case report. BMC Ophthalmol. 2022;22(1).

1178. Maamri A, Zemova E, Moslemani K, Flockerzi F, Seitz B. Recurrent ligneous conjunctivitis after cataract surgery in a 67-year-old male patient: a case report. BMC Ophthalmol. 2022;22(1):103.

1179. Maricic N, Schwermer M, Schramm A, Morosan-Puopolo G, Ketteler P, Brand-Saberi B. Zebrafish as an Orthotopic Tumor Model for Retinoblastoma Mimicking Routes of Human Metastasis. Cancers. 2022;14(23).

1180. Maricic N, Schwermer M, Schramm A, Morosan-Puopolo G, Ketteler P, Brand-Saberi B. Zebrafish as an Orthotopic Tumor Model for Retinoblastoma Mimicking Routes of Human Metastasis. Cancers (Basel). 2022;14(23).

1181. McClements DJ, Öztürk B. Utilization of Nanotechnology to Improve the Application and Bioavailability of Phytochemicals Derived from Waste Streams. J Agric Food Chem. 2022;70(23):6884-900.

1182. Michaely LM, Schuwerk L, Allnoch L, Schön K, Waltl I, Larsen PK, et al. Intact Type I Interferon Receptor Signaling Prevents Hepatocellular Necrosis but Not Encephalitis in a Dose-Dependent Manner in Rift Valley Fever Virus Infected Mice. Int J Mol Sci. 2022;23(20).

1183. Monzur S, Hassan G, Afify SM, Kumon K, Mansour H, Nawara HM, et al. Diphenyleneiodonium efficiently inhibits the characteristics of a cancer stem cell model derived from induced pluripotent stem cells. Cell Biochem Funct. 2022;40(3):310-20.

1184. Mukherjee S, Ghosh S, Sengupta A, Sarkar S, Keswani T, Chatterjee R, et al. IL-6 dependent expansion of inflammatory MDSCs (CD11b+ Gr-1+) promote Th-17 mediated immune response during experimental cerebral malaria. Cytokine. 2022;155.

1185. Mukherjee S, Ghosh S, Sengupta A, Sarkar S, Keswani T, Chatterjee R, et al. IL-6 dependent expansion of inflammatory MDSCs (CD11b+ Gr-1+) promote Th-17 mediated immune response during experimental cerebral malaria. Cytokine. 2022;155:155910.

1186. Padhy I, Paul P, Sharma T, Banerjee S, Mondal A. Molecular Mechanisms of Action of Eugenol in Cancer: Recent Trends and Advancement. Life. 2022;12(11).

1187. Pal P, Jana S, Biswas I, Mandal DP, Bhattacharjee S. Biphasic effect of the dietary phytochemical linalool on angiogenesis and metastasis. Mol Cell Biochem. 2022;477(4):1041-52.

1188. Pal P, Jana S, Biswas I, Mandal DP, Bhattacharjee S. Biphasic effect of the dietary phytochemical linalool on angiogenesis and metastasis. Mol Cell Biochem. 2022;477(4):1041-52.

1189. Pereira-Wilson C. Can dietary flavonoids be useful in the personalized treatment of colorectal cancer? World Journal of Gastrointestinal Oncology. 2022;14(6):1115-23.

1190. Prajapati KS, Gupta S, Kumar S. Targeting Breast Cancer-Derived Stem Cells by Dietary Phytochemicals: A Strategy for Cancer Prevention and Treatment. Cancers (Basel). 2022;14(12).

1191. Prajapati KS, Gupta S, Kumar S. Targeting Breast Cancer-Derived Stem Cells by Dietary Phytochemicals: A Strategy for Cancer Prevention and Treatment. Cancers. 2022;14(12):20.

1192. Qiu YY, Chang QC, Gao JF, Bao MJ, Luo HT, Song JH, et al. Multiple biochemical indices and metabolomics of Clonorchis sinensis provide a novel interpretation of biomarkers. Parasites and Vectors. 2022;15(1).

1193. Rao Malla R, Marni R, Kumari S, Chakraborty A, Lalitha P. Microbiome Assisted Tumor Microenvironment: Emerging Target of Breast Cancer. Clinical Breast Cancer. 2022;22(3):200-11.

1194. Reyes-Duarte I, Burgara-Estrella AJ, Bustamante-Córdova L, Moya-Camarena SY, Parra-Sánchez H, Álvarez-Bajo O, et al. Conjugated linoleic acid modifies transcriptional cytokine profile and induces early specific secretory IgA response in Giardia lamblia infected mice. Iranian Journal of Basic Medical Sciences. 2022;25(12):1468-76.

1195. Schirtzinger EE, Kim Y, Davis AS. Improving human coronavirus OC43 (HCoV-OC43) research comparability in studies using HCoV-OC43 as a surrogate for SARS-CoV-2. J Virol Methods. 2022;299.

1196. Schirtzinger EE, Kim Y, Davis AS. Improving human coronavirus OC43 (HCoV-OC43) research comparability in studies using HCoV-OC43 as a surrogate for SARS-CoV-2. J Virol Methods. 2022;299:114317.

1197. Shannar A, Sarwar MS, Kong AT. A New Frontier in Studying Dietary Phytochemicals in Cancer and in Health: Metabolic and Epigenetic Reprogramming. Prev Nutr Food Sci. 2022;27(4):335-46.

1198. Sharma M, Tollefsbol TO. Combinatorial epigenetic mechanisms of sulforaphane, genistein and sodium butyrate in breast cancer inhibition. Experimental Cell Research. 2022;416(1).

1199. Shoaib S, Islam N, Yusuf N. Phytocompounds from the Medicinal and Dietary Plants: Multi-target Agents for Cervical Cancer Prevention and Therapy. Current Medicinal Chemistry. 2022;29(26):4481-506.

1200. Sohel M, Islam MN, Hossain MA, Sultana T, Dutta A, Rahman MS, et al. Pharmacological Properties to Pharmacological Insight of Sesamin in Breast Cancer Treatment: A Literature-Based Review Study. International Journal of Breast Cancer. 2022;2022.

1201. Song D, Lee J, Kwak W, Song M, Oh H, Kim Y, et al. Stimbiotic Supplementation Alleviates Poor Performance and Gut Integrity in Weaned Piglets Induced by Challenge with E. coli. Animals (Basel). 2022;12(14).

1202. Song D, Lee J, Kwak W, Song M, Oh H, Kim Y, et al. Stimbiotic Supplementation Alleviates Poor Performance and Gut Integrity in Weaned Piglets Induced by Challenge with <i>E. coli</i>. Animals. 2022;12(14):13.

1203. Song Y, Jiang S, Li C, Loor JJ, Jiang Q, Yang Y, et al. Free fatty acids promote degranulation of azurophil granules in neutrophils by inducing production of NADPH oxidase-derived reactive oxygen species in cows with subclinical ketosis. J Dairy Sci. 2022;105(3):2473-86.

1204. Song YX, Jiang S, Li CY, Loor JJ, Jiang QM, Yang YC, et al. Free fatty acids promote degranulation of azurophil granules in neutrophils by inducing production of NADPH oxidase-derived reactive oxygen species in cows with subclinical ketosis. J Dairy Sci. 2022;105(3):2473-86.

1205. Sudheer S, Gangwar P, Usmani Z, Sharma M, Sharma VK, Sana SS, et al. Shaping the gut microbiota by bioactive phytochemicals: An emerging approach for the prevention and treatment of human diseases. Biochimie. 2022;193:38-63.

1206. Telang NT. Stem Cell Models for Breast and Colon Cancer: Experimental Approach for Drug Discovery. Int J Mol Sci. 2022;23(16).

1207. Theotokis P, Kesidou E, Mitsiadou D, Petratos S, Damianidou O, Boziki M, et al. Lumbar spine intrathecal transplantation of neural precursor cells promotes oligodendrocyte proliferation in hot spots of chronic demyelination. Brain Pathol. 2022;32(4).

1208. Wang P, Long F, Lin H, Wang S, Wang T. Dietary Phytochemicals Targeting Nrf2 to Enhance the Radiosensitivity of Cancer. Oxidative Med Cell Longev. 2022;2022.

1209. Wang P, Long F, Lin H, Wang S, Wang T. Dietary Phytochemicals Targeting Nrf2 to Enhance the Radiosensitivity of Cancer. Oxid Med Cell Longev. 2022;2022:7848811.

1210. Wang P, Long F, Lin H, Wang T. Dietary phytochemicals targeting Nrf2 for chemoprevention in breast cancer. Food and Function. 2022.

1211. Wang P, Long F, Lin H, Wang T. Dietary phytochemicals targeting Nrf2 for chemoprevention in breast cancer. Food Funct. 2022;13(8):4273-85.

1212. Warrier NM, Krishnan RK, Prabhu V, Hariharapura RC, Agarwal P, Kumar P. Survivin Inhibition by Piperine Sensitizes Glioblastoma Cancer Stem Cells and Leads to Better Drug Response. Int J Mol Sci. 2022;23(14).

1213. Warrier NM, Krishnan RK, Prabhu V, Hariharapura RC, Agarwal P, Kumar P. Survivin Inhibition by Piperine Sensitizes Glioblastoma Cancer Stem Cells and Leads to Better Drug Response. Int J Mol Sci. 2022;23(14).

1214. Wawszczyk J, Jesse K, Smolik S, Kapral M. Mechanism of Pterostilbene-Induced Cell Death in HT-29 Colon Cancer Cells. Molecules. 2022;27(2).

1215. Wolff CM, Bekeschus S. Synergistic In Vitro Anticancer Toxicity of Pulsed Electric Fields and Glutathione. Int J Mol Sci. 2022;23(23).

1216. Xia X, Lin Q, Zhao N, Zeng J, Yang J, Liu Z, et al. Anti-Colon Cancer Activity of Dietary Phytochemical Soyasaponin I and the Induction of Metabolic Shifts in HCT116. Molecules. 2022;27(14).

1217. Xu Q, Wang Q, Hu LT, Lin J, Jiang N, Peng XD, et al. NADPH oxidase 2 plays a protective role in experimental Aspergillus fumigatus keratitis in mice through killing fungi and limiting the degree of inflammation. Int J Ophthalmol. 2022;15(7):1044-52.

1218. Xu Q, Wang Q, Hu LT, Lin J, Jiang N, Peng XD, et al. NADPH oxidase 2 plays a protective role in experimental Aspergillus fumigatus keratitis in mice through killing fungi and limiting the degree of inflammation. Int J Ophthalmol. 2022;15(7):1044-52.

1219. Xu Y, Harinck L, Lokras AG, Gerde P, Selg E, Sjöberg CO, et al. Leucine improves the aerosol performance of dry powder inhaler formulations of siRNA-loaded nanoparticles. Int J Pharm. 2022;621.

1220. Xu Y, Harinck L, Lokras AG, Gerde P, Selg E, Sjöberg CO, et al. Leucine improves the aerosol performance of dry powder inhaler formulations of siRNA-loaded nanoparticles. Int J Pharm. 2022;621:121758.

1221. Yin X, Zhang J, Zhao W, Liu Z, Wang J. Combined Levo-tetrahydropalmatine and diphenyleneiodonium chloride enhances antitumor activity in hepatocellular carcinoma. Pharmacol Res. 2022;179.

1222. Yoo HS, Won SB, Kwon YH. Luteolin Induces Apoptosis and Autophagy in HCT116 Colon Cancer Cells via p53-Dependent Pathway. Nutrition and Cancer. 2022;74(2):677-86.

1223. Yoo HS, Won SB, Kwon YH. Luteolin Induces Apoptosis and Autophagy in HCT116 Colon Cancer Cells via p53-Dependent Pathway. Nutr Cancer. 2022;74(2):677-86.

1224. Zhu Y, Wang H, Hua J, Ye W, Chen L, Ni Z, et al. Isolation and Pathogenicity of a Novel Goose Astrovirus from Overfed Adult Landaise Geese in China. Viruses. 2022;14(12).

1225. Acikgoz Pinar A, Yildiz E, Altundag K. Dietary Phytochemical Index and the Risk of Breast Cancer: A Case-Control Study. Nutrition and Cancer. 2023;75(2):482-7.

1226. Ahmed S, Mansour M, Ishak RAH, Mortada ND. Customizable resveratrol spray-dried micro-composites for inhalation as a promising contender for treatment of idiopathic pulmonary fibrosis. Int J Pharm. 2023;642:123117.

1227. Ahmed S, Mansour M, Ishak RAH, Mortada ND. Customizable resveratrol spray-dried micro-composites for inhalation as a promising contender for treatment of idiopathic pulmonary fibrosis. Int J Pharm. 2023;642:22.

1228. Al-Janabi AHA, Roodbari NH, Tabrizi MH. Investigating the anticancer and anti-angiogenic effects of graphene oxide nanoparticles containing 6-gingerol modified with chitosan and folate. Cancer Nanotechnol. 2023;14(1):13.

1229. Alhadidi MHA, Tabrizi MH, Ghobeh M. Evaluation of the effect of designed PLGA-arctiin nanoparticles modified with folic acid and chitosan on colon cancer cells. Biotechnol Appl Biochem. 2023.

1230. Alhadidi MHA, Tabrizi MH, Ghobeh M. Evaluation of the effect of designed PLGA-arctiin nanoparticles modified with folic acid and chitosan on colon cancer cells. Biotechnol Appl Biochem. 2023:9.

1231. Bentyaghoob S, Dehghani F, Alimohammadi A, Shateri Z, Kahrizsangi MA, Nejad ET, et al. Oxidative balance score and dietary phytochemical index can reduce the risk of colorectal cancer in Iranian population. BMC Gastroenterol. 2023;23(1):183.

1232. Bentyaghoob S, Dehghani F, Alimohammadi A, Shateri Z, Kahrizsangi MA, Nejad ET, et al. Oxidative balance score and dietary phytochemical index can reduce the risk of colorectal cancer in Iranian population. BMC Gastroenterol. 2023;23(1):10.

1233. Beretti F, Gatti M, Zavatti M, Bassoli S, Pellacani G, Maraldi T. Reactive Oxygen Species Regulation of Chemoresistance and Metastatic Capacity of Melanoma: Role of the Cancer Stem Cell Marker CD271. Biomedicines. 2023;11(4).

1234. Brane A, Arora I, Tollefsbol TO. Peripubertal Nutritional Prevention of Cancer-Associated Gene Expression and Phenotypes. Cancers (Basel). 2023;15(3).

1235. Catani MV, Tullio V, Maccarrone M, Gasperi V. DNA-Protein-Interaction (DPI)-ELISA Assay for PPAR-γ Receptor Binding. Methods Mol Biol. 2023;2576:133-43.

1236. Davenne T, Percier P, Larbanoix L, Moser M, Leo O, Meylan E, et al. Inhaled dry powder cisplatin increases antitumour response to anti-PD1 in a murine lung cancer model. J Control Release. 2023;353:317-26.

1237. Davenne T, Percier P, Larbanoix L, Moser M, Leo O, Meylan E, et al. Inhaled dry powder cisplatin increases antitumour response to anti-PD1 in a murine lung cancer model. J Control Release. 2023;353:317-26.

1238. Delafiori J, Faria AVDS, de Oliveira AN, Sales GM, Dias-Audibert FL, Catharino RR. Unraveling the Metabolic Alterations Induced by Zika Infection in Prostate Epithelial (PNT1a) and Adenocarcinoma (PC-3) Cell Lines. J Proteome Res. 2023;22(1):193-203.

1239. Delafiori J, Faria AVS, de Oliveira AN, Sales GM, Dias-Audibert FL, Catharino RR. Unraveling the Metabolic Alterations Induced by Zika Infection in Prostate Epithelial (PNT1a) and Adenocarcinoma (PC-3) Cell Lines. J Proteome Res. 2023;22(1):193-203.

1240. Ding Y, Hou R, Yu J, Xing C, Zhuang C, Qu Z. Dietary Phytochemicals as Potential Chemopreventive Agents against Tobacco-Induced Lung Carcinogenesis. Nutrients. 2023;15(3).

1241. Duran Ramirez JM, Gomez J, Hanson BM, Isa T, Myckatyn TM, Walker JN. Staphylococcus aureus Breast Implant Infection Isolates Display Recalcitrance To Antibiotic Pocket Irrigants. Microbiol Spectr. 2023;11(1).

1242. El-Ghareeb WR, Kishawy ATY, Anter RGA, Aboelabbas Gouda A, Abdelaziz WS, Alhawas B, et al. Novel Antioxidant Insights of Myricetin on the Performance of Broiler Chickens and Alleviating Experimental Infection with Eimeria spp.: Crosstalk between Oxidative Stress and Inflammation. Antioxidants (Basel). 2023;12(5).

1243. El-Ghareeb WR, Kishawy ATY, Anter RGA, Gouda AA, Abdelaziz WS, Alhawas B, et al. Novel Antioxidant Insights of Myricetin on the Performance of Broiler Chickens and Alleviating Experimental Infection with <i>Eimeria</i> spp.: Crosstalk between Oxidative Stress and Inflammation. Antioxidants. 2023;12(5):19.

1244. El-Wakil ES, Abdelmaksoud HF, Wakid MH, Alsulami MN, Hammam O, Albohiri HH, et al. Annona muricata Leaf as an Anti-Cryptosporidial Agent: An In Silico Molecular Docking Analysis and In Vivo Studies. Pharmaceuticals (Basel). 2023;16(6).

1245. El-Wakil ES, Abdelmaksoud HF, Wakid MH, Alsulami MN, Hammam O, Albohiri HH, et al. <i>Annona muricata</i> Leaf as an Anti-Cryptosporidial Agent: An In Silico Molecular Docking Analysis and In Vivo Studies. Pharmaceuticals. 2023;16(6):15.

1246. Ferreira MS, Sousa JR, Bezerra Júnior PS, Cerqueira VD, Oliveira Júnior CA, Rivero GRC, et al. Experimental Yellow Fever in Squirrel Monkey: Characterization of Liver In Situ Immune Response. Viruses. 2023;15(2).

1247. Gano CA, Fatima S, Failes TW, Arndt GM, Sajinovic M, Mahns D, et al. Anti-cancer potential of synergistic phytochemical combinations is influenced by the genetic profile of prostate cancer cell lines. Front Nutr. 2023;10.

1248. Gano CA, Fatima S, Failes TW, Arndt GM, Sajinovic M, Mahns D, et al. Anti-cancer potential of synergistic phytochemical combinations is influenced by the genetic profile of prostate cancer cell lines. Front Nutr. 2023;10:1119274.

1249. Giordano KR, Saber M, Green TRF, Rojas-Valencia LM, Ortiz JB, Murphy SM, et al. Colony-Stimulating Factor-1 Receptor Inhibition Transiently Attenuated the Peripheral Immune Response to Experimental Traumatic Brain Injury. Neurotrauma Reports. 2023;4(1):284-96.

1250. Gong H, Rahman SU, Zhou K, Lin Z, Mi R, Huang Y, et al. Temporal metabolic profiling of erythrocytes in mice infected with Babesia microti. Microb Pathog. 2023;175.

1251. Gong H, Rahman SU, Zhou K, Lin Z, Mi R, Huang Y, et al. Temporal metabolic profiling of erythrocytes in mice infected with Babesia microti. Microb Pathog. 2023;175:105954.

1252. Hwang IC, Valeriano VD, Song JH, Pereira M, Oh JK, Han K, et al. Mucosal immunization with lactiplantibacillus plantarum-displaying recombinant SARS-CoV-2 epitopes on the surface induces humoral and mucosal immune responses in mice. Microb Cell Fact. 2023;22(1).

1253. Hwang IC, Valeriano VD, Song JH, Pereira M, Oh JK, Han K, et al. Mucosal immunization with lactiplantibacillus plantarum-displaying recombinant SARS-CoV-2 epitopes on the surface induces humoral and mucosal immune responses in mice. Microb Cell Fact. 2023;22(1):96.

1254. Jahangiri A, Nokhodchi A, Asare-Addo K, Salehzadeh E, Emami S, Yaqoubi S, et al. Carrier-Free Inhalable Dry Microparticles of Celecoxib: Use of the Electrospraying Technique. Biomedicines. 2023;11(6).

1255. Jahangiri A, Nokhodchi A, Asare-Addo K, Salehzadeh E, Emami S, Yaqoubi S, et al. Carrier-Free Inhalable Dry Microparticles of Celecoxib: Use of the Electrospraying Technique. Biomedicines. 2023;11(6):13.

1256. Kaushik M, Tiku AB. Molecular pathways modulated by phytochemicals in head and neck cancer. Journal of Cell Communication and Signaling. 2023;17(3):469-83.

1257. Kesavan Y, Sai Srinivasan S, Pathak S, Ramalingam S. Role of Dietary Phytochemicals in Targeting Human miRNAs for Cancer Prevention and Treatment. Current Gene Therapy. 2023;23(5):343-55.

1258. Latha K, Patel Y, Rao S, Watford WT. The Influenza-Induced Pulmonary Inflammatory Exudate in Susceptible Tpl2-Deficient Mice Is Dictated by Type I IFN Signaling. Inflammation. 2023;46(1):322-41.

1259. Li X, Zang N, Zhang N, Pang L, Lv L, Meng X, et al. DNA damage resulting from human endocrine disrupting chemical exposure: Genotoxicity, detection and dietary phytochemical intervention. Chemosphere. 2023;338:139522.

1260. Liang D, Wang W, Chen G, Li J, Dou G, Gan H, et al. Cepharanthine Dry Powder Inhaler for the Treatment of Acute Lung Injury. Molecules. 2023;28(11).

1261. Lim D, Kim K, Duysak T, So E, Jeong JH, Choy HE. Bacterial cancer therapy using the attenuated fowl-adapted Salmonella enterica serovar Gallinarum. Molecular Therapy Oncolytics. 2023;31.

1262. Liu S, Zhang Y, Zheng X, Wang Z, Wang P, Zhang M, et al. Sulforaphane Inhibits Foam Cell Formation and Atherosclerosis via Mechanisms Involving the Modulation of Macrophage Cholesterol Transport and the Related Phenotype. Nutrients. 2023;15(9).

1263. Lothong M, Rukarcheep D, Wattanaphansak S, Thammacharoen S, Deachapunya C, Poonyachoti S. Differential innate immune response of endometrial cells to porcine reproductive and respiratory syndrome virus type 1 versus type 2. PLoS One. 2023;18(4 April).

1264. Lothong M, Rukarcheep D, Wattanaphansak S, Thammacharoen S, Deachapunya C, Poonyachoti S. Differential innate immune response of endometrial cells to porcine reproductive and respiratory syndrome virus type 1 versus type 2. PLoS One. 2023;18(4):e0284658.

1265. Manogaran P, Beeraka NM, Paulraj RS, Sathiyachan-Dran P, Thammaiappa M. Impediment of Cancer by Dietary Plant-derived Alkaloids Through Oxi-dative Stress: Implications of PI3K/AKT Pathway in Apoptosis, Autopha-gy, and Ferroptosis. Curr Top Med Chem. 2023;23(10):860-77.

1266. Marino P, Pepe G, Basilicata MG, Vestuto V, Marzocco S, Autore G, et al. Potential Role of Natural Antioxidant Products in Oncological Diseases. Antioxidants. 2023;12(3).

1267. Matsuyama-Kato A, Shojadoost B, Boodhoo N, Raj S, Alizadeh M, Fazel F, et al. Activated Chicken Gamma Delta T Cells Are Involved in Protective Immunity against Marek’s Disease. Viruses. 2023;15(2).

1268. Matsuyama-Kato A, Shojadoost B, Boodhoo N, Raj S, Alizadeh M, Fazel F, et al. Activated Chicken Gamma Delta T Cells Are Involved in Protective Immunity against Marek's Disease. Viruses. 2023;15(2).

1269. Mirmohammadali SN, Rosenkranz SK. Dietary phytochemicals, gut microbiota composition, and health outcomes in human and animal models. Bioscience of Microbiota, Food and Health. 2023;42(3):152-71.

1270. Mishra AP, Singh P, Yadav S, Nigam M, Seidel V, Rodrigues CF. Role of the Dietary Phytochemical Curcumin in Targeting Cancer Cell Signalling Pathways. Plants. 2023;12(9).

1271. Mishra AP, Swetanshu, Singh P, Yadav S, Nigam M, Seidel V, et al. Role of the Dietary Phytochemical Curcumin in Targeting Cancer Cell Signalling Pathways. Plants (Basel). 2023;12(9).

1272. Morshed A, Al Azad S, Mia MAR, Uddin MF, Ema TI, Yeasin RB, et al. Oncoinformatic screening of the gene clusters involved in the HER2-positive breast cancer formation along with the in silico pharmacodynamic profiling of selective long-chain omega-3 fatty acids as the metastatic antagonists. Mol Divers. 2023;27(6):2651-72.

1273. Morshed AKMH, Al Azad S, Mia MAR, Uddin MF, Ema TI, Yeasin RB, et al. Oncoinformatic screening of the gene clusters involved in the HER2-positive breast cancer formation along with the in silico pharmacodynamic profiling of selective long-chain omega-3 fatty acids as the metastatic antagonists. Mol Divers. 2023;27(6):2651-72.

1274. Murai T, Matsuda S. Pleiotropic Signaling by Reactive Oxygen Species Concerted with Dietary Phytochemicals and Microbial-Derived Metabolites as Potent Therapeutic Regulators of the Tumor Microenvironment. Antioxidants. 2023;12(5).

1275. Pereira RD, Rabelo RAN, Oliveira NFD, Porto SLT, Andrade A, Queiroz CM, Jr., et al. A 5-Lipoxygenase Inhibitor, Zileuton, Modulates Host Immune Responses and Improves Lung Function in a Model of Severe Acute Respiratory Syndrome (SARS) Induced by <i>Betacoronavirus</i>. Viruses-Basel. 2023;15(10):18.

1276. Pereira RDD, Rabelo RAN, Oliveira NFDM, Porto SLT, Andrade ACDSP, Queiroz-Junior CM, et al. A 5-Lipoxygenase Inhibitor, Zileuton, Modulates Host Immune Responses and Improves Lung Function in a Model of Severe Acute Respiratory Syndrome (SARS) Induced by Betacoronavirus. Viruses. 2023;15(10).

1277. Peter RM, Chou PJ, Shannar A, Patel K, Pan Y, Dave PD, et al. An Update on Potential Molecular Biomarkers of Dietary Phytochemicals Targeting Lung Cancer Interception and Prevention. Pharm Res. 2023.

1278. Rajaei N, Rahgouy G, Panahi N, Razzaghi-Asl N. Bioinformatic analysis of highly consumed phytochemicals as P-gp binders to overcome drug-resistance. Research in Pharmaceutical Sciences. 2023;18(5):505-16.

1279. Rudzińska A, Juchaniuk P, Oberda J, Wiśniewska J, Wojdan W, Szklener K, et al. Phytochemicals in Cancer Treatment and Cancer Prevention—Review on Epidemiological Data and Clinical Trials. Nutrients. 2023;15(8).

1280. Saleh MM, Darwish ZE, El Nouaem MI, Fayed NA, Mourad GM, Ramadan OR. The potential preventive effect of dietary phytochemicals In Vivo. BDJ Open. 2023;9(1).

1281. Shah D, Gandhi M, Kumar A, Cruz-Martins N, Sharma R, Nair S. Current insights into epigenetics, noncoding RNA interactome and clinical pharmacokinetics of dietary polyphenols in cancer chemoprevention. Crit Rev Food Sci Nutr. 2023;63(12):1755-91.

1282. Shoaib S, Ansari MA, Ghazwani M, Hani U, Jamous YF, Alali Z, et al. Prospective Epigenetic Actions of Organo-Sulfur Compounds against Cancer: Perspectives and Molecular Mechanisms. Cancers. 2023;15(3).

1283. Sohel M, Aktar S, Biswas P, Amin MA, Hossain MA, Ahmed N, et al. Exploring the anti-cancer potential of dietary phytochemicals for the patients with breast cancer: A comprehensive review. Cancer Medicine. 2023;12(13):14556-83.

1284. Song D, Lee J, Kwak W, Oh H, Chang S, An J, et al. Effects of stimbiotic supplementation on gut health, immune response, and intestinal microbiota in weaned piglets challenged with E. coli. Front Vet Sci. 2023;10:1187002.

1285. Song D, Lee J, Kwak W, Oh H, Chang S, An J, et al. Effects of stimbiotic supplementation on gut health, immune response, and intestinal microbiota in weaned piglets challenged with <i>E. coli</i>. Front Vet Sci. 2023;10:15.

1286. Telang NT. Natural products as drug candidates for breast cancer (Review). Oncol Lett. 2023;26(2).

1287. Tung YC, Sung PH, Chen PC, Wang HC, Lee JH, Su ZY. Chemoprevention of lotus leaf ethanolic extract through epigenetic activation of the NRF2-mediated pathway in murine skin JB6 P+ cell neoplastic transformation. J Tradit Complement Med. 2023;13(4):337-44.

1288. Walter J, Mende J, Hutagalung S, Alhalabi OT, Grutza M, Zheng G, et al. The Single-Dose Application of Interleukin-4 Ameliorates Secondary Brain Damage in the Early Phase after Moderate Experimental Traumatic Brain Injury in Mice. Int J Mol Sci. 2023;24(16).

1289. Wang M, Liu J, Zhao Y, Li H, Zhou J, Cheng Z, et al. TRIM25 participates in the fibrous tissue hyperplasia induced by ALV-J infection in chickens by targeting 14-3-3σ protein. Res Vet Sci. 2023;155:126-36.

1290. Wang M, Liu J, Zhao Y, Li H, Zhou J, Cheng Z, et al. TRIM25 participates in the fibrous tissue hyperplasia induced by ALV-J infection in chickens by targeting 14-3-3σ protein. Res Vet Sci. 2023;155:126-36.

1291. Yao CJ, Chang CL, Hu MH, Liao CH, Lai GM, Chiou TJ, et al. Drastic Synergy of Lovastatin and Antrodia camphorata Extract Combination against PC3 Androgen-Refractory Prostate Cancer Cells, Accompanied by AXL and Stemness Molecules Inhibition. Nutrients. 2023;15(21).

1292. Ye Y, Ma Y, Kong M, Wang Z, Sun K, Li F. Effects of Dietary Phytochemicals on DNA Damage in Cancer Cells. Nutrition and Cancer. 2023;75(3):761-75.

1293. Zhang L, Cao Y, Guo X, Wang X, Han X, Kanwore K, et al. Hypoxia-induced ROS aggravate tumor progression through HIF-1α-SERPINE1 signaling in glioblastoma. Journal of Zhejiang University: Science B. 2023;24(1):32-49.

1294. Zhang L, Cao Y, Guo X, Wang X, Han X, Kanwore K, et al. Hypoxia-induced ROS aggravate tumor progression through HIF-1α-SERPINE1 signaling in glioblastoma. J Zhejiang Univ Sci B. 2023;24(1):32-49.

1295. Zhang W, Wang C, Chen F, He Y, Yin S, Peng Y, et al. Phytochemicals and Glioma: Results from Dietary Mixed Exposure. Brain Sci. 2023;13(6).

1296. Zhang WCB, Wang C, Chen F, He YQ, Yin S, Peng Y, et al. Phytochemicals and Glioma: Results from Dietary Mixed Exposure. Brain Sci. 2023;13(6):16.

1297. Zhu A, Li X, Wang J. Integrating bulk-seq and single-cell-seq reveals disulfidptosis potential index associating with neuroblastoma prognosis and immune infiltration. J Cancer Res Clin Oncol. 2023;149(18):16647-58.

1298. Zhu A, Li X, Wang J. Integrating bulk-seq and single-cell-seq reveals disulfidptosis potential index associating with neuroblastoma prognosis and immune infiltration. J Cancer Res Clin Oncol. 2023;149(18):16647-58.

1299. Zhu W, Oteiza PI. NADPH oxidase 1: A target in the capacity of dimeric ECG and EGCG procyanidins to inhibit colorectal cancer cell invasion. Redox Biol. 2023;65.

1300. Zhu W, Oteiza PI. NADPH oxidase 1: A target in the capacity of dimeric ECG and EGCG procyanidins to inhibit colorectal cancer cell invasion. Redox Biol. 2023;65:102827.

1301. Zhu W, Yang S, Meng D, Wang Q, Ji J. Targeting NADPH Oxidase and Integrin α5β1 to Inhibit Neutrophil Extracellular Traps-Mediated Metastasis in Colorectal Cancer. Int J Mol Sci. 2023;24(21).

1302. Zhu W, Yang S, Meng D, Wang Q, Ji J. Targeting NADPH Oxidase and Integrin α5β1 to Inhibit Neutrophil Extracellular Traps-Mediated Metastasis in Colorectal Cancer. Int J Mol Sci. 2023;24(21).

1303. Maddaloni E, Bolli GB, Frier BM, Little RR, Leslie RD, Pozzilli P, et al. C-peptide determination in the diagnosis of type of diabetes and its management: A clinical perspective. Diabetes Obes Metab.15.

1304. Park JW, Chang SY, Lim JS, Park SJ, Park JJ, Cheon JH, et al. Impact of Visceral Fat on Survival and Metastasis of Stage III Colorectal Cancer. Gut Liver.9.

1305. Wang YY, Chen B, Zhang JW, Li HR, Zeng XF, Zhang Z, et al. Diets with higher insulinaemic potential are associated with increased risk of overall and cardiovascular disease-specific mortality. Br J Nutr.10.

1306. Corallini A, Altavilla G, Cecchetti MG, Fabris G, Grossi MP, Balboni PG, et al. Ependymomas, Malignant Tumors of Pancreatic Islets, and Osteosarcomas Induced in Hamsters by BK Virus, a Human Papovavirus. J Natl Cancer Inst. 1978;61(3):875-83.

1307. Fredholm BB, Lunell NO, Persson B, Wager J. Actions of salbutamol in late pregnancy: Plasma cyclic AMP, insulin and C-peptide, carbohydrate and lipid metabolites in diabetic and non-diabetic women. Diabetologia. 1978;14(4):235-42.

1308. Lunell NO, Wager J, Fredholm BB, Persson B. Metabolic effects of oral salbutamol in late pregnancy. Eur J Clin Pharmacol. 1978;14(2):95-9.

1309. Prosser PR, Karam JH. Diabetes Mellitus Following Rodenticide Ingestion in Man. JAMA: The Journal of the American Medical Association. 1978;239(12):1148-50.

1310. Weber CJ, Hardy MA, Pi-Sunyer FX, Zimmerman E, Reemtsma K. Tissue culture preservation and intramuscular transplantation of pancreatic islets. Surgery. 1978;84(1):166-74.

1311. Cohen MP, Foa PP. Special topics in endocrinology and metabolism. 1979;(I-VIII+144 p.); US$ 18.00.

1312. Madsbad S, Hilsted J, Krarup T. Hormonal, metabolic and cardiovascular responses to hypoglycemia in insulin dependent diabetics with and without beta-cell function. Acta Endocrinologica. 1979;94(Suppl. 237):61.

1313. Nylund L, Lunell NO, Persson B, Fredholm BB, Lagercrantz H. Acute metabolic and circulatory effects of cigarette smoking in late pregnancy. Gynecol Obstet Invest. 1979;10(1):39-45.

1314. Smith-Laing G, Sherlock S, Faber OK. Effects of Spontaneous Portal-Systemic Shunting on Insulin Metabolism. Gastroenterology. 1979;76(4):685-90.

1315. Deckert T. The influence of supervision and endogenous insulin secretion on the course of insulin-dependent diabetes mellitus. Acta Endocrinologica, Supplement. 1980;238:31-8.

1316. Emmanouel DS, Lindheimer MD, Katz AI. Pathogenesis of endocrine abnormalities in uremia. Endocr Rev. 1980;1(1):28-44.

1317. Giugliano D, Luyckx AS, Lefebvre PJ. Plasma C-peptide response to arginine in insulin-dependent diabetic subjects. Journal of Endocrinological Investigation: Official Journal of the Italian Society of Endocrinology. 1980;3(1):19-23.

1318. Gunnarsson R, Arner P, Groth CG, Heding LG, Lundgren G, Ostman J. Plasma C-peptide as an indicator of human pancreatic graft function. Acta Medica Scandinavica, Supplement. 1980;639:53-6.

1319. Sakamoto Y, Fukumitsu S. A Case of Insulin Autoimmune Syndrome Induced by α-Mercaptopropionyl Glycine Administration. Journal of the Japan Diabetes Society. 1980;23(9):889-96.

1320. Tasaka Y, Inoue S, Takei M, Hirata Y. Contents of Insulin and C-Peptide in Diabetic Pancreas and Their Relations to Stability of Fasting Blood Sugar Levels During Diabetic Life. Journal of the Japan Diabetes Society. 1980;23(1):39-45.

1321. Tsubouchi H, Kamibeppu A, Fujisaki K, Nagahama J, Hashimoto S. Hepatic gluconeogenic key enzymes in patients with hepatic cancer. Gastroenterologia Japonica. 1980;15(6):564-9.

1322. Valente U, Ferro M, Barocci S, Campisi C, Parodi F, Cataldi L, et al. Report of clinical cases of human fetal pancreas transplantation. Transplant Proc. 1980;12(4 Suppl. 2):213-7.

1323. Barbieri C, Ferrari C, Caldara R, Crossignani RM, Bertazzoni A. Endocrine and metabolic effects of labetalol in man. Journal of Cardiovascular Pharmacology. 1981;3(5):986-91.

1324. Danowski TS, Ohlsen P, Sharma U. C-peptide reactivity and complications in obese and non-obese insulin-treated diabetes mellitus. Obesity and Metabolism. 1981;1(3):182-92.

1325. Lunell N, Hjemdahl P, Fredholm B, Nisell H, Persson B, Wager J. Circulatory and metabolic effects of a combined alpha‐ and beta‐ adrenoceptor blocker (labetalol) in hypertension of pregnancy. Br J Clin Pharmacol. 1981;12(3):345-8.

1326. Martin MJ, Horwitz DL, Nattrass M, Granger JF, Rochman H, Ash S. Effects of mild hyperinsulinemia on the metabolic response to exercise. Metabolism. 1981;30(7):688-94.

1327. Dhermy P, Mascaro JM. Malherb's tumor (so-called calcified epithelioma) in orbito-palpebral location. Archives d"ophtalmologie et revue generale d"ophtalmologie. 1966;26(1):33-48.

1328. Fadly AM, Winterfield RW, Olander HJ. The oncogenic potential of some avian adenoviruses causing diseases in chickens. Avian Dis. 1976;20(1):139-45.

1329. Hawthorne JN, White DA. Myo-inositol Lipids. Vitamins and Hormones. 1976;33(C):529-73.

1330. Wong PP, Kuo T, Ryan CA. Differential accumulation of proteinase inhibitor I in normal and crown gall tissue of tobacco, tomato, and potato. Plant Physiol. 1976;57(2):214-7.

1331. Sargent Iii T, Budinger TF, Braun G, Shulgin AT. An iodinated catecholamine congener for brain imaging and metabolic studies. J Nucl Med. 1978;19(1):71-6.

1332. Cook RW, Williams JF. Pathology of Taenia taeniaeformis infection in the rat: Gastrointestinal changes. J Comp Pathol. 1981;91(2):205-17.

1333. Dooley JF, Warshawsky D, Yerganian G, Christian RT. CELL-MEDIATED MUTAGENESIS OF A NON-TUMORIGENIC EPITHELIAL-CELL LINE (DPI-3) WITH POLYCYCLIC AROMATIC AND HETEROAROMATIC HYDROCARBONS. In Vitro-Journal of the Tissue Culture Association. 1981;17(3):201-.

1334. Rawyler AJ, Roelofsen B, Wirtz KW, Op den Kamp JA. (poly) Phosphoinositide phosphorylation is a marker for plasma membrane in Friend erythroleukaemic cells. FEBS Lett. 1982;148(1):140-4.

1335. Calnek BW, Schat KA, Ross LJN, Chen CLH. Further characterization of marek's disease virus‐infected lymphocytes. II. In vitro infection. Int J Cancer. 1984;33(3):399-406.

1336. Haley PJ, Hoover EA, Quackenbush SL, Gasper PW, Macy DW. Influence of antibody infusion on pathogenesis of experimental feline leukemia virus infection. J Natl Cancer Inst. 1985;74(4):821-7.

1337. Aberizk WJ, Silver B, Craig Henderson I, Cady B, Harris JR. The use of radiotherapy for treatment of isolated locoregional recurrence of breast carcinoma after mastectomy. Cancer. 1986;58(6):1214-8.

1338. Kato M, Kawai S, Takenawa T. [Role of inositol phospholipids metabolism in the signal transduction of erbB gene product]. Gan To Kagaku Ryoho. 1986;13(3 Pt 2):854-60.

1339. Penn EJ, Hobson C, Rees DA, Magee AI. Structure and assembly of desmosome junctions: biosynthesis, processing, and transport of the major protein and glycoprotein components in cultured epithelial cells. J Cell Biol. 1987;105(1):57-68.

1340. Maly FE, Cross AR, Jones OT, Wolf-Vorbeck G, Walker C, Dahinden CA, et al. The superoxide generating system of B cell lines. Structural homology with the phagocytic oxidase and triggering via surface Ig. J Immunol. 1988;140(7):2334-9.

1341. Bernstein JJ, Goldberg WJ, Laws ER, Jr. Human malignant astrocytoma xenografts migrate in rat brain: a model for central nervous system cancer research. J Neurosci Res. 1989;22(2):134-43.

1342. Coogan TP, Latta DM, Imbra RJ, Costa M. Effect of nickel(II) on DNA-protein interactions. Biological Trace Element Research. 1989;21(1):13-21.

1343. Daugschies A, Jacobs M, Rommel M. Heart failure in a pig chronically infected with Sarcocystis miescheriana--a case report. Berliner und Munchener Tierarztliche Wochenschrift. 1989;102(6):184-7.

1344. Henkler G, Klotzbach M, Koch H, Muller W, Pergande G, Richter J. Progress in the field of drug development, part 22. Pharmazie. 1989;44(4):241-50.

1345. Kornguth SE, Kalinke T, Robins HI, Cohen JD, Turski P. Preferential Binding of Radiolabeled Poly-L-lysines to C6 and U87 MG Glioblastomas Compared with Endothelial Cells in Vitro. Cancer Res. 1989;49(22):6390-5.

1346. Angst BD, Nilles LA, Green KJ. Desmoplakin II expression is not restricted to stratified epithelia. J Cell Sci. 1990;97(2):247-57.

1347. Bernstein JJ, Goldberg WJ, Laws ER, Jr., Conger D, Morreale V, Wood LR. C6 glioma cell invasion and migration of rat brain after neural homografting: ultrastructure. Neurosurgery. 1990;26(4):622-8.

1348. Ebrahim‐Nesbat F, Tiedemann Sv, Albrecht S, Heitefuss R, Hüttermann A. Electron microscopical studies of poplar clones inoculated with Xanthomonas populi subsp. populi. European Journal of Forest Pathology. 1990;20(6-7):367-75.

1349. Höhl B, Pfautsch M, Barz W. Histology of Disease Development in Resistant and Susceptible Cultivars of Chickpea (Cicer arietinum L.) Inoculated with Spores of Ascochyta rabiei. Journal of Phytopathology. 1990;129(1):31-45.

1350. Pollock CA, Allen BJ, Warden RA, Caterson RJ, Blagojevic N, Cocksedge B, et al. Total-Body Nitrogen by Neutron Activation in Maintenance Dialysis. American Journal of Kidney Diseases. 1990;16(1):38-45.

1351. Tochikura TS, Hayes KA, Cheney CM, Tanabe-Tochikura A, Rojko JL, Mathes LE, et al. In vitro replication and cytopathogenicity of the feline immunodeficiency virus for feline T4 thymic lymphoma 3201 cells. Virology. 1990;179(1):492-7.

1352. Cela A, Leong I, Krueger J. Tigliane-type phorbols stimulate human melanocyte proliferation: Potentially safer agents for melanocyte culture. Journal of Investigative Dermatology. 1991;96(6):987-90.

1353. Goldberg WJ, Laws ER, Jr., Bernstein JJ. Individual C6 glioma cells migrate in adult rat brain after neural homografting. Int J Dev Neurosci. 1991;9(4):427-37.

1354. Goldberg WJ, Laws ER, Bernstein JJ. INDIVIDUAL C6 GLIOMA-CELLS MIGRATE IN ADULT-RAT BRAIN AFTER NEURAL HOMOGRAFTING. Int J Dev Neurosci. 1991;9(4):427-&.

1355. Goldberg WJ, Laws Jr ER, Bernstein JJ. Individual C6 glioma cells migrate in adult rat brain after neural homografting. Int J Dev Neurosci. 1991;9(4):427-33,35.

1356. Hancock JT, White JI, Jones OT, Silver IA. The use of diphenylene iodonium and its analogues to investigate the role of the NADPH oxidase in the tumoricidal activity of macrophages in vitro. Free Radic Biol Med. 1991;11(1):25-9.

1357. Hogikyan ND, Hayden RE, McLear PW. Cutaneous photoprotection using a hydroxyl radical scavenger in photodynamic therapy. American Journal of Otolaryngology--Head and Neck Medicine and Surgery. 1991;12(1):1-5.

1358. Leen E, Goldberg JA, Robertson J, Sutherland GR, Hemingway DM, Cooke TG, et al. Detection of hepatic metastases using duplex/color Doppler sonography. Ann Surg. 1991;214(5):599-604.

1359. Leen E, Goldberg JA, Robertson J, Sutherland GR, Hemingway DM, Cooke TG, et al. DETECTION OF HEPATIC METASTASES USING DUPLEX COLOR DOPPLER SONOGRAPHY. Ann Surg. 1991;214(5):599-604.

1360. Leen E, Goldberg JA, Robertson J, Sutherland GR, McArdle CS. The use of duplex sonography in the detection of colorectal hepatic metastases. Br J Cancer. 1991;63(2):323-5.

1361. Gastl G, Finstad CL, Guarini A, Bosl G, Gilboa E, Bander NH, et al. RETROVIRAL VECTOR-MEDIATED LYMPHOKINE GENE-TRANSFER INTO HUMAN RENAL-CANCER CELLS. Cancer Res. 1992;52(22):6229-36.

1362. Grunt THW, Somay C, Oeller H, Dittrich E, Dittrich C. Comparative analysis of the effects of dimethyl sulfoxide and retinoic acid on the antigenic pattern of human ovarian adenocarcinoma cells. J Cell Sci. 1992;103(2):501-9.

1363. Ho KC, Quarmby VE, French FS, Wilson EM. Molecular cloning of rat prostate transglutaminase complementary DNA: The major androgen-regulated protein DP1 of rat dorsal prostate and coagulating gland. J Biol Chem. 1992;267(18):12660-7.

1364. Keller R, Bassetti S, Keist R, Mülsch A, Klauser S. Induction of nitric oxide synthase is a necessary precondition for expression of tumor necrosis factor-independent tumoricidal activity by activated macrophages. Biochem Biophys Res Commun. 1992;184(3):1364-71.

1365. Mokrý J, Nĕmecek S, Adler J. Inoculation of C6 glioma cell suspension into the brain of adult rats: morphological study. Sb Ved Pr Lek Fak Karlovy Univerzity Hradci Kralove. 1992;35(4):293-305.

1366. Ravdin PM, Green S, Dorr TM, McGuire WL, Fabian C, Pugh RP, et al. Prognostic significance of progesterone receptor levels in estrogen receptor-positive patients with metastatic breast cancer treated with tamoxifen: Results of a prospective southwest oncology group study. J Clin Oncol. 1992;10(8):1284-91.

1367. Robertson J, Leen E, Goldberg JA, Angerson WJ, Sutherland GR, McArdle CS. Flow measurement using duplex Doppler ultrasound: haemodynamic changes in patients with colorectal liver metastases. Clin Phys Physiol Meas. 1992;13(4):299-310.

1368. Emmendörffer A, Roesler J, Eisner J, Raeder E, Lohmann‐Matthes ML, Meier B. Production of oxygen radicals by fibroblasts and neutrophils from a patient with x‐linked chronic granulomatous disease. European Journal of Haematology. 1993;51(4):223-7.

1369. Leen E, Goldberg JA, Anderson JR, Robertson J, Moule B, Cooke TG, et al. Hepatic perfusion changes in patients with liver metastases: Comparison with those patients with cirrhosis. Gut. 1993;34(4):554-7.

1370. Leen E, Goldberg JA, Anderson JR, Robertson J, Moule B, Cooke TG, et al. Hepatic perfusion changes in patients with liver metastases: comparison with those patients with cirrhosis. Gut. 1993;34(4):554-7.

1371. Leen E, Goldberg JA, Robertson J, Angerson WJ, Sutherland GR, Cooke TG, et al. Image‐directed doppler ultrasonography: A novel technique for the diagnosis of colorectal liver metastases. J Clin Ultrasound. 1993;21(4):221-30.

1372. Leen E, Goldberg JA, Robertson J, Angerson WJ, Sutherland GR, Cooke TG, et al. Early detection of occult colorectal hepatic metastases using duplex colour Doppler sonography. Br J Surg. 1993;80(10):1249-51.

1373. Leen E, Goldberg JA, Robertson J, Angerson WJ, Sutherland GR, Cooke TG, et al. Image-directed Doppler ultrasonography: a novel technique for the diagnosis of colorectal liver metastases. J Clin Ultrasound. 1993;21(4):221-30.

1374. Leen E, Goldberg JA, Robertson J, Angerson WJ, Sutherland GR, Cooke TG, et al. EARLY DETECTION OF OCCULT COLORECTAL HEPATIC METASTASES USING DUPLEX COLOR DOPPLER SONOGRAPHY. Br J Surg. 1993;80(10):1249-51.

1375. Mourali N, Tabbane F, Muenz LR, Behi J, Moussa FB, Jaziri M, et al. Ten-year results utilizing chemotherapy as primary treatment in nonmetastatic, rapidly progressing breast cancer. Cancer Investigation. 1993;11(4):363-70.

1376. Naftalin RJ, Rist RJ. THE RELATIONSHIP BETWEEN SUGAR METABOLISM, TRANSPORT AND SUPEROXIDE RADICAL PRODUCTION IN RAT PERITONEAL-MACROPHAGES. Biochim Biophys Acta. 1993;1148(1):39-50.

1377. Norian LA, Rosenbusch RF. Mycoplasma bovoculi--augmented bovine natural killer activity. Comp Immunol Microbiol Infect Dis. 1993;16(2):113-22.

1378. Norian LA, Rosenbusch RF. MYCOPLASMA-BOVOCULI - AUGMENTED BOVINE NATURAL-KILLER ACTIVITY. Comp Immunol Microbiol Infect Dis. 1993;16(2):113-22.

1379. Rottenberg ME, Sunnemark D, Leandersson T, Orn A. ORGAN-SPECIFIC REGULATION OF INTERFERON-GAMMA, INTERLEUKIN-2 AND INTERLEUKIN-2 RECEPTOR DURING MURINE INFECTION WITH TRYPANOSOMA-CRUZI. Scand J Immunol. 1993;37(5):559-68.

1380. Stevenhagen A, Vanfurth R. INTERFERON-GAMMA ACTIVATES THE OXIDATIVE KILLING OF CANDIDA-ALBICANS BY HUMAN GRANULOCYTES. Clin Exp Immunol. 1993;91(1):170-5.

1381. Filippone A, Basilico R, Guidotti A, Boni R, Belfiore G, Bonomo L. Color Doppler ultrasonography in the identification and characterization of secondary focal lesions of the liver. Radiol Med. 1994;87(3):283-8.

1382. Goldman R, Ferber E, Meller R, Zor U. A ROLE FOR REACTIVE OXYGEN SPECIES IN ZYMOSAN AND BETA-GLUCAN INDUCED PROTEIN-TYROSINE PHOSPHORYLATION AND PHOSPHOLIPASE A(2) ACTIVATION IN MURINE MACROPHAGES. Biochim Biophys Acta-Mol Cell Res. 1994;1222(2):265-76.

1383. Hall DG, Stoica G. Characterization of brain and bone-metastasizing clones selected from an ethylnitrosourea-induced rat mammary carcinoma. Clin Exp Metastasis. 1994;12(4):283-95.

1384. Junco Petrement E, Madero R. PCR: Relationship with the type of membrane, dialysis dose, buffer and dialysis technique. Nefrologia. 1994;14(SUPPL. 2):131-8.

1385. Leen E, Angerson WJ, Wotherspoon H, Moule B, Cooke TG, McArdle CS. Comparison of the Doppler perfusion index and intraoperative ultrasonography in diagnosing colorectal liver metastases: Evaluation with postoperative follow-up results. Ann Surg. 1994;220(5):663-7.

1386. Riemann D, Göhring B, Langner J. Expression of aminopeptidase N/CD13 in tumour-infiltrating lymphocytes from human renal cell carcinoma. Immunol Lett. 1994;42(1-2):19-23.

1387. Andrianarivo AG, Muiya P, Opollo M, Loganhenfrey LL. Trypanosoma congolense: Comparative Effects of a Primary Infection on Bone Marrow Progenitor Cells from N′Dama and Boran Cattle. Exp Parasitol. 1995;80(3):407-18.

1388. Chan MM, Ho CT, Huang HI. Effects of three dietary phytochemicals from tea, rosemary and turmeric on inflammation-induced nitrite production. Cancer Lett. 1995;96(1):23-9.

1389. Chan MMY, Ho CT, Huang HI. Effects of three dietary phytochemicals from tea, rosemary and turmeric on inflammation-induced nitrite production. Cancer Lett. 1995;96(1):23-9.

1390. Ehrenberg B, Roitman L, Lavi A, Nitzan Y, Malik Z, Sessler JL. Spectroscopic studies of photosensitization in solutions and in cells. Proceedings of SPIE - The International Society for Optical Engineering. 1995;2325:68-79.

1391. Leen E, Angerson WJ, Wotherspoon H, Moule B, Cook TG, McArdle CS. Detection of colorectal liver metastases: comparison of laparotomy, CT, US, and Doppler perfusion index and evaluation of postoperative follow-up results. Radiology. 1995;195(1):113-6.

1392. Leen E, Angerson WJ, Wotherspoon H, Moule B, Cook TG, McArdle CS. DETECTION OF COLORECTAL LIVER METASTASES - COMPARISON OF LAPAROTOMY, CT, US, AND DOPPLER PERFUSION INDEX AND EVALUATION OF POSTOPERATIVE FOLLOW-UP RESULTS. Radiology. 1995;195(1):113-6.

1393. Martin K, Trouche D, Hagemeier C, Sorensen TS, La Thangue NB, Kouzarides T. Stimulation of E2F1/DP1 transcriptional activity by MDM2 oncoprotein. Nature. 1995;375(6533):691-4.

1394. Miesel R, Sanocka D, Kurpisz M, Kroger H. ANTIINFLAMMATORY EFFECTS OF NADPH OXIDASE-INHIBITORS. Inflammation. 1995;19(3):347-62.

1395. Riemann D, Kehlen A, Langner J. Stimulation of the expression and the enzyme activity of aminopeptidase N/CD13 and dipeptidylpeptidase IV/CD26 on human renal cell carcinoma cells and renal tubular epithelial cells by T cell-derived cytokines, such as IL-4 and IL-13. Clin Exp Immunol. 1995;100(2):277-83.

1396. Shibata H, Rai SK, Satoh M, Murakoso K, Sumi K, Uga S, et al. The use of PCR in detecting toxoplasma parasites in the blood and brains of mice experimentally infected with Toxoplasma gondii. Kansenshogaku zasshi The Journal of the Japanese Association for Infectious Diseases. 1995;69(2):158-63.

1397. Surh YJ, Lee RJ, Park KK, Mayne ST, Liem A, Miller JA. Chemoprotective effects of capsaicin and diallyl sulfide against mutagenesis or tumorigenesis by vinyl carbamate and N-nitrosodiinethylamine. Carcinogenesis. 1995;16(10):2467-71.

1398. Thannickal VJ, Fanburg BL. Activation of an H2O2-generating NADH oxidase in human lung fibroblasts by transforming growth factor β1. J Biol Chem. 1995;270(51):30334-8.

1399. Tschickardt ME, Lu Y, Jacim M, Ussery GD, Steimle V, Mach B, et al. RB and a novel E2F-1 binding protein in MHC class II deficient B-cell lines and normal IFN-gamma induction of the class IL transactivator CIITA in class II non-inducible RB-defective tumor lines. Int J Cancer. 1995;62(4):461-5.

1400. Yang J, Luo X, Cai L. Expression of desmoplakin II in tumors. Zhonghua bing li xue za zhi Chinese journal of pathology. 1995;24(3):170-2.

1401. Barham HM, Stratford IJ. Enzymology of the reduction of the novel fused pyrazine mono-N-oxide bioreductive drug, RB90740 roles for P450 reductase and cytochrome b5 reductase. Biochem Pharmacol. 1996;51(6):829-37.

1402. Chan TYK, Critchley JAJH, Chan AYW. An estimate of the incidence of pesticide poisoning in Hong Kong. Veterinary and Human Toxicology. 1996;38(5):362-4.

1403. Gordon LB, Nolan SC, Marling-Berg CJ, Knopf PM. Normal cerebrospinal fluid inhibits development of tumor-specific CTL activity from BALB/C mouse splenocytes following tumor infusion into brain. Faseb J. 1996;10(6):A1471.

1404. Kema GHJ, Yu D, Rijkenberg FHJ, Shaw MW, Baayen RP. Histology of the pathogenesis of Mycosphaerella graminicola in wheat. Phytopathology. 1996;86(7):777-86.

1405. Kiehntocf M, Karawelew L, Herrmann F. Anti-inflammatory glucocortjcoids inhibit apoptosis of human neutrophils in a bcl2 dependent pathway. Experimental Hematology. 1996;24(9):1074.

1406. Kotsonis P, Majewski H. The structural requirements for phorbol esters to enhance noradrenaline and dopamine release from rat brain cortex. British Journal of Pharmacology. 1996;119(1):115-25.

1407. Leen E, Angerson WG, Cooke TG, McArdle CS. Prognostic power of Doppler perfusion index in colorectal cancer - Correlation with survival. Ann Surg. 1996;223(2):199-203.

1408. Lehmann KJ, Walz M, Bode A, Teubner J, Stahl J, Georgi M. Development of a clinical-radiological CD-ROM database for focal lesions of the liver. RoFo Fortschritte auf dem Gebiete der Rontgenstrahlen und der Neuen Bildgebenden Verfahren. 1996;165(4):380-5.

1409. Messina M, Messina V, editors. Nutritional implications of dietary phytochemicals. Advances in Experimental Medicine and Biology; 1996.

1410. Miesel R, Kurpisz M, Kroger H. Suppression of inflammatory arthritis by simultaneous inhibition of nitric oxide synthase and NADPH oxidase. Free Radic Biol Med. 1996;20(1):75-81.

1411. Ng KH, Looi LM, Bradley DA. Microcalcification clustering parameters in breast disease: a morphometric analysis of radiographs of excision specimens. Br J Radiol. 1996;69(820):326-34.

1412. Post C, Ratnasabaoathv R. The soy-derived dietary phytochemical genistein. but not its analog daidzein. stabilizes the apolipoprotein ii mrna in the avian liver by stimulating the hepatic expression of the estrogen-regulated mrna stabilizing factor. Faseb J. 1996;10(3):A620.

1413. Reinhold D, Wrenger S, Bank U, Buhling F, Hoffmann T, Neubert K, et al. CD26 mediates the action of HIV-1 tat protein on DNA synthesis and cytokine production in U937 cells. Immunobiology. 1996;195(1):119-28.

1414. Ruhrberg C, Williamson JA, Sheer D, Watt FM. Chromosomal localisation of the human envoplakin gene (EVPL) to the region of the tylosis oesophageal cancer gene (TOCG) on 17q25. Genomics. 1996;37(3):381-5.

1415. Sodhi MPS, Khanna RNS, Sadana JR, Chand P. Experimental Absidia corymbifera infection in rabbits: Clinicopathological studies. Mycopathologia. 1996;134(1):7-11.

1416. Strayer DS. SV40 as an effective gene transfer vector in vivo. J Biol Chem. 1996;271(40):24741-6.

1417. Vuong PN, Bayssade-Dufour C, Albaret JL, Farhati K. Histopathological observations in new and classic models of experimental Schistosoma haematobiurn infections. Tropical Medicine and International Health. 1996;1(3):348-58.

1418. Carrasco L, deLara FCM, DeLasMulas JM, GomezVillamandos JC, Villeda CJ, Wilkinson PJ. Ultrastructural changes related to the lymph node haemorrhages in acute African swine fever. Res Vet Sci. 1997;62(3):199-204.

1419. Cooper DA, Webb DR, Peters JC. Evaluation of the potential for olestra to affect the availability of dietary phytochemicals. J Nutr. 1997;127(8 Suppl):1699s-709s.

1420. Kitamura F, Saji S, Fukada D, Miya K, Kunieda K, Sugiyama Y, et al. Predicion of metachronous liver metastasis on curatively resected colorectal cancer by immunohistochemical staining of p53, c-erbB-2, PCNA, CD44, and nm23. Japanese Journal of Gastroenterological Surgery. 1997;30(4):838-45.

1421. Leen E, Anderson JR, Robertson J, Ogorman P, Cooke TG, McArdle CS. Doppler index perfusion in the detection of hepatic metastases secondary to gastric carcinoma. Am J Surg. 1997;173(2):99-102.

1422. Lisgaris M, Desai S, Budev M, Rehm S. Outcome of treatment of diabetic foot infections with home intravenous antibiotic therapy (HIVAT). Clin Infect Dis. 1997;25(2):425.

1423. Quade BJ, McLachlin CM, SotoWright V, Zuckerman J, Mutter GL, Morton CC. Disseminated peritoneal leiomyomatosis - Clonality analysis by X chromosome inactivation and cytogenetics of a clinically benign smooth muscle proliferation. Am J Pathol. 1997;150(6):2153-66.

1424. Ricke J, Wolf M, Hosten N, Zielinski C, Liebig T, Lopez-Hänninen E, et al. How safe is teleradiological telediagnosis for CT imaging? RoFo Fortschritte auf dem Gebiete der Rontgenstrahlen und der Neuen Bildgebenden Verfahren. 1997;166(3):243-7.

1425. Sokoloski JA, Hodnick WF, Mayne ST, Cinquina C, Kim CS, Sartorelli AC. Induction of the differentiation of HL-60 promyelocytic leukemia cells by vitamin E and other antioxidants in combination with low levels of vitamin D3: possible relationship to NF-kappaB. Leukemia. 1997;11(9):1546-53.

1426. Watanabe S, Kawamura N, Nakamura M, Katsuoka Y. Digital image processing for evaluation of staging of bladder cancer. British Journal of Urology. 1997;80(SUPPL. 2):54.

1427. Zhang Z, Teel RW. Effects of orally administered capsaicin on hamster lung and liver enzyme activity. Pharmacology Reviews and Communications. 1997;9(4):247-58.

1428. Zippelius A, Kufer P, Honold G, Köllermann MW, Oberneder R, Schlimok G, et al. Limitations of reverse-transcriptase polymerase chain reaction analyses for detection of micrometastatic epithelial cancer cells in bone marrow. J Clin Oncol. 1997;15(7):2701-8.

1429. Baba Y, Furusawa M, Murakami R, Baba T, Yokoyama T, Nishimura R, et al. Optimal image resolution for digital storage of radiotherapy-planning images. Int J Radiat Oncol Biol Phys. 1998;41(4):955-7.

1430. Bennink MR, Om AS. Inhibition of Colon Cancer (CC) by soy phytochemicals but not by soy protein. Faseb J. 1998;12(5):A655.

1431. Buchbinder SS, Leichter IS, Bamberger PN, Novak B, Lederman R, Fields S, et al. Analysis of clustered microcalcifications by using a single numeric classifier extracted from mammographic digital images. Acad Radiol. 1998;5(11 SUPPL. 3):779-84.

1432. Göhring B, Holzhausen HJ, Meye A, Heynemann H, Rebmann U, Langner J, et al. Endopeptidase 24.11/CD10 is down-regulated in renal cell cancer. Int J Mol Med. 1998;2(4):409-14.

1433. Gopalkrishnan RV, Lam EWF, Kedinger C. The p53 tumor suppressor inhibits transcription of the TATA-less mouse DP1 promoter. J Biol Chem. 1998;273(18):10972-8.

1434. Harttig U, Bailey GS. Chemoprotection by natural chlorophylls in vivo: Inhibition of dibenzo[α,l]pyrene-DNA adducts in rainbow trout liver. Carcinogenesis. 1998;19(7):1323-6.

1435. Hihara H, Maeda M, Nakamura K, Ishino S, Tsukamoto K, Yuasa N, et al. Rapid induction of lymphoid leukosis and ascites by avian leukosis virus from a lymphoid leukosis cell line. J Vet Med Sci. 1998;60(1):77-85.

1436. Jankevicius F, Shibayama T, Decken K, Bojar H, Gerharz CD, Ebert T, et al. Dual-parameter immunoflow cytometry in diagnosis and follow-up of patients with bladder cancer. Eur Urol. 1998;34(6):492-9.

1437. Kehlen A, Göhring B, Langner J, Riemann D. Regulation of the expression of aminopeptidase A, aminopeptidase N/CD13 and dipeptidylpeptidase IV/CD26 in renal carcinoma cells and renal tubular epithelial cells by cytokines and cAMP-increasing mediators. Clin Exp Immunol. 1998;111(2):435-41.

1438. Leavens H, Deluyker H, de Kruif A, Koenen F. An experimental infection with classical swine fever virus in weaner pigs: I. transmission of the virus, course of the disease, and antibody response. Veterinary Quarterly. 1998;20(2):41-5.

1439. Lo YYC, Conquer JA, Grinstein S, Cruz TF. Interleukin-1β induction of c-fos and collagenase expression in articular chondrocytes:: Involvement of reactive oxygen species. J Cell Biochem. 1998;69(1):19-29.

1440. Macé K, Offord EA, Harris CC, Pfeifer AM. Development of in vitro models for cellular and molecular studies in toxicology and chemoprevention. Arch Toxicol Suppl. 1998;20:227-36.

1441. Mersseman B, De Wagter C. Characteristics of a commercially available film digitizer and their significance for film dosimetry. Phys Med Biol. 1998;43(6):1803-12.

1442. Pennisi F, Ascanio B, Farina R. Hemodynamic changes in patient with colorectal adenocarcinoma: The role of color Doppler USD. Radiol Med. 1998;95(6):583-7.

1443. Pennisi F, Ascanio B, Farina R. [Hemodynamic changes in patients with colorectal adenocarcinoma: the role of color Doppler US]. Radiol Med. 1998;95(6):583-7.

1444. Piedras P, Hammond-Kosack KE, Harrison K, Jones JDG. Rapid, Cf-9 and Avr9-dependent production of active oxygen species in tobacco suspension cultures. Molecular Plant-Microbe Interactions. 1998;11(12):1155-66.

1445. Surh YJ. Cancer chemoprevention by dietary phytochemicals: A mechanistic viewpoint. Cancer Journal. 1998;11(1):6-10.

1446. Surh YJ, Lee E, Lee JM. Chemoprotective properties of some pungent ingredients present in red pepper and ginger. Mutat Res. 1998;402(1-2):259-67.

1447. Waladkhani AR, Clemens MR. Effect of dietary phytochemicals on cancer development (review). Int J Mol Med. 1998;1(4):747-53.

1448. Walsh KM, Leen E, MacSween RNM, Morris AJ. Hepatic blood flow changes in chronic hepatitis C measured by duplex Doppler color sonography - Relationship to histological features. Dig Dis Sci. 1998;43(12):2584-90.

1449. Zhai S, Dai R, Friedman FK, Vestal RE. Comparative inhibition of human cytochromes P450 1A1 and 1A2 by flavonoids. Drug Metab Dispos. 1998;26(10):989-92.

1450. Zhang Z, Blake DR, Stevens CR, Kanczler JM, Winyard PG, Symons MC, et al. A reappraisal of xanthine dehydrogenase and oxidase in hypoxic reperfusion injury: the role of NADH as an electron donor. Free Radic Res. 1998;28(2):151-64.

1451. Grzegorzewska AE, Mariak I, Dobrowolska-Zachwieja A. Differences in assessment of patients with satisfactory or complicated continuous ambulatory peritoneal dialysis courses. Advances in peritoneal dialysis Conference on Peritoneal Dialysis. 1999;15:116-20.

1452. Kumar GS, Parihar NS. Immunological response to PPD and phosphatide antigens in buffaloes experimentally infected with Mycobacterium bovis. Indian Journal of Animal Sciences. 1999;69(2):73-5.

1453. Leen E. The detection of occult liver metastases of colorectal carcinoma. J Hepatobiliary Pancreat Surg. 1999;6(1):7-15.

1454. Miyazawa K, Kishimoto S, Fukushima S, Takeuchi Y. In vitro antitumor activity, intracellular accumulation, and DNA adduct formation of cis [((1 R, 2 R)-1, 2-cyclohexanediamine-N, N′ bis (myristato)] platinum (II) suspended in Lipiodol. Drug Delivery System. 1999;14(5):401-5.

1455. Ritenbaugh C. Evolutionary perspectives on diet and cancer. Anthropology and Medicine. 1999;6(3):323-36.

1456. Sauer H, Dagdanova A, Hescheler J, Wartenberg M. Redox-regulation of intrinsic prion expression in multicellular prostate tumor spheroids. Free Radic Biol Med. 1999;27(11-12):1276-83.

1457. Shinomiya T, Mori T, Ariyama Y, Sakabe T, Fukuda Y, Murakami Y, et al. Comparative genomic hybridization of squamous cell carcinoma of the esophagus: The possible involvement of the DPI gene in the 13q34 amplicon. Genes Chromosomes and Cancer. 1999;24(4):337-44.

1458. Su TL, Chen CH, Lee YJ. Synthesis and structure-activity relationship studies of cytotoxic acridin-9-one derivatives+. Chinese Pharmaceutical Journal. 1999;51(1):103-15.

1459. Xiao W, Wang L, Ryan JM, H L. Incorporation of an 125I-labeled hexa-iodinated diglyceride analog into low-density lipoprotein and high specific uptake by cells of cervical carcinoma cell lines. Radiation Research. 1999;152(3):250-6.

1460. Zhukov A, Ingelman-Sundberg M. Relationship between cytochrome P450 catalytic cycling and stability: Fast degradation of ethanol-inducible cytochrome P450 2E1 (CYP2E1) in hepatoma cells is abolished by inactivation of its electron donor NADPH-cytochrome P450 reductase. Biochem J. 1999;340(2):453-8.

1461. Agrawal MC, George J, Gupta S. Role of Lymnaea luteola in spreading Schistosoma incognitum in an endemic area. Indian Journal of Animal Sciences. 2000;70(4):349-52.

1462. Arai Y, Nakamura Y, Inoue F, Yamamoto K, Saito K, Furusawa S. Glucocorticoid-induced apoptotic pathways in eosinophils: comparison with glucocorticoid-sensitive leukemia cells. International journal of hematology. 2000;71(4):340-9.

1463. Daniel D, Schlesner J, Wehrstedt K, Hufnagl P, Dietel M, editors. Influence of image resolution on image quality in telepathology. Electronic Journal of Pathology and Histology; 2000.

1464. Dorsam G, Taher MM, Valerie KC, Kuemmerle NB, Chan JC, Franson RC. Diphenyleneiodium chloride blocks inflammatory cytokine-induced up-regulation of group IIA phospholipase A(2) in rat mesangial cells. J Pharmacol Exp Ther. 2000;292(1):271-9.

1465. Jeffrey M, Halliday WG, Bell J, Johnston AR, Macleod NK, Ingham C, et al. Synapse loss associated with abnormal PrP precedes neuronal degeneration in the scrapie-infected murine hippocampus. Neuropathology and Applied Neurobiology. 2000;26(1):41-54.

1466. Kim YH, Lee WJ, Lee JY, Kim SH, Lim HK. Dynamic pulse inversion harmonic imaging of nodular hepatocellular carcinomas using a microbubble contrast agent: Comparison with triphasic helical CT findings. Ultrasound in Medicine and Biology. 2000;26(SUPPL. 2):A63.

1467. Kruger S, Strobel D, Wehler M, Wein A, Hahn EG, Becker D. Hepatic Doppler perfusion index - A sensitive tool for detection of liver metastases? Ultraschall Med. 2000;21(5):206-9.

1468. Lee YL, Shih K, Bao P, Ghirnikar RS, Eng LF, editors. Cytokine chemokine expression in contused rat spinal cord. Neurochemistry International; 2000.

1469. Leen EL, Oppo K, Angerson WJ, McArdle C. Adjuvant chemotherapy following curative surgery of colorectal cancer: Can DPI predict response? Radiology. 2000;217:331-.

1470. Matise I, Sirinarumitr T, Bosworth BT, Moon HW, editors. Ultrastructure and DNA fragmentation analysis of arterioles in swine infected with shiga toxin-producing Escherichia coli. Advances in Experimental Medicine and Biology; 2000.

1471. Orta AJ, Sullivan JT. Short-term immunoisolation of incompatible xenografts in a snail, Biomphalaria glabrata. Developmental and Comparative Immunology. 2000;24(6-7):543-51.

1472. Pani G, Colavitti R, Borrello S, Galeotti T. Endogenous oxygen radicals modulate protein tyrosine phosphorylation and JNK-1 activation in lectin-stimulated thymocytes. Biochem J. 2000;347(1):173-81.

1473. Patrocinio AC, Schiabel H, Benatti RH, Goes CE, Nunes FLS, editors. Investigation of clustered microcalcification features for an automated classifier as part of a mammography CAD scheme. Annual International Conference of the IEEE Engineering in Medicine and Biology - Proceedings; 2000.

1474. Peeters CF, Thomas CM, Sweep FC, Span PN, Wobbes T, Ruers TM. Elevated serum endothelin-1 levels in patients with colorectal cancer; relevance for prognosis. Int J Biol Markers. 2000;15(4):288-93.

1475. Polanowska J, Le Cam L, Orsetti B, Vallés H, Fabbrizio E, Fajas L, et al. Human E2F5 gene is oncogenic in primary rodent cells and is amplified in human breast tumors. Genes Chromosomes and Cancer. 2000;28(1):126-30.

1476. Taiwo VO, Anosa VO. <i>In vitro</i> erythrophagocytosis by cultured macrophages stimulated with extraneous substances and those isolated from the blood, spleen and bone marrow of Boran and N'Dama cattle infected with <i>Trypanosoma congolense</i> and <i>Trypanosoma vivax</i>. Onderstepoort J Vet Res. 2000;67(4):273-87.

1477. Tolando R, Jovanovic A, Brigelius-Flohé R, Ursini F, Maiorino M. Reactive oxygen species and proinflammatory cytokine signaling in endothelial cells: effect of selenium supplementation. Free Radic Biol Med. 2000;28(6):979-86.

1478. Tummala PE, Chen XL, Medford RM. NF- kappa B independent suppression of endothelial vascular cell adhesion molecule-1 and intercellular adhesion molecule-1 gene expression by inhibition of flavin binding proteins and superoxide production. J Mol Cell Cardiol. 2000;32(8):1499-508.

1479. Wang BS, Chen YJ, Liu SH, Lin-Shiau SY. An increase in free radical production by means of an anion channel blocker DIDS in mouse peritoneal neutrophils. Proceedings of the National Science Council, Republic of China Part B, Life sciences. 2000;24(4):178-86.

1480. Abid MR, Tsai JC, Spokes KC, Deshpande SS, Irani K, Aird WC. Vascular endothelial growth factor induces manganese-superoxide dismutase expression in endothelial cells by a Rac1-regulated NADPH oxidase-dependent mechanism. Faseb J. 2001;15(11):2548-+.

1481. Betz A, Lampen N, Martinek S, Young MW, Darnell Jr JE. A Drosophila PIAS homologue negatively regulates stat92E. Proc Natl Acad Sci U S A. 2001;98(17):9563-8.

1482. Blum CA, Xu M, Orner GA, Fong AT, Bailey GS, Stoner GD, et al. β-Catenin mutation in rat colon tumors initiated by 1,2-dimethylhydrazine and 2-amino-3-methylimidazo[4,5-f]quinoline, and the effect of post-initiation treatment with chlorophyllin and indole-3-carbinol. Carcinogenesis. 2001;22(2):315-20.

1483. Dewulf J, Laevens H, Koenen F, Mintiens K, De Kruif A. An experimental infection with classical swine fever virus in pregnant sows: Transmission of the virus, course of the disease, antibody response and effect on gestation. Journal of Veterinary Medicine, Series B. 2001;48(8):583-91.

1484. Ferreira AM, Martins MS, Vitor RW. Virulence for BALB/c mice and antigenic diversity of eight Toxoplasma gondii strains isolated from animals and humans in Brazil. Parasite. 2001;8(2):99-105.

1485. Ghirnikar RS, Lee YL, Eng LF. Chemokine antagonist infusion promotes axonal sparing after spinal cord contusion injury in rat. J Neurosci Res. 2001;64(6):582-9.

1486. Ichinose Y, Andi S, Doi R, Tanaka R, Taguchi F, Sasabe M, et al. Generation of hydrogen peroxide is not required for harpin-induced apoptotic cell death in tobacco BY-2 cell suspension culture. Plant Physiology and Biochemistry. 2001;39(9):771-6.

1487. Kono H, Rusyn I, Uesugi T, Yamashina S, Connor HD, Dikalova A, et al. Diphenyleneiodonium sulfate, an NADPH oxidase inhibitor, prevents early alcohol-induced liver injury in the rat. American Journal of Physiology - Gastrointestinal and Liver Physiology. 2001;280(5 43-5):G1005-G12.

1488. Laudanski P, Dzieciol J, Anchim T, Wolczyński S, editors. The influence of glyco-nitric oxide conjugate on proliferation of breast cancer cells in vitro. Folia Histochem Cytobiol; 2001.

1489. Lobell RB, Omer CA, Abrams MT, Bhimnathwala HG, Brucker MJ, Buser CA, et al. Evaluation of farnesyl: protein transferase and geranylgeranyl:: Protein transferase inhibitor combinations in preclinical models. Cancer Res. 2001;61(24):8758-68.

1490. Mai JC, Mi ZB, Kim SH, Ng B, Robbins PD. A proapoptotic peptide for the treatment of solid tumors. Cancer Res. 2001;61(21):7709-12.

1491. Mastrangelo P, Westaway D. Biology of the prion gene complex. Biochemistry and Cell Biology. 2001;79(5):613-28.

1492. Sauer H, Klimm B, Hescheler J, Wartenberg M. Activation of p90RSK and growth stimulation of multicellular tumor spheroids are dependent on reactive oxygen species generated after purinergic receptor stimulation by ATP. Faseb J. 2001;15(11):2539-+.

1493. Wedgwood S, Dettman RW, Black SM. ET-1 stimulates pulmonary arterial smooth muscle cell proliferation via induction of reactive oxygen species. American Journal of Physiology - Lung Cellular and Molecular Physiology. 2001;281(5 25-5):L1058-L67.

1494. White JE, Tsan MF. Differential induction of TNF-α and MnSOD by endotoxin -: Role of reactive oxygen species and NADPH oxidase. Am J Respir Cell Mol Biol. 2001;24(2):164-9.

1495. Wilmer WA, Dixon CL, Hebert C. Chronic exposure of human mesangial cells to high glucose environments activates the p38 MAPK pathway. Kidney Int. 2001;60(3):858-71.

1496. Benedetto N, Auriault C. Prolactin-cytokine network in defence against Acanthamoeba castellani in murine microglia. Eur Cytokine Netw. 2002;13(4):447-55.

1497. Bengaly Z, Sidibe I, Ganaba R, Desquesnes M, Boly H, Sawadogo L. Comparative pathogenicity of three genetically distinct types of Trypanosoma congolense in cattle: Clinical observations and haematological changes. Veterinary Parasitology. 2002;108(1):1-19.

1498. Choi C, Cho WS, Kim B, Chae C. Expression of interferon-gamma and tumour necrosis factor-alpha in pigs experimentally infected with porcine reproductive and respiratory syndrome virus (PRRSV). J Comp Pathol. 2002;127(2-3):106-13.

1499. Datta A, Nag A, Raychaudhuri P. Differential regulation of E2F1, DP1, and the E2F1/DP1 complex by ARF. Mol Cell Biol. 2002;22(24):8398-408.

1500. Garvey R, McGowran M, editors. Bio 2002 - International Biotechnology Convention and Exhibition: Anti-infectives: The next generation: 9 12 June 2002, Toronto, Canada. IDrugs; 2002.

1501. Haddad JJ. Recombinant human interleukin (IL)-1β-mediated regulation of hypoxia-inducible factor-1α (HIF-1α) stabilization, nuclear translocation and activation requires an antioxidant/reactive oxygen species (ROS)-sensitive mechanism. Eur Cytokine Netw. 2002;13(2):250-60.

1502. Holian O, Wahid S, Atten MJ, Attar BM. Inhibition of gastric cancer cell proliferation by resveratrol: role of nitric oxide. Am J Physiol-Gastroint Liver Physiol. 2002;282(5):G809-G16.

1503. Holy JM. Curcumin disrupts mitotic spindle structure and induces micronucleation in MCF-7 breast cancer cells. Mutat Res Genet Toxicol Environ Mutagen. 2002;518(1):71-84.

1504. Ikegami H, Kajikawa S, Ito K, Nii A, Okamiya H, Nakayama H, et al. Immunohistochemical study on inducible type of nitric oxide (iNOS), basic fibroblast growth factor (bFGF) and tumor growth factor-beta1 (TGF-beta1) in arteritis induced in rats by fenoldopam and theophylline, vasodilators. Exp Toxicol Pathol. 2002;54(1):1-7.

1505. Jyonouchi H, Sun S, Itokazu N. Innate immunity associated with inflammatory responses and cytokine production against common dietary proteins in patients with autism spectrum disorder. Neuropsychobiology. 2002;46(2):76-84.

1506. Lee JE, Austic RE, Naqi SA, Golemboski KA, Dieter RR. Dietary arginine intake alters avian leukocyte population distribution during infectious bronchitis challenge. Poult Sci. 2002;81(6):793-8.

1507. Lindsay DG, editor Workshop on European research on phytochemicals: International Symposium on: Dietary Phytochemicals and Human Health. Salamanca (Spain), April 18-20, 2002. Phytochemistry Reviews; 2002.

1508. Ogunremi OA, Lankester MW, Dergousoff SJ, Gajadhar AA. Detection of anti-Parelaphostrongylus tenuis antibodies in experimentally infected and free-ranging moose (Alces alces). Journal of Wildlife Diseases. 2002;38(4):796-803.

1509. Oleszek W, editor Dietary phytochemicals and human health. Phytochemistry Reviews; 2002.

1510. Paysant JR, Rupin A, Verbeuren TJ. Effect of NADPH oxidase inhibition on E-selectin expression induced by concomitant anoxia/reoxygenation and TNF-α. Endothelium: Journal of Endothelial Cell Research. 2002;9(4):263-71.

1511. Prober DA, Edgar BA. Interactions between Ras1, dMyc, and dPI3K signaling in the developing Drosophila wing. Genes and Development. 2002;16(17):2286-99.

1512. Ratna WN, Simonelli JA. The action of dietary phytochemicals quercetin, catechin, resveratrol and naringenin on estrogen-mediated gene expression. Life Sci. 2002;70(13):1577-89.

1513. Widyma A, Piekarska J. The apoptosis in the course of experimental infection with T. pseudospiralis in mice. Wiadomości parazytologiczne. 2002;48(4):413-7.

1514. Yamakawa T, Tanaka S, Yamakawa Y, Kamei J, Numaguchi K, Motley ED, et al. Lysophosphatidylcholine activates extracellular signal-regulated kinases 1/2 through reactive oxygen species in rat vascular smooth muscle cells. Arterioscler Thromb Vasc Biol. 2002;22(5):752-8.

1515. Zhu BT. Catechol-O-Methyltransferase (COMT)-mediated methylation metabolism of endogenous bioactive catechols and modulation by endobiotics and xenobiotics: importance in pathophysiology and pathogenesis. Curr Drug Metab. 2002;3(3):321-49.

1516. Ajami K, Abbott CA, Obradovic M, Gysbers V, Kähne T, McCaughan GW, et al. Structural requirements for catalysis, expression, and dimerization in the CD26/DPIV gene family. Biochemistry. 2003;42(3):694-701.

1517. Blum CA, Xu M, Orner GA, Darío Díaz G, Li Q, Dashwood WM, et al. Promotion versus suppression of rat colon carcinogenesis by chlorophyllin and chlorophyll: modulation of apoptosis, cell proliferation, and beta-catenin/Tcf signaling. Mutat Res. 2003;523-524:217-23.

1518. Castro GD, de Layño A, Costantini MH, Castro JA. Rat breast microsomal biotransformation of ethanol to acetaldehyde but not to free radicals:: Its potential role in the association between alcohol drinking and breast tumor promotion. Teratogenesis Carcinog Mutagen. 2003:61-70.

1519. Chan HK. Inhalation drug delivery devices and emerging technologies. Expert Opinion on Therapeutic Patents. 2003;13(9):1333-43.

1520. De Biase L, Pignatelli P, Lenti L, Tocci G, Piccioni F, Riondino S, et al. Enhanced TNFα and oxidative stress in patients with heart failure: Effect of TNFα on platelet O2- production. Thromb Haemost. 2003;90(2):317-25.

1521. Díaz GD, Li Q, Dashwood RH. Caspase-8 and apoptosis-inducing factor mediate a cytochrome c-independent pathway of apoptosis in human colon cancer cells induced by the dietary phytochemical chlorophyllin. Cancer Res. 2003;63(6):1254-61.

1522. Duquaine D, Hirsch GA, Chakrabarti A, Han Z, Kehrer C, Brook R, et al. Rapid-onset endothelial dysfunction with adriamycin: Evidence for a dysfunctional nitric oxide synthase. Vascular Medicine. 2003;8(2):101-7.

1523. Hu GG, Linning R, Bakkeren G. Ultrastructural comparison of a compatible and incompatible interaction triggered by the presence of an avirulence gene during early infection of the smut fungus, Ustilago hordei, in barley. Physiological and Molecular Plant Pathology. 2003;62(3):155-66.

1524. Kalkan S, Cevik AA, Cavdar C, Aygoren O, Akgun A, Ergun N, et al. Acute Methanol Poisonings Reported to the Drug and Poison Information Center in Izmir, Turkey. Veterinary and Human Toxicology. 2003;45(6):334-7.

1525. Kehlen A, Lendeckel U, Dralle H, Langner J, Hoang-Vu C. Biological Significance of Aminopeptidase N/CD13 in Thyroid Carcinomas. Cancer Res. 2003;63(23):8500-6.

1526. Koehl J, Oßwald W, Kohn H, Elstner EF, Heiser I. Different responses of two tobacco cultivars and their cell suspension cultures to quercinin, a novel elicitin from Phytophthora quercina. Plant Physiology and Biochemistry. 2003;41(3):261-9.

1527. Kramer JM, Davidge JT, Lockyer JM, Staveley BE. Expression of Drosophila FOXO regulates growth and can phenocopy starvation. BMC Developmental Biology. 2003;3:1-14.

1528. Li NY, Ragheb K, Lawler G, Sturgis J, Rajwa B, Melendez JA, et al. DPI induces mitochondrial superoxide-mediated apoptosis. Free Radic Biol Med. 2003;34(4):465-77.

1529. Lizcano JM, Alrubaie S, Kieloch A, Deak M, Leevers SJ, Alessi DR. Insulin-induced Drosophila S6 kinase activation requires phosphoinositide 3-kinase and protein kinase B. Biochem J. 2003;374(2):297-306.

1530. Meuillet EJ, Mahadevan D, Vankayalapati H, Berggren M, Williams R, Coon A, et al. Specific inhibition of the Akt1 pleckstrin homology domain by D-3-deoxy-phosphatidyl-myo-inositol analogues. Mol Cancer Ther. 2003;2(4):389-99.

1531. Muzaffar S, Jeremy JY, Angelini GD, Stuart-Smith K, Shukla N. Role of the endothelium and nitric oxide synthases in modulating superoxide formation induced by endotoxin and cytokines in porcine pulmonary arteries. Thorax. 2003;58(7):598-604.

1532. Samuilov VD, Lagunova EM, Kiselevsky DB, Dzyubinskaya EV, Makarova YV, Gusev MV. Participation of chloroplasts in plant apoptosis. Bioscience Reports. 2003;23(2-3):103-17.

1533. Sauer H, Wefer K, Vetrugno V, Pocchiari M, Gissel C, Sachinidis A, et al. Regulation of intrinsic prion protein by growth factors and TNF-α: The role of intracellular reactive oxygen species. Free Radic Biol Med. 2003;35(6):586-94.

1534. Shatrov VA, Brüne B. Induced expression of manganese superoxide dismutase by non-toxic concentrations of oxidized low-density lipoprotein (oxLDL) protects against oxLDL-mediated cytotoxicity. Biochem J. 2003;374:505-11.

1535. Tsuruga M, Dang Y, Shiono Y, Oka S, Yamazaki Y. Differential effects of vitamin E and three hydrophilic antioxidants on the actinomycin D-induced and colcemid-accelerated apoptosis in human leukemia CMK-7 cell line. Mol Cell Biochem. 2003;250(1-2):131-7.

1536. Venkata Dayakar B, Lin HJ, Chen CH, Ger MJ, Lee BH, Pai CH, et al. Ferredoxin from sweet pepper (Capsicum annuum L.) intensifying harpinpss-mediated hypersensitive response shows an enhanced production of active oxygen species (AOS). Plant Molecular Biology. 2003;51(6):913-24.

1537. Wang SY, Wang GK. Voltage-gated sodium channels as primary targets of diverse lipid-soluble neurotoxins. Cell Signal. 2003;15(2):151-9.

1538. Wennersten A, Holmin S, Mathiesen T. Characterization of Bax and Bcl-2 in apoptosis after experimental traumatic brain injury in the rat. Acta Neuropathol. 2003;105(3):281-8.

1539. Woo CH, Yoo MH, You HJ, Cho SH, Mun YC, Seong CM, et al. Transepithelial migration of neutrophils in response to leukotriene B<sub>4</sub> is mediated by a reactive oxygen species-extracellular signal-regulated kinase-linked cascade. J Immunol. 2003;170(12):6273-9.

1540. Zhang S, Qin C, Safe SH. Flavonoids as aryl hydrocarbon receptor agonists/antagonists: effects of structure and cell context. Environ Health Perspect. 2003;111(16):1877-82.

1541. Zhang X, Shan P, Sasidhar M, Chupp GL, Flavell RA, Choi AMK, et al. Reactive oxygen species and extracellular signal-regulated kinase 1/2 mitogen-activated protein kinase mediate hyperoxia-induced cell death in lung epithelium. Am J Respir Cell Mol Biol. 2003;28(3):305-15.

1542. Barnes PJ. Asthma guidelines: Recommendations versus reality. Respiratory Medicine. 2004;98(SUPPL. A):S1-S7.

1543. Bodnar RJ, Klein GE. Endogenous opiates and behavior: 2003. Peptides. 2004;25(12):2205-56.

1544. Busse W, Levine B, Andriano K, Lavecchia C, Yegen Ü. Efficacy, tolerability, and effect on asthma-related quality of life of formoterol BID via multidose dry powder inhaler and albuterol QID via metered dose inhaler in patients with persistent asthma: A multicenter, randomized, double-blind, double-dummy, placebo-controlled, parallel-group study. Clinical Therapeutics. 2004;26(10):1587-98.

1545. Carter O, Bailey GS, Dashwood RH, editors. The dietary phytochemical chlorophyllin alters E-cadherin and β-catenin expression in human colon cancer cells. J Nutr; 2004.

1546. Chen W, KuoLee R, Shen H, Bùsa M, Conlan JW. Toll-like receptor 4 (TLR4) does not confer a resistance advantage on mice against low-dose aerosol infection with virulent type A Francisella tularensis. Microb Pathog. 2004;37(4):185-91.

1547. Chen YC, Shen SC, Chow JM, Ko CH, Tseng SW. Flavone inhibition of tumor growth via apoptosis in vitro and in vivo. Int J Oncol. 2004;25(3):661-70.

1548. Dannon EA, Wydra K. Interaction between silicon amendment, bacterial wilt development and phenotype of Ralstonia solanacearum in tomato genotypes. Physiological and Molecular Plant Pathology. 2004;64(5):233-43.

1549. Dodd-O JM, Welsh LE, Salazar JD, Walinsky PL, Peck EA, Shake JG, et al. Effect of NADPH oxidase inhibition on cardiopulmonary bypass-induced lung injury. American Journal of Physiology - Heart and Circulatory Physiology. 2004;287(2 56-2):H927-H36.

1550. Dong JM, Zhao SG, Huang GY, Liu Q. NADPH oxidase-mediated generation of reactive oxygen species is critically required for survival of undifferentiated human promyelocytic leukemia cell line HL-60. Free Radic Res. 2004;38(6):629-37.

1551. Farah MH. Cumulative labeling of embryonic mouse neural retina with bromodeoxyuridine supplied by an osmotic minipump. Journal of Neuroscience Methods. 2004;134(2):169-78.

1552. Gao ZC, Ma MX, Gu LX, Liu J, Wang J, Shen DH. Clinical investigation of application of lung biopsy in the diagnosis of diffuse pulmonary interstitial disease. Zhongguo wei zhong bing ji jiu yi xue = Chinese critical care medicine = Zhongguo weizhongbing jijiuyixue. 2004;16(10):615-7.

1553. Gennari F, Mehta S, Wang Y, St. Clair Tallarico A, Palu G, Marasco WA. Direct Phage to Intrabody Screening (DPIS): Demonstration by isolation of cytosolic intrabodies against the TES1 site of Epstein Barr virus latent membrane protein 1 (LMP1) that block NF-κB transactivation. Journal of Molecular Biology. 2004;335(1):193-207.

1554. Goyal P, Weissmann N, Grimminger F, Hegel C, Bader L, Rose F, et al. Upregulation of NAD(P)H oxidase 1 in hypoxia activates hypoxia-inducible factor 1 via increase in reactive oxygen species. Free Radic Biol Med. 2004;36(10):1279-88.

1555. Gujral JS, Hinson JA, Farhood A, Jaeschke H. NADPH oxidase-derived oxidant stress is critical for neutrophil cytotoxicity during endotoxemia. Am J Physiol-Gastroint Liver Physiol. 2004;287(1):G243-G52.

1556. Higgins RJ, McKisic M, Dickinson PJ, Jimenez DF, Dow SW, Tripp LD, et al. Growth inhibition of an orthotopic glioblastoma in immunocompetent mice by cationic lipid-DNA complexes. Cancer Immunol Immunother. 2004;53(4):338-44.

1557. Jang BC, Paik JH, Jeong HY, Oh HJ, Park JW, Kwon TK, et al. Leptomycin B-induced apoptosis is mediated through caspase activation and down-regulation of Mcl-1 and XIAP expression, but not through the generation of ROS in U937 leukemia cells. Biochem Pharmacol. 2004;68(2):263-74.

1558. Kim YM, Kim HJ, Song EJ, Lee KJ. Glucuronic acid is a novel inducer of heat shock response. Mol Cell Biochem. 2004;259(1-2):23-33.

1559. Kundu JK, Surh YJ. Molecular basis of chemoprevention by resveratrol: NF-kappaB and AP-1 as potential targets. Mutat Res. 2004;555(1-2):65-80.

1560. Laÿ B. Image partitioning and statistics characterize cancer cells & tissues. Advanced Imaging. 2004;19(8):20-3.

1561. Licanin Z, Lincender L, Djurović V, Salihefendić N, Smajlović F. Color Doppler sonography of focal abdominal lesions. Medicinski arhiv. 2004;58(1 Suppl 2):119-21.

1562. Macaldowie C, Maley SW, Wright S, Bartley P, Esteban-Redondo I, Buxton D, et al. Placental pathology associated with fetal death in cattle inoculated with Neospora caninum by two different routes in early pregnancy. J Comp Pathol. 2004;131(2-3):142-56.

1563. Mirza N, Kasper Schwartz S, Antin-Ozerkis D. Laryngeal findings in users of combination corticosteroid and bronchodilator therapy. Laryngoscope. 2004;114(9):1566-9.

1564. Morrey JD, Day CW, Julander JG, Olsen AL, Sidwell RW, Cheney CD, et al. Modeling hamsters for evaluating West Nile virus therapies. Antiviral Research. 2004;63(1):41-50.

1565. Nakayama Y, Su W, Ohguchi A, Nakayama H, Doi K. Experimental encephalomyocarditis virus infection in pregnant mice. Exp Mol Pathol. 2004;77(2):133-7.

1566. Neilan JG, Zsak L, Lu Z, Burrage TG, Kutish GF, Rock DL. Neutralizing antibodies to African swine fever virus proteins p30, p54, and p72 are not sufficient for antibody-mediated protection. Virology. 2004;319(2):337-42.

1567. Nguyen TTT, Tran E, Nguyen TH, Do PT, Huynh TH, Huynh H. The role of activated MEK-ERK pathway in quercetin-induced growth inhibition and apoptosis in A549 lung cancer cells. Carcinogenesis. 2004;25(5):647-59.

1568. Ohlsson M, Mattsson P, Svensson M. A temporal study of axonal degeneration and glial scar formation following a standardized crush injury of the optic nerve in the adult rat. Restorative Neurology and Neuroscience. 2004;22(1):1-10.

1569. Ohnishi H, Asamoto M, Tujimura K, Hokaiwado N, Takahashi S, Ogawa K, et al. Inhibition of cell proliferation by nobiletin, a dietary phytochemical, associated with apoptosis and characteristic gene expression, but lack of effect on early rat hepatocarcinogenesis <i>in vivo</i>. Cancer Sci. 2004;95(12):936-42.

1570. Paperna I, Lainson R. Hepatozoon cf. terzii (Sambon & Seligman, 1907) infection in the snake Boa constrictor constrictor from north Brazil: Transmission to the mosquito Culex quinquefasciatus and the lizard Tropidurus torquatus. Parasite. 2004;11(2):175-81.

1571. Park JS, Choi KS, Jeong EJ, Kwon DH, Benveniste EN, Choi CH. Reactive oxygen species mediate chloroquine-induced expression of chemokines by human astroglial cells. Glia. 2004;47(1):9-20.

1572. Pawate S, Shen Q, Fan F, Bhat NR. Redox regulation of glial inflammatory response to lipopolysaccharide and interferongamma. J Neurosci Res. 2004;77(4):540-51.

1573. Pensaert MB, Sanchez Jr RE, Ladekjær-Mikkelsen AS, Allan GM, Nauwynck HJ, editors. Viremia and effect of fetal infection with porcine viruses with special reference to porcine circovirus 2 infection. Vet Microbiol; 2004.

1574. Rockwell P, Martinez J, Papa L, Gomes E. Redox regulates COX-2 upregulation and cell death in the neuronal response to cadmium. Cell Signal. 2004;16(3):343-53.

1575. Rollwitz J, Lupke M, Simkó M. Fifty-hertz magnetic fields induce free radical formation in mouse bone marrow-derived promonocytes and macrophages. Biochim Biophys Acta-Gen Subj. 2004;1674(3):231-8.

1576. Salguero FJ, Sánchez-Cordón PJ, Sierra MA, Jover A, Núñez A, Gómez-Villamandos JC. Apoptosis of thymocytes in experimental African swine fever virus infection. Histol Histopath. 2004;19(1):77-84.

1577. Scaife RM. G2 cell cycle arrest, down-regulation of cyclin B, and induction of mitotic catastrophe by the flavoprotein inhibitor diphenyleneiodonium. Mol Cancer Ther. 2004;3(10):1229-37.

1578. Scaife RM. G<sub>2</sub> cell cycle arrest, down-regulation of cyclin B, and induction of mitotic catastrophe by the flavoprotein inhibitor diphenyleneiodonium. Mol Cancer Ther. 2004;3(10):1229-37.

1579. Shahidi F, editor Functional foods: Their role in health promotion and disease prevention. Journal of Food Science; 2004.

1580. Shetty K, Wahlqvist ML. A model for the role of the proline-linked pentose-phosphate pathway in phenolic phytochemical bio-synthesis and mechanism of action for human health and environmental applications. Asia Pac J Clin Nutr. 2004;13(1):1-24.

1581. Singh A, Kumari A, Maiti NK, Oberoi MS. Characterization of fowl adenoviruses isolated from field outbreaks of hydropericardium syndrome in Punjab. Indian Journal of Animal Sciences. 2004;74(5):457-61.

1582. Singh RP, Sharma G, Mailikarjuna GU, Dhanalakshmi S, Agarwal C, Agarwal R. In Vivo Suppression of Hormone-Refractory Prostate Cancer Growth by Inositol Hexaphosphate: Induction of Insulin-Like Growth Factor Binding Protein-3 and Inhibition of Vascular Endothelial Growth Factor. Clin Cancer Res. 2004;10(1 I):244-50.

1583. Singh RP, Sharma G, Mallikarjuna GU, Dhanalakshmi S, Agarwal C, Agarwal R. <i>In vivo</i> suppression of hormone-refractory prostate cancer growth by inositol hexaphosphate:: Induction of insulin-like growth factor binding protein-3 and inhibition of vascular endothelial growth factor. Clin Cancer Res. 2004;10(1):244-50.

1584. Sun SS, Han J, Ralph WM, Chandrasekaran A, Liu K, Auborn KJ, et al. Endoplasmic reticulum stress as a correlate of cytotoxicity in human tumor cells exposed to diindolylmethane in vitro. Cell Stress Chaperones. 2004;9(1):76-87.

1585. Wang S, Leonard SS, Ye J, Gao N, Wang L, Shi X. Role of reactive oxygen species and Cr(VI) in Ras-mediated signal transduction. Mol Cell Biochem. 2004;255(1-2):119-27.

1586. Wang SW, Leonard SS, Ye JP, Gao N, Wang LY, Shi XL. Role of reactive oxygen species and Cr(VI) in Ras-mediated signal transduction. Mol Cell Biochem. 2004;255(1-2):119-27.

1587. Weng W, Hasemi Y, Fan W, editors. Experimental and numerical study on fluid structure of gravity current with salt water modeling. Progress in Safety Science and Technology Volume 4:Proceedings of the 2004 International Symposium on Safety Science and Technology; 2004.

1588. Xiang W, Windl O, Wünsch G, Dugas M, Kohlmann A, Dierkes N, et al. Identification of differentially expressed genes in scrapie-infected mouse brains by using global gene expression technology. J Virol. 2004;78(20):11051-60.

1589. Xie Z, Singh M, Singh K. ERK1/2 and JNKs, but not p38 Kinase, Are Involved in Reactive Oxygen Species-Mediated Induction of Osteopontin Gene Expression by Angiotensin II and Interleukin-1β in Adult Rat Cardiac Fibroblasts. J Cell Physiol. 2004;198(3):399-407.

1590. Yama S, Nishioka W, Hirokami Y, Setoguchi R, Takeyama N, Saeki K, et al. Effects of tacrolimus (FK506) on encephalomyocarditic virus-induced diabetes in mice. Microbiol Immunol. 2004;48(1):7-13.

1591. Yamanouchi-Ueno A, Nakayama Y, Doi K. Characteristics of testicular lesions in mice infected with a low dose of encephalomyocarditis (EMC) virus. Exp Mol Pathol. 2004;77(1):72-6.

1592. Zhao M, Wimmer A, Trieu K, Discipio RG, Schraufstatter IU. Arrestin regulates MAPK activation and prevents NADPH oxidase-dependent death of cells expressing CXCR2. J Biol Chem. 2004;279(47):49259-67.

1593. Azzalin A, Del Vecchio I, Chiarelli LR, Valentini G, Comincini S, Ferretti L. Absence of interaction between doppel and GFAP, Grb2, PrPC proteins in human tumor astrocytic cells. Anticancer Res. 2005;25(6 B):4369-74.

1594. Beddhu S, Ramkumar N, Pappas LM. Normalization of protein intake by body weight and the associations of protein intake with nutritional status and survival. Journal of Renal Nutrition. 2005;15(4):387-97.

1595. Cao D, Qiao B, Ge Z, Yuan Y. Amplification loop cascade for increasing caspase activity induced by docetaxel. J Cell Biochem. 2005;96(4):810-20.

1596. Cao DX, Qiao B, Ge ZQ, Yuan YJ. Amplification loop cascade for increasing caspase activity induced by docetaxel. J Cell Biochem. 2005;96(4):810-20.

1597. Cunningham GA, McClenaghan NH, Flatt PR, Newsholme P. L-alanine induces changes in metabolic and signal transduction gene expression in a clonal rat pancreatic β-cell line and protects from pro-inflammatory cytokine-induced apoptosis. Clinical Science. 2005;109(5):447-55.

1598. Dashwood WM, Carter O, Al-Fageeh M, Li QJ, Dashwood RH. Lysosomal trafficking of β-catenin induced by the tea polyphenol epigallocatechin-3-gallate. Mutat Res-Fundam Mol Mech Mutagen. 2005;591(1-2):161-72.

1599. Datta A, Sen J, Hagen J, Korgaonkar CK, Caffrey M, Quelle DE, et al. ARF directly binds DP1:: Interaction with DP1 coincides with the G<sub>1</sub> arrest function of ARF. Mol Cell Biol. 2005;25(18):8024-36.

1600. Ding Y, Chen ZJ, Liu S, Che D, Vetter M, Chang CH. Inhibition of Nox-4 activity by plumbagin, a plant-derived bioactive naphthoquinone. J Pharm Pharmacol. 2005;57(1):111-6.

1601. Ding YX, Chen ZJ, Liu SG, Che DN, Vetter M, Chang CH. Inhibition of Nox-4 activity by plumbagin, a plant-derived bioactive naphthoquinone. J Pharm Pharmacol. 2005;57(1):111-6.

1602. Françoise R, Michels JJ, Plancoulaine B, Herlin P. Optimal resolution for automatic quantification of blood vessels on digitized images of the whole cancer section. Image Analysis and Stereology. 2005;24(1):59-67.

1603. Gorrell MD. Dipeptidyl peptidase IV and related enzymes in cell biology and liver disorders. Clinical Science. 2005;108(4):277-92.

1604. He YY, Huang JL, Block ML, Hong JS, Chignell CF. Role of phagocyte oxidase in UVA-induced oxidative stress and apoptosis in keratinocytes. Journal of Investigative Dermatology. 2005;125(3):560-6.

1605. Hebert C, Siavash H, Norris K, Nikitakis NG, Sauk JJ. Endostatin inhibits nitric oxide and diminishes VEGF and collagen XVIII in squamous carcinoma cells. Int J Cancer. 2005;114(2):195-201.

1606. Kallio A, Zheng A, Dahllund J, Heiskanen KM, Härkönen P. Role of mitochondria in tamoxifen-induced rapid death of MCF-7 breast cancer cells. Apoptosis. 2005;10(6):1395-410.

1607. Kersemans V, Cornelissen B, Bacher K, Kersemans K, Thierens H, Dierckx RA, et al. In vivo evaluation and dosimetry of 123I-2-iodo-D-phenylalanine, a new potential tumor-specific tracer for SPECT, in an R1M rhabdomyosarcoma athymic mouse model. J Nucl Med. 2005;46(12):2104-11.

1608. Kersemans V, Cornelissen B, Kersemans K, Bauwens M, Achten E, Dierckx RA, et al. In vivo characterization of <SUP>123/125</SUP>I-2-iodo-L-phenylalanine in an RIM rhabdomyosarcoma athymic mouse model as a potential tumor tracer for SPECT. J Nucl Med. 2005;46(3):532-9.

1609. Khatri M, Palmquist JM, Cha RM, Sharma JM. Infection and activation of bursal macrophages by virulent infectious bursal disease virus. Virus Res. 2005;113(1):44-50.

1610. Kim BC, Kim HG, Lee SA, Lim S, Park EH, Kim SJ, et al. Genipin-induced apoptosis in hepatoma cells is mediated by reactive oxygen species/c-Jun NH2-terminal kinase-dependent activation of mitochondrial pathway. Biochem Pharmacol. 2005;70(9):1398-407.

1611. LaForce C, Prenner BM, Andriano K, Lavecchia C, Yegen Ü. Efficacy and safety of formoterol delivered via a new multidose dry powder inhaler (Certihaler™) in adolescents and adults with persistent asthma. Journal of Asthma. 2005;42(2):101-6.

1612. Laube BL, editor The expanding role of aerosols in systemic drug delivery, gene therapy, and vaccination. Respiratory Care; 2005.

1613. Lee SC, Hwang BK. Induction of some defense-related genes and oxidative burst is required for the establishment of systemic acquired resistance in Capsicum annuum. Planta. 2005;221(6):790-800.

1614. Nabekura T, Kamiyama S, Kitagawa S. Effects of dietary chemopreventive phytochemicals on P-glycoprotein function. Biochem Biophys Res Commun. 2005;327(3):866-70.

1615. Okamura M, Jameson RA, Kashiwagi H, Hattori T, Hayashizaki N, Sakakibara K, et al., editors. Experimental results of DPIS with a new RFQ. Radiation Effects and Defects in Solids; 2005.

1616. Ponampalam R, editor A centralised Drug and Poison Information Centre. Singapore General Hospital Proceedings; 2005.

1617. Qin LY, Li GR, Qian X, Liu YX, Wu XF, Liu B, et al. Interactive role of the toll-like receptor 4 and reactive oxygen species in LPS-induced microglia activation. Glia. 2005;52(1):78-84.

1618. Roumen RM, Scheltinga MR, Slooter GD, van der Linden AW. Doppler perfusion index fails to predict the presence of occult hepatic colorectal metastases. Eur J Surg Oncol. 2005;31(5):521-7.

1619. Russell WR, Scobbie L, Chesson A. Structural modification of phenylpropanoid-derived compounds and the effects on their participation in redox processes. Bioorganic and Medicinal Chemistry. 2005;13(7):2537-46.

1620. Russo M, Tedesco I, Iacomino G, Palumbo R, Galano G, Russo GL. Dietary phytochemicals in chemoprevention of cancer. Current Medicinal Chemistry: Immunology, Endocrine and Metabolic Agents. 2005;5(1):61-72.

1621. Ryang BS, Matsumoto T, Kobori T, Kosaka Y, Ohki ST. 2b protein is essential to induce a novel gradual cell death in Zucchini yellow mosaic virus-inoculated cucumber cotyledon co-infected with Cucumber mosaic virus. Journal of General Plant Pathology. 2005;71(4):308-13.

1622. Shahidi F, editor Nutraceuticals and functional foods in health promotion and disease prevention. Acta Horticulturae; 2005.

1623. Shahiwala A, Misra A. A preliminary pharmacokinetic study of liposomal leuprolide dry powder inhaler: a technical note. AAPS PharmSciTech. 2005;6(3):E482-6.

1624. Singh RP, Agarwal R. Prostate cancer and inositol hexaphosphate: efficacy and mechanisms. Anticancer Res. 2005;25(4):2891-903.

1625. Traka M, Gasper AV, Smith JA, Hawkey CJ, Bao Y, Mithen RF. Transcriptome analysis of human colon Caco-2 cells exposed to sulforaphane. J Nutr. 2005;135(8):1865-72.

1626. Wang XM, Yu DMT, McCaughan GW, Gorrell MD. Fibroblast activation protein increases apoptosis, cell adhesion, and migration by the LX-2 human stellate cell line. Hepatology. 2005;42(4):935-45.

1627. Wartenberg M, Hoffmann E, Schwindt H, Grünheck F, Petros J, Arnold JR, et al. Reactive oxygen species-linked regulation of the multidrug resistance transporter P-glycoprotein in Nox-1 overexpressing prostate tumor spheroids. FEBS Lett. 2005;579(20):4541-9.

1628. Wartenberg M, Hoffmann E, Schwindt H, Grünheck F, Petros J, Arnold JRS, et al. Reactive oxygen species-linked regulation of the multidrug resistance transporter P-glycoprotein in Nox-1 overexpressing prostate tumor spheroids. FEBS Lett. 2005;579(20):4541-9.

1629. Wilson MJ, Haller R, Li SY, Slaton JW, Sinha AA, Wasserman NF. Elevation of dipeptidylpeptidase IV activities in the prostate peripheral zone and prostatic secretions of men with prostate cancer: Possible prostate cancer disease marker. J Urol. 2005;174(3):1124-8.

1630. Wittwer F, Jaquenoud M, Brogiolo W, Zarske M, Wüstemann P, Fernandez R, et al. Susi, a negative regulator of Drosophila PI3-Kinase. Developmental Cell. 2005;8(6):817-27.

1631. Yamashina S, Takei Y, Ikejima K, Enomoto N, Kitamura T, Sato N. Ethanol-induced sensitization to endotoxin in Kupffer cells is dependent upon oxidative stress. Alcoholism (NY). 2005;29(12):246S-50S.

1632. Ateghang B, Wartenberg M, Gassmann M, Sauer H. Regulation of cardiotrophin-1 expression in mouse embryonic stem cells by HIF-1α and intracellular reactive oxygen species. J Cell Sci. 2006;119(6):1043-52.

1633. Bárta I, Šmerák P, Polívková Z, Šestáková H, Langová M, Turek B, et al. Current trends and perspectives in nutrition and cancer prevention. Neoplasma. 2006;53(1):19-25.

1634. Bobe G, Wang B, Seeram NP, Nair MG, Bourquin LD. Dietary anthocyanin-rich tart cherry extract inhibits intestinal tumorigenesis in APCMin mice fed suboptimal levels of sulindac. J Agric Food Chem. 2006;54(25):9322-8.

1635. Carl-McGrath S, Lendeckel U, Ebert M, Röcken C. Ectopeptidases in tumour biology: A review. Histol Histopath. 2006;21(10-12):1339-53.

1636. Cheng LT, Chen W, Tang W, Wang T. Does loss of residual renal function lead to malnutrition in peritoneal dialysis patients? Clin Nephrol. 2006;66(3):192-201.

1637. Cheranov SY, Jaggar JH. TNF-α dilates cerebral arteries via NAD(P)H oxidase-dependent Ca 2+ spark activation. American Journal of Physiology - Cell Physiology. 2006;290(4):C964-C71.

1638. Cho SO, Kim KH, Yoon JH, Kim H. Signaling for integrin α5/β1 expression in <i>Helicobacter pylori</i>-infected gastric epithelial AGS cells. In: Diederich M, editor. Signal Transduction Pathways, Pt A: Apoptotic and Extracellular Signaling. Annals of the New York Academy of Sciences. 1090. Oxford: Blackwell Publishing; 2006. p. 298-304.

1639. Chow JM, Shen SC, Wu CY, Chen YC. 12-o-Tetradecanoylphorbol 13-acetate prevents baicalein-induced apoptosis via activation of protein kinase C and JNKs in human leukemia cells. Apoptosis. 2006;11(11):1999-2011.

1640. de Galan BE, Simsek S, Tack CJ, Heine RJ. Efficacy and safety of inhaled insulin in the treatment of diabetes mellitus. Netherlands Journal of Medicine. 2006;64(9):319-25.

1641. Fast MD, Muise DM, Easy RE, Ross NW, Johnson SC. The effects of Lepeophtheirus salmonis infections on the stress response and immunological status of Atlantic salmon (Salmo salar). Fish and Shellfish Immunology. 2006;21(3):228-41.

1642. Femling JK, Cherny VV, Morgan D, Rada B, Davis AP, Czirják G, et al. The antibacterial activity of human neutrophils and eosinophils requires proton channels but not BK channels. Journal of General Physiology. 2006;127(6):659-72.

1643. Figueroa JD, Benton RL, Velazquez I, Torrado AI, Ortiz CM, Hernandez CM, et al. Inhibition of EphA7 up-regulation after spinal cord injury reduces apoptosis and promotes locomotor recovery. J Neurosci Res. 2006;84(7):1438-51.

1644. Gupta S, Agrawal MC, Khanna A. Heterologous experimental schistosome infection and fluke recovery from the goats. Indian Journal of Animal Sciences. 2006;76(11):882-5.

1645. Jeronimo J, Long R, Neve L, Ferris D, Noller K, Spitzer M, et al. Preparing digitized cervigrams for colposcopy research and education: determination of optimal resolution and compression parameters. J Low Genit Tract Dis. 2006;10(1):39-44.

1646. Kersemans V, Cornelissen B, Kersemans K, Dierckx RA, De Spiegeleer B, Mertens J, et al. Comparative biodistribution study of the new tumor tracer [ 123I]-2-iodo-L-phenylalanine with [123I]-2-iodo-L-tyrosine. Nucl Med Biol. 2006;33(1):111-7.

1647. Köhle C, Bock KW. Activation of coupled Ah receptor and Nrf2 gene batteries by dietary phytochemicals in relation to chemoprevention. Biochem Pharmacol. 2006;72(7):795-805.

1648. Komine Y, Komine K, Kai K, Itagaki M, Kuroishi T, Aso H, et al. Effect of combination therapy with lactoferrin and antibiotics against staphylococcal mastitis on drying cows. J Vet Med Sci. 2006;68(3):205-11.

1649. Kondrashev S, Okamura M, Jameson R, Kanesue T, Kashiwagi H, Sakakibara K, et al., editors. Acceleration of intense beams of highly-charged ions using direct plasma injection scheme. HB 2006 - 39th ICFA Advanced Beam Dynamics Workshop on High Intensity High Brightness Hadron Beams; 2006.

1650. Kuhar M, Sen S, Singh N. Role of mitochondria in quercetin-enhanced chemotherapeutic response in human non-small cell lung carcinoma H-520 cells. Anticancer Res. 2006;26(2a):1297-303.

1651. Lee JP, Sung MJ, Ha YS, Ji AL, Mi YY, Young HK, et al. The enhanced monocyte adhesiveness after UVB exposure requires ROS and NF-κB signaling in human keratinocyte. Journal of Biochemistry and Molecular Biology. 2006;39(5):618-25.

1652. Li J, Zhang ZG, Ji R, Wang YC, Zheng XB. Hydrogen peroxide regulates elicitor PB90-induced cell death and defense in non-heading Chinese cabbage. Physiological and Molecular Plant Pathology. 2006;67(3-5):220-30.

1653. Maley SW, Buxton D, Macaldowie CN, Anderson IE, Wright SE, Bartley PM, et al. Characterization of the Immune Response in the Placenta of Cattle Experimentally Infected with Neospora caninum in Early Gestation. J Comp Pathol. 2006;135(2-3):130-41.

1654. Mandlekar S, Hong JL, Kong ANT. Modulation of metabolic enzymes by dietary phytochemicals: A review of mechanisms underlying beneficial versus unfavorable effects. Curr Drug Metab. 2006;7(6):661-75.

1655. McGuire SO, Sortwell CE, Shukitt-Hale B, Joseph JA, Hejna MJ, Collier TJ. Dietary supplementation with blueberry extract improves survival of transplanted dopamine neurons. Nutritional Neuroscience. 2006;9(5-6):251-8.

1656. Mochizuki T, Furuta S, Mitsushita J, Shang W, Ito M, Yokoo Y, et al. Inhibition of NADPH oxidase 4 activates apoptosis via the AKT/apoptosis signal-regulating kinase 1 pathway in pancreatic cancer PANC-1 cells. Oncogene. 2006;25(26):3699-707.

1657. O'Gorman GM, Park SDE, Hill EW, Meade KG, Mitchell LC, Agaba M, et al., editors. Cytokine mRNA profiling of peripheral blood mononuclear cells from trypanotolerant and trypanosusceptible cattle infected with Trypanosoma congolense. Physiol Genomics; 2006.

1658. Ochiai T, Nishimura K, Noguchi H, Kitajima M, Tsukada A, Watanabe E, et al. Prognostic impact of orotate phosphoribosyl transferase among 5-fluorouracil metabolic enzymes in resectable colorectal cancers treated by oral 5-fluorouracil-based adjuvant chemotherapy. Int J Cancer. 2006;118(12):3084-8.

1659. Ohori H, Yamakoshi H, Tomizawa M, Shibuya M, Kakudo Y, Takahashi A, et al. Synthesis and biological analysis of new curcumin analogues bearing an enhanced potential for the medicinal treatment of cancer. Mol Cancer Ther. 2006;5(10):2563-71.

1660. Porthouse KH, Chirgwin SR, Coleman SU, Taylor HW, Klei TR. Inflammatory responses to migrating <i>Brugia pahangi</i> third-stage larvae. Infect Immun. 2006;74(4):2366-72.

1661. Psalla D, Psychas V, Spyrou V, Billinis C, Papaioannou N, Vlemmas I. Pathogenesis of experimental encephalomyocarditis: A histopathological, immunohistochemical and virological study in rats. J Comp Pathol. 2006;134(1):30-9.

1662. Qin B, Cartier L, Dubois-Dauphin M, Li B, Serrander L, Krause KH. A key role for the microglial NADPH oxidase in APP-dependent killing of neurons. Neurobiology of Aging. 2006;27(11):1577-87.

1663. Reis K, Hälldin J, Fernaeus S, Pettersson C, Land T. NADPH oxidase inhibitor diphenyliodonium abolishes lipopolysaccharide-induced down-regulation of transferrin receptor expression in N2a and BV-2 cells. J Neurosci Res. 2006;84(5):1047-52.

1664. Rodríguez A, Pérez-Gracia E, Espinosa JC, Pumarola M, Torres J, Ferrer I. Increased expression of water channel aquaporin 1 and aquaporin 4 in Creutzfeldt-Jakob disease and in bovine spongiform encephalopathy-infected bovine-PrP transgenic mice. Acta Neuropathol. 2006;112(5):573-85.

1665. Sánchez-Cordón PJ, Romero-Trevejo JL, Pedrera M, Raya AI, Gómez-Villamandos JC. The Role of B Cells in the Immune Response to Pestivirus (Classical Swine Fever Virus). J Comp Pathol. 2006;135(1):32-41.

1666. Shah S, Chan D, Tear S. Licensing highlights. IDrugs. 2006;9(3):221-6.

1667. Siddiqui IA, Zaman N, Aziz MH, Reagan-Shaw SR, Sarfaraz S, Adhami VM, et al. Inhibition of CWR22Rnu1 tumor growth and PSA secretion in athymic nude mice by green and black teas. Carcinogenesis. 2006;27(4):833-9.

1668. Soon OC, Kyung HK, Yoon JH, Kim H, editors. Signaling for integrin α5/β1 expression in Helicobacter pylori-infected gastric epithelial AGS cells. Annals of the New York Academy of Sciences; 2006.

1669. Spencer JPE, Abd El Mohsen M, Minihane AM. Metabolism of dietary phytochemicals: A review of the metabolic forms identified in humans. Current Topics in Nutraceutical Research. 2006;4(3-4):187-203.

1670. Sulda ML, Abbott CA, Hildebrandt M. DPIV/CD26 and FAP in cancer: a tale of contradictions. Advances in experimental medicine and biology. 2006;575:197-206.

1671. Thakur V, Pritchard MT, McMullen MR, Wang QF, Nagy LE. Chronic ethanol feeding increases activation of NADPH oxidase by lipopolysaccharide in rat Kupffer cells:: role of increased reactive oxygen in LPS-stimulated ERK1/2 activation and TNF-α production. J Leukoc Biol. 2006;79(6):1348-56.

1672. Wang XM, Yu DM, McCaughan GW, Gorrell MD. Extra-enzymatic roles of DPIV and FAP in cell adhesion and migration on collagen and fibronectin. Advances in experimental medicine and biology. 2006;575:213-22.

1673. Yoshida T, Yamagishi Si, Nakamura K, Matsui T, Imaizumi T, Inoue H, et al. Pigment epithelium-derived factor (PEDF) blocks the interleukin-6 signaling to C-reactive protein expression in Hep3B cells by suppressing Rac-1 activation. Life Sci. 2006;79(21):1981-7.

1674. Yu JH, Kim CS, Yoo DG, Song YJ, Joo HK, Kang G, et al. NADPH oxidase and mitochondrial ROS are involved in the TNF-α-induced vascular cell adhesion molecule-1 and monocyte adhesion in cultured endothelial cells. Korean Journal of Physiology and Pharmacology. 2006;10(4):217-22.

1675. Zhang HS, Wang SQ. Salvianolic acid B from Salvia miltiorrhiza inhibits tumor necrosis factor-α (TNF-α)-induced MMP-2 upregulation in human aortic smooth muscle cells via suppression of NAD(P)H oxidase-derived reactive oxygen species. J Mol Cell Cardiol. 2006;41(1):138-48.

1676. Zhang HS, Wang SQ. Notoginsenoside R1 inhibits TNF-α-induced fibronectin production in smooth muscle cells via the ROS/ERK pathway. Free Radic Biol Med. 2006;40(9):1664-74.

1677. Zhang HS, Wang SQ. Salvianolic acid B from Salvia miltiorrhiza inhibits tumor necrosis factor-alpha (TNF-alpha)-induced MMP-2 upregulation in human aortic smooth muscle cells via suppression of NAD(P)H oxidase-derived reactive oxygen species. J Mol Cell Cardiol. 2006;41(1):138-48.

1678. Zhang Z, Bashiruddin JB, Doel C, Horsington J, Durand S, Alexandersen S. Cytokine and toll-like receptor mRNAs in the nasal-associated lymphoid tissues of cattle during foot-and-mouth disease virus infection. J Comp Pathol. 2006;134(1):56-62.

1679. Zhao Y, Chaiswing L, Bakthavatchalu V, Oberley TD, St. Clair DK. Ras mutation promotes p53 activation and apoptosis of skin keratinocytes. Carcinogenesis. 2006;27(8):1692-8.

1680. Zhao YF, Chaiswing L, Bakthavatchalu V, Oherley TD, St Clair DK. Ras mutation promotes p53 activation and apoptosis of skin keratinocytes. Carcinogenesis. 2006;27(8):1692-8.

1681. Zhu QS, Xia L, Mills GB, Lowell CA, Touw IP, Corey SJ. G-CSF induced reactive oxygen species involves Lyn-PI3-kinase-Akt and contributes to myeloid cell growth. Blood. 2006;107(5):1847-56.

1682. Anantharam V, Kaul S, Song C, Kanthasamy A, Kanthasamy AG. Pharmacological inhibition of neuronal NADPH oxidase protects against 1-methyl-4-phenylpyridinium (MPP+)-induced oxidative stress and apoptosis in mesencephalic dopaminergic neuronal cells. NeuroToxicology. 2007;28(5 SPEC. ISS.):988-97.

1683. Becher R, Bucht A, Ovrevik J, Hongslo JK, Dahlman HJ, Samuelsen JT, et al. Involvement of NADPH oxidase and iNOS in rodent pulmonary cytokine responses to urban air and mineral particles. Inhal Toxicol. 2007;19(8):645-55.

1684. Begleiter A, Leith MK, Patel D, Hasinoff BB. Role of NADPH cytochrome P450 reductase in activation of RH1. Cancer Chemother Pharmacol. 2007;60(5):713-23.

1685. Bian ZM, Elner SG, Elner VM. Regulation of VEGF mRNA expression and protein secretion by TGF-β2 in human retinal pigment epithelial cells. Experimental Eye Research. 2007;84(5):812-22.

1686. Biswas S, Gupta MK, Chattopadhyay D, Mukhopadhyay CK. Insulin-induced activation of hypoxia-inducible factor-1 requires generation of reactive oxygen species by NADPH oxidase. American Journal of Physiology - Heart and Circulatory Physiology. 2007;292(2):H758-H66.

1687. de Marco MF, Salguero FJ, Bautista MJ, Núñez A, Sánchez-Cordón PJ, Gómez-Villamandos JC. An immunohistochemical study of the tonsils in pigs with acute African swine fever virus infection. Res Vet Sci. 2007;83(2):198-203.

1688. Fernández de Marco M, Salguero FJ, Bautista MJ, Núñez A, Sánchez-Cordón PJ, Gómez-Villamandos JC. An immunohistochemical study of the tonsils in pigs with acute African swine fever virus infection. Res Vet Sci. 2007;83(2):198-203.

1689. Hong HY, Kim BC. Mixed lineage kinase 3 connects reactive oxygen species to c-Jun NH2-terminal kinase-induced mitochondrial apoptosis in genipin-treated PC3 human prostate cancer cells. Biochem Biophys Res Commun. 2007;362(2):307-12.

1690. Ito S, Chen C, Satoh J, Yim S, Gonzalez FJ. Dietary phytochemicals regulate whole-body CYP1A1 expression through an arylhydrocarbon receptor nuclear translocator-dependent system in gut. Journal of Clinical Investigation. 2007;117(7):1940-50.

1691. Julander JG, Furuta Y, Shafer K, Sidwell RW. Activity of T-1106 in a hamster model of yellow fever virus infection. Antimicrobial Agents and Chemotherapy. 2007;51(6):1962-6.

1692. Kundu JK, Surh YJ. Epigallocatechin gallate inhibits phorbol ester-induced activation of NF-kappa B and CREB in mouse skin: role of p38 MAPK. Ann N Y Acad Sci. 2007;1095:504-12.

1693. KuoLee R, Zhao X, Austin J, Harris G, Conlan JW, Chen W. Mouse model of oral infection with virulent type A Francisella tularensis. Infect Immun. 2007;75(4):1651-60.

1694. Kwon KH, Barve A, Yu S, Huang MT, Kong ANT. Cancer chemoprevention by phytochemicals: Potential molecular targets, biomarkers and animal models. Acta Pharmacol Sin. 2007;28(9):1409-21.

1695. Lagathu C, Eustace B, Prot M, Frantz D, Gu Y, Bastard JP, et al. Some HIV antiretrovirals increase oxidative stress and alter chemokine, cytokine or adiponectin production in human adipocytes and macrophages. Antiviral Therapy. 2007;12(4):489-500.

1696. Lambert IH. Activation and inactivation of the volume-sensitive taurine leak pathway in NIH3T3 fibroblasts and Ehrlich Lettre ascites cells. Am J Physiol Cell Physiol. 2007;293(1):C390-400.

1697. Lepisto AJ, Xu M, Yagita H, Weinberg AD, Hendricks RL. Expression and function of the OX40/OX40L costimulatory pair during herpes stromal keratitis. J Leukoc Biol. 2007;81(3):766-74.

1698. Ma T, Ye Z, Jun Z. The expression and significance of thymidine phosphorylase and dihydropyrimidine dehydrogenase in gastric carcinoma. Chinese Journal of Clinical Oncology. 2007;34(4):28-31.

1699. Maalej N, Asuni G, Al‐dhukair A, Naqvi A. SU‐FF‐I‐71: Measurement of the 2‐D Modulation Transfer Function for Screen‐Film Magnification Mammography. Med Phys. 2007;34(6):2354.

1700. Mahipal SVK, Subhashini J, Reddy MC, Reddy MM, Anilkumar K, Roy KR, et al. Effect of 15-lipoxygenase metabolites, 15-(<i>S</i>)-HPETE and 15-(<i>S</i>)-HETE on chronic myelogenous leukemia cell line K-562:: Reactive oxygen species (ROS) mediate caspase-dependent apoptosis. Biochem Pharmacol. 2007;74(2):202-14.

1701. Manson MM, Foreman BE, Howells LM, Moiseeva EP, editors. Determining the efficacy of dietary phytochemicals in cancer prevention. Biochemical Society Transactions; 2007.

1702. Masamune A, Watanabe T, Kikuta K, Satoh K, Shimosegawa T. NADPH oxidase plays a crucial role in the activation of pancreatic stellate cells. American Journal of Physiology - Gastrointestinal and Liver Physiology. 2007;294(1):G99-G108.

1703. Mehrabanpour MJ, Dadras H, Khodakaram-Tafti A, Rahimian A, Toffan A. Pathological findings of highly pathogenic avian influenza virus A/duck/Vietnam/12/2005 (H5N1) in turkeys. International Journal of Poultry Science. 2007;6(9):679-83.

1704. Meyns T, Maes D, Calus D, Ribbens S, Dewulf J, Chiers K, et al. Interactions of highly and low virulent <i>Mycoplasma hyopneumoniae</i> isolates with the respiratory tract of pigs. Vet Microbiol. 2007;120(1-2):87-95.

1705. Miller MA, Hutchins GD, editors. Development of anatomically realistic PET and PET/CT phantoms with rapid prototyping technology. IEEE Nuclear Science Symposium Conference Record; 2007.

1706. Miller RL, Sun GY, Sun AY. Cytotoxicity of paraquat in microglial cells: Involvement of PKCδ- and ERK1/2-dependent NADPH oxidase. Brain Res. 2007;1167(1):129-39.

1707. Mishra M, Mishra S, Mishra B. A paradigm shift in dry powder inhalation technology. Acta Pharmaceutica Sciencia. 2007;49(3):259-74.

1708. Moiseeva EP, Almeida GM, Jones GDD, Manson MM. Extended treatment with physiologic concentrations of dietary phytochemicals results in altered gene expression, reduced growth, and apoptosis of cancer cells. Mol Cancer Ther. 2007;6(11):3071-9.

1709. Munoz C, Hevezi J, Waggener R. SU‐FF‐J‐96: Development of a Methodology to Determine Dosimetric Accuracy in Moving Tumors Using a CIRS Dynamic Phantom. Med Phys. 2007;34(6):2390.

1710. Murillo MM, Carmona-Cuenca I, Del Castillo G, Ortiz C, Roncero C, Sánchez A, et al. Activation of NADPH oxidase by transforming growth factor-β in hepatocytes mediates up-regulation of epidermal growth factor receptor ligands through a nuclear factor-κB-dependent mechanism. Biochem J. 2007;405:251-9.

1711. Nagao J, editor Distal gastrectomy by distal pouch interposition reconstruction improves QOL. Journal of the Medical Society of Toho University; 2007.

1712. Nair S, Li WG, Kong ANT. Natural dietary anti-cancer chemopreventive compounds: redox-mediated differential signaling mechanisms in cytoprotection of normal cells versus cytotoxicity in tumor cells. Acta Pharmacol Sin. 2007;28(4):459-72.

1713. Nishikawa H, Wakano K, Kitani S. Inhibition of NADPH oxidase subunits translocation by tea catechin EGCG in mast cell. Biochem Biophys Res Commun. 2007;362(2):504-9.

1714. Nitti M, Furfaro AL, Traverso N, Odetti P, Storace D, Cottalasso D, et al. PKC delta and NADPH oxidase in AGE-induced neuronal death. Neurosci Lett. 2007;416(3):261-5.

1715. Nonn L, Duong D, Peehl DM. Chemopreventive anti-inflammatory activities of curcumin and other phytochemicals mediated by MAP kinase phosphatase-5 in prostate cells. Carcinogenesis. 2007;28(6):1188-96.

1716. Park SE, Song JD, Kim KM, Park YM, Kim ND, Yoo YH, et al. Diphenyleneiodonium induces ROS-independent p53 expression and apoptosis in human RPE cells. FEBS Lett. 2007;581(2):180-6.

1717. Prasanna SJ, Saha B, Nandi D. Involvement of oxidative and nitrosative stress in modulation of gene expression and functional responses by IFNγ. International Immunology. 2007;19(7):867-79.

1718. Prawan A, Khor TO, Li W, Kong ANT. Application of pharmacogenomics to dietary cancer chemoprevention. Current Pharmacogenomics. 2007;5(3):198-200.

1719. Qian L, Gao X, Pei Z, Wu X, Block M, Wilson B, et al. NADPH oxidase inhibitor DPI is neuroprotective at femtomolar concentrations through inhibition of microglia over-activation. Parkinsonism and Related Disorders. 2007;13(SUPPL. 3):S316-S20.

1720. Russo GL. Ins and outs of dietary phytochemicals in cancer chemoprevention. Biochem Pharmacol. 2007;74(4):533-44.

1721. Ryan E, Zhang Z, Brooks HW, Horsington J, Brownlie J. Foot-and-Mouth Disease Virus Crosses the Placenta and Causes Death in Fetal Lambs. J Comp Pathol. 2007;136(4):256-65.

1722. Schertel C, Conradt B. C. elegans orthologs of components of the RB tumor suppressor complex have distinct pro-apoptotic functions. Development. 2007;134(20):3691-701.

1723. Stone V, Dean N, Sivasithamparam K, Barbetti MJ. Breaching by a new strain of Leptosphaeria maculans of anatomical barriers in cotyledons of Brassica napus cultivar Surpass 400 with resistance based on a single dominant gene. Journal of General Plant Pathology. 2007;73(5):297-303.

1724. Tang M, Ogawa K, Asamoto M, Hokaiwado N, Seeni A, Suzuki S, et al. Protective effects of citrus nobiletin and auraptene in transgenic rats developing adenocarcinoma of the prostate (TRAP) and human prostate carcinoma cells. Cancer Sci. 2007;98(4):471-7.

1725. Tang MX, Ogawa K, Asamoto M, Hokaiwado N, Seeni A, Suzuki S, et al. Protective effects of citrus nobiletin and auraptene in transgenic rats developing adenocarcinoma of the prostate (TRAP) and human prostate carcinoma cells. Cancer Sci. 2007;98(4):471-7.

1726. Tello-Montoliu A, Moltó JM, López-Hernández N, García-Medina A, Roldán V, Sogorb F, et al. Common carotid artery intima-media thickness and intracranial pulsatility index in non-ST-elevation acute coronary syndromes. Cerebrovascular Diseases. 2007;24(4):338-42.

1727. Thacker TC, Palmer MV, Waters WR. Associations between cytokine gene expression and pathology in Mycobacterium bovis infected cattle. Veterinary Immunology and Immunopathology. 2007;119(3-4):204-13.

1728. Tilton SC, Hendricks JD, Orner GA, Pereira CB, Bailey GS, Williams DE. Gene expression analysis during tumor enhancement by the dietary phytochemical, 3,3'-diindolylmethane, in rainbow trout. Carcinogenesis. 2007;28(7):1589-98.

1729. Tsuji PA, Walle T. Benzo[a]pyrene-induced cytochrome P450 1A and DNA binding in cultured trout hepatocytes - inhibition by plant polyphenols. Chem Biol Interact. 2007;169(1):25-31.

1730. Wang X, Zou Y, Sun A, Xu D, Niu Y, Wang S, et al. Emodin induces growth arrest and death of human vascular smooth muscle cells through reactive oxygen species and p53. Journal of Cardiovascular Pharmacology. 2007;49(5):253-60.

1731. Webb RA, Hoque T, Dimas S. Expulsion of the gastrointestinal cestode, Hymenolepis diminuta by tolerant rats: Evidence for mediation by a Th2 type immune enhanced goblet cell hyperplasia, increased mucin production and secretion. Parasite Immunology. 2007;29(1):11-21.

1732. Xia C, Meng Q, Lin LZ, Rojanasakul Y, Wang XR, Jiang BH. Reactive oxygen species regulate angiogenesis and tumor growth through vascular endothelial growth factor. Cancer Res. 2007;67(22):10823-30.

1733. Zheng YS, Cui ZZ, Zhao P, Li HM, Liu CY, Tian ZJ, et al. Effects of reticuloendotheliosis virus and Marek's Disease virus infection and co-infection on IFN-gamma production in SPF chickens. J Vet Med Sci. 2007;69(2):213-6.

1734. An DJ, Song DS, Park BK. Systemic cytokine profiles of mice vaccinated with naked DNAs encoding six open reading frame antigens of porcine circovirus type 2 (PCV2). Res Vet Sci. 2008;85(3):503-9.

1735. Barh D, Viswanathan G. Syzygium cumini inhibits growth and induces apoptosis in cervical cancer cell lines: a primary study. Ecancermedicalscience. 2008;2:83.

1736. Barve A, Khor TO, Hao X, Keum YS, Yang CS, Reddy B, et al. Murine prostate cancer inhibition by dietary phytochemicals--curcumin and phenyethylisothiocyanate. Pharm Res. 2008;25(9):2181-9.

1737. Biancotti JC, Kumar S, De Vellis J. Activation of inflammatory response by a combination of growth factors in cuprizone-induced demyelinated brain leads to myelin repair. Neurochem Res. 2008;33(12):2615-28.

1738. Bode AM, Dong Z. Modulation of cell signal transduction by tea and ginger. Dietary Modulation of Cell Signaling Pathways2008. p. 45-74.

1739. Breijo M, Anesetti G, Martínez L, Sim RB, Ferreira AM. Echinococcus granulosus: The establishment of the metacestode is associated with control of complement-mediated early inflammation. Exp Parasitol. 2008;118(2):188-96.

1740. Chandor A, Dijols S, Ramassamy B, Frapart Y, Mansuy D, Stuehr D, et al. Metabolic activation of the antitumor drug 5-(aziridin-1-yl)-2,4- dinitrobenzamide (CB1954) by NO synthases. Chem Res Toxicol. 2008;21(4):836-43.

1741. Chieli E, Romiti N. Kidney proximal human tubule HK-2 cell line as a tool for the investigation of P-glycoprotein modulation by natural compounds. Boletin Latinoamericano y del Caribe de Plantas Medicinales y Aromaticas. 2008;7(6):281-94.

1742. Crevel G, Bennett D, Cotterill S. The human TPR protein TTC4 is a putative Hsp90 co-chaperone which interacts with CDC6 and shows alterations in transformed cells. PLoS One. 2008;3(3).

1743. De Carvalho DD, Sadok A, Bourgarel-Rey V, Gattacceca F, Penel C, Lehmann M, et al. Nox1 downstream of 12-lipoxygenase controls cell proliferation but not cell spreading of colon cancer cells. Int J Cancer. 2008;122(8):1757-64.

1744. Diaz LA, Occelli M, Almeida FL, Almirón WR, Contigiani MS. Eared dove (Zenaida auriculata, Columbidae) as host for St. Louis encephalitis virus (Flaviviridae, Flavivirus). Vector-Borne and Zoonotic Diseases. 2008;8(2):277-82.

1745. Donohue JF, Hanania NA, Fogarty C, Campbell SC, Rinehart M, Denis-Mize K. Long-term safety of nebulized formoterol: Results of a twelve-month open-label clinical trial. Therapeutic Advances in Respiratory Disease. 2008;2(4):199-208.

1746. Eggler AL, Gay KA, Mesecar AD. Molecular mechanisms of natural products in chemoprevention: Induction of cytoprotective enzymes by Nrf2. Molecular Nutrition and Food Research. 2008;52(SUPPL. 1):S84-S94.

1747. Erichsen M, Rasmussen CV, Christoffersen JK. Pulmonary embolism with lethal outcome and claims to the Danish Patient Insurance Association. Ugeskrift for Laeger. 2008;170(22):1909-12.

1748. Feagins LA, Hui YZ, Zhang X, Hormi-Carver K, Thomas T, Terada LS, et al. Mechanisms of oxidant production in esophageal squamous cell and Barrett's cell lines. American Journal of Physiology - Gastrointestinal and Liver Physiology. 2008;294(2):G411-G7.

1749. Gopalakrishnan A, Tony Kong AN. Anticarcinogenesis by dietary phytochemicals: Cytoprotection by Nrf2 in normal cells and cytotoxicity by modulation of transcription factors NF-κB and AP-1 in abnormal cancer cells. Food Chem Toxicol. 2008;46(4):1257-70.

1750. Guiti M, Azizian AR, Alam NR, Shakiba M. The Diagnostic Accuracy of Digitized Mammography. Iran J Radiol. 2008;5(2):71-6.

1751. Hsieh TC, Wu JM. Suppression of cell proliferation and gene expression by combinatorial synergy of EGCG, resveratrol and gamma-tocotrienol in estrogen receptor-positive MCF-7 breast cancer cells. Int J Oncol. 2008;33(4):851-9.

1752. Huang DZ, Li KY, Zhang QP, Wu L, Liu JY. Hemodynamic changes and complications after liver transplantation detected by color Doppler ultrasonography and intravenous contrast-enhanced ultrasound. Journal of Clinical Rehabilitative Tissue Engineering Research. 2008;12(5):840-4.

1753. Huang TL, Huang HJ. ROS and CDPK-like kinase-mediated activation of MAP kinase in rice roots exposed to lead. Chemosphere. 2008;71(7):1377-85.

1754. Jin S, Ray RM, Johnson LR. TNF-α/cycloheximide-induced apoptosis in intestinal epithelial cells requires Rac1-regulated reactive oxygen species. American Journal of Physiology - Gastrointestinal and Liver Physiology. 2008;294(4):G928-G37.

1755. Jin S, Ray RM, Johnson LR. TNF-α/cycloheximide-induced apoptosis in intestinal epithelial cells requires Rac1-regulated reactive oxygen species. Am J Physiol-Gastroint Liver Physiol. 2008;294(4):G928-G37.

1756. John T, Caballero OL, Svobodová SJ, Kong A, Chua R, Browning J, et al. ECSA/DPPA2 is an embryo-cancer antigen that Is coexpressed with cancer-testis antigens in non-small cell lung cancer. Clin Cancer Res. 2008;14(11):3291-8.

1757. Julander JG, Bowen RA, Rao JR, Day C, Shafer K, Smee DF, et al. Treatment of Venezuelan equine encephalitis virus infection with (-)-carbodine. Antiviral Research. 2008;80(3):309-15.

1758. Julander JG, Skirpstunas R, Siddharthan V, Shafer K, Hoopes JD, Smee DF, et al. C3H/HeN mouse model for the evaluation of antiviral agents for the treatment of Venezuelan equine encephalitis virus infection. Antiviral Research. 2008;78(3):230-41.

1759. Kim WH, Goo SY, Shin MH, Chun SJ, Lee H, Lee KH, et al. Vibrio vulnificus-induced death of Jurkat T-cells requires activation of p38 mitogen-activated protein kinase by NADPH oxidase-derived reactive oxygen species. Cellular Immunology. 2008;253(1-2):81-91.

1760. Kretlow A, Wang Q, Beekes M, Naumann D, Miller LM. Changes in protein structure and distribution observed at pre-clinical stages of scrapie pathogenesis. Biochimica et Biophysica Acta - Molecular Basis of Disease. 2008;1782(10):559-65.

1761. Lee IT, Wang SW, Lee CW, Chang CC, Lin CC, Luo SF, et al. Lipoteichoic acid induces HO-1 expression via the TLR2/MyD88/c-Src/NADPH oxidase pathway and Nrf2 in human tracheal smooth muscle cells. J Immunol. 2008;181(7):5098-110.

1762. Li C, Barker SJ, Gilchrist DG, Lincoln JE, Cowling WA. Leptosphaeria maculans elicits apoptosis coincident with leaf lesion formation and hyphal advance in Brassica napus. Molecular Plant-Microbe Interactions. 2008;21(9):1143-53.

1763. Lin CY, Chin CH, Wu HH, Chen SH, Ho CW, Ko MT. Hubba: hub objects analyzer - a framework of interactome hubs identification for network biology. Nucleic Acids Res. 2008;36:W438-W43.

1764. Lindh C, Wennersten A, Arnberg F, Holmin S, Mathiesen T. Differences in cell death between high and low energy brain injury in adult rats. Acta Neurochirurgica. 2008;150(12):1269-75.

1765. Longpre JM, Loo G. Paradoxical effect of diphenyleneiodonium in inducing DNA damage and apoptosis. Free Radic Res. 2008;42(6):533-43.

1766. Nisimoto Y, Tsubouchi R, Diebold BA, Qiao SL, Ogawa H, Ohara T, et al. Activation of NADPH oxidase 1 in tumour colon epithelial cells. Biochem J. 2008;415:57-65.

1767. Oh SH, Kim YS, Lim SC, Hou YF, Chang IY, You HJ. Dihydrocapsaicin (DHC), a saturated structural analog of capsaicin, induces autophagy in human cancer cells in a catalase-regulated manner. Autophagy. 2008;4(8):1009-19.

1768. Oh YI, Kim SH, Kim JH, Kang CW, editors. Effects of retinoic acid-induced PKC-δ on the insulin like growth factor-I (IGF-I) system is involved in reactive oxygen species (ROS) in MCF-7 cells. BioMedical Engineering and Informatics: New Development and the Future - Proceedings of the 1st International Conference on BioMedical Engineering and Informatics, BMEI 2008; 2008.

1769. Onoue S, Hashimoto N, Yamada S. Dry powder inhalation systems for pulmonary delivery of therapeutic peptides and proteins. Expert Opinion on Therapeutic Patents. 2008;18(4):429-42.

1770. Ovanesov MV, Moldovan K, Smith K, Vogel MW, Pletnikov MV. Persistent Borna Disease Virus (BDV) infection activates microglia prior to a detectable loss of granule cells in the hippocampus. Journal of Neuroinflammation. 2008;5.

1771. Parkin DR, Lu Y, Bliss RL, Malejka-Giganti D. Inhibitory effects of a dietary phytochemical 3,3′-diindolylmethane on the phenobarbital-induced hepatic CYP mRNA expression and CYP-catalyzed reactions in female rats. Food Chem Toxicol. 2008;46(7):2451-8.

1772. Parkin DR, Lu YJ, Bliss RL, Malejka-Giganti D. Inhibitory effects of a dietary phytochemical 3,3′-diindolylmethane on the phenobarbital-induced hepatic CYP mRNA expression and CYP-catalyzed reactions in female rats. Food Chem Toxicol. 2008;46(7):2451-8.

1773. Prawan A, Kong ANT. Dietary factors in food: Induction of Nrf2-mediated defense genes in normal cells versus inhibition of cell growth genes in tumor cells. Dietary Modulation of Cell Signaling Pathways2008. p. 189-203.

1774. Rani-Beeram S, Meyer K, McCrate A, Hong Y, Nielsen M, Swavey S. A fluorinated ruthenium porphyrin as a potential photodynamic therapy agent: synthesis, characterization, DNA binding, and melanoma cell studies. Inorg Chem. 2008;47(23):11278-83.

1775. Riccioni G, Mancini B, Di Ilio E, Bucciarelli T, D'Orazio N. Protective effect of lycopene in cardiovascular disease. Eur Rev Med Pharmacol Sci. 2008;12(3):183-90.

1776. Roy M, Sinha D, Mukherjee S, Paul S, Bhattacharya RK. Protective effect of dietary phytochemicals against arsenite induced genotoxicity in mammalian V79 cells. Indian J Exp Biol. 2008;46(10):690-7.

1777. Sánchez-Cordón PJ, Romero-Trevejo JL, Pedrera M, Sánchez-Vizcaíno JM, Bautista MJ, Gómez-Villamandos JC. Role of hepatic macrophages during the viral haemorrhagic fever induced by African Swine Fever Virus. Histol Histopath. 2008;23(6):683-91.

1778. Satsu H, Hiura Y, Mochizuki K, Hamada M, Shimizu M. Activation of pregnane X receptor and induction of MDR1 by dietary phytochemicals. J Agric Food Chem. 2008;56(13):5366-73.

1779. Singh J, Yao M, Jardine G, Dong Q. A dietary phytochemical cocktail impedes prostate cancer growth in vitro and in vivo. EJC Suppl. 2008;6(9):129-.

1780. Skugor S, Glover KA, Nilsen F, Krasnov A. Local and systemic gene expression responses of Atlantic salmon (Salmo salar L.) to infection with the salmon louse (Lepeophtheirus salmonis). BMC Genomics. 2008;9.

1781. Son TG, Camandola S, Mattson MP. Hormetic dietary phytochemicals. Neuromolecular Med. 2008;10(4):236-46.

1782. Song JD, Kim KM, Kim KH, Kim CD, Kim JM, Yoo YH, et al. Differential role of diphenyleneiodonium, a flavoenzyme inhibitor, on p53-dependent and -independent cell cycle progression. Int J Oncol. 2008;33(6):1299-306.

1783. Sun J, Rui HL. Apple phytochemical extracts inhibit proliferation of estrogen-dependent and estrogen-independent human breast cancer cells through cell cycle modulation. J Agric Food Chem. 2008;56(24):11661-7.

1784. Surh YJ, Kundu JK, Na HK. Nrf2 as a master redox switch in turning on the cellular signaling involved in the induction of cytoprotective genes by some chemopreventive phytochemicals. Planta Med. 2008;74(13):1526-39.

1785. Takahashi M, Hoshino Y, Tanaka T, Takahashi H, Nishizawa T, Okamoto H. Production of monoclonal antibodies against hepatitis E virus capsid protein and evaluation of their neutralizing activity in a cell culture system. Archives of Virology. 2008;153(4):657-66.

1786. Tashkin DP, Rennard SI, Martin P, Ramachandran S, Martin UJ, Silkoff PE, et al. Efficacy and safety of budesonide and formoterol in one pressurized metered-dose inhaler in patients with moderate to very severe chronic obstructive pulmonary disease: Results of a 6-month randomized clinical trial. Drugs. 2008;68(14):1975-2000.

1787. Traore K, Sharma R, Thimmulappa RK, Watson WH, Biswal S, Trush MA. Redox-regulation of ERK 1/2-directed phosphatase by reactive oxygen species: Role in signaling TPA-induced growth arrest in ML-1 cells. J Cell Physiol. 2008;216(1):276-85.

1788. Venail F, Bonafe A, Poirrier V, Mondain M, Uziel A. Comparison of Echo-planar diffusion-weighted imaging and delayed postcontrast T1-weighted MR imaging for the detection of residual cholesteatoma. American Journal of Neuroradiology. 2008;29(7):1363-8.

1789. Venkatachalam P, de Toledo S, Pandey B, Tephly L, Carter A, Little J, et al. Regulation of normal cell cycle progression by flavin-containing oxidases. Oncogene. 2008;27(1):20-31.

1790. Wang J, Li L, Cang H, Shi G, Yi J. NADPH oxidase-derived reactive oxygen species are responsible for the high susceptibility to arsenic cytotoxicity in acute promyelocytic leukemia cells. Leuk Res. 2008;32(3):429-36.

1791. Wang YJ, Lin MW, Lin AA, Peng H, Wu SN. Evidence for state-dependent block of DPI 201-106, a synthetic inhibitor of Na<SUP>+</SUP> channel inactivation, on delayed-rectifier K<SUP>+</SUP> current in pituitary tumor (GH<sub>3</sub>) cells. J Physiol Pharmacol. 2008;59(3):409-23.

1792. Xin MW, Yao TW, Nadvi NA, Osborne B, McCaughan GW, Gorrell MD. Fibroblast activation protein and chronic liver disease. Frontiers in Bioscience. 2008;13(8):3168-80.

1793. Yoon HJ, Jeon SB, Kim IH, Park EJ. Regulation of TLR2 expression by prostaglandins in brain glia. J Immunol. 2008;180(12):8400-9.

1794. Zhao Y, Liu J, McMartin KE. Inhibition of NADPH oxidase activity promotes differentiation of B16 melanoma cells. Oncol Rep. 2008;19(5):1225-30.

1795. Zhao YF, Liu JF, McMartin KE. Inhibition of NADPH oxidase activity promotes differentiation of B16 melanoma cells. Oncol Rep. 2008;19(5):1225-30.

1796. Aviv Y, Etzion S, Win T, Bittner S, Granot Y. TW96, a synthetic 1,4-naphthoquinone, differentially regulates vascular and endothelial cells survival. Vascular Pharmacology. 2009;51(4):225-35.

1797. Barh D. Biomarkers, critical disease pathways, drug targets, and alternative medicine in male breast cancer. Current Drug Targets. 2009;10(1):1-8.

1798. Borchi E, Parri M, Papucci L, Becatti M, Nassi N, Nassi P, et al. Role of NADPH oxidase in H9c2 cardiac muscle cells exposed to simulated ischaemia-reperfusion. J Cell Mol Med. 2009;13(8 B):2724-35.

1799. Daghestani HN, Fernig DG, Day BW. Evaluation of biosensor surfaces for the detection of microtubule perturbation. Biosensors and Bioelectronics. 2009;25(1):136-41.

1800. Das I, Saha T. Effect of garlic on lipid peroxidation and antioxidation enzymes in DMBA-induced skin carcinoma. Nutrition. 2009;25(4):459-71.

1801. Durand SV, Hulst MM, de Wit AA, Mastebroek L, Loeffen WL. Activation and modulation of antiviral and apoptotic genes in pigs infected with classical swine fever viruses of high, moderate or low virulence. Archives of virology. 2009;154(9):1417-31.

1802. Firestone GL, Sundar SN. Minireview: Modulation of hormone receptor signaling by dietary anticancer indoles. Molecular Endocrinology. 2009;23(12):1940-7.

1803. George TW, Niwat C, Waroonphan S, Gordon MH, Lovegrove JA, Paterson E. Effects of chronic and acute fruit and vegetable juice consumption on cardiovascular disease risk factors. Acta Horticulturae. 2009;841:201-6.

1804. Gwathmey JK, Tsaioun K, Hajjar RJ. Cardionomics: A new integrative approach for screening cardiotoxicity of drug candidates. Expert Opinion on Drug Metabolism and Toxicology. 2009;5(6):647-60.

1805. Hattori T, Ito T, Hayashizaki N, Ishibashi T, Lu L, Tamura J, et al., editors. C6+ ion hybrid single cavity linac with direct plasma injection scheme for cancer therapy. Proceedings of the 24th Linear Accelerator Conference, LINAC 2008; 2009.

1806. Hsieh TC, Wu JM. Targeting CWR22Rv1 prostate cancer cell proliferation and gene expression by combinations of the phytochemicals EGCG, genistein and quercetin. Anticancer Res. 2009;29(10):4025-32.

1807. Jacques C, Soustelle L, Nagy I, Diebold C, Giangrande A. A novel role of the glial fate determinant glial cells missing in hematopoiesis. International Journal of Developmental Biology. 2009;53(7):1013-22.

1808. Juhasz A, Ge Y, Holbeck S, Shoemaker R, Roy K, Doroshow J. Distinct pharmacologic mechanisms of action of the flavin dehydrogenase inhibitors diphenylene iodonium (DPI) and di-2-thienyliodonium (DTI) based on NCI60 tumor cell line panel profiling. Cancer Res. 2009;69:1.

1809. Julander JG, Smee DF, Morrey JD, Furuta Y. Effect of T-705 treatment on western equine encephalitis in a mouse model. Antiviral Research. 2009;82(3):169-71.

1810. Kennedy BC, Maier LM, D'Amico R, Mandigo CE, Fontana EJ, Waziri A, et al. Dynamics of central and peripheral immunomodulation in a murine glioma model. BMC Immunol. 2009;10.

1811. Kreis W. Secondary plant products and cancer. Deutsche Zeitschrift fur Onkologie. 2009;41(3):100-8.

1812. Kumar S, Buza JJ, Burgess SC. Genotype-dependent tumor regression in Marek's disease mediated at the level of tumor immunity. Cancer Microenviron. 2009;2(1):23-31.

1813. Kundu JK, Surh YJ. Molecular basis of chemoprevention with dietary phytochemicals: Redox-regulated transcription factors as relevant targets. Phytochemistry Reviews. 2009;8(2):333-47.

1814. Lau JS, Lee PK, Tsang KH, Ng CH, Lam YW, Cheng SH, et al. Luminescent cyclometalated iridium(III) polypyridine indole complexes--synthesis, photophysics, electrochemistry, protein-binding properties, cytotoxicity, and cellular uptake. Inorg Chem. 2009;48(2):708-18.

1815. Lee YS. Arachidonic Acid Activates K<SUP>+</SUP>-Cl<SUP>-</SUP>-cotransport in HepG2 Human Hepatoblastoma Cells. Korean J Physiol Pharmacol. 2009;13(5):401-8.

1816. Leung HY, Yung LH, Poon CH, Shi G, Lu AL, Leung LK. Genistein protects against polycyclic aromatic hydrocarbon-induced oxidative DNA damage in non-cancerous breast cells MCF-10A. British Journal of Nutrition. 2009;101(2):257-62.

1817. Leung HY, Yung LH, Shi G, Lu AL, Leung LK. The red wine polyphenol resveratrol reduces polycyclic aromatic hydrocarbon-induced DNA damage in MCF-10A cells. Br J Nutr. 2009;102(10):1462-8.

1818. Lin RJ, Cheng MJ, Huang JC, Lo WL, Yeh YT, Yen CM, et al. Cytotoxic Compounds from the Stems of <i>Cinnamomum tenuifolium</i>. J Nat Prod. 2009;72(10):1816-24.

1819. Martín R, Hernández M, Ibeas E, Fuentes L, Salicio V, Arnés M, et al. Secreted phospholipase A<sub>2</sub>-IIA modulates key regulators of proliferation on astrocytoma cells. J Neurochem. 2009;111(4):988-99.

1820. Mishra MK, Ghosh D, Duseja R, Basu A. Antioxidant potential of Minocycline in Japanese Encephalitis Virus infection in murine neuroblastoma cells: correlation with membrane fluidity and cell death. Neurochem Int. 2009;54(7):464-70.

1821. Moiseeva EP, Manson MM. Dietary chemopreventive phytochemicals: too little or too much? Cancer Prev Res (Phila). 2009;2(7):611-6.

1822. Naikwade SR, Bajaj AN, Gurav P, Gatne MM, Singh Soni P. Development of budesonide microparticles using spray-drying technology for pulmonary administration: Design, characterization, in vitro evaluation, and in vivo efficacy study. AAPS PharmSciTech. 2009;10(3):993-1012.

1823. Nakamura Y, Yogosawa S, Izutani Y, Watanabe H, Otsuji E, Sakai T. A combination of indol-3-carbinol and genistein synergistically induces apoptosis in human colon cancer HT-29 cells by inhibiting Akt phosphorylation and progression of autophagy. Mol Cancer. 2009;8:100.

1824. Palomeque J, Rueda OV, Sapia L, Valverde CA, Salas M, Petroff MV, et al. Angiotensin II-induced oxidative stress resets the Ca2+ dependence of Ca2+-calmodulin protein kinase II and promotes a death pathway conserved across different species. CircRes. 2009;105(12):1204-12.

1825. Park SY, Choi ES, Hwang J, Kim D, Ryu TK, Lee TK. Physiological and biochemical responses of Prorocentrum minimum to high light stress. Ocean Science Journal. 2009;44(4):199-204.

1826. Piekarska J, Szczypka M, Obmińska-Mrukowicz B, Gorczykowski M. Effect of phytohaemagglutinin-P on apoptosis and necrosis in Trichinella spiralis infected mice. Veterinary Parasitology. 2009;159(3-4):240-4.

1827. Pitman MR, Sulda ML, Kuss B, Abbott CA. Dipeptidyl peptidase 8 and 9 - Guilty by association? Frontiers in Bioscience. 2009;14(10):3619-33.

1828. Qian JS, Pang RP, Zhu KS, Liu DY, Li ZR, Deng CY, et al. Static Pressure Promotes Rat Aortic Smooth Muscle Cell Proliferation via Upregulation of Volume-Regulated Chloride Channel. Cell Physiol Biochem. 2009;24(5-6):461-70.

1829. Qian L, Hu XM, Zhang D, Snyder A, Wu HM, Li YC, et al. β2 Adrenergic Receptor Activation Induces Microglial NADPH Oxidase Activation and Dopaminergic Neurotoxicity Through an ERK-Dependent/Protein Kinase A-Independent Pathway. Glia. 2009;57(15):1600-9.

1830. Redondo E, Masot AJ, Fernández A, Gázquez A. Histopathological and Immunohistochemical Findings in the Lungs of Pigs Infected Experimentally with Mycoplasma hyopneumoniae. J Comp Pathol. 2009;140(4):260-70.

1831. Reiterová K, Antolová D, Hurníková Z. Humoral immune response of mice infected with low doses of Trichinella spiralis muscle larvae. Veterinary Parasitology. 2009;159(3-4):232-5.

1832. Roman-Campos D, Duarte HLL, Sales Jr PA, Natali AJ, Ropert C, Gazzinelli RT, et al. Changes in cellular contractility and cytokines profile during Trypanosoma cruzi infection in mice. Basic Research in Cardiology. 2009;104(3):238-46.

1833. Saito T, Watanabe C, Takemae N, Chaisingh A, Uchida Y, Buranathai C, et al. Pathogenicity of highly pathogenic avian influenza viruses of H5N1 subtype isolated in Thailand for different poultry species. Vet Microbiol. 2009;133(1-2):65-74.

1834. Sanmun D, Witasp E, Jitkaew S, Tyurina YY, Kagan VE, Åhlin A, et al. Involvement of a functional NADPH oxidase in neutrophils and macrophages during programmed cell clearance: Implications for chronic granulomatous disease. American Journal of Physiology - Cell Physiology. 2009;297(3):C621-C31.

1835. Scofield VL, Yan M, Kuang X, Kim SJ, Crunk D, Wong PK. The drug monosodium luminol (GVT) preserves thymic epithelial cell cytoarchitecture and allows thymocyte survival in mice infected with the T cell-tropic, cytopathic retrovirus ts1. Immunol Lett. 2009;122(2):159-69.

1836. Srivastava R, Kalita J, Khan MY, Misra UK. Free radical generation by neurons in rat model of Japanese encephalitis. Neurochem Res. 2009;34(12):2141-6.

1837. Tosetti F, Noonan DM, Albini A. Metabolic regulation and redox activity as mechanisms for angioprevention by dietary phytochemicals. Int J Cancer. 2009;125(9):1997-2003.

1838. Umekawa T, Tsuji H, Uemura H, Khan SR. Superoxide from NADPH oxidase as second messenger for the expression of osteopontin and monocyte chemoattractant protein-1 in renal epithelial cells exposed to calcium oxalate crystals. BJU International. 2009;104(1):115-20.

1839. Wang W, Masaaki A, Zhou J. Effect of ROS on parthenolide-induced apoptosis in multiple myeloma cell. Chinese Pharmacological Bulletin. 2009;25(1):92-4.

1840. Wipf P, Arnold D, Carter K, Dong SZ, Johnston PA, Sharlow E, et al. A Case Study from the Chemistry Core of the Pittsburgh Molecular Library Screening Center: The Polo-like Kinase Polo-Box Domain (Plk1-PBD). Curr Top Med Chem. 2009;9(13):1194-205.

1841. Won SY, Choi SH, Jin BK. Prothrombin kringle-2-induced oxidative stress contributes to the death of cortical neurons in vivo and in vitro: Role of microglial NADPH oxidase. Journal of Neuroimmunology. 2009;214(1-2):83-92.

1842. Xavier CPR, Lima CF, Preto A, Seruca R, Fernandes-Ferreira M, Pereira-Wilson C. Luteolin, quercetin and ursolic acid are potent inhibitors of proliferation and inducers of apoptosis in both KRAS and BRAF mutated human colorectal cancer cells. Cancer Lett. 2009;281(2):162-70.
[truncated: 363,293 more chars]
