# Supplementary material for: Dietary phytochemical index and the risk of cancer: A systematic review and meta-analysis
Source: PLoS One. 2025 Apr 2;20(4):e0319591. doi: 10.1371/journal.pone.0319591 (PMC11964270; doi:10.1371/journal.pone.0319591)
Supplement: S6 Table — (DOCX) [file pone.0319591.s006.docx]

**Table S6:** The risk of bias assessment of case-control studies using ROBINS-I tool.

| **Study/Year** | **Bias due to confounding** | **Bias due to selection of participants** | **Bias due to exposure assessment** | **Bias due to misclassification during follow-up** | **Bias due to missing data** | **Bias due to measurement of the outcome** | **Bias due to selective reporting of the results** | **Overall judgement** |
| --- | --- | --- | --- | --- | --- | --- | --- | --- |
| Bahadoran, 2013 | Moderate | Low | Low | - | Low | Low | Low | Moderate |
| Aghababayan,2019 | Moderate | Low | Low | - | Low | Low | Low | Moderate |
| Ghoreishy, 2021 | Moderate | Low | Low | - | Low | Low | Low | Moderate |
| Rigi, 2021 | Moderate | Low | Low | - | Low | Low | Low | Moderate |
| Pinar, 2022 | Moderate | Moderate | Low | - | Low | Low | Low | Moderate |
| Bentyaghoob, 2023 | Moderate | Low | Low | - | Low | Low | Low | Moderate |
| Mousavi, 2024 | Moderate | Low | Low | - | Low | Low | Low | Moderate |
| Mahmoodi, 2024 | Moderate | Moderate | Low | - | Low | Low | Low | Moderate |
