# Supplementary material for: Dietary phytochemical index and the risk of cancer: A systematic review and meta-analysis
Source: PLoS One. 2025 Apr 2;20(4):e0319591. doi: 10.1371/journal.pone.0319591 (PMC11964270; doi:10.1371/journal.pone.0319591)
Supplement: S7 Table — (DOCX) [file pone.0319591.s007.docx]

**Table S7.** Sensitivity analyses after removing any study in the association between dietary phytochemical index with the risk of cancers.

| **Study omitted** | **e^coef (95% confidence interval)** |
| --- | --- |
| Aghababayan | 0.36 (0.26 – 0.50) |
| Bahadoran | 0.36 (0.27 – 0.50) |
| Bentyaghoob | 0.35 (0.25 – 0.49) |
| Ghoreishy (Premenopausal) | 0.33 (0.24 – 0.46) |
| Ghoreishy (Postmenopausal) | 0.34 (0.22 – 0.51) |
| Rigi | 0.34 (0.25 – 0.48) |
| Pinar | 0.35 (0.26 – 0.48) |
| Mousavi | 0.38 (0.27 – 0.52) |
| Mahmoodi | 0.36 (0.26 – 0.48) |
| Combined | 0.40 (0.29 – 0.54) |
