# Supplementary material for: Dietary phytochemical index and the risk of cancer: A systematic review and meta-analysis
Source: PLoS One. 2025 Apr 2;20(4):e0319591. doi: 10.1371/journal.pone.0319591 (PMC11964270; doi:10.1371/journal.pone.0319591)
Supplement: S1 Fig — (DOCX) [file pone.0319591.s008.docx]

**Figure S1.** Egger's funnel plot (with pseudo 95% confidence interval) depicting log RR (relative risk) against their corresponding standard error for assessing the presence of publication bias in studies that investigated the association between dietary phytochemical index (DPI) with the cancer risk.

Egger's publication bias plot

Standardized effect

Precision

0

2

4

-4

-2

0

2
